# Parametric Life Cycle Assessment of Nuclear Power for Simplified Models

**DOI:** 10.1021/acs.est.3c03190

**Published:** 2023-09-12

**Authors:** Thomas Gibon, Álvaro Hahn Menacho

**Affiliations:** † Luxembourg Institute of Science and Technology, 4362 Esch-sur-Alzette, Luxembourg; ‡ Paul Scherrer Institute, 5232 Villigen, Switzerland

**Keywords:** life cycle assessment, nuclear power, parametric LCA

## Abstract

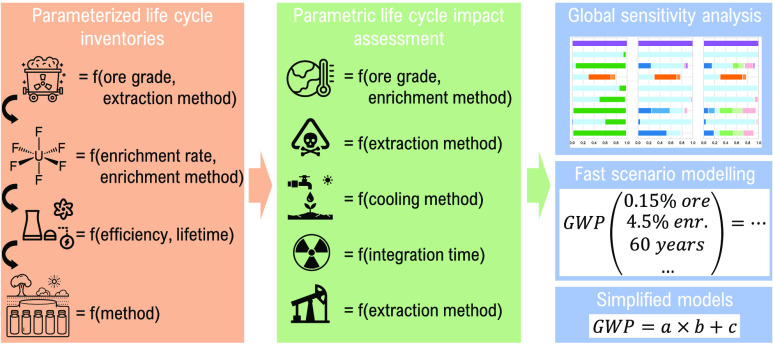

Electrifying the global economy is accepted as a main decarbonization lever to reach the Paris Agreement targets. The IEA’s 2050 Net Zero transition pathways all involve some degree of nuclear power, highlighting its potential as a low-carbon electricity source. Greenhouse gas emissions of nuclear power reported in the life cycle assessment literature vary widely, from a few grams of CO_2_ equivalents to more than 100 g/kWh, globally. The reasons for such a variation are often misunderstood when reported and used by policymakers. To fill this gap, one can make LCA models explicit, exploring the role of the most significant parameters, and develop simplified models for the scientific community, policymakers, and the public. We developed a parametric cradle-to-grave life cycle model with 20 potentially significant variables: ore grade, extraction technique, enrichment technique, and power plant construction requirements, among others. Average GHG emissions of global nuclear power in 2020 are found to be 6.1 g CO_2_ equiv/kWh, whereas pessimistic and optimistic scenarios provide extreme values of 5.4–122 g CO_2_ equiv/kWh. We also provide simplified models, one per environmental impact indicator, which can be used to estimate environmental impacts of electricity generated by a pressurized water reactor without running the full-scale model.

## Abstract 2

Environmental impacts of nuclear power are influenced by a limited number of parameters, allowing the creation of simplified LCA models.

## 1 Introduction

### 1.1 Motivation

Electricity generation has become key to solving the dilemma of increased energy demand while reducing greenhouse gas (GHG) emissions. An extensive review of decarbonization scenarios by the IPCC^1^ shows that more than 50% (median value) of final energy needs to be covered by electricity by 2050 (from 20% in 2021) if we are to respect the Paris Agreement target of limiting warming “well below” 2 °C. Simultaneously, this electricity will need to be generated by low-carbon technologies, such as wind power, photovoltaics, hydropower, geothermal power, combustion technologies with carbon capture and storage, or nuclear power. In 2021, the latter represented 10% of electricity production globally, 25% in the European Union, and up to 69% in France. Nuclear power is both low-carbon and dispatchable,^2^ which makes it a suitable candidate for a decarbonized portfolio, but is not devoid of controversies, namely, the multihazard risk and magnitude of power plant accidents,^3^ potential impacts of uranium mining,^4^ the feasibility of long-term storage of highly radioactive waste,^5^ the potential contribution of that technology to decarbonize national grids,^6−8^ or nuclear proliferation risks.^9^

Because of these challenges, the share of nuclear power in global, long-term energy scenarios varies widely across energy modeling exercises. Examples of nuclear-free scenarios abound: Hansen et al.^10^ have identified 181 studies of 100% renewable electricity scenarios, several of which address the global power system and show the feasibility of attaining a fully renewable grid by 2050.^11−17^ On the other hand, although somewhat rarer, global nuclear-rich scenarios are also regularly proposed.^18−20^ Sepulveda et al.^18^ show that achieving deep decarbonization without firm low-carbon capacity appears highly challenging from an economic viewpoint. Nuclear power plays a role in all “Net Zero by 2050” scenarios of the IEA.^19^ Duan et al.^20^ propose a least-cost optimization framework to devise decarbonization scenarios in which nuclear power is competitive under favorable capital cost assumptions.

Questions about the actual low-carbon characteristics of nuclear power are also regularly raised.^21^ Across nuclear-using countries, national environmental agencies and other official bodies report widely different GHG emission factors: 6 g CO_2_ equiv/kWh in France,^22^ 6.4 in the UK,^23^ 10–20 g CO_2_ equiv/kWh in Switzerland,^24^ 13 g CO_2_ equiv/kWh in the US,^25^ or 67.8 g CO_2_ equiv/kWh in Germany.^26^ Bruckner et al.^27^ provide a range of 4–110 g CO_2_ equiv/kWh, largely based on a life cycle assessment review by Warner and Heath.^28^ The complexity of nuclear fuel supply chains, as well as plant construction or waste management, makes it challenging to pinpoint the influence of the options and assumptions made at every life cycle phase related to the delivery of 1 kWh of nuclear power. Furthermore, non-GHG environmental indicators are seldom put forward, yet some can prove critical (water usage, ionizing radiation) or show lower impacts than alternatives (materials, land use).^29^ As the energy landscape becomes increasingly dependent on resource scarcity and geopolitics, understanding the environmental profile of nuclear power becomes essential. First, identifying the most influential parameters in the life cycle assessment (LCA) of nuclear power with a parametric model, and second, developing simplified models for various impact indicators based on a selection of these parameters—instead of proposing ranges of values—can support decision-making in energy policy. Through building parameterized life cycle inventories for each step of the nuclear power supply chain, we aim here at offering such simplified models.

The objectives of the present study include (1) confirming previously identified parameters as influential for LCA results of nuclear power and (2) producing simplified models for a selection of indicators, analyzing sensitivity, and validating the models.

### 1.2 State of the Art

This section provides a review of LCA studies including nuclear power, specifically light water reactors. Two previous review efforts were merged,^28,30^ to which a list of recent publications was added. Warner and Heath^28^ compiled 99 independent estimates of life cycle GHG emissions from 27 LCA studies on light water reactors, with the aim of harmonizing results, for the following parameters: capacity factor, lifetime, thermal efficiency, inclusion of waste handling, and/or facility construction/decommissioning. Among challenges met along the harmonization process, the authors report unspecified mining methods, unreported ore grade, no decommissioning details, or no mine rehabilitation included. Kadiyala et al.^30^ gathered 50 estimates, of which 24 overlap with ref (28), the remaining was either excluded at the screening phase in ref (28) or not available yet at the time of publication. In addition to these 125 single estimates, 18 more were collected from more recent publications.^31−37^ The literature review from ref (38) was excluded as it does not provide each study’s characteristics in a systematic manner, apart from the source, year, and default life cycle GHG emission value (or a minimum–maximum range) which did not allow for cross-parameter comparison. The full compilation (including ref (38)) of 275 published estimates is available in the Supporting Information (SI).

**Figure 1:**
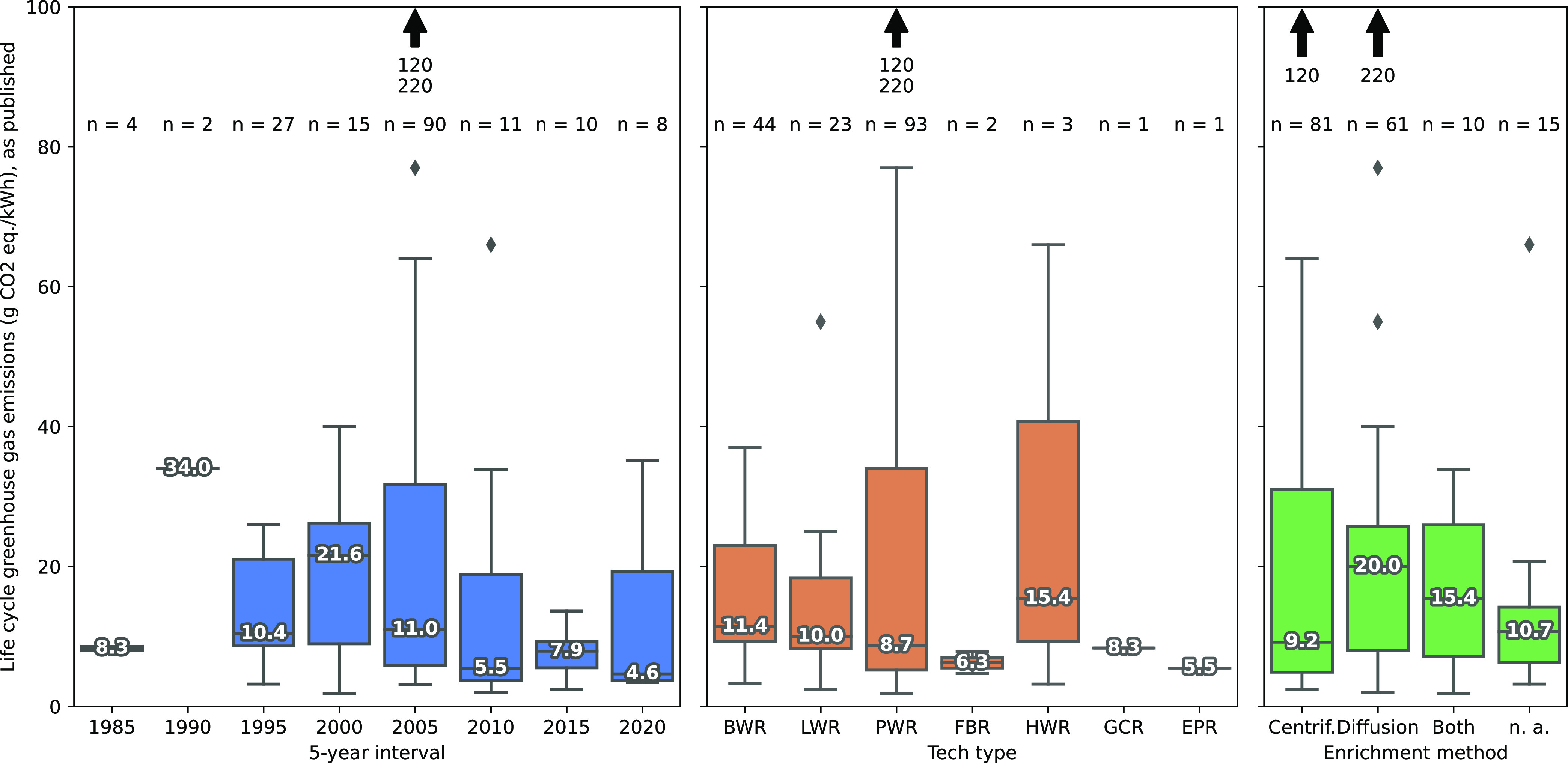
Life cycle greenhouse gas emission values from the literature review (*n* = 167), by 5-year interval of publication date, reactor technology type, and enrichment method. Median, sample size, and potential outliers are provided for each series of each category. BWR = boiling water reactor, LWR = light water reactor (unspecified BWR or PWR), PWR = pressurized water reactor, FBR = fast breeder reactor, HWR = heavy water reactor, GCR = gas-cooled reactor, and EPR = evolutionary power reactor.

The retained categories to classify the results of the preliminary literature review were 5-year interval of publication date, reactor technology, and enrichment method, as shown in Figure 1. A first observation is that the median GHG values decrease slightly with every 5-year period, which can be explained by three main effects: (1) a gradual switch to a more low-carbon background, (2) the increasing share of pressurized water reactor (PWR) in analyzed technologies, and (3) a growing share of centrifugation in the enrichment mix. All median values from 2010 and later fall within 4.2–7.9 g CO_2_ equiv/kWh, which is significantly lower than earlier reported results (median values for the 1990–2005 period range from 10.4 to 34.0 g CO_2_ equiv/kWh). The only pre-1990 results consist of two reports not disclosing full calculations.^39,40^ Light water reactors represent the majority of LCA studies, with PWRs showing slightly lower life cycle GHG emissions than boiling water reactors (BWR), with median values of 8.7 and 11.4 g CO_2_ equiv, respectively. Regarding enrichment, the literature review shows that centrifugation is associated with lower life cycle GHG emissions than gaseous diffusion, with 9.2 and 20.0 g CO_2_ equiv/kWh, respectively.

The influence of these parameters has been clearly identified in prior reviews and is confirmed by the present update. Note that, to keep the consistency with previous literature reviews, data points have not been weighted: it could indeed be argued that several data points originating from the same study are often variations of the same model, and therefore should not weigh as much as a single data point from another given study. For example, Lenzen^41^ provides 21 life cycle GHG emissions values, some of which are simple variations of the same model. This model would therefore have more weight in the literature review than a model used to calculate a single value. The full list of reviewed sources is available in the SI.

## 2 Methods

### 2.1 Goal and Scope

In the present case, the functional unit can be defined as “generating 1 kWh of high-voltage electricity from a pressurized water reactor.” The nuclear reactor and uranium fuel chain are modeled to be representative of the 2020 global situation (or the most recent year available). The system associated with this functional unit is represented in Figure S1 (SI).

### 2.2 Life Cycle Inventory Building

For each life cycle step, data was collected with the following procedure, in order of priority: direct elicitation from World Nuclear Association (WNA) experts, academic literature, technical literature, and finally, the ecoinvent 3.8 database as a fallback. The life cycle inventories were built in spreadsheets, which run with *brightway2*, an LCA Python module.^42^ In addition, the *lca_algebraic* module was used to parameterize the inventories.^43^ The spreadsheet and Jupyter notebooks are available in the SI. The main default parameters of each life cycle phase are listed in Table 1. Data and sources are provided in Section 3, together with the details of the parameterization process.

**Table 1:** Structuring Constants Used as Default Parameter Values for the Life Cycle Inventory

constants	parameter	unit	value	source
mining	waste-to-ore ratio		5	(44)
	ore grade	t U/t ore	0.21%	(45)
		t U_3_0_8_/t ore	0.25%	(45)
milling	extraction losses		4.05%	(44)
conversion	losses		0.00%	(44)
enrichment	enrichment rate		4.15%	(45)
	tails assay		0.22%	(45)
	cut	kg U/kg U	0.12	calculated from^45^
	SWU per kg feed	SWU/kg	0.82	calculated from^45^
	SWU per kg product	SWU/kg	6.67	calculated from^45^
fuel fabrication	losses		0%	WNA consultation
	SWU per kg fuel	SWU/kg	6.74	WNA consultation
power plant	burnup rate	GW-day/ton	42	WNA consultation
	efficiency		34%	WNA consultation
	nameplate capacity	MW	1000	WNA consultation
	lifetime	years	60	WNA consultation

### 2.3 Life Cycle Impact Assessment

Whereas greenhouse gas emissions are the staple indicator of all LCA studies reviewed in Section 1.2, we select nine environmental impact categories following the selection process recommended in Zampori and Pant:^46^ climate change, freshwater eutrophication, ionizing radiation, human toxicity, freshwater ecotoxicity, land use, water resource depletion, as well as mineral, and nonrenewable resource depletion. These additional indicators help to grasp the multidimensionality of environmental sustainability.^47^

### 2.4 Impact Variability and Key Parameters

Following the method in refs (43, 48) a global sensitivity analysis (GSA) is carried out to compute Sobol indices^49^ and identify the variables of the parameterized LCI that contribute the most to the variance of the result for each impact category. GSA and Sobol indices to account for LCA results variability caused by the input parameters have gained increasing ground.^50−52^ The first step to conduct GSA based on Sobol indices requires the use of Monte Carlo simulations to vary all input parameters simultaneously, thus overcoming the limitations of one-at-a-time analysis methods. In the present case, only the uncertainty of the parameterized activities are accounted for, the uncertainty of other inventories is not propagated, and the result of the GSA is therefore a lower bound of the total uncertainty of the model. The *lca_algebraic* module was used to perform the Monte Carlo simulations and to compute Sobol indices. The first-order Sobol indices are formally defined as
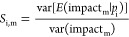
where *S*_i,m_ is the first-order Sobol index of the parameter *p*_i_ for impact_m_. By definition, first-order Sobol indices sum to a value lower or equal to one—the latter occurs if the model is purely additive, but there are likely interactions between parameters. Higher-order interactions can also be calculated but are not presented here.

## 3 Parameterization of the LCI

### 3.1 Mining and Milling

The uranium fuel chain starts with the extraction of uranium ore. According to the literature survey, mining technique^28^ and the energy mix of the mine itself^53^ are key characteristics. The ore grade of the uranium deposit at the mining site also appears significant, although no clear trend can be extracted from site-specific data;^28,54,55^ uncertainty remains therefore important.

Mining techniques include three main categories: open-cast mining, underground mining, and in situ leaching (ISL). Uranium can also be obtained as a byproduct from other mining activities or by heap leaching, but this is not included in this study. There are fundamental differences between the three mining techniques, chiefly: energy requirements per ton of ore, land use, chemical use, and milling requirements (which ISL does not have, as yellowcake is a direct product of that production pathway).

Ore grade is the parameter with the highest variation: the lowest economically viable mines’ ore contains about 300 ppm of uranium (0.03%),^56^ whereas at the other end of the spectrum, some Canadian mines offer ore grades up to 20%.^57^ Including noneconomically viable sites, estimating this value is equivalent to estimating the expected amount of uranium ore in the Earth’s crust, i.e., about 3 ppm (0.0003%).^58−60^ Economically viable mines are found to have an average uranium tenor of 0.15%,^61^ a value widely used in nuclear power LCA literature.^55,62,63^ The distribution of available tonnage with respect to ore grade can be estimated from the World Distribution of Uranium Deposits (UDEPO) database maintained by the IAEA. According to Monnet et al.,^61^ the best fit for this distribution (over the UDEPO database) is a log-normal curve of average 1544 ppm and a standard deviation of 1299 ppm. These values are retained to model the ore grade distribution process in the life cycle inventory. In addition, the relationship between recovery rate and ore grade is defined below (Parker 2016).
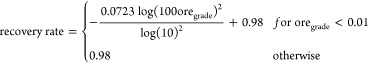


Mining energy mix is deemed an influential parameter.^53^ Mining energy inputs include heat from diesel or propane, as well as electricity from diesel generators or the grid on some occasions. Depending on the grid electricity mix, the latter option may decrease the GHG footprint of mining (and milling) significantly.

Open cast and underground mines are equipped with a mill that crushes extracted ore for grounds to be leached in sulfuric acid tanks. The solution obtained is then used to recover the uranium as yellowcake (U_3_O_8_).^64^ After milling, yellowcake is then transported to a conversion facility, and the tailings are stored in a final repository. We assume natural attenuation instead of active remediation of site. Tests have been carried out at the Irkol deposit in Kazakhstan, showing that “in four years, the ISL-affected area had reduced by half, and after 12 years, it was fully restored naturally.” More densely populated areas require that groundwater be restored to baseline standards, and newer mines even include a water restoration circuit by design.^65^ In terms of energy requirements, values for milling are extracted from ref (62).

### 3.2 Conversion

Conversion involves a series of processes aiming at producing uranium hexafluoride (UF_6_) from yellowcake and other chemicals. Up to this stage, the share of uranium-235 (^235^U) in the uranium product is about 0.7% (its natural abundance). The global conversion market is shared between a few sites; we assume here that all plants are supplied by this global market, namely, from CNNC (China), Rosatom (Russia), Cameco (Canada), and Orano (France). Another company, ConverDyn, represents 12% of global capacity but has been idle for several years. These various shares are gathered from World Nuclear Association;^66^ electricity consumption is matched with local electricity mixes. At this stage, electricity and heat inputs are the sole parameters—meaning that the electricity mixes remain unchanged.

### 3.3 Enrichment

The main parameter at this stage is the enrichment mix. A main difference between gaseous diffusion and centrifugation, the two major commercial routes historically, is their electricity inputs; power consumption is set at 2500 kWh/SWU (range 2400–3000^67^) for gaseous diffusion and 50 kWh/SWU (range 40–100^67^) for centrifugation, respectively, parameterized with triangular distributions. In the full model, the enrichment mix is conservatively left as a mix, namely, with 80% of centrifugation and 20% of gaseous diffusion. This is required to understand the influence of the enrichment technique on overall results. Fixed enrichment (to either technique) is also tested in Section 4.

### 3.4 Fuel Fabrication

Manufacturing the fuel elements as used in the operation phase consists of producing uranium dioxide (UO_2_) from enriched UF_6_ (or UO_3_, not modeled here), conditioning it in pellets, and encasing the pellets in fuel rods, usually with zirconium alloy (“zircalloy”). Finally, fuel rods are assembled into a “fuel assembly.” According to the WNA, a full PWR core “may contain 193 fuel assemblies composed of over 50 000 fuel rods and some 18 million fuel pellets.”^68^ Little data is available on this phase, and the only parameter is the electricity consumption for fuel fabrication of 36 kWh/kg fuel element (range 36–50 kWh, from ref (69) and expert elicitation).

### 3.5 Plant Construction, Operation, and Decommissioning

Plant construction requirements may span wide ranges, especially in terms of material inputs. Figure S4 (in the SI) shows ranges found in literature from various sources.^33,69−73^ To consider the variability in material and energy inputs during the construction phase, average values were retained and then multiplied by an “intensity factor” (range 0.5–2.0). While each bulk material input could be parameterized independently, this would unnecessarily encumber the model with supposedly dependent variables.

Operation is relatively influential in the full LCA, with highly varying parameters: infrastructure lifetime is reported to range from 25 to 60 years,^28^ with extensions to 80 years approved in the US;^74^ availability, i.e., the time during which the plant is ready to operate, excluding planned maintenance and unplanned interventions, which range from about 65% (e.g., with the French fleet, due to load following and in 2021 long overhauls and unplanned inspections) to above 90%;^75^ capacity, often fixed at 1000 MW in LCA studies, but ranging from 730 to 62400 MW (the scope of ref (34) being the whole French nuclear fleet).

Operation consists of using the fuel assemblies in reactors to generate heat via fission, which in turn generates electricity. Structuring factors for this phase are discharge burnup rate and turbine efficiency. Discharge burnup rate measures how much energy is harnessed from a given quantity of nuclear fuel at a specific enrichment rate, a proposal for the relationship between the former and the latter is made in this model (see SI). “Burnup” is a misnomer as the fuel does not undergo any combustion, but it is the term conventionally used. Burnup rate is quantified in GW-day/t U; most LWRs range between 35 and 50 GWd/t U, with some exceptions above 60.

Finally, cooling is a major source of water consumption, specifically in a closed loop with cooling towers, which evaporate a significant share of the water, typically collected in a nearby river. This uptake is estimated to be 2.3 l/kWh in closed-loop cooling (river) and 0.0 l/kWh for coastal power plants,^34^ which can release the entirety of the cooling water back into its source without causing temperature variations with potential impacts on biodiversity.

Decommissioning is assumed to last for seven years, following the assumptions made in Zhang and Bauer,^35^ including the following energy inputs: 54 TJ of diesel for machinery, 53 GWh of electricity, and 14 TJ of heat (light fuel oil). All details for on-site activities (construction, operation, and decommissioning) are available in the inventory file as Supporting Information, SI.

### 3.6 Spent Fuel Management

After being depleted, fuel rods are removed from the reactor and stored in interim storage to cool down. Used fuel storage conventionally takes place at the nuclear plant site, in dry casks or dedicated pools of water. It is to be noted that some countries, such as France, reprocess the fuel into mixed oxide (MOX) fuel, but we exclude reprocessing from this LCA, which therefore represents a 100% open cycle.

### 3.7 Final Waste Disposal

Once sufficiently cooled, the encapsulated fuel rods can be stored in a final repository. The corresponding LCI was adapted from the Swedish repository project at Forsmark, Östhammar municipality.

### 3.8 Summary

Table 2 shows a summary of the parameters used in the full model, with their default value, minimum, maximum, standard deviation, distribution type, and unit. The full parameter setup, with formulas, inventory integration, and testing, is available in the Python Jupyter Notebooks, allowing us to reproduce the results.

**Table 2:** Variable Parameters Used for the LCA Model

name	default	min	max	std	distribution	unit
mining						
mining technique (ISL share)	0.574	0	1		linear	dimensionless
mining electricity switch	diesel				diesel/grid	
uranium ore grade	0.001544	1e-05	0.02	0.731586	log-normal	dimensionless
tailings ^222^Rn	0.01951	0.01	1		triangular	Bq/s
integration time	80 000	100	80 000		linear	year
milling						
milling electricity switch	diesel				diesel/grid	
conversion						
conversion electricity input	11.8	10.3	16.9		triangular	kWh/kg U in UF_6_
conversion heat input	26	26	665		triangular	kWh/kg U in UF_6_
enrichment						
rate of enrichment	0.042	0.03	0.05		triangular	dimensionless
rate of feed	0.0071				fixed	dimensionless
rate of tailings	0.0022				fixed	dimensionless
electricity consumption of centrifugation	50	40	100		triangular	kWh/SWU
electricity consumption of diffusion	2500	2400	3000		triangular	kWh/SWU
enrichment technology	centrifugation				centrifugation (80%) diffusion (20%)	
fuel fabrication						
fuel fabrication electricity	36	36	50		triangular	kWh/kg fuel
power plant construction and decommissioning						
lifetime	60	30	80		triangular	year
nameplate capacity	1000				fixed	MWe
electricity production						
efficiency of electricity generation	0.33	0.3	0.34		fixed	dimensionless
availability of power plant	0.9	0.65	1		triangular	dimensionless
cooling type	river				river/coastal	

## 4 Results

### 4.1 Monte Carlo Simulation

**Figure 2:**
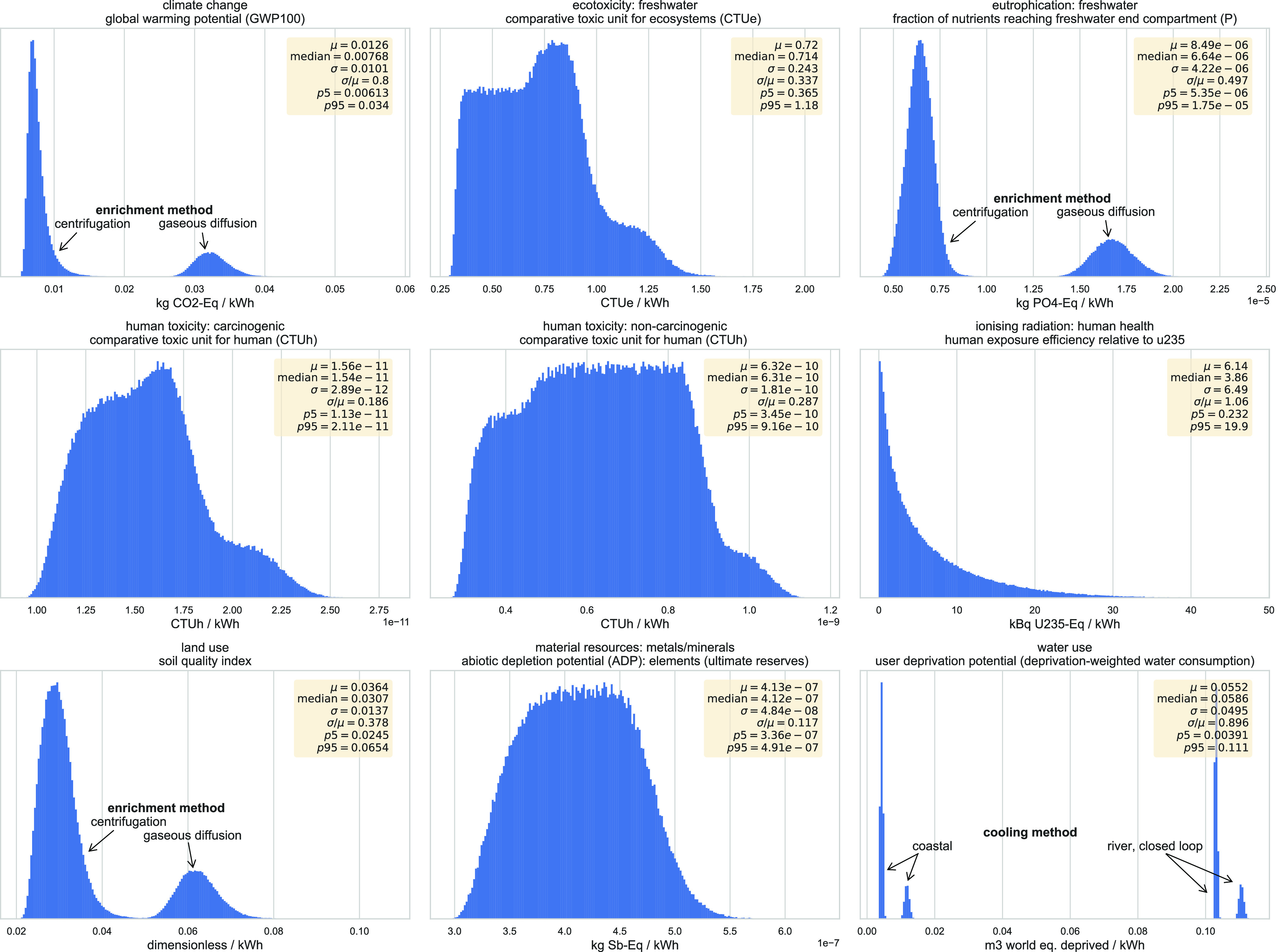
Monte Carlo results for the different impact LCA impact categories in the form of distributions. Distributions with distinct, separate peaks are annotated with corresponding parameter choices. Statistical indicators shown include mean (μ), median, standard deviation (σ), coefficient of variation (σ/μ), and percentiles (5% and 95%).

Figure 2 presents the Monte Carlo simulation results for the nine impact categories assessed after varying the 19 variables according to the ranges specified in Table 3. Regarding the dispersion of these results, the coefficient of variation (standard deviation over mean) ranges from 11.7% for material resources to 106% for ionizing radiation. For impacts influenced by the Boolean modeling choices, results are distributed around hotspots, namely, enrichment technology, with centrifugation as the “lower pole.” Regarding greenhouse gas emissions, the mean of the distribution is 12.1 g CO_2_ equiv/kWh with a standard deviation of 10.1 g CO_2_ equiv/kWh and a variability of 83.5% (5%–95% range of 6.0–33.5 g). Notably, the process of gaseous diffusion, discontinued globally as of 2020, has been kept here for the purpose of analysis and comparability with older publications.

**Table 3:** Simplified Models per Impact Category, From the Full LCA Model^a^

impact category	unit (per kWh)	simplified model
climate change	kg CO_2_ equiv	
freshwater ecotoxicity	CTU_e_	
freshwater eutrophication	kg P	
human toxicity (carcinogenic)	CTU_h_	
human toxicity (non-carcinogenic)	CTU_h_	
ionizing radiation	kg ^235^U equiv	(see the SI for simplified model)
land use	dimensionless	
material resources	kg Sb equiv	
water use	l	

a At least 90% of the global variation can be explained by the parameters retained.

The Monte Carlo simulations use precalculated background values and explicit parameters; equations thus obtained can serve as simplified models for the nine impact categories analyzed here. These fully parametric equations are provided in a spreadsheet as SI.

### 4.2 Foreground Contribution Analysis

**Figure 3:**
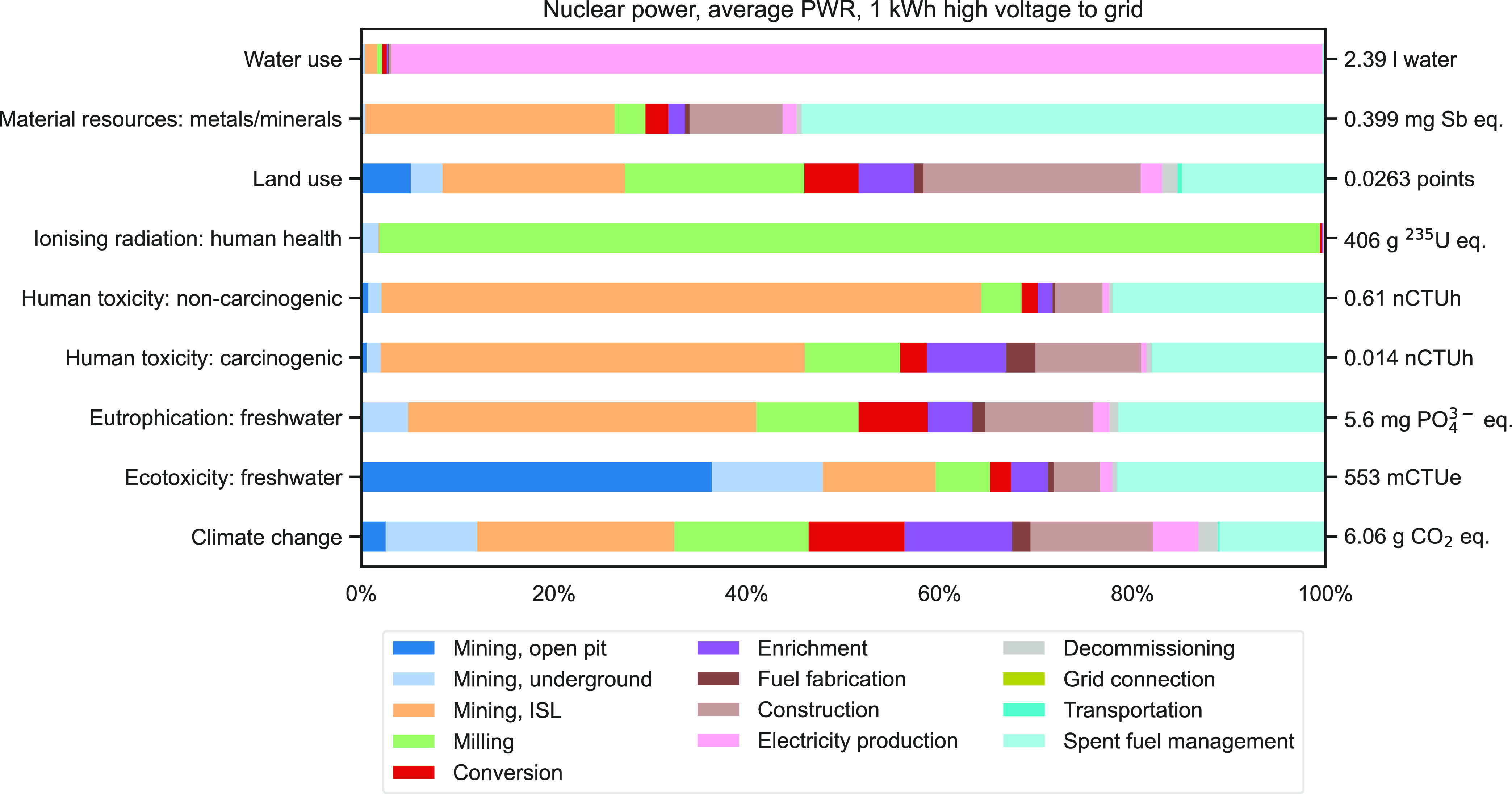
Contribution analysis of foreground processes for the production of 1 kWh of high-voltage electricity for nine indicators (with enrichment via centrifugation only).

The life cycle environmental impacts of 1 kWh of nuclear power are shown in Figure 3 as a contribution analysis, with enrichment through centrifugation only. In terms of greenhouse gas emissions, the total of 6.1 g CO_2_ equiv is dominated by the fuel supply chain. Mining and milling represent 46% of GHG emissions, with the current mining split, conversion, enrichment, and fuel fabrication contributing another 23%, construction 13%, operation 5%, and backend processes 13%. A similar pattern can be found for land use and, to a certain extent, eutrophication and human toxicity, although ISL contributes more to these two indicators. Contrasting with climate change, freshwater ecotoxicity is dominated by open pit mining because of blasting operations. With 80 000 years as the integration time, ionizing radiation is almost exclusively due to the milling process, in particular to the emissions of radon in milling tailings; but in the short term (100 years), underground mining becomes dominant. On the material resource side, ISL and spent fuel management contribute significantly—both because of copper requirements. The contribution of ISL is explained by the use of sulfuric acid, which requires the extraction of sulfide ore, a coproduct of copper extraction. In the “cutoff” paradigm of the LCI database, these substances cannot be provided to the market independently and therefore share the burdens associated with their extraction. The contribution of spent fuel management, on the other hand, originates from the direct use of copper, as spent fuel is assumed to be encapsulated in massive copper casks. Virtually all water use, representing water removed from its environment, is a consequence of cooling during the operation phase, where water is transferred from a cooling source (typically a river) to the atmosphere.

### 4.3 Global Sensitivity Analysis

To identify the parameters with the highest contribution to the variance of each impact category, a GSA was conducted on the 19 variable parameters. The number of iterations was doubled until convergence was found at 2.^16^ Enrichment technology explains more than 90% of the variance for water use, climate change, eutrophication, and land use. It also contributes significantly to the variance of ecotoxicity and human toxicity (carcinogenic) impacts, with 38 and 45%, respectively. The share of in situ leaching in uranium extraction is significant for material resources, human toxicity, ecotoxicity, and to a lower extent, ionizing radiation. Finally, the amount of radon-222 emitted from milling tailings (which depends on remediation efforts) is an influent parameter regarding ionizing radiation. All other parameters do not explain more than 8% of the overall variance. The sum of Sobol indices for each impact category is always lower than 1, indicating that some of the variance is explained by interactions between parameters. Lacirignola et al.^51^ arbitrarily set 0.6 as the Sobol indices’ aggregated contribution minimum threshold; in the present model, the lowest Sobol indices sum, 0.76, occurs for ionizing radiation.

**Figure 4:**
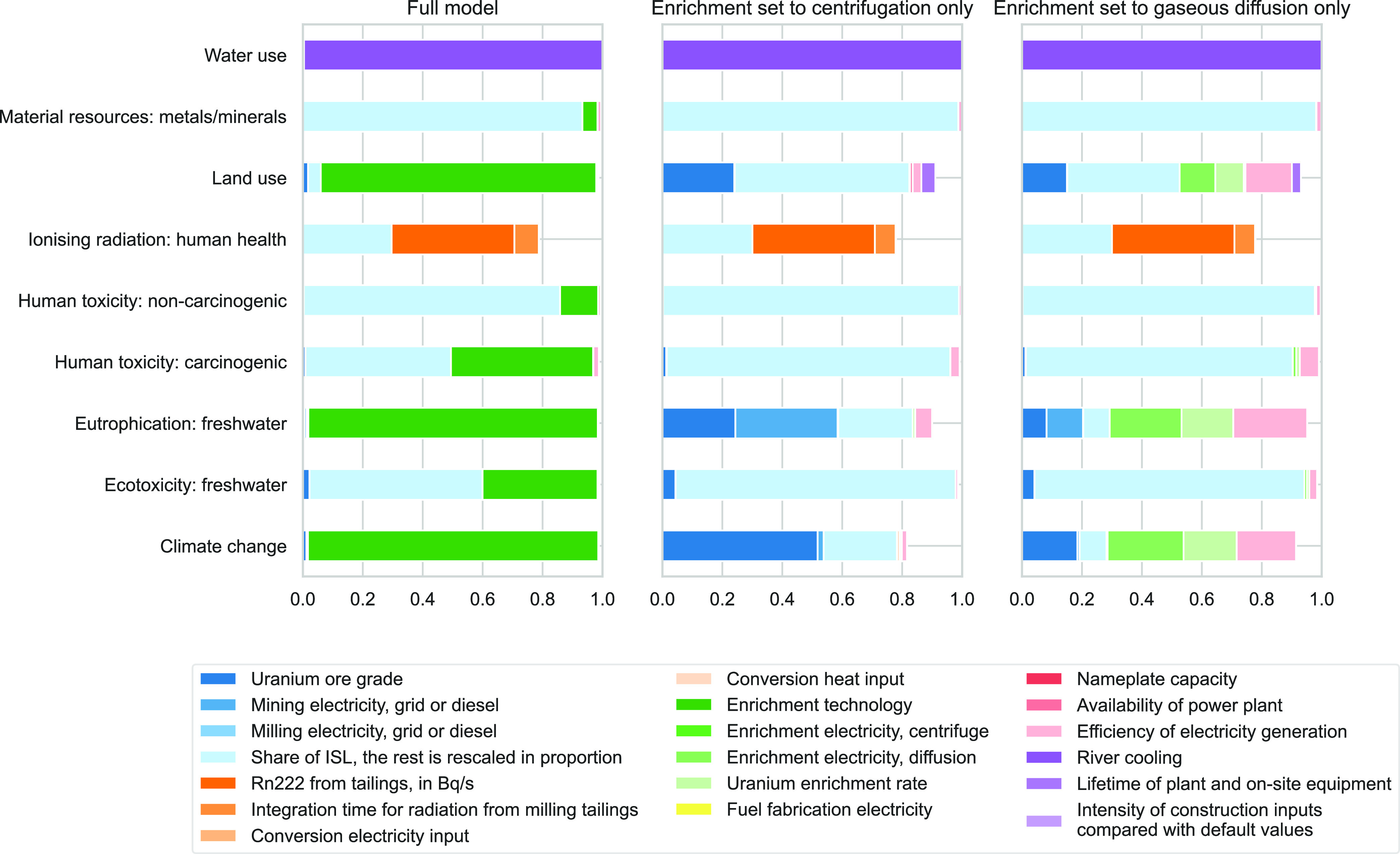
First-order Sobol indices quantifying the contribution of each input parameter to the total variance of each impact category for the full model, enrichment set to centrifugation and to gaseous diffusion.

To get a better insight into the other parameters, the GSA was run again fixing this time the enrichment technology, as shown on the second and third panels of Figure 4, which correspond to centrifugation and gaseous diffusion, respectively. When enrichment is set to centrifugation only, the share of ISL in the uranium extraction mix becomes the most influential parameter, followed by uranium ore grade, mining energy source, lifetime, and construction intensity of nuclear power plant, as well as efficiency of electricity generation. In the case of gaseous diffusion, the same influent parameters are found, in addition to the enrichment’s per-SWU electricity input, as well as the enrichment rate, which determines the overall electricity consumption of the enrichment phase. In both cases, ionizing radiation is not affected.

### 4.4 Simplified Models

The GSA provides a list of the most influential parameters for each impact model, which allows the generation of simplified models, i.e., formal equations with the minimum amount of parameters that can explain the maximum variation. These models can be used to get first-order estimates of the life cycle impacts of nuclear power without having to run a full LCA model. They are valid as long as parameter values remain within their respective range of definition. Expectedly, from Figure 4, and as seen in Table 3, if the enrichment technique is not fixed, then it is a major parameter for almost all impact category indicators.

### 4.5 Scenarios

**Figure 5:**
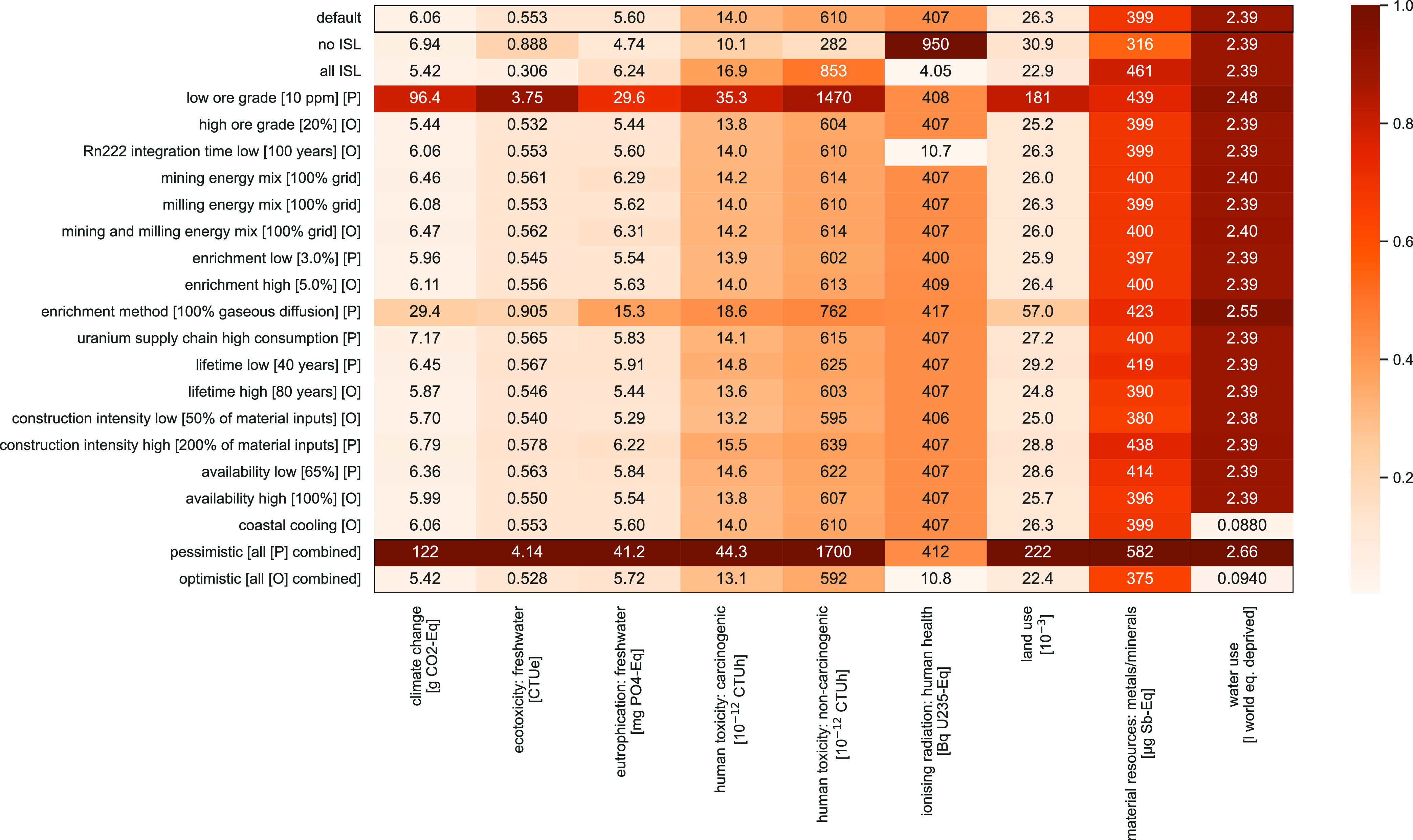
Heatmap showing the environmental impacts for the nine impact categories under the different modeling scenarios, either optimistic [O] or pessimistic [P]. Values for the combined optimistic and pessimistic scenarios are shown in the last two lines. All values are shown with three significant digits.

A stress test of the model can be performed by assigning extreme values to the various parameters. This section presents the results of setting each parameter to either its minimum or maximum value, labeled optimistic, “O”, or pessimistic, “P”, depending on the sign of the variation correlation with the overall score correlation, as shown in Figure 5. A combination of all optimistic and pessimistic settings is also provided to represent extreme cases. This analysis is performed for the sake of comparison with previous literature, which also explains why gaseous diffusion was retained as an enrichment technology. Note that the share of ISL and mining and milling energy mixes are not labeled as optimistic or pessimistic as they correlate both positively and negatively with the various impact scores.

When distributions are disregarded, parameters leading to the highest variation for all indicators (except ionizing radiation, material and water requirements) are uranium ore grade and enrichment method (gaseous diffusion). The share of ISL in uranium extraction mix has a significant influence on all indicators (except water use), including ionizing radiation, as most radioactive emissions occur from milling tailings, which ISL does not create. To highlight a few results, life cycle impact scores for the generation of 1 kWh range from 5.42 to 122 g CO_2_ equiv for climate change, 10.8–950 Bq ^235^U equiv/kWh for ionizing radiation, 22.4–222 millipoints for land use, and from 282 to 1700 CTUh for combined human toxicity (carcinogenic and non-carcinogenic).

## 5 Discussion

Reported life cycle impact scores for nuclear power vary widely, especially regarding GHG emissions, with an overall range of 1.8–220 g CO_2_ equiv/kWh. The present parametric model shows a possible variation of 5.4–122 g CO_2_ equiv/kWh, therefore not covering the full range found from the literature survey but relatively close to the IPCC range of 3.7–110 g CO_2_ equiv/kWh. The global sensitivity analysis identifies enrichment method, the share of in situ leaching in the uranium extraction mix, as well as uranium ore grade as the three main parameters influencing the overall LCIA scores. Considering these results and the characteristics of the global uranium chain as of 2020 (more extraction via ISL, no more gaseous diffusion, relatively cleaner background electricity mix), it is highly unlikely that nuclear power display more than 20 g CO_2_ equiv/kWh, except for cases where uranium would be sourced from sub-100 ppm ore, which represents a small share of the global tonnage (see Figure 2 in the SI). When ionizing radiation is considered, radon-222 emissions and radiation integration time are highly significant (see note in the SI).

Nuclear power plant infrastructure-related parameters, such as construction intensity or lifetime, are found to be of minor effect across all indicators. Minimum lifetime has been set to 40 years, a commonly accepted value for first- and second-generation reactors, which means that premature phaseouts are not covered in the model. On the other hand, longer lifetimes may lead to higher maintenance inputs, which could have been parameterized as a function of lifetime—this has not been considered. Given the relatively low importance of infrastructure in the overall footprint, no significant changes are to be expected. Operation parameters, i.e., availability and efficiency, are uninfluential, except when gaseous diffusion is used for enrichment. These results confirm the importance of the uranium fuel chain over the life cycle for all indicators, especially the mining phase concerning GHG emissions. As shown in Figure S2, new extraction techniques such as ISL are dominating the market, which is beneficial regarding GHG emissions and ionizing radiation, but may lead to potentially concerning trade-offs such as higher emissions of human toxicity substances. Finally, the type of cooling is found to be the major parameter influencing the water use indicator.

The parametric equations calculated in Section 4.1 can be used for global sensitivity analysis. While simplified equations can be seen as more user-friendly, there is technically no obstacle to use the full (i.e., with the 20 parameters) equations systematically—as shown, they can be easily transcribed to a spreadsheet without any loss of performance (see the SI). An argument in favor of fully explicit equations over simplified models is that end users may have primary data for parameters with little influence on the default configuration, which end up having a higher influence on another user-specific configuration. The example in this study is that uranium ore grade is a relatively insignificant parameter for climate change (it is filtered out in the default model, as its Sobol index is below 1% of the overall variance) as long as the enrichment technique is not chosen. Once enrichment is set to gaseous diffusion or centrifugation, it becomes a major parameter.

Nuclear power generation involves many industrial activities, from extraction to various refining processes, enrichment and fuel fabrication, power plant construction, operation and decommissioning, as well as interim and permanent spent fuel management. A parametric model was developed to account for the potential options and variability at many steps of the life cycle. Results from the analyses run with this model have highlighted influential parameters, quantifying their importance for each of the nine environmental impact categories retained. These results confirm the findings of past studies regarding the significance of ore grade, milling tailing management, extraction mix, enrichment technique, power plant operation, as well as other minor aspects of the nuclear fuel chain. While simplified LCA models are undoubtedly helpful in understanding relationships between input parameters and impact assessment results, there is technically no obstacle in keeping all parameters as variables in LCIA models for various impact indicators.

Limitations include four main challenges. First, it is computationally impossible to parameterize all variables in the model; the list of retained parameters has been determined based on a literature survey. Second, most parameters have been modeled as triangular distributions, as is common practice when not enough data points are available. Collecting more data and choosing more accurate distributions for the most influential parameters could improve the model significantly. Third, interactions between parameters (e.g., energy requirements vs ore grade, ore recovery rate, burnup rate vs enrichment rate) rely on models which themselves carry over significant uncertainties, which are not covered in the present life cycle model. Fourth, the nuclear power industry is constantly evolving, e.g., in terms of post-Fukushima safety requirements. This means that inventories cannot be 100% up to date.

Future research should focus on detailing further the relationships between parameters, which can be done by collecting more data, especially at the uranium extraction phase. More parameters could clearly be added, such as water use (which could depend on the location of a power plant and the cooling source, either river or sea), electricity mix (which is here fixed to the specific global average for extraction, enrichment, and fuel fabrication), or land use of each life cycle phase, which has become the object of recent meta-analyses highlighting variations.^76,77^ Other considerations should also be accounted for, as electricity generation technology and uranium supply chain evolve. As new reactor designs, such as small modular reactors,^78,79^ become commercially available, the range of installed capacity will widen (e.g., 100–1600 MW) and become an interesting parameter to analyze. Similarly, the use of reprocessed spent fuel for new rod fabrication is already a mature solution;^80^ a recycling rate parameter could be integrated in the present model. Last, immaterial inputs (financing, insurance···) have not been accounted for; inventories could be completed with input–output data.

## Supporting Information Available

Functional unit; system boundaries; data collection; inputs for surface, open pit mining, per kg of uranium in ore; inputs for milling, and per kg of uranium in yellow cake; additional results (PDF)
Literature review file (“Systematic_review”) (XLSX)
Full parametric model (“parametric_LCA_all_impacts_1.0”) (XLSX)
Jupyter notebooks that can be used to replicate the results (“nuclear-parametric-lca”) (ZIP)

(PDF)
(XLSX)
(XLSX)
(ZIP)
