# Supplementary material for: Parametric Life Cycle Assessment of Nuclear Power for Simplified Models
Source: Environ Sci Technol. 2023 Sep 12;57(38):14194–205. doi: 10.1021/acs.est.3c03190 (PMC10537461; doi:10.1021/acs.est.3c03190)
Supplement: Supplementary file 1 — es3c03190_si_001.pdf [file es3c03190_si_001.pdf]

Supporting Information to “Parametric life cycle assessment of nuclear power for simplified models

Thomas Gibon[1], Álvaro José Hahn Menacho[1,2]*

[1]Luxembourg Institute of Science and Technology, 5 avenue des Hauts-Fourneaux, 4362 Esch-sur-Alzette, Luxembourg

[2]Paul Scherrer Institute, 5232 Villigen, Switzerland

*thomas.gibon@list.lu

Supporting information files

Supporting information

This file.

Literature review

The details of the literature review are available in “**Systematic_review.xlsx**”, with references, year of publications, lifecycle GHG values, and other details depending on the source.

Full parametric model

The full parametric model is available as “**parametric_LCA_all_impacts_1.0.xlsx**”, in which lifecycle impact assessment is performed for the 9 selected indicators. The 21 parameters can be set manually, and LCIA results are automatically calculated, outputting the exact same values as the full LCA model immediately.

Code availability

To ensure reproducibility, the whole code is available as jupyter notebooks in “**nuclear-parametric-lca.zip**”. This compressed folder also contains the notebooks as html files, which can be read (but not run) without any python distribution.

Contents

Figures....3
Tables....3
Functional unit....4
System boundaries....5
Data collection....6
Uranium extraction....6
Conversion....10
Enrichment....11
Fuel fabrication....12
Construction....12
Operation....13
Spent fuel management....14
Final waste disposal....14
Life cycle impact assessment....16
A note on ionizing radiation....16
Additional results....17
Variability of lifecycle impact results with respect to ore grade....17
Variability of lifecycle impact results with respect to extraction technique (ISL)....17
Extra results for simplified models....18
Extra files.... **Error! Bookmark not defined.**
Literature review....1
Full parametric model....1
Code availability....1
References....19

Figures

Figure S1. Basic diagram of the nuclear fuel chain to produce “1 kWh, high voltage, to grid” 5
Figure S2. Uranium ore grade range with respect to tonnage, per mine 6
Figure S3. Global uranium mix, by extraction technique 7
Figure S4. Total energy requirements (diesel, heat, electricity) per unit of uranium ore (U_3O_8) 8
Figure S5. Bulk material requirements for a nuclear power plant 12
Figure S6. Relationship between discharge burnup rate and enrichment rate 13
Figure S7. Parameters of the model and their distributions 15
Figure S8. Variability of environmental impact with respect to ore grade 17
Figure S9. Variability of environmental impact with respect to extraction technique 17

Tables

Table S1. Inputs for surface, open pit mining, per kg of uranium in ore 8
Table S2. Inputs for underground mining, per kg of uranium in ore. 8
Table S3. Inputs for surface mining, in-situ leaching, per kg of U in yellowcake 8
Table S4. Inputs for milling, per kg of uranium in yellowcake. 9
Table S5. Inputs for uranium (underground & open pit) mining and milling 9
Table S6. Inputs for uranium (ISL) mining and milling 9
Table S7. Inputs for conversion, per kg UF_6 (non-enriched). 10
Table S8. Inputs for fuel fabrication, per kg fuel element. 12
Table S9. Inputs for NPP construction, 1000 MW reactor. 12
Table S10. Chemical inputs for NPP operation, 1000 MW reactor. 13
Table S11. Inputs for interim storage of spent fuel, per TWh of average NPP operation. 14
Table S12. Inputs for one spent fuel canister. 14
Table S13. Inputs for encapsulation of spent fuel from interim storage, per TWh of NPP operation. ... 14
Table S14. Inputs for deep waste repository, per TWh of NPP operation. 14
Table S15. Life cycle impact assessment categories. 16
Table S16. Simplified models, explaining 80% of the overall variance, with enrichment technique set to 100% centrifugation. 18
Table S17. Simplified models, explaining 80% of the overall variance, with enrichment technique set to 100% centrifugation and ore grade set to its default value of 1544 ppm. 18

Functional unit

Process-based LCA is an ISO-standardized method for assessing the environmental impacts of a product or a service. More specifically, the ISO14040/44 standards define the processes to follow in order to account for all flows of energy and materials, emissions and waste, linked directly or indirectly to a so-called "functional unit". This functional unit is strictly defined to represent (non-exhaustively) either a service provided, the use of a product, the activity of a territory over a given period, or the deployment of a technology.

In the present case, the functional unit can be defined as "generating 1 kWh of high-voltage electricity from a pressurized water reactor". The nuclear reactor and uranium fuel chain are modelled to be representative of the 2020 global situation (or the most recent year available). The system associated with this functional unit is represented in Figure S1.

System boundaries

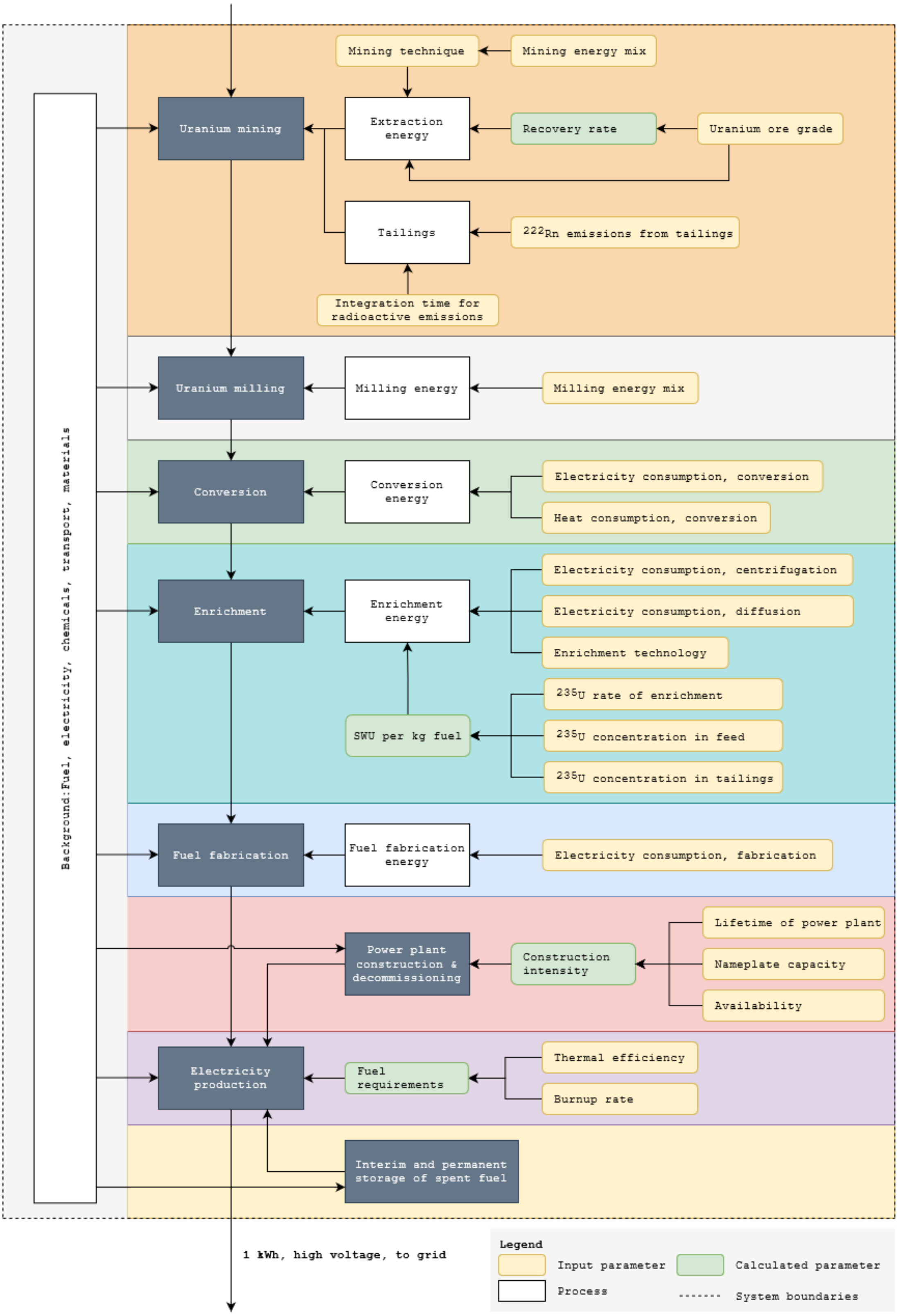

Figure S1. *Basic diagram of the nuclear fuel chain to produce "1 kWh, high voltage, to grid" as functional unit. Square boxes represent each of the processes modelled. Rounded yellow boxes represent the variables defined to parameterize the inventories. Finally, each background color within the system boundaries represents a life cycle step. Milling is considered an additional step to mining, but in the case of ISL extraction the product is yellowcake (no milling is involved).*

Data collection

Nuclear power has been subject to a consultation process with the World Nuclear Association in order to build new life cycle inventories for the front-end, core, and back-end processes of the nuclear life cycle. Significant changes have been brought to the nuclear power inventory in Gibon *et al.* [1] regarding the mining & milling (using Haque *et al.* [2] as main source for this step), and spent fuel management, which reflects recent changes in the nuclear power industry.

Uranium extraction

Data for the extraction of uranium was collected from the IAEA's UDEPO database, which contains grade-tonnage data for all operational uranium extraction sites in the world. Although the IEAE does not communicate on the exact mine-level data, a graphical representation of the database is available on Figure S2. The retained distribution is lognormal, with mean 0.1544% and standard deviation 0.1299%, as reported in Monnet *et al.* [3].

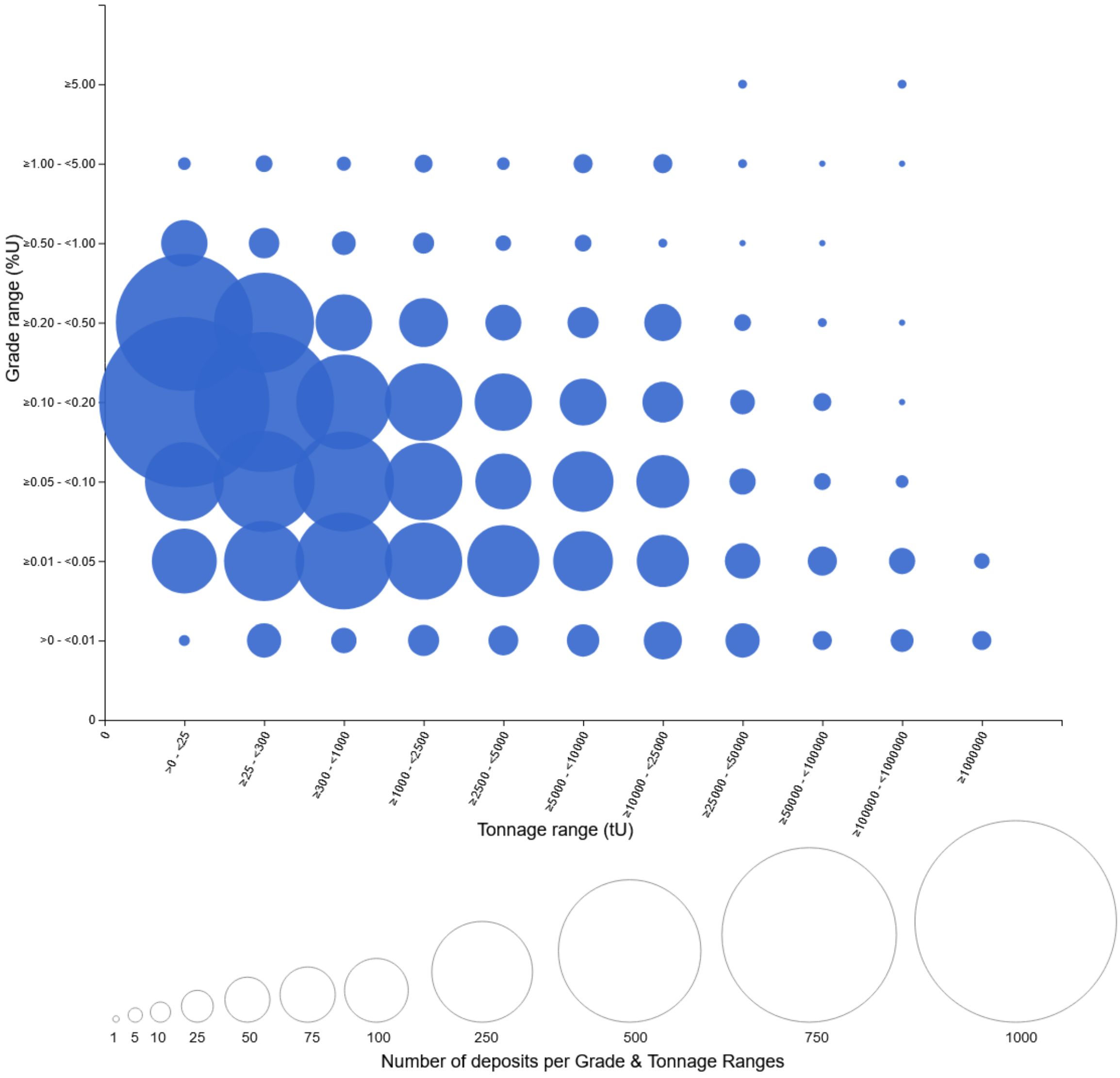

Figure S2. *Uranium ore grade range with respect to tonnage, per mine.*
Available at https://infcis.iaea.org/UDEPO/Chart

The global extraction technique mix has significantly evolved between 1998 and 2021 (Figure S3), ISL dominates the uranium production market with two-thirds of the global production for that last year. This mode shift has consequences on the life cycle environmental profile of nuclear fuel as ISL has for example a lower GHG footprint than other techniques [2]. In the present model, the share of ISL in the mining mix is retained as a parameter, with the open pit and underground mining shares rescaling accordingly. In the absence of a proper technology description, other extraction techniques are aggregated into the "open pit" category.

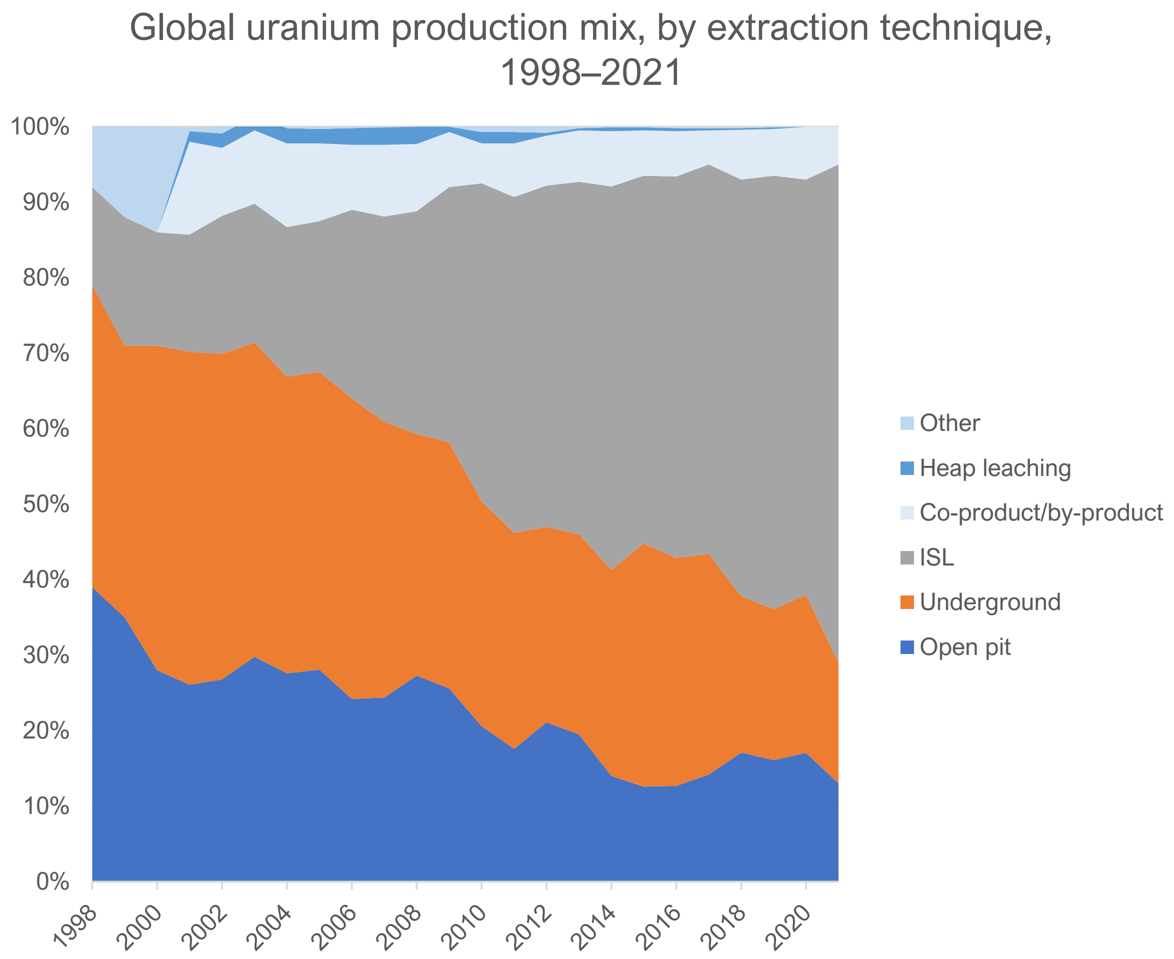

Figure S3. *Global uranium mix, by extraction technique. Before 2001 and after 2020, "Co-product/by-product" and "Heap leaching" were merged into "Other". Sources: [4] and prior IAEA "Red Book" reports.*

Figure S4 shows how energy requirements vary with ore grade and extraction technique for 23 data points [2, 5-9]. The correlation is weak, but the general trend is that energy use tends to decrease with increasing ore grade. For ISL, this decrease is very slow, which can be interpreted by the fact that this technique does not require to move an amount of waste rock proportional to ore grade, unlike the two others. Outliers include [8], showing that only 53 MJ of energy is necessary to the open cast extraction of 1 kg U_3O_8 at 0.15%, or 440 MJ/kg U_3O_8 via ISL, more than double the next-highest value. In general, more data points are required to reduce the uncertainty of this parameter before a solid interpretation can be made.

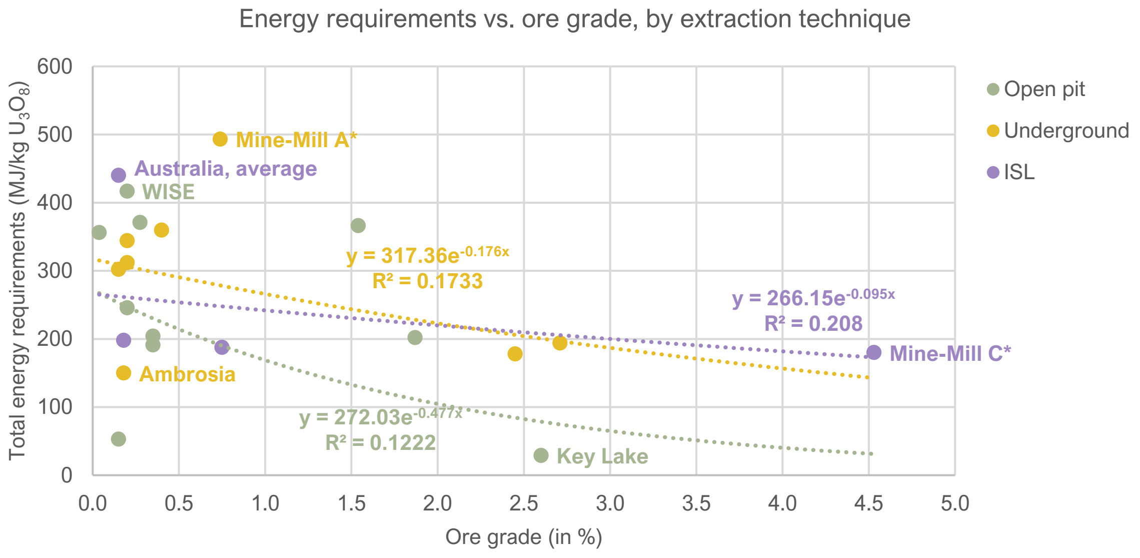

Figure S4. *Total energy requirements (diesel, heat, electricity) per unit of uranium ore (U_3O_8), per extraction technique and ore grade. The mines with minimum and maximum energy requirements for each category is indicated. Some mines ("Mine-Mill" A and C) are anonymized in the original source [7]. *Milling energy requirements included.*

Regarding energy inputs of mining, the retained values are shown in **Table S1**, **Table S2**, **Table S3**, and **Table S4**. For comparison, values form the various sources available is shown in **Table S5** and **Table S6**.

Table S1. *Inputs for surface, open pit mining, per kg of uranium in ore.*

Inputs	Amount	Unit	Comment
blasting	1.52	kg	WNA consultation
diesel, burned in building machine	12.2	MJ	WNA consultation
diesel, burned in diesel-electric generating set, 10MW	293.9	MJ	WNA consultation
mine infrastructure construction, open cast, uranium	6.17E-08	unit	ecoinvent assumption

Table S2. *Inputs for underground mining, per kg of uranium in ore.*

Inputs	Amount	Unit	Comment
blasting	0.29	kg	WNA consultation
diesel, burned in diesel-electric generating set, 10MW	133.4	MJ	WNA consultation
heat, district or industrial, other than natural gas	247.5	MJ	WNA consultation
electricity, medium voltage	68.1	MJ	WNA consultation
mine infrastructure, underground, uranium	2.78E-07	unit	ecoinvent assumption

Table S3. *Inputs for surface mining, in-situ leaching, per kg of U in yellowcake.*

Inputs	Amount	Unit	Comment
ammonium nitrate	2.5	MJ	WNA consultation
electricity, medium voltage	43.4	kg	WNA consultation
diesel, burned in diesel-electric generating set, 10MW	32.95	kg	WNA consultation
petrol, unleaded, burned in machinery	4.1	kg	WNA consultation
heat, central or small-scale, other than natural gas	103.9	kg	WNA consultation
steel, chromium steel 18/8	0.108	kg	ecoinvent assumption
sulfuric acid	65.5	kg	WNA consultation
water, decarbonised	173.2	kg	WNA consultation
hydrogen peroxide, without water, in 50% solution state	0.61	kg	Haque et al. (2014)
phosphoric acid, industrial grade, without water, in 85% solution state	0.23	kg	Haque et al. (2014), D2EHPA
hydrochloric acid, without water, in 30% solution state	0.03	kg	Haque et al. (2014)
sodium bicarbonate	0.3	Kg	Haque et al. (2014)
sodium hydroxide, without water, in 50% solution state	1.37	kg	Haque et al. (2014)
sodium chlorate, powder	8.21	kg	Haque et al. (2014)

Table S4. *Inputs for milling, per kg of uranium in yellowcake.*

Inputs	Amount	Unit	Comment
Electricity, medium voltage	22.5	kWh	WNA consultation
Tailing, from uranium milling	-0.25	m^3	ecoinvent assumption
Sulfuric acid	55	kg	WNA consultation
Diesel, burned in diesel-electric generating set, 10MW	57	kg	WNA consultation
Uranium mine operation, open cast, WNA	30%	kg	WNA consultation
Uranium mine operation, underground, WNA	70%	kg	WNA consultation

Table S5. *Inputs for uranium (underground & open pit) mining and milling*

Chemicals	Parker et al. 2016 - Weighted average for underground / open pit / raisebore mining + Milling		Uranium ore underground mining and milling: Ecoinvent3.7 - Uranium ore, as U [10]| uranium mine operation, underground		Uranium, in yellowcake [10]| production	
Ammonia	0.404	kg/kg U3O8			0.9	kg/kg
Lime/Quicklime	2.91	kg/kg U3O8				
Hydrogen peroxide	0.202	kg/kg U3O8				
Diluent (kerosene)	n.a.	kg/kg U3O8				
D2EHPA (Di-(2-ethylhexyl)phosphoric acid)	n.a.	kg/kg U3O8				
Amine	n.a.	kg/kg U3O8				
TBP (tributyl phosphate)	n.a.	kg/kg U3O8				
Hydrochloric acid	n.a.	kg/kg U3O8				
Sodium carbonate	n.a.	kg/kg U3O8				
Sodium hydroxide	n.a.	kg/kg U3O8			0.026	kg/kg
Sulphuric acid	n.a.	kg/kg U3O8			35	kg/kg
Sodium chlorate	n.a.	kg/kg U3O8			1	kg/kg
Ammonium sulfate					0.106	kg/kg
Chemical inorganic					0.26	kg/kg
Chemical organic					0.315	kg/kg
Ethylenediamine					0.012	kg/kg
Soda ash					2.5	kg/kg
Sodium chloride					2.5	kg/kg
Other non chemical - for operation						
Bentonite						
Barite						
Blasting	0.0912	kg/kg U3O8	0.26	kg/kg ore		
Diesel	36.86	MJ/kg U3O8	300	MJ/kg ore	176	MJ/kg
Water			0.1	m3/kg ore	1	m3/kg
Electricity	22	kWh/kg U3O8				
Heat (other than gas)					250.8	MJ/kg

Table S6. *Inputs for uranium (ISL) mining and milling*

Chemicals	Haque et al. 2014 - In situ leaching - Australia		Ecoinvent3.7 - Uranium, in yellowcake (GLO)| uranium production, in yellowcake, in-situ leaching	
Ammonia	-	kg/kg U3O8 as yellow cake		
Lime/Quicklime	-	kg/kg U3O8 as yellow cake		
Hydrogen peroxide	0.61	kg/kg U3O8 as yellow cake		
Diluent (kerosene)	0.88	kg/kg U3O8 as yellow cake		
D2EHPA (Di-(2-ethylhexyl)phosphoric acid)	0.23	kg/kg U3O8 as yellow cake		
Amine	0.23	kg/kg U3O8 as yellow cake		
TBP (tributyl phosphate)	0.23	kg/kg U3O8 as yellow cake		
Hydrochloric acid	0.03	kg/kg U3O8 as yellow cake		
Sodium carbonate	0.3	kg/kg U3O8 as yellow cake		
Sodium hydroxide	1.37	kg/kg U3O8 as yellow cake		
Sulphuric acid	7.87	kg/kg U3O8 as yellow cake	20.0	kg/kg
Sodium chlorate	8.21	kg/kg U3O8 as yellow cake		
Other non chemical - for operation				
Bentonite	0.08	kg/kg U3O8 as yellow cake		
Barite	0.21	kg/kg U3O8 as yellow cake		
Blasting				
Diesel	11.66	MJ/kg U3O8 as yellow cake	886.6	MJ/kg
Water			9.1229347	m3/kg
Electricity (pumping)	28	kWh/kg U3O8 as yellow cake		
Heat (other than gas)				

Conversion

In the conversion step, yellowcake undergoes several processes: dissolution in nitric acid, solvent extraction, washing, and concentration by evaporation. The resulting solution is then calcined to produce uranium trioxide or dioxide. This uranium oxide is then combined with gaseous hydrogen fluoride in a kiln to produce uranium tetrafluoride (UF_4), which finally reacts with gaseous fluorine (F_2) to produce uranium hexafluoride (UF_6). Because of the kiln, conversion is a heat-intensive process, and requires about 600 MJ of energy per kg UF6 [11], this heat input is kept as a parameter.

Table S7. *Inputs for conversion, per kg UF_6 (non-enriched).*

Inputs	Amount	Unit	Comment
acetylene	0.000025	kg	ecoinvent assumption
aluminium, wrought alloy	0.05	kg	ecoinvent assumption
argon, liquid	0.0018	kg	ecoinvent assumption
brass	0.0018	kg	ecoinvent assumption
chemical, organic	0.00082	kg	ecoinvent assumption
chemicals, inorganic	0.0311	kg	ecoinvent assumption
concrete, normal	0.00029	m3	ecoinvent assumption
diesel, burned in diesel-electric generating set, 10MW	1.28	MJ	ecoinvent assumption
**Electricity, high voltage, uranium enrichment mix**	**40.0**	**kWh**	**WNA consultation**
heat, district or industrial, natural gas	13.68	MJ	ecoinvent assumption
hydrochloric acid, without water, in 30% solution state	0.0002	kg	ecoinvent assumption
hydrogen peroxide, without water, in 50% solution state	0.00068	kg	ecoinvent assumption
hydrogen, liquid	0.000011	kg	ecoinvent assumption
low level radioactive waste	-0.00063	m3	ecoinvent assumption
lubricating oil	0.0092	kg	ecoinvent assumption
methanol	0.00032	kg	ecoinvent assumption
nitric acid, without water, in 50% solution state	0.0015	kg	ecoinvent assumption
nitrogen, liquid	0.00039	kg	ecoinvent assumption
oxygen, liquid	0.000036	kg	ecoinvent assumption
phosphoric acid, fertiliser grade, without water, in 70% solution state	0.00012	kg	ecoinvent assumption
polyvinylchloride, bulk polymerised	0.00087	kg	ecoinvent assumption
soap	0.00088	kg	ecoinvent assumption
sodium hydroxide, without water, in 50% solution state	0.0028	kg	ecoinvent assumption
spent anion exchange resin from potable water production	-0.058	kg	ecoinvent assumption
steel, low-alloyed, hot rolled	0.15	kg	ecoinvent assumption
uranium enrichment centrifuge facility	2.22E-08	unit	ecoinvent assumption
**uranium hexafluoride, WNA**	**1.20**	**kg**	**Global average (WNA 2019)**
waste mineral oil	-0.0024	kg	ecoinvent assumption
treatment of municipal solid waste, sanitary landfill	-0.235	kg	ecoinvent assumption

Conversion generates low-level radioactive waste, 90% of which is directed to interim storage, while 9% is incinerated (plasma torch) and 1% is surface or trench-deposited, as assumed in [11]. The original model assumes the same shares, with the plasma torch incineration being modelled on the Zwilag treatment plant in Würenlingen, Switzerland[1]. Radioactive emissions from the waste treatment were adjusted from 1.66 and 3.04 GBq/m^3 of carbon-14 and tritium, respectively (1993 data) to 0.04 and 8.40 GBq/m^3 (2017 data, from [12], assuming a constant throughput of waste, i.e. 5 m^3/year).

[1] More details on the facility at https://www.zwilag.ch/en/function-of-facility-_content---1--1065.html

Enrichment

Two main technologies used to dominate the uranium enrichment market: gaseous diffusion, and centrifugation. Both techniques exploit the difference of molecular mass between ^{235}U and ^{238}U to achieve separation and increase the enrichment rate (of ^{235}U, the fissile isotope) of the product. As the molecular masses of the two isotopes is minuscule (about 0.4%), enrichment is an energy-intensive process. The unit conventionally used to quantify the amount of enrichment work, used to separate enriched (product) and depleted (tails) uranium from an instream (feed) of natural uranium is the "separative work unit" (SWU).

Enrichment processes involve the separation of a feed of UF6 into two outputs with different $^{235}U/^{238}U$ isotope concentrations, the enriched product and the depleted tails. Depending on the feed assay (the original concentration), the desired enrichment rate and the tails assay, a centrifuge, or more likely an array thereof, will provide a variable amount of work. Following Glaser [13], we write the mass balance of the enrichment process as:

$$\boldsymbol{FN_F = PN_P + WN_w}$$

We use the notations of [13] where $\boldsymbol{F}$, $\boldsymbol{P}$, and $\boldsymbol{W}$ are the feed, product, and tails streams, typically in kg/year, and $\boldsymbol{N_X}$ are the respective fraction of the fissile material ^{235}U, in each stream. We define the *cut* $\boldsymbol{\theta}$ as the proportion of the feed exiting the process as product, i.e. $\boldsymbol{P = \theta F}$. It can be shown that the cut is dependent on the various rates $\boldsymbol{N_X}$, and is therefore fixed for a given configuration. The work (energy) needed to enrich or deplete a flow is defined through the function $\boldsymbol{V(N)}$, which obeys the following equation:

$$\boldsymbol{\delta U = PV(N_P) + WV(N_W) - FV(N_F)}$$

Where $\boldsymbol{\delta U}$ is the separative power for producing quantity P from quantity F. There is no exact analytical expression for $\boldsymbol{V(N)}$ but using Taylor series, its second derivative can be estimated, from which $\boldsymbol{V(N)}$ is given the standard expression:

$$\boldsymbol{V(N) = (2N - 1)\ln\left(\frac{N}{1 - N}\right)}$$

Combining the two latter equations, the amount of SWU per enriched material can be computed as $\boldsymbol{\frac{\delta U}{P}}$, which after simplification yields the following expression:

$$\boldsymbol{\frac{\delta U}{P} = SWU = V(N_P) - V(N_W) + \frac{N_P - N_W}{N_F - N_W}\left(V(N_W) - V(N_F)\right)}$$

This value is used in the life cycle inventories.

A few examples:

- 1 kg UF6 at $\boldsymbol{N_P = 3.8}$% and $\boldsymbol{N_W = 0.20}$% tails assay requires 6.09 SWU, from 7.05 kg feed,
- 1 kg UF6 at $\boldsymbol{N_P = 5.0}$% and $\boldsymbol{N_W = 0.25}$% tails assay requires 7.92 SWU, from 10.3 kg feed.

Depending on the actual technique used, the energy value of a SWU can span from about 40 kWh/SWU for gas centrifugation, to more than 2 MWh/SWU in gas diffusion techniques. Here, The feed contains the natural ^{235}U content of 0.71%, tails are usually about 0.22%; with these parameters 1 SWU could enrich fuel to about 1.41%.

Fuel fabrication

Table S8. *Inputs for fuel fabrication, per kg fuel element.*

Inputs	Amount	Unit	Comment
Cement	0.0065	kg	ecoinvent 3.7
Chromium	0.6	kg	ecoinvent 3.7
Electricity, medium voltage	50	kWh	From WNA consultation
Uranium, enriched, per SWU	6.74	SWU	Mass balance calculation
Water, decarbonised	300	kg	ecoinvent 3.7

Construction

The construction phase contains only a single parameter, which scales all of material flows up or down in the same proportion. The dependence between the intensity in material inputs can be explained by the fact that more cables would require simultaneously more steel, aluminum and copper, a thicker reactor containment building would require simultaneously more concrete and reinforcing steel, etc. The initial average values are the results of a review [11, 14-18], as shown on **Figure S5**.

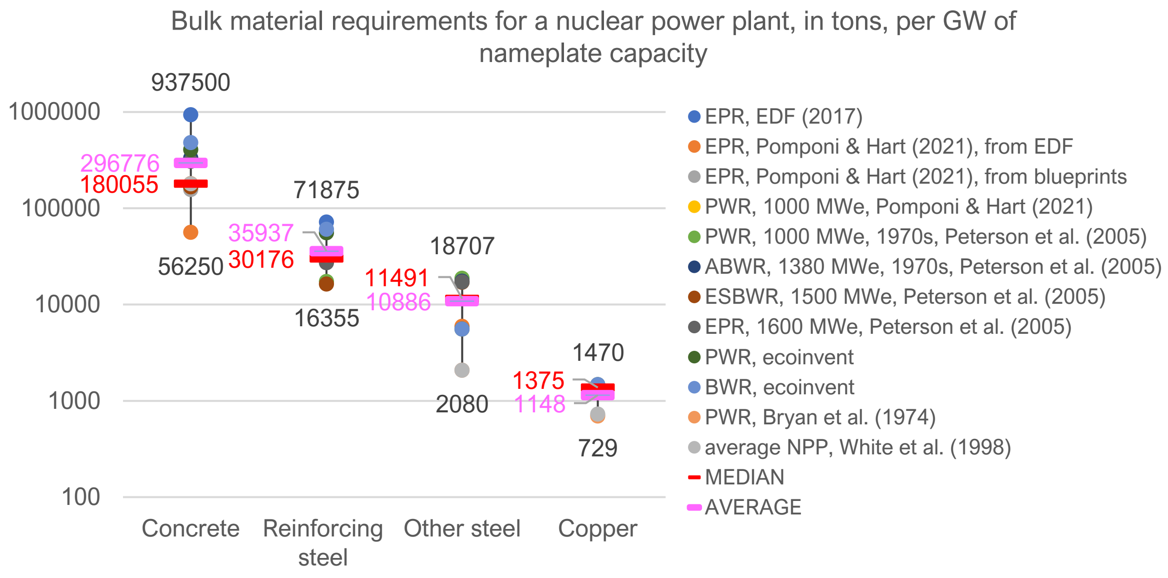

Figure S5. *Bulk material requirements for a nuclear power plant, in tons per GW of nameplate capacity.*

Table S9. *Inputs for NPP construction, 1000 MW reactor.*

Inputs	Amount	Unit	Comment
concrete production, normal	123657	m3	Average of literature (see Figure 6 in main text)
copper, cathode	1147600	kg	Average of literature (see Figure 6 in main text)
reinforcing steel production	35936572	kg	Average of literature (see Figure 6 in main text)
steel production, low-alloyed, hot rolled	10885813	kg	Average of literature (see Figure 6 in main text)
aluminium, cast alloy	64000	kg	ecoinvent assumption
excavation, hydraulic digger	85000	m3	ecoinvent assumption
electricity, low voltage	531000000	kWh	ecoinvent assumption
diesel, burned in building machine	190000000	MJ	ecoinvent assumption
inert waste, for final disposal	-322000000	kg	ecoinvent assumption
heat, district or industrial, other than natural gas	135850000	MJ	ecoinvent assumption

Operation

As the key component of nuclear fuel is the ^{235}U content, the relationship between burnup rate and enrichment should be approximately linear, but long-term studies show that high variations can be found between the two quantities. Hu *et al.* [19] show that fuel burnup and enrichment rate have concomitantly and steadily increased over the 1968–2013 period in US reactors, but without a strict linear relationship between the two, for the following reasons: nonroutine reactor operations (e.g. premature shutdown of reactors), leaking fuel rods prematurely replaced, or the fact that assemblies used in test programs can allow burnup rates higher than what is achievable under normal operations.

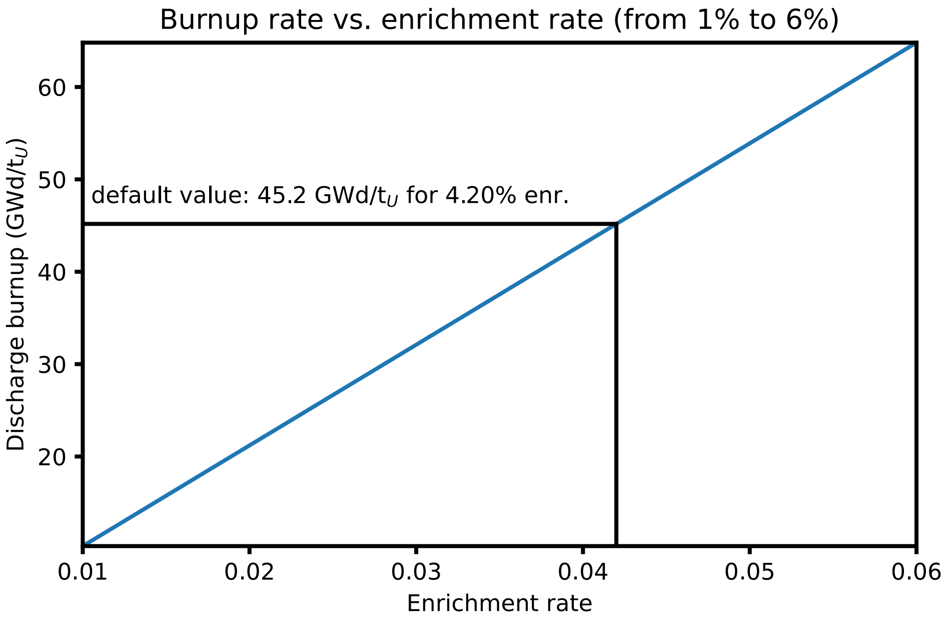

Figure S6. *Relationship between discharge burnup rate and enrichment rate, following the model of Burns et al. [20].*

Burns *et al.* [20] propose a linear model for burnup-enrichment relationship, with a slope of 10.9 GWd/tU per % of enrichment, i.e., the enrichment range commonly found in most fuel assemblies of 3%–5% would yield 32–54 GWd/tU respectively – which is retained for the model. The overall thermal efficiency of nuclear power plants spans over a much narrower range, usually around 33%, as found in the literature survey. It is kept as a parameter nonetheless.

Table S10. *Chemical inputs for NPP operation, 1000 MW reactor.*

Inputs	Amount	Unit	Comment
argon, liquid	3.23E-05	kg	ecoinvent assumption
boric acid, anhydrous, powder	2.38E-06	kg	WNA consultation
carbon dioxide, liquid	2.07E-07	kg	ecoinvent assumption
chemical, inorganic	2.90E-06	kg	ecoinvent assumption
hydrogen liquid, production mix	2.14E-05	kg	WNA consultation
hydrazine	5.02E-07	kg	WNA consultation
nitrogen, liquid	7.65E-05	kg	ecoinvent assumption
oxygen, liquid	2.07E-05	kg	ecoinvent assumption
sodium hypochlorite, without water, in 15% solution state	8.89E-06	kg	WNA consultation
sodium hydroxide, without water, in 50% solution state	8.94E-07	kg	WNA consultation
acetylene	4.46E-08	kg	ecoinvent assumption
anionic resin	7.97E-08	kg	ecoinvent assumption
cationic resin	7.97E-08	kg	ecoinvent assumption
chemical, organic	1.71E-06	kg	ecoinvent assumption
lubricating oil	2.01E-06	kg	ecoinvent assumption
cement, production mix	1.14E-06	kg	ecoinvent assumption
pitch	9.56E-07	kg	ecoinvent assumption
diesel, burned in diesel-electric generating set	1.48E-03	MJ	WNA consultation
paper, woodfree, coated	7.97E-08	kg	ecoinvent assumption

Spent fuel management

Table S11. *Inputs for interim storage of spent fuel, per TWh of average NPP operation.*

Inputs	Amount	Unit	Comment
petrol, low-sulfur	1.00E+01	kg	From WNA consultation
diesel, burned in building machine	8.41E+03	MJ	From WNA consultation
hazardous waste, for incineration	1.11E+02	kg	From WNA consultation
inert waste, for final disposal	1.88E+02	kg	From WNA consultation
water, decarbonised	3.12E+02	kg	From WNA consultation
electricity, high voltage	3.78E+05	kWh	From WNA consultation
chemicals, inorganic	2.11E-01	kg	From WNA consultation
acrylic dispersion, without water, in 65% solution state	1.34E-02	kg	From WNA consultation
butyl acetate	8.50E-02	kg	From WNA consultation
ethanol, without water, in 99.7% solution state, from fermentation	6.38E+00	kg	From WNA consultation
ethyl acetate	4.67E-02	kg	From WNA consultation
hydrazine	3.00E-01	kg	From WNA consultation
isopropanol	2.05E+00	kg	From WNA consultation
lubricating oil	9.05E-02	kg	From WNA consultation
methyl ethyl ketone	2.83E-03	kg	From WNA consultation
methyl methacrylate	1.59E-03	kg	From WNA consultation
refrigerant R134a	3.10E-01	kg	From WNA consultation
silicone product	5.56E-02	kg	From WNA consultation
soap	3.59E+00	kg	From WNA consultation
anionic resin	9.73E+01	kg	From WNA consultation
monoethanolamine	6.80E-03	kg	From WNA consultation
sodium chloride, powder	1.70E+00	kg	From WNA consultation
ethylene glycol	5.35E-01	kg	From WNA consultation

Final waste disposal

High-level radioactive waste disposal is the last step of the so-called “backend” part of the uranium fuel chain. The most popular solution for long-term waste disposal is storage in a deep geological repository. The most advanced such repository as of 2022 is the Onkalo site, situated near the Olkiluoto power plant in Finland, which should begin operations in 2023. Spent fuel needs to be conditioned before final storage. Here we have chosen to model Vattenfall’s encapsulation process, which consists in enclosing spent fuel in copper-cast iron canisters. Two designs exist depending on the copper-to-insert (cast iron) ratio, both designs can contain 3.6 tons of spent fuel for a total weight of 24.3-24.6 tons [21]. We use the 50-mm copper design for the LCA model. Each canister can contain 3600 kg of spent fuel elements, consisting of UO_2 in their zirconium envelope. The uranium fuel chain model shows that 2.92 mg of uranium in fuel elements is required per kWh of electricity, which translates to 3.31 mg of UO_2, or 7.98 mg of fuel elements including the zircalloy envelope. About 2.2 canisters are therefore needed per TWh of electricity output.

Table S12. *Inputs for one spent fuel canister.*

Inputs	Amount	Unit	Comment
copper, cathode	7400	kg	Hedman *et al.* [21]
cast iron	13600	kg	Hedman *et al.* [21]
welding, arc, aluminium	3.30	m	Assuming welding around the cap (diameter 1050 mm) and approximating fusion welding with arc welding

Table S13. *Inputs for encapsulation of spent fuel from interim storage, per TWh of NPP operation.*

Inputs	Amount	Unit	Comment
Spent fuel canister	2.2	unit	From WNA consultation
diesel, burned in diesel-electric generating set, 10MW	1448	MJ	From WNA consultation
ethanol, without water, in 95% solution state, from fermentation	0.028	kg	From WNA consultation
lubricating oil	0.81	kg	From WNA consultation
soap	4.4	kg	From WNA consultation
electricity, medium voltage	310282	kWh	From WNA consultation

Table S14. *Inputs for deep waste repository, per TWh of NPP operation.*

Inputs	Amount	Unit	Comment
market group for concrete, normal	2.59	m3	From WNA consultation
blasting	1140	kg	From WNA consultation
diesel, burned in diesel-electric generating set, 10MW	52640	MJ	From WNA consultation
light fuel oil	9984	kg	From WNA consultation
electricity, medium voltage	738766	kWh	From WNA consultation
reinforcing steel	113	kg	From WNA consultation

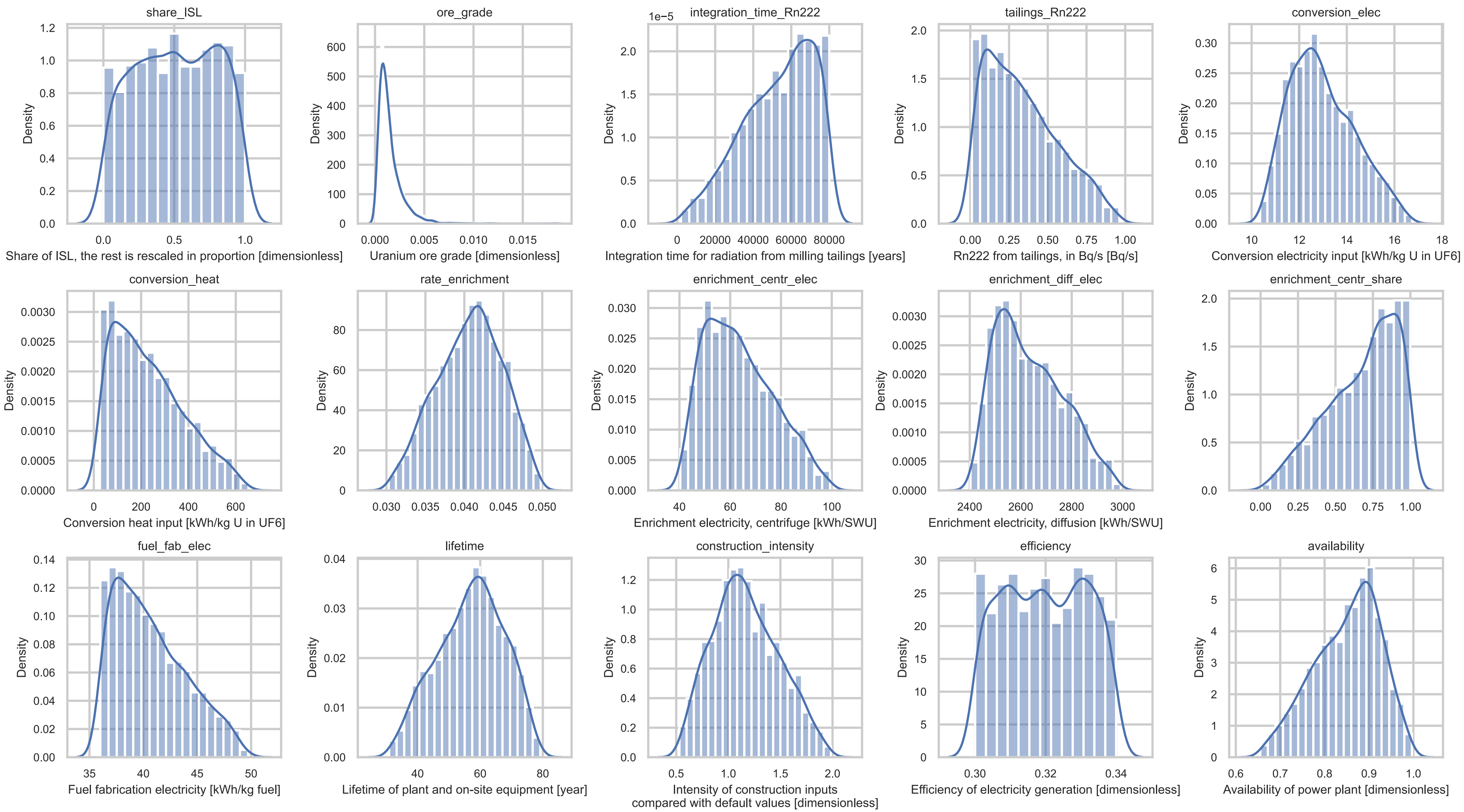

Figure S7. *Parameters of the model and their distributions.*

Life cycle impact assessment

Table S15. *Life cycle impact assessment categories.*

Category	Unit	Reference	Description
Climate change	kg CO_2 eq.	IPCC (2013)	Radiative forcing as global warming potential, integrated over 100 years (GWP100), based on IPCC baseline model.
Freshwater eutrophication	kg P eq.	EUTREND, Struijs *et al.* [22]	Expression of the degree to which the emitted nutrients reach the freshwater end compartment. As the limiting nutrient in freshwater aquatic ecosystems, a surplus of phosphorus will lead to eutrophication.
Ionizing radiation	kBq ^{235}U eq	Frischknecht *et al.* [23]	Human exposure efficiency relative to ^{235}U radiation. The original model is Dreicer *et al.* [24] and follows the linear no-threshold paradigm to account for low dose radiation.
Human toxicity	CTUh (comparative toxic units for humans)	USEtox 2.1. model Rosenbaum *et al.* [25]	"The characterization factor for human toxicity impacts (human toxicity potential) is expressed in comparative toxic units (CTUh), the estimated increase in morbidity in the total human population, per unit mass of a chemical emitted, assuming equal weighting between cancer and non-cancer due to a lack of more precise insights into this issue. Unit: [CTUh per kg emitted] = [disease cases per kg emitted]"[2]
Ecotoxicity, freshwater	CTUe (comparative toxic units for ecosystems)		"The characterization factor for aquatic ecotoxicity impacts (ecotoxicity potential) is expressed in comparative toxic units (CTUe), an estimate of the potentially affected fraction of species (PAF) integrated over time and volume, per unit mass of a chemical emitted.<br><br>Unit: [CTUe per kg emitted] = [PAF × m³ × day per kg emitted]" [2]
Land use	points	LANCA model, Bos *et al.* [26]	The LANCA model provides five indicators for assessing the impacts due to the use of soil: 1. erosion resistance, 2. mechanical filtration, 3. physicochemical filtration, 4. groundwater regeneration and 5. biotic production
Water resource depletion	m^3	Swiss Ecoscarcity Frischknecht *et al.* [27]	Water use related to local consumption of water.<br><br>Note: only air emissions are accounted for.<br><br>In this method, all flows have an identical characterization factor of 42.95 m^3/m^3 of water consumed
Mineral, fossil and renewable resource depletion	kg Sb eq.	Van Oers *et al.* [28]	Scarcity of resource in relation to that of antimony. Scarcity is calculated as « reserve base ».

A note on ionizing radiation

When ionizing radiation is considered, radon-222 emissions and radiation integration time are highly significant. Ionizing radiation is an indicator seldom assessed, or at least analyzed in detail in LCA. The latest characterization factors were published by Frischknecht *et al.* [23], relying on a 1995 report [24], using the so-called "linear no-threshold" (LNT) model that assumes that human health impacts occur from the first radionuclide emissions. Although conservative and precautionary, this LNT model has been criticized as no effects of radiation have been discerned under 100 mSv of received dose[29]. As life cycle impact assessment is by construction linear, it suffers the same criticism. Similarly, the question of integration time raises the question of whether radionuclide emissions will still be of concern to humanity in 80000 years (when climate change commonly uses a 100-year horizon).

[2] From USEtox FAQ, available at https://usetox.org/faq

Additional results

Variability of lifecycle impact results with respect to ore grade

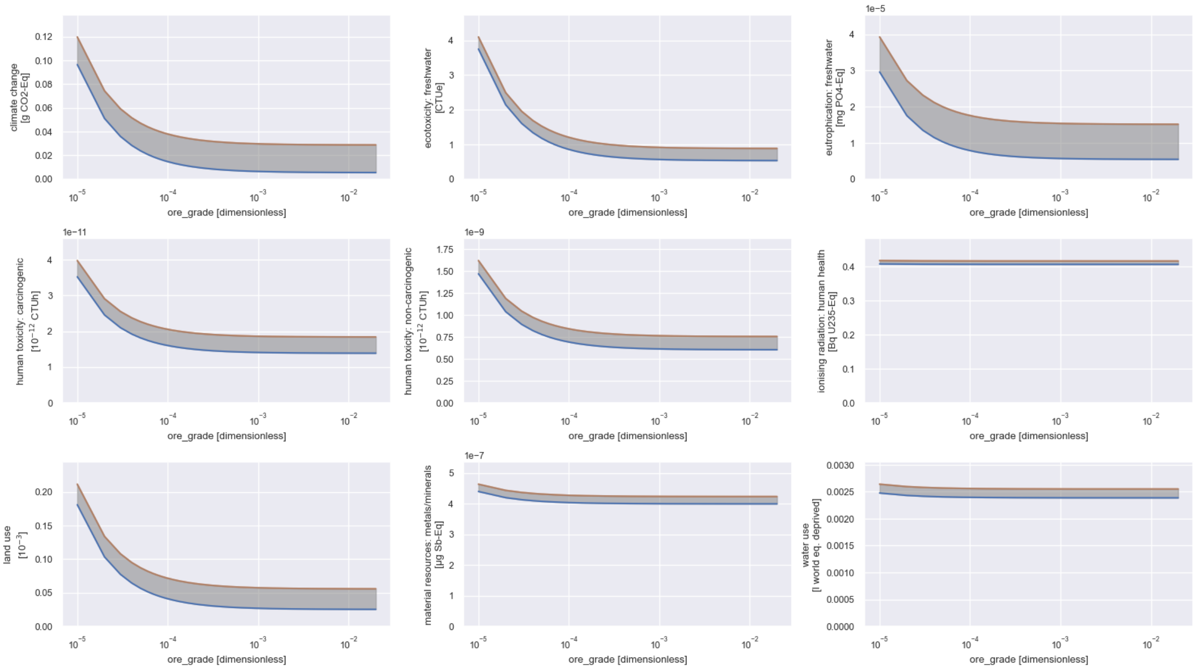

Figure S8. *Variability of environmental impact with respect to ore grade, from 10 ppm (0.00001) to 20% (0.2), for the two enrichment techniques, gaseous diffusion (orange) and centrifugation (blue). Any enrichment mix other than 100%/0% or 0%/100% will lead to a result in the grey area between the curves.*

Variability of lifecycle impact results with respect to extraction technique (ISL)

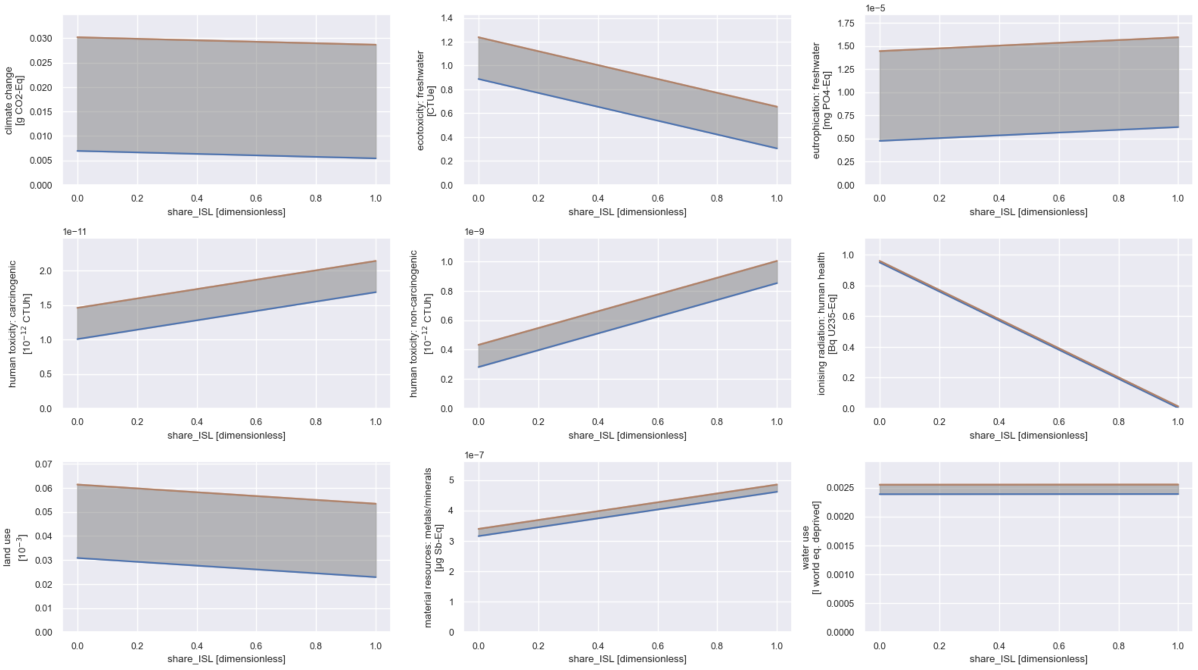

Figure S9. *Variability of environmental impact with respect to extraction technique (ISL), from 0% to 100% ISL, for the two enrichment techniques, gaseous diffusion (orange) and centrifugation (blue). Any extraction mix other than 100%/0% or 0%/100% will lead to a result in the grey area between the curves.*

Extra results for simplified models

Table S16. *Simplified models, explaining 80% of the overall variance, with enrichment technique set to 100% centrifugation.*

Impact category	Unit (per kWh)	Simplified model
Climate change	kg CO2 eq.	$\begin{cases} \frac{1.0(efficiency ore_{grade}(0.0723\log(100ore_{grade})^2-5.196)(0.04562construction_{intensity}+0.001093lifetime+0.002532)e^{0.707ore_{grade}}+lifetime(7.717\cdot10^{-6}ore_{grade}share_{ISL}((5.121\log(100ore_{grade})^2-368.1)e^{0.0485ore_{grade}}-141.4)e^{0.658ore_{grade}}+0.0005519ore_{grade}(0.0723\log(100ore_{grade})^2-5.196)e^{0.707ore_{grade}}-(share_{ISL}-1.0)(3.442\cdot10^{-6}ore_{grade}((0.5629\log(100ore_{grade})^2-40.45)e^{0.482ore_{grade}}-176.1)e^{0.176ore_{grade}}+4.276\cdot10^{-6}ore_{grade}((5.288\log(100ore_{grade})^2-380.0)e^{0.176ore_{grade}}-203.8)e^{0.482ore_{grade}}+(0.0002014ore_{grade}+7.043\cdot10^{-7})(0.0723\log(100ore_{grade})^2-5.196)e^{0.658ore_{grade}})e^{0.0485ore_{grade}}))e^{-0.707ore_{grade}}}{efficiency lifetime ore_{grade}(0.0723\log(100ore_{grade})^2-5.196)} & \text{for } ore_{grade} < 0.01 \\ \frac{0.04562construction_{intensity}}{lifetime}+0.001093+\frac{0.002532}{lifetime}+\frac{5.774\cdot10^{-6}share_{ISL}}{efficiency}-\frac{0.0001167share_{ISL}e^{-0.482ore_{grade}}}{efficiency}-\frac{0.0001677share_{ISL}e^{-0.176ore_{grade}}}{efficiency}+\frac{0.00021share_{ISL}e^{-0.0485ore_{grade}}}{efficiency}+\frac{0.001093}{efficiency}+\frac{0.0001167e^{-0.482ore_{grade}}}{efficiency}+\frac{0.0001677e^{-0.176ore_{grade}}}{efficiency}-\frac{7.043\cdot10^{-7}share_{ISL}}{efficiency ore_{grade}}+\frac{7.043\cdot10^{-7}}{efficiency ore_{grade}} & \text{otherwise} \end{cases}$
Freshwater ecotoxicity	CTUe	$0.9376-0.6161share_{ISL}$
Freshwater eutrophication	kg P	$\begin{cases} \frac{(-2.408\cdot10^{-5}lifetime ore_{grade}share_{ISL}(0.002038mining_{electricityswitchdieselgenerator}+0.1277mining_{electricityswitchgrid}+(0.7779-0.01082\log(100ore_{grade})^2)e^{0.0485ore_{grade}}+0.001484)e^{0.658ore_{grade}}+1.0lifetime(share_{ISL}-1.0)(1.074\cdot10^{-5}ore_{grade}(0.002538mining_{electricityswitchdieselgenerator}+0.159mining_{electricityswitchgrid}+(0.009123-0.0001269\log(100ore_{grade})^2)e^{0.482ore_{grade}}+0.001848)e^{0.176ore_{grade}}+1.334\cdot10^{-5}ore_{grade}(0.002936mining_{electricityswitchdieselgenerator}+0.184mining_{electricityswitchgrid}+(0.2387-0.003321\log(100ore_{grade})^2)e^{0.176ore_{grade}}+0.002138)e^{0.482ore_{grade}}-(1.055\cdot10^{-6}ore_{grade}+6.299\cdot10^{-10})(0.0723\log(100ore_{grade})^2-5.196)e^{0.658ore_{grade}})e^{0.0485ore_{grade}}+1.0ore_{grade}(0.0723\log(100ore_{grade})^2-5.196)(3.915\cdot10^{-5}construction_{intensity}+2.158\cdot10^{-6}lifetime+6.357\cdot10^{-7})e^{0.707ore_{grade}})e^{-0.707ore_{grade}}}{lifetime ore_{grade}(0.0723\log(100ore_{grade})^2-5.196)} & \text{for } ore_{grade} < 0.01 \\ \frac{(2.408\cdot10^{-5}lifetime ore_{grade}share_{ISL}(0.0003921mining_{electricityswitchdieselgenerator}+0.02457mining_{electricityswitchgrid}+0.1497e^{0.0485ore_{grade}}+0.0002856)e^{0.658ore_{grade}}+2.158\cdot10^{-6}lifetime ore_{grade}e^{0.707ore_{grade}}-1.0lifetime(share_{ISL}-1.0)(1.074\cdot10^{-5}ore_{grade}(0.0004884mining_{electricityswitchdieselgenerator}+0.03061mining_{electricityswitchgrid}+0.001756e^{0.482ore_{grade}}+0.0003558)e^{0.176ore_{grade}}+1.334\cdot10^{-5}ore_{grade}(0.0005651mining_{electricityswitchdieselgenerator}+0.03541mining_{electricityswitchgrid}+0.04594e^{0.176ore_{grade}}+0.0004116)e^{0.482ore_{grade}}+1.055\cdot10^{-6}ore_{grade}e^{0.658ore_{grade}}+6.299\cdot10^{-10}e^{0.658ore_{grade}})e^{0.0485ore_{grade}}+1.0ore_{grade}(3.915\cdot10^{-5}construction_{intensity}+6.357\cdot10^{-7})e^{0.707ore_{grade}})e^{-0.707ore_{grade}}}{lifetime ore_{grade}} & \text{otherwise} \end{cases}$
Human toxicity (carcinogenic)	CTUh	$6.898\cdot10^{-12}share_{ISL}+1.077\cdot10^{-11}$
Human toxicity (non-carcinogenic)	CTUh	$5.842\cdot10^{-10}share_{ISL}+2.998\cdot10^{-10}$
Ionising radiation	kg ^{235}U eq.	$-94.5tailings_{Rn222}(share_{ISL}-1.0)\left(e^{9.2\cdot10^{-6}integration_{timeRn222}}-1.0\right)e^{-9.2\cdot10^{-6}integration_{timeRn222}}$
Land use	dimensionless	$\begin{cases} \frac{(2.415\cdot10^{-5}lifetime ore_{grade}share_{ISL}((23.74\log(100ore_{grade})^2-1706.0)e^{0.0485ore_{grade}}-176.7)e^{0.658ore_{grade}}-1.0lifetime(share_{ISL}-1.0)(1.338\cdot10^{-5}ore_{grade}((6.927\log(100ore_{grade})^2-497.8)e^{0.176ore_{grade}}-254.6)e^{0.482ore_{grade}}+1.077\cdot10^{-5}ore_{grade}((18.21\log(100ore_{grade})^2-1309.0)e^{0.482ore_{grade}}-220.0)e^{0.176ore_{grade}}+(0.009409ore_{grade}+3.704\cdot10^{-6})(0.0723\log(100ore_{grade})^2-5.196)e^{0.658ore_{grade}})e^{0.0485ore_{grade}}+1.0ore_{grade}(0.008631lifetime+0.3932)(0.0723\log(100ore_{grade})^2-5.196)e^{0.707ore_{grade}})e^{-0.707ore_{grade}}}{lifetime ore_{grade}(0.0723\log(100ore_{grade})^2-5.196)} & \text{for } ore_{grade} < 0.01 \\ -0.005473share_{ISL}-0.0004562share_{ISL}e^{-0.482ore_{grade}}-0.0006557share_{ISL}e^{-0.176ore_{grade}}+0.0008214share_{ISL}e^{-0.0485ore_{grade}}+0.02204+0.0004562e^{-0.482ore_{grade}}+0.0006557e^{-0.176ore_{grade}}-\frac{3.704\cdot10^{-6}share_{ISL}}{ore_{grade}}+\frac{3.704\cdot10^{-6}}{ore_{grade}}+\frac{0.3932}{lifetime} & \text{otherwise} \end{cases}$
Material resources	kg Sb eq.	$1.497\cdot10^{-7}share_{ISL}+3.263\cdot10^{-7}$
Water use	l	$2.355river_{cooling}+0.09439$

Table S17. *Simplified models, explaining 80% of the overall variance, with enrichment technique set to 100% centrifugation and ore grade set to its default value of 1544 ppm.*

Impact category	Unit (per kWh)	Simplified model
Climate change	kg CO2 eq.	$0.001093+\frac{0.00253+0.0456\cdot construction_{intensity}}{lifetime}+\frac{0.00185-0.000528\cdot share_{ISL}}{efficiency}$
Freshwater ecotoxicity	CTUe	$0.938-0616\cdot share_{ISL}$
Freshwater eutrophication	kg P	$4.26\cdot10^{-6}+mining_{diesel}(1.34\cdot10^{-8}-3.51\cdot10^{-9}\cdot share_{ISL})+mining_{grid}(8.42\cdot10^{-7}-2.20\cdot10^{-7}\cdot share_{ISL})+\frac{6.36\cdot10^{-7}+3.92\cdot10^{-5}\cdot construction_{intensity}}{lifetime}$
Human toxicity (carcinogenic)	CTUh	$1.08\cdot10^{-11}+6.90\cdot10^{-12}\cdot share_{ISL}$
Human toxicity (non-carcinogenic)	CTUh	$3.00\cdot10^{-10}+5.84\cdot10^{-10}\cdot share_{ISL}$
Ionising radiation	kg ^{235}U eq.	$94.5\cdot tailings_{Rn222}(1-share_{ISL})(1-e^{-9.2\cdot10^{-6}integration_{time}})$
Land use	dimensionless	$0.0256-0.00818share_{ISL}+\frac{0.393}{lifetime}$
Material resources	kg Sb eq.	$3.26\cdot10^{-7}+1.50\cdot10^{-7}\cdot share_{ISL}$
Water use	l	$0.0944+2.36\cdot river_{cooling}$

References

1. Gibon, T., A. Arvesen, and E.G. Hertwich, *Life cycle assessment demonstrates environmental co-benefits and trade-offs of low-carbon electricity supply options.* Renewable and Sustainable Energy Reviews, 2017. **76**: p. 1283-1290.
2. Haque, N. and T. Norgate, *The greenhouse gas footprint of in-situ leaching of uranium, gold and copper in Australia.* Journal of Cleaner Production, 2014. **84**: p. 382-390 Available from: https://www.sciencedirect.com/science/article/pii/S0959652613006367.
3. Monnet, A., S. Gabriel, and J. Percebois, *Statistical model of global uranium resources and long-term availability.* EPJ Nuclear Sciences & Technologies, 2016. **2**: p. 17.
4. Grancea, L., et al., *Uranium Resources, Production and Demand 2020*. 2020, Organisation for Economic Co-Operation and Development.
5. Mudd, G.M. and M. Diesendorf, *Sustainability of uranium mining and milling: toward quantifying resources and eco-efficiency.* Environmental Science & Technology, 2008. **42**(7): p. 2624-2630.
6. Wernet, G., et al., *The ecoinvent database version 3 (part I): overview and methodology.* The International Journal of Life Cycle Assessment, 2016. **21**(9): p. 1218-1230.
7. Parker, D.J., C.S. McNaughton, and G.A. Sparks, *Life Cycle Greenhouse Gas Emissions from Uranium Mining and Milling in Canada.* Environmental Science & Technology, 2016. **50**(17): p. 9746-9753 Available from: https://doi.org/10.1021/acs.est.5b06072.
8. Farjana, S.H., et al., *Comparative life-cycle assessment of uranium extraction processes.* Journal of Cleaner Production, 2018. **202**: p. 666-683 Available from: https://www.sciencedirect.com/science/article/pii/S0959652618324478.
9. World Information Service on Energy. *Nuclear Fuel Energy and CO_2 Balance Calculator*. 2009; Available from: http://www.wise-uranium.org/nfceh.html.
10. International Energy Agency, *Energy Technology Perspectives 2020*. 2020.
11. Dones, R., et al., *Life Cycle Inventories for the Nuclear and Natural Gas Energy Systems, and Examples of Uncertainty Analysis (14 pp).* The International Journal of Life Cycle Assessment, 2005. **10**(1): p. 10-23 Available from: https://doi.org/10.1065/lca2004.12.181.2.
12. OSPAR Commission, *Seventh Swiss Implementation Report of PARCOM Recommendation 91/4 on radioactive discharges*. 2019 Available from: https://www.ospar.org/documents?v=40960.
13. Glaser, A., *Characteristics of the gas centrifuge for uranium enrichment and their relevance for nuclear weapon proliferation.* Science & Global Security, 2008. **16**(1-2): p. 1-25.
14. EDF, *EDF Energy sets out progress at Hinkley Point C new nuclear power station*. 2017 Available from: https://www.edf.fr/sites/groupe/files/contrib/groupe-edf/espaces-dedies/espace-medias/cp/2017/cp_edf_20170331_hinkley_va.pdf.
15. Pomponi, F. and J. Hart, *The greenhouse gas emissions of nuclear energy – Life cycle assessment of a European pressurised reactor.* Applied Energy, 2021. **290**: p. 116743 Available from: https://www.sciencedirect.com/science/article/pii/S0306261921002555.
16. Peterson, P.F., H. Zhao, and R. Petroski, *Metal and concrete inputs for several nuclear power plants.* University of California Berkeley, Report UCBTH-05-001, 2005.
17. Bryan, R. and I. Dudley, *Estimated quantities of materials contained in a 1000-MW (e) PWR Power Plant*. 1974, Oak Ridge National Lab., Tenn.(USA).
18. White, S.W. and G.L. Kulcinski, *Birth to death analysis of the energy payback ratio and CO2 gas emission rates from coal, fission, wind, and DT-fusion electrical power plants.* Fusion engineering and design, 2000. **48**(3-4): p. 473-481.
19. Hu, J., et al., *US Commercial Spent Nuclear Fuel Assembly Characteristics-1968-2013.* 2016.
20. Burns, J.R., et al., *Reactor and fuel cycle performance of light water reactor fuel with 235U enrichments above 5%.* Annals of Nuclear Energy, 2020. **142**: p. 107423 Available from: https://www.sciencedirect.com/science/article/pii/S0306454920301213.
21. Hedman, T., A. Nyström, and C. Thegerström, *Swedish containers for disposal of spent nuclear fuel and radioactive waste.* Comptes Rendus Physique, 2002. **3**(7-8): p. 903-913.
22. Struijs, J., et al., *Aquatic eutrophication*. 2009, Chapter.
23. Frischknecht, R., et al., *Human health damages due to ionising radiation in life cycle impact assessment.* Environmental Impact Assessment Review, 2000. **20**(2): p. 159-189 Available from: https://www.sciencedirect.com/science/article/pii/S0195925599000426.

24. Dreicer, M., V. Tort, and H. Margerie, *The external costs of the nuclear fuel cycle: implementation in France.* 1995.
25. Rosenbaum, R.K., et al., *USEtox—the UNEP-SETAC toxicity model: recommended characterisation factors for human toxicity and freshwater ecotoxicity in life cycle impact assessment.* The International Journal of Life Cycle Assessment, 2008. **13**(7): p. 532-546.
26. Bos, U., et al., *LANCA®-characterization factors for life cycle impact assessment: version 2.0*. 2016: Fraunhofer Verlag Stuttgart.
27. Frischknecht, R., et al., *Swiss ecological scarcity method: the new version 2006.* 2006.
28. Van Oers, L., et al., *Abiotic resource depletion in LCA*. 2002, Road and Hydraulic Engineering Institute, Ministry of Transport and Water, Amsterdam.
29. Sacks, B., G. Meyerson, and J.A. Siegel, *Epidemiology Without Biology: False Paradigms, Unfounded Assumptions, and Specious Statistics in Radiation Science (with Commentaries by Inge Schmitz-Feuerhake and Christopher Busby and a Reply by the Authors).* Biological theory, 2016. **11**: p. 69-101 Available from: https://pubmed.ncbi.nlm.nih.gov/27398078.
