# Supplementary material for: Parametric Life Cycle Assessment of Nuclear Power for Simplified Models
Source: Environ Sci Technol. 2023 Sep 12;57(38):14194–205. doi: 10.1021/acs.est.3c03190 (PMC10537461; doi:10.1021/acs.est.3c03190)
Supplement: Supplementary file 4 — es3c03190_si_004.zip [file es3c03190_si_004.zip › 1_setup.html]

1\_setup


# LCA database for electricity generation technologies¶

This notebook is setting up the ecoinvent database, making sure it can be parameterized with prospective scenarios for electricity mixes. Once all tests have passed, the database can be used for the assessment of electricity technologies in the project.

This setup procedure is uniquely dedicated to the modelling of nuclear power for the corresponding publication "Fully-parameterized life cycle assessment of nuclear power for simplified models".

References

- **Main results**: UNECE (2022) *Life Cycle Assessment of Electricity Generation Options*, available from https://unece.org/sites/default/files/2022-04/LCA\_3\_FINAL%20March%202022.pdf
- **Original inventories** (before update): UNEP International Resource Panel (2016) *Green Energy Choices*, available from https://www.resourcepanel.org/reports/green-energy-choices-benefits-risks-and-trade-offs-low-carbon-technologies-electricity
- **Full scale assessment**: Hertwich, E. G., Gibon, T., Bouman, E. A., Arvesen, A., Suh, S., Heath, G. A., ... & Shi, L. (2015). Integrated life-cycle assessment of electricity-supply scenarios confirms global environmental benefit of low-carbon technologies. *Proceedings of the National Academy of Sciences*, 112(20), 6277-6282. https://doi.org/10.1073/pnas.1312753111
- **Methods (THEMIS model construction)**: Gibon, T., Wood, R., Arvesen, A., Bergesen, J. D., Suh, S., & Hertwich, E. G. (2015). A methodology for integrated, multiregional life cycle assessment scenarios under large-scale technological change. Environmental Science & Technology, 49(18), 11218-11226. https://doi.org/10.1021/acs.est.5b01558
- **IAM-LCA coupling**: Sacchi, R., Terlouw, T., Siala, K., Dirnaichner, A., Bauer, C., Cox, B., ... & Luderer, G. (2022). PRospective EnvironMental Impact asSEment (premise): A streamlined approach to producing databases for prospective life cycle assessment using integrated assessment models. Renewable and Sustainable Energy Reviews, 160, 112311.

Contact thomas.gibon@list.lu

In [1]:

```
# Let's get comfortable first
from IPython.core.display import display, HTML
display(HTML("<style>.container { width:80% !important; }</style>"))
```

```
C:\Users\Gibon\AppData\Local\Temp\ipykernel_16124\1931309167.py:2: DeprecationWarning: Importing display from IPython.core.display is deprecated since IPython 7.14, please import from IPython display
  from IPython.core.display import display, HTML
```

In [2]:

```
# This avoids a crash later in the code...
import os
os.environ["KMP_DUPLICATE_LIB_OK"]="TRUE"
```

In [3]:

```
# There are quite a few modules to import
import brightway2 as bw
import bw2analyzer as bwa
import bw2io
import openpyxl
import pandas as pd
import numpy as np
import scipy as sp
import pickle
from pypardiso import spsolve
import matplotlib.pyplot as plt
from matplotlib import cm
# import networkx as nx
import time
from premise import *
from importlib import reload 

# This is a local function used to easily write the database
from utils.database_writer import database_writer
```

In [4]:

```
# Set your environment variables
ECOINVENT_FOLDER = 'D:/Nextcloud/ecoinvent/ecoinvent 3.8_cutoff_ecoSpold02/datasets'
REMIND_KEY       = 'data/remind_key.txt'
```

In [5]:

```
# This forces svg to save text as text
plt.rcParams['svg.fonttype'] = 'none'
```

In [6]:

```
bw.projects.set_current('nuclear_param')
```

In [7]:

```
bw.databases
```

Out[7]:

```
Databases dictionary with 5 object(s):
	Nuclear_DB
	UNEP_IRP_EUR
	biosphere3
	ecoinvent 3.8 cutoff
	ecoinvent_remind_SSP2-Base_2020
```

In [8]:

```
bw.bw2setup()
```

```
Biosphere database already present!!! No setup is needed
```

In [9]:

```
bw.databases
```

Out[9]:

```
Databases dictionary with 5 object(s):
	Nuclear_DB
	UNEP_IRP_EUR
	biosphere3
	ecoinvent 3.8 cutoff
	ecoinvent_remind_SSP2-Base_2020
```

## Import ecoinvent data¶

The following is necessary if ecoinvent is not imported

In [9]:

```
if 'ecoinvent 3.8 cutoff' not in bw.databases:
    ei38 = bw.SingleOutputEcospold2Importer(ECOINVENT_FOLDER,
                                       db_name='ecoinvent 3.8 cutoff')
    ei38.apply_strategies()
    ei38.write_database()
```

In [10]:

```
bw.databases
```

Out[10]:

```
Databases dictionary with 3 object(s):
	biosphere3
	ecoinvent 3.8 cutoff
	ecoinvent_remind_SSP2-Base_2020
```

In [11]:

```
bio = bw.Database('biosphere3')
```

## Create REMIND-adjusted ecoinvent database¶

This is the kind of background we are going to use for the project, we will need to link inventories against it. Because the nuclear power inventory was built to be adapted to the REMIND regions, we (at least) need the regionalization, especially for Europe, which will be used as our standard region for the modelling.

In [12]:

```
with open(REMIND_KEY) as f:
    remind_key = f.readline()
```

In [13]:

```
ndb = NewDatabase(
            scenarios=[
                {"model":"remind", "pathway":"SSP2-Base", "year":2020} # We use the basic 
            ],
            source_db="ecoinvent 3.8 cutoff", # <-- name of the database in the BW2 project. Must be a string.
            source_version="3.8", # <-- version of ecoinvent. Can be "3.5", "3.6", "3.7" or "3.8". Must be a string.
            key=remind_key # <-- decryption key
            # to be requested from the library maintainers if you want ot use default scenarios included in `premise`
    )
```

```
premise v.(1, 3, 9)
+------------------------------------------------------------------+
| Warning                                                          |
+------------------------------------------------------------------+
| Because some of the scenarios can yield LCI databases            |
| containing net negative emission technologies (NET),             |
| it is advised to account for biogenic CO2 flows when calculating |
| Global Warming potential indicators.                             |
| `premise_gwp` provides characterization factors for such flows.  |
| It also provides factors for hydrogen emissions to air.          |
|                                                                  |
| Within your bw2 project:                                         |
| from premise_gwp import add_premise_gwp                          |
| add_premise_gwp()                                                |
+------------------------------------------------------------------+
+--------------------------------+----------------------------------+
| Utils functions                | Description                      |
+--------------------------------+----------------------------------+
| clear_cache()                  | Clears the cache folder. Useful  |
|                                | when updating `premise`or        |
|                                | encountering issues with         |
|                                | inventories.                     |
+--------------------------------+----------------------------------+
| get_regions_definition(model)  | Retrieves the list of countries  |
|                                | for each region of the model.    |
+--------------------------------+----------------------------------+
| ndb.NewDatabase(...)           | Generates a summary of the most  |
| ndb.generate_scenario_report() | important scenarios' variables.  |
+--------------------------------+----------------------------------+
Keep uncertainty data?
NewDatabase(..., keep_uncertainty_data=True)

Hide these messages?
NewDatabase(..., quiet=True)

//////////////////// EXTRACTING SOURCE DATABASE ////////////////////
Done!

////////////////// IMPORTING DEFAULT INVENTORIES ///////////////////
Done!

/////////////////////// EXTRACTING IAM DATA ////////////////////////
Done!
```

In [14]:

```
ndb.update_all()
```

```
`update_all()` will skip the following steps:
update_two_wheelers(), update_cars(), and update_buses()
If you want to update these steps, please run them separately afterwards.

////////////////// MEDIUM AND HEAVY DUTY TRUCKS ////////////////////
Extracted 1 worksheets in 13.95 seconds
Applying strategy: migrate_datasets
Applying strategy: migrate_exchanges
Create fleet average vehicles...
Done!

/////////////////////////// ELECTRICITY ////////////////////////////
Update natural gas extraction datasets.
Update efficiency of solar PV.
Log of changes in photovoltaics efficiencies saved in C:\Users\Gibon\AppData\Roaming\Python\Python39\site-packages\premise\data/logs
Create biomass markets.
Empty old electricity datasets
Create high voltage markets.
Create medium voltage markets.
Create low voltage markets.
Log of deleted electricity markets saved in C:\Users\Gibon\AppData\Roaming\Python\Python39\site-packages\premise\data/logs
Log of created electricity markets saved in C:\Users\Gibon\AppData\Roaming\Python\Python39\site-packages\premise\data/logs
Done!
Adjust efficiency of power plants...
Log of changes in power plants efficiencies saved in C:\Users\Gibon\AppData\Roaming\Python\Python39\site-packages\premise\data/logs
Rescale inventories and emissions for Biomass IGCC
Rescale inventories and emissions for Gas OC
Rescale inventories and emissions for Biomass IGCC CCS
Rescale inventories and emissions for Coal CHP
Rescale inventories and emissions for Gas CC CCS
Rescale inventories and emissions for Coal IGCC
Rescale inventories and emissions for Coal PC CCS
Rescale inventories and emissions for Oil ST
Rescale inventories and emissions for Gas CHP
Rescale inventories and emissions for Coal PC
Rescale inventories and emissions for Coal IGCC CCS
Rescale inventories and emissions for Biomass CHP
Rescale inventories and emissions for Gas CC

///////////////////////////// CEMENT //////////////////////////////

Start integration of cement data...

Log of deleted cement datasets saved in C:\Users\Gibon\AppData\Roaming\Python\Python39\site-packages\premise\data/logs
Log of created cement datasets saved in C:\Users\Gibon\AppData\Roaming\Python\Python39\site-packages\premise\data/logs

Create new clinker production datasets and delete old datasets
Adjusting emissions of hot pollutants for clinker production datasets...

Create new clinker market datasets and delete old datasets
Adjust clinker-to-cement ratio in "unspecified cement" datasets

Create new cement market datasets

Create new cement production datasets and adjust electricity consumption
Done!

////////////////////////////// STEEL //////////////////////////////
Log of deleted steel datasets saved in C:\Users\Gibon\AppData\Roaming\Python\Python39\site-packages\premise\data\logs
Log of created steel datasets saved in C:\Users\Gibon\AppData\Roaming\Python\Python39\site-packages\premise\data\logs
Create steel markets for different regions
Create new steel production datasets and empty old datasets
Create pig iron production datasets
Done!

////////////////////////////// FUELS ///////////////////////////////
Generate region-specific direct air capture processes.
Generate region-specific hydrogen production pathways.
Generate region-specific hydrogen supply chains.
Generate region-specific biogas and syngas supply chains.
Generate region-specific synthetic fuel supply chains.
Generate region-specific biofuel supply chains.
Generate new fuel markets.
--> petrol, unleaded
--> petrol, low-sulfur
--> diesel, low-sulfur
--> diesel
--> natural gas
--> hydrogen
Warning: ('iron-nickel-chromium alloy production', 'RoW') has no fossil CO2 output flow.
Warning: ('acetylene production', 'RER') has no fossil CO2 output flow.
Warning: ('polylactide production, granulate', 'GLO') has no fossil CO2 output flow.
Warning: ('transport, pipeline, offshore, long distance, natural gas', 'DZ') has no fossil CO2 output flow.
Warning: ('sulfite pulp production, bleached', 'RER') has no fossil CO2 output flow.
Warning: ('gold-silver mine operation and beneficiation', 'CA-QC') has no fossil CO2 output flow.
Warning: ('polyester-complexed starch biopolymer production', 'RoW') has no fossil CO2 output flow.
Warning: ('sawing and planing, azobe, air dried', 'RER') has no fossil CO2 output flow.
Warning: ('methanol production', 'GLO') has no fossil CO2 output flow.
Warning: ('sulfite pulp production, bleached', 'RoW') has no fossil CO2 output flow.
Warning: ('natural gas production', 'US') has no fossil CO2 output flow.
Warning: ('natural gas pressure reduction from high to low pressure', 'RoW') has no fossil CO2 output flow.
Warning: ('natural gas production', 'US') has no fossil CO2 output flow.
Warning: ('gold-silver mine operation and beneficiation', 'CA-QC') has no fossil CO2 output flow.
Warning: ('sawing and planing, azobe, air dried', 'RoW') has no fossil CO2 output flow.
Warning: ('sawing and planing, azobe, air dried', 'RER') has no fossil CO2 output flow.
Warning: ('polyester-complexed starch biopolymer production', 'RER') has no fossil CO2 output flow.
Warning: ('ilmenite - magnetite mine operation', 'GLO') has no fossil CO2 output flow.
Warning: ('sawing and planing, azobe, air dried', 'RoW') has no fossil CO2 output flow.
Warning: ('iron-nickel-chromium alloy production', 'RER') has no fossil CO2 output flow.
Warning: ('transport, pipeline, onshore, long distance, natural gas', 'DZ') has no fossil CO2 output flow.
Warning: ('trichloromethane production', 'RoW') has no fossil CO2 output flow.
Warning: ('cast iron production', 'RER') has no fossil CO2 output flow.
Warning: ('gold-silver mine operation and beneficiation', 'CA-QC') has no fossil CO2 output flow.
Warning: ('ilmenite - magnetite mine operation', 'GLO') has no fossil CO2 output flow.
Warning: ('gold-silver mine operation and beneficiation', 'CA-QC') has no fossil CO2 output flow.
Warning: ('acetylene production', 'RoW') has no fossil CO2 output flow.
Warning: ('cast iron production', 'RoW') has no fossil CO2 output flow.
Warning: ('nickel mine operation and benefication to nickel concentrate, 16% Ni', 'CA-QC') has no fossil CO2 output flow.
Warning: ('trichloromethane production', 'RER') has no fossil CO2 output flow.
Warning: ('Farming and supply of wheat straw', 'RER') has no fossil CO2 output flow.
Warning: ('lead acid battery, for lorry', 'RER') has no fossil CO2 output flow.
Log of deleted fuel markets saved in C:\Users\Gibon\AppData\Roaming\Python\Python39\site-packages\premise\data\logs
Log of created fuel markets saved in C:\Users\Gibon\AppData\Roaming\Python\Python39\site-packages\premise\data\logs
Done!
```

In [15]:

```
ndb.write_db_to_brightway()
```

```
Write new database(s) to Brightway2.
Prepare database 1.
- check for duplicates...
- check for values format...
- relinking exchanges...
Done!
Database ecoinvent_remind_SSP2-Base_2020 already existing: it will be overwritten.
Vacuuming database
```

```
Writing activities to SQLite3 database:
0% [##############################] 100% | ETA: 00:00:00
Total time elapsed: 00:02:58
```

```
Title: Writing activities to SQLite3 database:
  Started: 12/07/2022 15:50:21
  Finished: 12/07/2022 15:53:19
  Total time elapsed: 00:02:58
  CPU %: 74.40
  Memory %: 3.23
Created database: ecoinvent_remind_SSP2-Base_2020
```

## Import nuclear inventory¶

If you need to reimport the inventories from the original files, the code and data to write the brightway-format database (in Excel) are available on the repository. If you do this, you'll need to add two cells on A1 of the first sheet with these values: ["Database", "UNEP\_IRP"].

**Don't run this section if you have already imported the inventories**

In [25]:

```
wb=openpyxl.load_workbook('data/THEMIS_inventories_2010_regionalized.xlsx')
```

```
C:\Users\Gibon\AppData\Roaming\Python\Python39\site-packages\openpyxl\worksheet\_reader.py:312: UserWarning: Data Validation extension is not supported and will be removed
  warn(msg)
```

In [18]:

```
bw.databases
```

Out[18]:

```
Databases dictionary with 3 object(s):
	biosphere3
	ecoinvent 3.8 cutoff
	ecoinvent_remind_SSP2-Base_2020
```

In [27]:

```
database_writer('data/THEMIS_inventories_2010_regionalized.xlsx', year=2020)
```

```
Extracted 24 worksheets in 1.25 seconds
Applying strategy: csv_restore_tuples
Applying strategy: csv_restore_booleans
Applying strategy: csv_numerize
Applying strategy: csv_drop_unknown
Applying strategy: csv_add_missing_exchanges_section
Applying strategy: normalize_units
Applying strategy: normalize_biosphere_categories
Applying strategy: normalize_biosphere_names
Applying strategy: strip_biosphere_exc_locations
Applying strategy: set_code_by_activity_hash
Applying strategy: link_iterable_by_fields
Applying strategy: assign_only_product_as_production
Applying strategy: link_technosphere_by_activity_hash
Applying strategy: drop_falsey_uncertainty_fields_but_keep_zeros
Applying strategy: convert_uncertainty_types_to_integers
Applying strategy: convert_activity_parameters_to_list
Applied 16 strategies in 25.94 seconds
Applying strategy: migrate_datasets
Applying strategy: migrate_exchanges
Applying strategy: migrate_datasets
Applying strategy: migrate_exchanges
Applying strategy: migrate_datasets
Applying strategy: migrate_exchanges
Applying strategy: migrate_datasets
Applying strategy: migrate_exchanges
Applying strategy: migrate_datasets
Applying strategy: migrate_exchanges
Applying strategy: migrate_datasets
Applying strategy: migrate_exchanges
Applying strategy: migrate_datasets
Applying strategy: migrate_exchanges
Applying strategy: migrate_datasets
Applying strategy: migrate_exchanges
Applying strategy: link_iterable_by_fields
Applying strategy: link_iterable_by_fields
358 datasets
3096 exchanges
1083 unlinked exchanges
  Type biosphere: 95 unique unlinked exchanges
  Type technosphere: 77 unique unlinked exchanges
```

```
D:\Nextcloud\unece\nuclear-lca\utils\database_writer.py:79: SettingWithCopyWarning: 
A value is trying to be set on a copy of a slice from a DataFrame.
Try using .loc[row_indexer,col_indexer] = value instead

See the caveats in the documentation: https://pandas.pydata.org/pandas-docs/stable/user_guide/indexing.html#returning-a-view-versus-a-copy
  techno_map[['ei37product',
D:\Nextcloud\unece\nuclear-lca\utils\database_writer.py:79: SettingWithCopyWarning: 
A value is trying to be set on a copy of a slice from a DataFrame.
Try using .loc[row_indexer,col_indexer] = value instead

See the caveats in the documentation: https://pandas.pydata.org/pandas-docs/stable/user_guide/indexing.html#returning-a-view-versus-a-copy
  techno_map[['ei37product',
D:\Nextcloud\unece\nuclear-lca\utils\database_writer.py:79: SettingWithCopyWarning: 
A value is trying to be set on a copy of a slice from a DataFrame.
Try using .loc[row_indexer,col_indexer] = value instead

See the caveats in the documentation: https://pandas.pydata.org/pandas-docs/stable/user_guide/indexing.html#returning-a-view-versus-a-copy
  techno_map[['ei37product',
```

```
Applying strategy: migrate_datasets
Applying strategy: migrate_exchanges
Applying strategy: migrate_datasets
Applying strategy: migrate_exchanges
Applying strategy: migrate_datasets
Applying strategy: migrate_exchanges
Applying strategy: migrate_datasets
Applying strategy: migrate_exchanges
Applying strategy: migrate_datasets
Applying strategy: migrate_exchanges
Applying strategy: link_iterable_by_fields
Applying strategy: link_iterable_by_fields
Applying strategy: link_iterable_by_fields
358 datasets
3096 exchanges
6 unlinked exchanges
  Type technosphere: 1 unique unlinked exchanges
[{'name': 'port maintenance and land use', 'amount': 2.54e-12, 'location': 'RER', 'unit': 'unit', 'categories': ('Service activities incidental to water transportation',), 'database': 'ecoinvent 3.7 cutoff', 'code': 'dc05747b-a67e-43e8-89a1-b79a8f9cdc8a', 'type': 'technosphere', 'reference product': 'port maintenance and land use', 'ecoinvent 2.2 name': 'operation, maintenance, port', 'ecoinvent 2.2 unit': 'unit', 'UNEP IRP name': 'operation, maintenance, port/ RER/ unit'}]
Applying strategy: drop_unlinked
Applied 1 strategies in 0.00 seconds
```

```
Writing activities to SQLite3 database:
0% [##############################] 100% | ETA: 00:00:00
Total time elapsed: 00:00:00
```

```
Title: Writing activities to SQLite3 database:
  Started: 12/07/2022 16:32:52
  Finished: 12/07/2022 16:32:52
  Total time elapsed: 00:00:00
  CPU %: 80.10
  Memory %: 3.58
Created database: UNEP_IRP_EUR
```

# Test¶

In [29]:

```
unep_db=bw.Database('UNEP_IRP_EUR')
```

In [34]:

```
nuclear = unep_db.get('unep_irp112001')
```

In [50]:

```
m=('EF v3.0', 'climate change', 'global warming potential (GWP100)')
```

In [51]:

```
LCA = nuclear.lca(method=m)
```

In [52]:

```
LCA.lci()
```

In [53]:

```
LCA.lcia()
```

In [54]:

```
LCA.score
```

Out[54]:

```
0.006420694973024735
```
