# Supplementary material for: Parametric Life Cycle Assessment of Nuclear Power for Simplified Models
Source: Environ Sci Technol. 2023 Sep 12;57(38):14194–205. doi: 10.1021/acs.est.3c03190 (PMC10537461; doi:10.1021/acs.est.3c03190)
Supplement: Supplementary file 4 — es3c03190_si_004.zip [file es3c03190_si_004.zip › 2_parameterization.html]

2\_parameterization


# Parameterizing the nuclear power inventory¶

In this notebook we will use `lca_algebraic` to pass input parameters as variables instead of values in the nuclear power inventory from UNECE (2022). This should allow to identify the main parameters affecting the life cycle results as well as providing a simplified LCA for the main impact indicators.

Each phase of the nuclear (once-through) cycle is parameterized:

- mining (product: ore, $U\_3O\_8$): ore grade, energy mix (diesel/electricity), electricity source, mining mix (open pit, underground, ISL),
- milling (product: yellowcake, $U\_3O\_8$): electricity source, tailings' $^{222}Rn$ emissions,
- conversion (product: gas, $UF\_6$): heat input,
- enrichment (product: gas, $UF\_6$): enrichment technology, energy input, enrichment rate,
- fuel fabrication (product: fuel, $UO\_2$): electricity input,
- operation (product: electricity): plant lifetime, plant availability,
- backend (interim storage and final repository).

The main output is a set of distributions for each impact indicator. As well as a one-at-a-time sensitivity analysis per parameter and impact indicator.

Version 1.0.3 of `lca\_algebraic` is used in the present work (with small modifications, namely on exchange-matching for updating inventories).

Credits to Raphaël Jolivet and colleagues for the development of the wonderful `lca_algebraic` package, available here.

Authors: thomas.gibon@list.lu, alvaro.hahn@list.lu

## Initialization¶

In [6]:

```
# Let's get comfortable first
from IPython.core.display import display, HTML
display(HTML("<style>.container { width:80% !important; }</style>"))
```

```
C:\Users\Gibon\AppData\Local\Temp\ipykernel_26116\1931309167.py:2: DeprecationWarning: Importing display from IPython.core.display is deprecated since IPython 7.14, please import from IPython display
  from IPython.core.display import display, HTML
```

In [7]:

```
# Usual modules
import pandas as pd
import time
import matplotlib.pyplot as plt
import numpy as np
import brightway2 as bw

# Custom utils defined for the parameterization
from lca_algebraic import *
from lca_algebraic.stats import * 
from lca_algebraic.helpers import _amountToFormula
import lca_algebraic
from matplotlib.patches import Rectangle
from sympy import init_printing, simplify, log, exp, N, Piecewise, sympify
from sympy.plotting import plot, plot3d
from sympy.solvers import solve
from scipy.stats import lognorm, linregress
from sigfig import round

from utils import utils

from IPython.display import Image

init_printing()
```

In [8]:

```
# This avoids a crash later in the code...
import os
os.environ["KMP_DUPLICATE_LIB_OK"]="TRUE"
```

In [9]:

```
# This forces svg to save text as text
plt.rcParams['svg.fonttype'] = 'none'
```

In [10]:

```
# sns.set_theme()
sns.set(font_scale=.8)
# sns.set_style('whitegrid')
```

In [11]:

```
# Initialisation of the project
# Parameterized inventory from WNA data collection
initProject('nuclear_param')

# Import ecoinvent DB (if not already done)
# Update the name and path to the location of the ecoinvent database
if 'ecoinvent 3.8 cutoff' not in bw.databases:
    importDb("ecoinvent 3.8 cutoff", 'D:/Nextcloud/ecoinvent/ecoinvent 3.8_cutoff_ecoSpold02/datasets')

# We use a separate DB for defining our foreground model / activities
# Choose any name

# Foreground database with parameters
USER_DB = 'Nuclear_DB'

# Declare ecoinvent as the background database
BG_DB = 'ecoinvent 3.8 cutoff'
```

```
Biosphere database already present!!! No setup is needed
```

In [12]:

```
# Recommendations from lca_algebraic

# This is better to cleanup the whole foreground model each time, and redefine it in the notebook
# instead of relying on a state or previous run.
# Any persistent state is prone to errors.
resetDb(USER_DB)

# Parameters are stored at project level : 
# Reset them also
# You may remove this line if you import a project and parameters from an external source (see loadParam(..))
resetParams()
```

```
Db Nuclear_DB was here. Reseting it
```

## Define input parameters¶

This section introduces the various parameters used in the full model. It is rather long and detailed, but a recap is provided at the end of this section.

### Mining¶

In the mining phase, the following parameters are included:

- mining technique mix (open cast, underground, ISL),
- ore grade,
- tailings radon-222 emissions,
- mining energy mix (diesel or electricity).

#### Ore grade¶

Ore grade is one of the most sensitive parameters. We use a global ore distribution following a log-normal law, with a mean grade of known deposits of 1544 ppmU, and a standard deviation of 1299 ppm, available in Monnet et al. (2016). Other values are reported in the same publication, they can be tested in this model.

Source: https://hal-cea.archives-ouvertes.fr/cea-01350727/document

(Other source: https://www.researchgate.net/publication/330994502\_Global\_Grade-and-Tonnage\_Modeling\_of\_Uranium\_deposits)

Realistically, at least in economically-viable terms, ore grade ranges from 0.01% to 20% (see below).

Source: Norgate et al. (2014)
https://doi.org/10.1016/j.jclepro.2013.11.034

#### Techniques¶

Mining techniques are continuously evolving, as shown by a changing global mining mix (from the OECD-NEA Red Book).

In [13]:

```
# Input parameters

# We will cover 93.5% of mining
# Therefore we will normalize
# This is conservative (by-product would have a lower impact becasue of allocation)

mining_shares = {'open_pit':.161/.935,
                'underground':.200/.935,
                'ISL':0.574/.935}

mining_energy_shares = {'diesel':0.3,
                        'electricity':0.7}

# Setting the share of in-site leaching as a parameter
share_ISL = newFloatParam(
    'share_ISL', 
    default=0.574, min=0, max=1,
    description="Share of ISL, the rest is rescaled in proportion",
    unit='dimensionless',
    label='Share of ISL, the rest is rescaled in proportion',
    dbname=USER_DB)

share_open_pit = (1-share_ISL) * mining_shares['open_pit'] / (mining_shares['open_pit'] + mining_shares['underground'])
share_underground = 1-share_ISL-share_open_pit

print([f'{k}: {v*100:.2f}%' for k,v in mining_shares.items()])

# Ore grade (TRIANGULAR distribution)
ore_grade = newFloatParam(
    'ore_grade', 
    default=0.0015,
    min=    0.00001,
    max=    0.020,
    unit='dimensionless',
    distrib=DistributionType.TRIANGLE,
    description="Uranium ore (U3O8) grade as commonly reported, from 0.02% to 20%, however we use 10 ppm – 20000 ppm (2%) as a conservative range",
    label="Uranium ore grade",
    dbname=USER_DB)

# We will be using the values from Table 4 above
# These are the official values from the IAEA's UDEPO database
og_avg = 0.001544
og_std = 0.001299

# Ore grade (LOGNORMAL distribution)
ore_grade = newFloatParam(
    'ore_grade', 
    default=og_avg,
    std=    np.log(1+(og_std/og_avg)**2)**.5, # careful, std for a lognormal distribution is actually the lognormal of the std
    min=    0.00001,
    max=    0.020,
    unit='dimensionless',
    distrib=DistributionType.LOGNORMAL,
    description="Uranium ore (U3O8) grade as commonly reported, from 0.02% to 20%, however we use 10 ppm – 20000 ppm (2%) as a conservative range",
    label="Uranium ore grade",
    dbname=USER_DB)

ore_grade.mean = np.log(og_avg/(1+(og_std/og_avg)**2)**.5)
ore_grade.sigma = og_std

# Recovery rate, as defined in Storm van Leeuwen (equation reused in Parker 2016)

a_rr = 0.98
b_rr = 0.0723
recovery_rate = Piecewise ( (a_rr - b_rr * log(ore_grade*100,10) * log(ore_grade*100,10), ore_grade < 0.01),
                            (a_rr, True))

# Total energy for extraction

a_op = 274
b_op = -0.482

a_ug = 317
b_ug = -0.176

a_is = 220
b_is = -0.0485

extraction_energy_open_pit = a_op * exp(b_op * ore_grade) / recovery_rate
extraction_energy_underground = a_ug * exp(b_ug * ore_grade) / recovery_rate
extraction_energy_ISL = a_is * exp(b_is * ore_grade) / recovery_rate

# Over how long should we integrate remaining radiations?
integration_time_Rn222 = newFloatParam(
    'integration_time_Rn222', 
    default=80000, min=100, max=80000, # In years, integration time
    distrib=DistributionType.TRIANGLE,
    description="Integration time for ionising radiation of milling tailings",
    unit='years',
    label='Integration time for radiation from milling tailings',
    dbname=USER_DB)

# Emissions of radon-222 from tailings
tailings_Rn222 = newFloatParam(
    'tailings_Rn222', 
    default=0.01951, min=0.01, max=1,
    distrib=DistributionType.TRIANGLE, # Distribution type, linear by default
    description="Rn222 from tailings, in Bq/s",
    label="Rn222 from tailings, in Bq/s",
    unit='Bq/s',
    dbname=USER_DB)

mining_electricity_switch = newEnumParam(
    'mining_electricity_switch', 
    label='Mining electricity, grid or diesel',
    values=['dieselgenerator',
            'grid'], # You can provide a statistical weight for each value, by using a dict
    default='dieselgenerator', 
    description="Choice of electricity source for mining",
    dbname=USER_DB)

l_230Th = 75380 # half-life of Thorium-230

# Calculated parameters

tailings_Rn222_kBq_per_m3 = 4 * tailings_Rn222 * 3600 * 8766 / 1000 \
    * l_230Th / N(log(2)) * (1 - exp(-integration_time_Rn222/(l_230Th / N(log(2)))))
```

```
['open_pit: 17.22%', 'underground: 21.39%', 'ISL: 61.39%']
```

```
Warning : LogNormal does not support min/max boundaries for parameter :  ore_grade
[ParamRegistry] Param ore_grade was already defined in 'Nuclear_DB' : overriding.
```

We create a dummy process reproducing exactly the ore grade as kg of CO2 emitted, to ensure the lognormal distribution is read correctly.
There are conflicts between how lognormal is characterized in brightway and lca\_algebraic, so just a healthy check.

In [14]:

```
CO2 = findBioAct('Carbon dioxide, fossil', categories=['air'])
dummy = newActivity(USER_DB, # We define foreground activities in our own DB
    'testing ore grade', # Name of the activity
    'kg', # Unit
    exchanges= { # We define exhanges as a dictionarry of 'activity : amount'
        CO2: ore_grade, # Amount can be a fixed value 
    })

# List of impacts to consider
impacts_all = [m for m in bw.methods if 'EF v3.0'== m[0] if len(m)==3]

# Select 9 categories
impacts = [impacts_all[i] for i in [1, 5, 10, 13, 17, 21, 22, 23, 27]]
```

In [15]:

```
multiLCAAlgebric(dummy, [impacts[0]])
```

```
Required param 'ore_grade' was missing, replacing by default value : 0.001544
```

Out[15]:

|  | climate change - global warming potential (GWP100)[kg CO2-Eq] |
| --- | --- |
| testing ore grade | 0.001544 |

In [16]:

```
# Looks good, let's have a look at the full distribution
distrib(
    dummy,
    [impacts[0]],
    # Optional layout parameters
    percentiles=[5, 95])
```

```
Generating samples ...
```

```
C:\Users\Gibon\.conda\envs\unece\lib\site-packages\SALib\sample\saltelli.py:94: UserWarning: 
        Convergence properties of the Sobol' sequence is only valid if
        `N` (10000) is equal to `2^n`.
        
  warnings.warn(msg)
```

```
Transforming samples ...
```

Out[16]:

|  | climate change - global warming potential (GWP100) [kg CO2-Eq / kWh] |
| --- | --- |
| mean | 0.001544 |
| median | 0.001181 |
| p | [0.0003548555832486859, 0.003935395487803796] |
| std | 0.001301 |
| var | 0.842366 |

In [17]:

```
# What we have modelled so far
utils._plot_params([ore_grade,integration_time_Rn222, tailings_Rn222], figsize=(8,4), size=1000)
```

The choice of integration time for the $^{222}Rn$ emissions is key for the "ionising radiation" indicator:

- Using a 100-year horizon (as done by UNSCEAR) doesn't leave much time for Rn to be emitted,
- Using a 80000-year horizon (as done by ecoinvent 2.0) make the Rn emissions a primary stressor.

The reason why ecoinvent integrates the radiation of milling tailings over 80000 years is that this value is of the order of magnitude of the half-life of $^{230}Th$ (72800 years). As $^{222}Rn$ is a (grand)daughter isotope of $^{230}Th$, this value is kept to ensure most decaying radon is considered in the timeframe. In reality, since a half-life is not a lifetime, radon is still emitted long after 80000 years.

The reason why UNSCEAR uses a time horizon of 100 years is given below:

We leave this integration time as a parameter to observe its actual effect on the LCA results.

In [19]:

```
## Influence of integration time on tailings emissions (in kBq)
plot(tailings_Rn222_kBq_per_m3.subs({tailings_Rn222:tailings_Rn222.default}),
    (integration_time_Rn222,100,1000000),
    adaptive=True,
     xscale='log',
    size=[4,2])
```

Out[19]:

```
<sympy.plotting.plot.Plot at 0x210206b00d0>
```

In [20]:

```
## Recovery rate
solve(recovery_rate)
```

Out[20]:

$\displaystyle \left[ 2.08131693873235 \cdot 10^{-6}\right]$

The equation found in Storm van Leeuwen falls to zero for an ore concentration of about 2 ppm, and is only valid until 1% (10000 ppm). It is assumed equal to 98% after that. For comparison, the abundance of uranium in the Earth crust is about 3 ppm.

In [21]:

```
# Actual recovery rate for the modal value of 0.15%
N(recovery_rate.subs({ore_grade:0.001544}),2)
```

Out[21]:

$\displaystyle 0.93$

In [22]:

```
## Energy use for extraction

# At 0.01% (100 ppm) 
[N(expr.subs({ore_grade:100e-6}), 3) for expr in (extraction_energy_open_pit,
                                                  extraction_energy_underground,
                                                  extraction_energy_ISL)]
```

Out[22]:

$\displaystyle \left[ 397.0, \ 459.0, \ 319.0\right]$

In [23]:

```
# At 0.001% (10 ppm), energy consumption would be as high as
[N(expr.subs({ore_grade:10e-6}), 3) for expr in (extraction_energy_open_pit,
                                                 extraction_energy_underground,
                                                 extraction_energy_ISL)]
```

Out[23]:

$\displaystyle \left[ 832.0, \ 963.0, \ 668.0\right]$

At 10 ppm we approach 1 GJ per kg extracted for underground mining. This is consistent with literature, as seen in Table 3.3 below – even though total energy requirements are not degressive with increasing ore grade.

### Milling¶

An exhaustive source for milling is a 2006 report by Manfred Lenzen. Milling is treated as a separate process in this model, although it is sometimes conflated with mining, as both processes often occur at the same location. Additionally, ISL extraction does not require milling, the modelled ISL process outputs yellowcake directly.

In [24]:

```
# We use a switch function as a handle for the choice of energy source
milling_electricity_switch = newEnumParam(
    'milling_electricity_switch',
    label='Milling electricity, grid or diesel',
    values=['dieselgenerator',
            'grid'],
    default='dieselgenerator', 
    description="Choice of electricity source for milling",
    dbname=USER_DB)
```

In [25]:

```
# Lenzen (2006) energy data
milling_data = pd.DataFrame([[25.8,54.3],
                             [215.0,126.2]],
                            index=['Electricity','Thermal energy'],
                            columns=['Milling','Mining'])

milling_data/ore_grade.default/1000 # in kWh/kg U in ore
```

Out[25]:

|  | Milling | Mining |
| --- | --- | --- |
| Electricity | 16.709845 | 35.168394 |
| Thermal energy | 139.248705 | 81.735751 |

...in kWh per kg U in ore, according to Lenzen (2006). It is to be noted that the milling electricity input is assumed to be about 22.5 kWh/kg U in Parker et al. (2016).
All values later are in MJ and original values are per t ore, milling energy inputs are ore-grade dependent.

In [26]:

```
# Get the milling requirements (only for open pit and underground)
milling_elec_default=milling_data.loc['Electricity','Milling']/ore_grade/1000
milling_heat_default=milling_data.loc['Thermal energy','Milling']/ore_grade/1000*3.6
```

### Conversion¶

The conversion phase uses yellowcake as an input and outputs uranium hexafluoride, to be enriched in the next stage. At this point, neither this process nor the next steps are dependent on ore grade any more. Apart from the uranium product, chemicals, and infrastructure, conversion inputs include heat and electricity.

In [27]:

```
# Conversion phase, electricity consumption
conversion_elec = newFloatParam(
    'conversion_elec', 
    default=11.8, min=10.3, max=16.9,
    distrib=DistributionType.TRIANGLE,
    label='Conversion electricity input',
    unit='kWh/kg U in UF6',
    description="Electricity required to convert 1 kg of UF6",
    dbname=USER_DB)

# Conversion phase, heat consumption
conversion_heat = newFloatParam(
    'conversion_heat', 
    default=26, min=26, max=665,
    distrib=DistributionType.TRIANGLE,
    label='Conversion heat input',
    unit='kWh/kg U in UF6',
    description="Heat required to convert 1 kg of UF6",
    dbname=USER_DB)

utils._plot_params([conversion_elec,
              conversion_heat],
            figsize=(4,2))
```

### Enrichment¶

The enrichment process has historically consisted in technology options: gaseous diffusion and centrifugation. According to the WNA, as of 2015, all uranium is enriched via centrifugation, as shown below. The option for diffusion is included for comparability with older studies. Laser enrichment and the recycling of weapon-grade highly enriched uranium are also considered in the near future, but not modelled here.

Enrichment performance is commonly calculated as separative work units, or SWU, a quantity of work used to separate enriched uranium from tails assays, starting from a feed of 0.71% $^{235}U$, the natural occurrence of uranium's fissile isotope. Mass balance equations can be found in Glaser (2008), they are used here to parameterize the enrichment life cycle inventory.

In [28]:

```
# Input parameters

# Rate of enrichment, calculated global average is 4.2%
rate_enrichment = newFloatParam(
    'rate_enrichment', 
    default=0.0415, min=0.03, max=0.05,
    distrib=DistributionType.TRIANGLE,
    description="Uranium enrichment rate",
    label="Uranium enrichment rate",
    dbname=USER_DB)

# Rate of feed, natural geological availability of 235U isotope
rate_feed = newFloatParam(
    'rate_feed', 
    default=0.0071,
    description="Uranium feed rate (natural rate)",
    label="Uranium feed rate",
    dbname=USER_DB)

# Rate of tailings, usually .25%
rate_tailings = newFloatParam(
    'rate_tailings', 
    default=0.0022,
    description="Uranium enrichment tailings rate",
    label="Uranium enrichment tailings rate",
    dbname=USER_DB)

# Electricity consumption of centrifugation, per SWU
enrichment_centr_elec = newFloatParam(
    'enrichment_centr_elec', 
    default=50, min=40, max=100,
    unit='kWh/SWU',
    distrib=DistributionType.TRIANGLE,
    description="Electricity required per SWU of enriched U, centrifuge",
    label="Enrichment electricity, centrifuge",
    dbname=USER_DB)

# Electricity consumption of diffusion, per SWU
enrichment_diff_elec = newFloatParam(
    'enrichment_diff_elec', 
    default=2500, min=2400, max=3000,
    unit='kWh/SWU',
    distrib=DistributionType.TRIANGLE,
    description="Electricity required per SWU of enriched U, diffusion",
    label="Enrichment electricity, diffusion",
    dbname=USER_DB)

# Switch to choose enrichment technique
enrichment_mix_switch = newEnumParam(
    'enrichment_mix_switch',
    label='Enrichment technology',
    values={'centrifugation':0.8,
            'diffusion':0.2},
    default='centrifugation',
    dbname=USER_DB)

# Bins are not properly built, so we do it here 
enrichment_mix_switch._bins = [0]
for i in range(len(enrichment_mix_switch.values)) :
    enumvalue = enrichment_mix_switch.values[i]
    enrichment_mix_switch._bins.append(enrichment_mix_switch._bins[i] + enrichment_mix_switch.weights[enumvalue])

enrichment_centr_share = newFloatParam(
    'enrichment_centr_share',
    label='Share of centrifugation in enrichment',
    default=1,
    min=0,
    max=1,
    distrib=DistributionType.TRIANGLE,
    dbname=USER_DB)
    
# Calculated parameters, see Glaser (2008) for the detailed equation derivation

# The cut is the amount of product (in kg) you can get per kg of feed
cut = (rate_feed - rate_tailings) / (rate_enrichment - rate_tailings)

# How much separative work is required per kg feed
SWU_per_kg_feed = cut       * (2 * rate_enrichment - 1) * log(rate_enrichment/(1-rate_enrichment)) + \
                  (1 - cut) * (2 * rate_tailings - 1)   * log(rate_tailings/(1-rate_tailings))     - \
                              (2 * rate_feed - 1)       * log(rate_feed/(1-rate_feed))

# ...and per kg fuel
SWU_per_kg_fuel = simplify(SWU_per_kg_feed / cut)
```

```
No 'min/max' provided, param rate_feed marked as FIXED
No 'min/max' provided, param rate_tailings marked as FIXED
```

In [29]:

```
SWU_per_kg_fuel.subs({rate_feed:rate_feed.default,
                      rate_tailings:rate_tailings.default})
```

Out[29]:

$\displaystyle 248.938919246123 rate\_{enrichment} + 1.0 \cdot \left(2 rate\_{enrichment} - 1\right) \log{\left(- \frac{rate\_{enrichment}}{rate\_{enrichment} - 1} \right)} - 6.63784589722586$

As expected, the amount of work (energy input) required to enrich fuel is increasingly higher as the enrichment rate increases. We plot the relationship between SWU and enrichment rate in the next cell.

In [30]:

```
f = rate_feed.default
t = rate_tailings.default
e = rate_enrichment.default

SWU_enrich = SWU_per_kg_fuel.subs({rate_feed:f,
                                   rate_tailings:t})
f = lambdify(rate_enrichment, SWU_enrich, 'numpy')

ee = np.linspace(0.01,0.06,1000)
fig, ax = plt.subplots(figsize=(6,4))
ax.plot(ee,f(ee))
ax.set_xlabel('Enrichment rate')
ax.set_ylabel('SWU per kg of enriched product')
ax.set_title('Separative work vs. enrichment rate (from 1% to 6%)')

ax.plot((e,e),(0,f(e)), c='k', linewidth=0.5)
ax.plot((0,e),(f(e),f(e)), c='k', linewidth=0.5)

ax.set_ylim((0,10))
ax.set_xlim((0.01,0.06))

ax.text(1.1*0.01,
        1.05*f(e),
        f'default value: {f(e):.2f} SWU for {e*100:.2f}% enrichment',
        ha='left')

plt.tight_layout()
```

### Fuel fabrication¶

The main input of fuel fabrication is energy. In terms of materials, the "fuel" consists in 60% zirconium, modelled here as chromium (the justification for choosing this proxy can be found in the "Fuel fabrication" ecoinvent report). The amount of materials (namely metals) used to make the pellets and rods is relatively significant in relation to the weight of the uranium fuel itself so it should be parameterized too. However the literature does not identify these inputs to be influential for the overall environmental profile. Hence we model energy inputs only.

In [31]:

```
# Input parameters
fuel_fab_elec = newFloatParam(
    'fuel_fab_elec', 
    default=36, min=36, max=50,
    distrib=DistributionType.TRIANGLE,
    description="Electricity required per kg of fuel, at fuel fabrication",
    label="Fuel fabrication electricity",
    unit='kWh/kg fuel',
    dbname=USER_DB)
```

### Power plant construction¶

The construction of a nuclear power plant involves large amounts of bulk materials (steel, concrete, copper, aluminium...). Bills of materials vary widely in the literature for each material input, which could all be parameterized. However, since this could lead to a high number of parameters, we choose to define a "construction intensity" variable instead, triangularly distributed in the range [0.5 - 2.0] and multiplied by the *median* amount of each material input (median from the literature survey).

In [32]:

```
# Input parameters
lifetime = newFloatParam(
    'lifetime', 
    label='Lifetime of plant and on-site equipment',
    unit='year',
    default=60, min=30, max=80,
    distrib=DistributionType.TRIANGLE,
    description="Plant lifetime in years",
    dbname=USER_DB)

capacity = newFloatParam(
    'capacity', 
    label='Nameplate capacity',
    unit='MWe',
    default=1000,
    distrib=DistributionType.FIXED,
    description="Capacity in MW",
    dbname=USER_DB)

construction_intensity = newFloatParam(
    'construction_intensity',
    label='Intensity of construction inputs\ncompared with default values',
    unit='dimensionless',
    distrib=DistributionType.TRIANGLE,
    default=1,
    min=0.5,
    max=2.0,
    description='Intensity of construction inputs\ncompared with default values',
    dbname=USER_DB)
```

### Electricity production¶

Somehow overlapping with the previous process (since we use the plant's lifetime and capacity to calculate output), this step includes efficiency, availability, and a model for the burnup rate.

In [33]:

```
efficiency = newFloatParam(
    'efficiency', 
    default=.33,
    min=.30,
    max=.34,
    unit='dimensionless',
    distrib=DistributionType.LINEAR,
    description="Overall thermal efficiency of electricity generation",
    label="Efficiency of electricity generation",
    dbname=USER_DB)

availability = newFloatParam(
    'availability', 
    default=.90,
    min=.65,
    max=1.,
    unit='dimensionless',
    distrib=DistributionType.TRIANGLE,
    description="Availability of the power plant, 1 - maintenance and unplanned interventions",
    label="Availability of power plant",
    dbname=USER_DB)

# Calculated parameters
# https://www.researchgate.net/publication/337982550_US_Commercial_Spent_Nuclear_Fuel_Assembly_Characteristics_1968-2013/figures?lo=1

burnup_rate = 43 + 10.9 * 100 * (rate_enrichment - 0.04)
burnup_rate.subs({rate_enrichment:e})


# Switch to choose water cooling (only water removed)
river_cooling = newBoolParam(
    'river_cooling',
    label='River cooling',
    default=1,
    dbname=USER_DB)
```

Burnup rate values for US reactors are available a US NRC technical report. Figure 10 and Table 2 provide all the enrichment rate-burnup rate combinations from the US nuclear fleet, for three time periods and two reactor designs (BWR and PWR). A first-order linear approximation yields the relationship between burnup rate $b$ (in GW-day/t) and enrichment rate $e$ (in t 235U/t U):

$$b(e) = b\_{min} + \frac{b\_{max} - b\_{min}}{e\_{max}- e\_{min}}(e - e\_{min})$$

with:
$b\_{min} = 35.7, b\_{max} = 54.3, e\_{min} = 3.96\%, e\_{max} = 4.80\%$.

Another source for the enrichment-burnup relationship is Burns et al. (2020) https://doi.org/10.1016/j.anucene.2020.107423

The relationship is sensibly different, with a reported slope of 10.9 MWd/kgU/% of enrichment.

We use this latter value for the rest of the notebook.

In [34]:

```
f = lambdify(rate_enrichment, burnup_rate, 'numpy')

ee = np.linspace(0.01,0.06,1000)
fig, ax = plt.subplots(figsize=(6,4))
ax.plot(ee,f(ee))
ax.set_xlabel('Enrichment rate')
ax.set_ylabel('Discharge burnup (GWd/t$_U$)')
ax.set_title('Burnup rate vs. enrichment rate (from 1% to 6%)')

# plot(burnup_rate, (rate_enrichment, *e_lims),
#      size=[4,2],
#     axis_center =(e_lims[0], burnup_rate.subs({rate_enrichment:e_lims[0]})))
ax.plot((e,e),(0,f(e)), c='k', linewidth=0.5)
ax.plot((0,e),(f(e),f(e)), c='k', linewidth=0.5)
ax.set_ylim((f(ee[0]),f(ee[-1])))
ax.set_xlim((0.01,0.06))
ax.text(1.10*0.01,
        1.05*f(e),
        f'default value: {f(e):.1f} GWd/t$_U$ for {e*100:.2f}% enr.',
        ha='left')
plt.tight_layout()
```

### List of all parameters¶

Recap of all parameters declared in the model.

In [35]:

```
list_parameters()
```

Out[35]:

| group | name | label | default | min | max | std | distrib | unit | db |
| --- | --- | --- | --- | --- | --- | --- | --- | --- | --- |
|  | availability | Availability of power plant | 0.9 | 0.65 | 1 |  | triangle | dimensionless | Nuclear\_DB |
|  | capacity | Nameplate capacity | 1000 |  |  |  | fixed | MWe | Nuclear\_DB |
|  | construction\_intensity | Intensity of construction inputs compared with default values | 1 | 0.5 | 2 |  | triangle | dimensionless | Nuclear\_DB |
|  | conversion\_elec | Conversion electricity input | 11.8 | 10.3 | 16.9 |  | triangle | kWh/kg U in UF6 | Nuclear\_DB |
|  | conversion\_heat | Conversion heat input | 26 | 26 | 665 |  | triangle | kWh/kg U in UF6 | Nuclear\_DB |
|  | efficiency | Efficiency of electricity generation | 0.33 | 0.3 | 0.34 |  | linear | dimensionless | Nuclear\_DB |
|  | enrichment\_centr\_elec | Enrichment electricity, centrifuge | 50 | 40 | 100 |  | triangle | kWh/SWU | Nuclear\_DB |
|  | enrichment\_centr\_share | Share of centrifugation in enrichment | 1 | 0 | 1 |  | triangle |  | Nuclear\_DB |
|  | enrichment\_diff\_elec | Enrichment electricity, diffusion | 2500 | 2400 | 3000 |  | triangle | kWh/SWU | Nuclear\_DB |
|  | enrichment\_mix\_switch | Enrichment technology | centrifugation |  |  |  |  |  | Nuclear\_DB |
|  | fuel\_fab\_elec | Fuel fabrication electricity | 36 | 36 | 50 |  | triangle | kWh/kg fuel | Nuclear\_DB |
|  | integration\_time\_Rn222 | Integration time for radiation from milling tailings | 80000 | 100 | 80000 |  | triangle | years | Nuclear\_DB |
|  | lifetime | Lifetime of plant and on-site equipment | 60 | 30 | 80 |  | triangle | year | Nuclear\_DB |
|  | milling\_electricity\_switch | Milling electricity, grid or diesel | dieselgenerator |  |  |  |  |  | Nuclear\_DB |
|  | mining\_electricity\_switch | Mining electricity, grid or diesel | dieselgenerator |  |  |  |  |  | Nuclear\_DB |
|  | ore\_grade | Uranium ore grade | 0.001544 | 1e-05 | 0.02 | 0.731586 | lognormal | dimensionless | Nuclear\_DB |
|  | rate\_enrichment | Uranium enrichment rate | 0.0415 | 0.03 | 0.05 |  | triangle |  | Nuclear\_DB |
|  | rate\_feed | Uranium feed rate | 0.0071 |  |  |  | fixed |  | Nuclear\_DB |
|  | rate\_tailings | Uranium enrichment tailings rate | 0.0022 |  |  |  | fixed |  | Nuclear\_DB |
|  | river\_cooling | River cooling | 1 |  |  |  |  |  | Nuclear\_DB |
|  | share\_ISL | Share of ISL, the rest is rescaled in proportion | 0.574 | 0 | 1 |  | linear | dimensionless | Nuclear\_DB |
|  | tailings\_Rn222 | Rn222 from tailings, in Bq/s | 0.01951 | 0.01 | 1 |  | triangle | Bq/s | Nuclear\_DB |

In [36]:

```
# And because a figure is worth 1000 words
utils._plot_params([p for p in params._param_registry().all() if p.distrib in ['linear', 'triangle', 'lognormal']], columns=5, size=2000, figsize=(16,9))
plt.savefig('output/parameters.svg')
```

## Transforming inventories¶

This section gathers all life cycle inventories from the imported database, and replaces fixed values by the distributions modelled above.

First we identify the inventories to modify.

In [37]:

```
# Mining and milling
tailings         = findActivity('Tailing, from uranium milling, WNA', loc='EUR', db_name='UNEP_IRP_EUR')
open_pit         = findActivity('Uranium mine operation, open cast, WNA', loc='GLO', db_name='UNEP_IRP_EUR')
underground      = findActivity('Uranium mine operation, underground, WNA', loc='GLO', db_name='UNEP_IRP_EUR')
ISL              = findActivity('Uranium mine operation, in-situ leaching, WNA', loc='GLO', db_name='UNEP_IRP_EUR')
diesel_machinery = findActivity('market for diesel, burned in building machine', loc='GLO', db_name='ecoinvent_remind_SSP2-Base_2020')
diesel_generator = findActivity('market for diesel, burned in diesel-electric generating set, 10MW', loc='GLO', db_name='ecoinvent_remind_SSP2-Base_2020')
elec_grid        = findActivity('Electricity, medium voltage, uranium milling mix', loc='GLO', db_name='UNEP_IRP_EUR')
milling          = findActivity('Market for milling, uranium, in yellowcake, WNA', loc='GLO', db_name='UNEP_IRP_EUR')
yellowcake       = findActivity('Market for uranium, in yellowcake, WNA', loc='GLO', db_name='UNEP_IRP_EUR')

# Conversion
conversion       = findActivity('Market for uranium hexafluoride, WNA', loc='GLO', db_name='UNEP_IRP_EUR')

# Enrichment
enrichment_centr = findActivity('Uranium production, centrifuge, WNA', loc='GLO', db_name='UNEP_IRP_EUR')
enrichment_diff  = findActivity('Uranium production, diffusion, WNA', loc='GLO', db_name='UNEP_IRP_EUR')
enrichment       = findActivity('Market for uranium, WNA, per separative work unit', loc='GLO', db_name='UNEP_IRP_EUR')

# Fuel fabrication
fuel_element     = findActivity('Market for nuclear fuel element, for PWR, WNA', loc='GLO', db_name='UNEP_IRP_EUR')
fuel_element_mix = findActivity('Fuel elements, WNA', loc='GLO', db_name='UNEP_IRP_EUR')


low_level_wt     = findActivity('market for low level radioactive waste, WNA', loc='EUR', db_name='UNEP_IRP_EUR')

# NPP construction
construction     = findActivity('Construction elements', loc='GLO', db_name='UNEP_IRP_EUR')

# Operation
chemicals        = findActivity('Chemicals, use phase', loc='GLO', db_name='UNEP_IRP_EUR')
elec_prod        = findActivity('electricity production, nuclear, PWR, THEMIS', loc='GLO', db_name='UNEP_IRP_EUR')

# Back-end
interim_storage  = findActivity('Interim storage of spent fuel, WNA', loc='GLO', db_name='UNEP_IRP_EUR')
low_voltage      = findActivity('market group for electricity, low voltage', loc='EUR', db_name='ecoinvent_remind_SSP2-Base_2020')
```

Then we make a copy, preserving the non-parameterized inventories.

In [38]:

```
open_pit_p = copyActivity(
    USER_DB,
    open_pit,
    'Uranium mine operation, open cast, parameterized')

underground_p = copyActivity(
    USER_DB,
    underground,
    'Uranium mine operation, underground, parameterized')

ISL_p = copyActivity(
    USER_DB,
    ISL,
    'Uranium mine operation, in-situ leaching, parameterized')

tailings_p = copyActivity(
    USER_DB, # The copy of a background activity is done in our own DB, so that we can safely update it                
    tailings, # Initial activity : won't be altered
    'Tailing, from uranium milling, parameterized') # New name

milling_p = copyActivity(
    USER_DB,
    milling,
    'Market for milling, uranium, in yellowcake, parameterized')

yellowcake_p = copyActivity(
    USER_DB,
    yellowcake,
    'Market for uranium, in yellowcake, parameterized')

conversion_p = copyActivity(
    USER_DB,
    conversion,
    'Market for uranium hexafluoride, parameterized')

enrichment_centr_p = copyActivity(
    USER_DB,
    enrichment_centr,
    'Uranium production, centrifuge, parameterized')

enrichment_diff_p = copyActivity(
    USER_DB,
    enrichment_diff,
    'Uranium production, diffusion, parameterized')

enrichment_p = newSwitchAct(
    dbname=USER_DB,
    name='Uranium production, enriched, parameterized',
    paramDef=enrichment_mix_switch,
    acts_dict={'centrifugation':enrichment_centr_p,
               'diffusion':enrichment_diff_p})

# enrichment_p = enrichment_centr_p

fuel_element_p = copyActivity(
    USER_DB, # The copy of a background activity is done in our own DB, so that we can safely update it                
    fuel_element, # Initial activity : won't be altered
    'Market for nuclear fuel element, for PWR, parameterized') # New name

fuel_element_mix_p = copyActivity(
    USER_DB, # The copy of a background activity is done in our own DB, so that we can safely update it                
    fuel_element_mix, # Initial activity : won't be altered
    'Fuel elements, parameterized') # New name

construction_p = copyActivity(
    USER_DB, # The copy of a background activity is done in our own DB, so that we can safely update it                
    construction, # Initial activity : won't be altered
    'Construction, parameterized') # New name

chemicals_p = copyActivity(
    USER_DB, # The copy of a background activity is done in our own DB, so that we can safely update it                
    chemicals, # Initial activity : won't be altered
    'Chemicals, use phase, parameterized') # New name

elec_prod_p = copyActivity(
    USER_DB, # The copy of a background activity is done in our own DB, so that we can safely update it                
    elec_prod, # Initial activity : won't be altered
    'electricity production, nuclear, PWR, parameterized') # New name
```

### Minor adjustments¶

Some exchanges have no name, but we can make sure they do.

In [39]:

```
for e in conversion.exchanges():
    if 'name' not in e.keys():
        print(f'Writing name for {e}')
        d,a = e['input']
        aa = bw.Database(d).get(a)
        e['name'] = aa['name']
        conversion.new_exchange(**e).save()
        e.delete()
```

In [40]:

```
# And some flows in fuel fabrication will cause problems because they are not unique
fuel_fab_elec_mix = pd.DataFrame([(e.input['location'],e['amount']) for e in fuel_element.exchanges() if 'medium voltage' in e.input['name']]).groupby(0).sum().to_dict()[1]
```

In [41]:

```
# Electricity mix for fuel fabrication, weighted by share of global production from each country/region
fuel_fab_elec_mix
```

Out[41]:

```
{'BR': 0.9426551453260016,
 'CN': 3.2992930086410053,
 'EUR': 9.450117831893166,
 'IN': 0.11311861743912019,
 'JPN': 3.7800471327572662,
 'OAS': 1.6496465043205026,
 'REF': 6.504320502749412,
 'US': 10.260801256873528}
```

In [42]:

```
fuel_fab_elec_inputs = pd.DataFrame([(e.input,e['amount']/50*fuel_fab_elec) for e in fuel_element.exchanges() if 'medium voltage' in e.input['name']]).groupby(0).sum().to_dict()[1]
```

In [43]:

```
# Same thing, parameterized
fuel_fab_elec_inputs
```

Out[43]:

```
{'market group for electricity, medium voltage' (kilowatt hour, IN, None): 0.0022623723487824*fuel_fab_elec,
 'market group for electricity, medium voltage' (kilowatt hour, OAS, None): 0.03299293008641*fuel_fab_elec,
 'market group for electricity, medium voltage' (kilowatt hour, CN, None): 0.0659858601728201*fuel_fab_elec,
 'market group for electricity, medium voltage' (kilowatt hour, REF, None): 0.130086410054988*fuel_fab_elec,
 'market group for electricity, medium voltage' (kilowatt hour, BR, None): 0.01885310290652*fuel_fab_elec,
 'market group for electricity, medium voltage' (kilowatt hour, US, None): 0.205216025137471*fuel_fab_elec,
 'market group for electricity, medium voltage' (kilowatt hour, JPN, None): 0.0756009426551453*fuel_fab_elec,
 'market group for electricity, medium voltage' (kilowatt hour, EUR, None): 0.189002356637863*fuel_fab_elec}
```

## Update parameterized inventories¶

Here we replace fixed values by parameters in the LCIs

In [44]:

```
# Update exchanges by their name 
mining_elec_mix = newSwitchAct(USER_DB, 
    'mining_elec_mix', # Name
    mining_electricity_switch, # Switch parameter
    { # Dictionary of enum values / activities
        'dieselgenerator' : diesel_generator, # in MJ
        'grid' : (elec_grid, 1/3.6)  # in MJ (convert from kWh)
    })

# No idea why diesel generator is saved as biosphere here
# so we have to change it
exc = mining_elec_mix.getExchange(name='diesel*')
exc['type']='technosphere'
exc.save()

# Mining techniques, 3 activities
open_pit_p.updateExchanges({
    # This is electricity
    'market for diesel, burned in diesel-electric generating set, 10MW*': dict(amount=mining_energy_shares['electricity'] * extraction_energy_open_pit,
                                                                              input=mining_elec_mix),
    # This is diesel used as fuel
    'market for diesel, burned in building machine*': mining_energy_shares['diesel'] * extraction_energy_open_pit
}
)

underground_p.updateExchanges({
    # This is electricity
    'market group for electricity, medium voltage*': dict(amount=mining_energy_shares['electricity'] * extraction_energy_underground,
                                                                              input=mining_elec_mix),
    # This SHOULD be diesel used as fuel
    'market for diesel, burned in diesel-electric generating set, 10MW*': dict(amount=mining_energy_shares['diesel'] * extraction_energy_underground,
                                                                              input=diesel_machinery),
}
)

ISL_p.updateExchanges({
    'market for diesel, burned in diesel-electric generating set, 10MW*': dict(amount=mining_energy_shares['diesel'] * extraction_energy_ISL,
                                                                              input=diesel_machinery),
    'market for electricity, medium voltage*': dict(amount=mining_energy_shares['electricity'] * extraction_energy_ISL,
                                                                              input=mining_elec_mix),
}
)

# Tailings production from milling
tailings_p.updateExchanges({
    
    # Update amount : the special symbol *old_amount* references the previous amount of this exchange
    'Radon-222*': tailings_Rn222_kBq_per_m3,
    
}) 

milling_elec_mix = newSwitchAct(USER_DB, 
    'milling_elec_mix', # Name
    milling_electricity_switch, # Sith parameter
    { # Dictionnary of enum values / activities
        'dieselgenerator' : diesel_generator, # in MJ
        'grid' : (elec_grid, 1/3.6)  # in MJ (convert from kWh)
    })

# No idea why diesel generator is saved as biosphere here
# so we have to change it
exc = milling_elec_mix.getExchange(name='diesel*')
exc['type']='technosphere'
exc.save()

# Milling can now be adapted
milling_p.updateExchanges({
    
    # Update amount : the special symbol *old_amount* references the previous amount of this exchange
    'Uranium mine operation, open cast, WNA*': dict(amount=share_open_pit/(share_open_pit + share_underground),
                                                   input=open_pit_p),
    
    'Uranium mine operation, underground, WNA*': dict(amount=share_underground/(share_open_pit + share_underground),
                                                     input=underground_p),
    
    'Electricity, medium voltage, uranium milling mix*': dict(amount=milling_elec_default,
                                                             input=milling_elec_mix),
    
    'market for diesel, burned in diesel-electric generating set, 10MW*': 0,
    'market for heat, district or industrial, other than natural gas*': milling_heat_default,
    'market for tailing, from uranium milling': tailings_p
    
}) 

# Yellowcake production is a mix of milling and ISL
yellowcake_p.updateExchanges({
    'Market for milling, uranium, in yellowcake, WNA':dict(amount=share_open_pit+share_underground,
                                                           input=milling_p),
    
    'Uranium mine operation, in-situ leaching, WNA': dict(amount=share_ISL,
                                                          input=ISL_p)
})

# Subsequent stages
conversion_p.updateExchanges({
    'Electricity, high voltage, uranium conversion mix':conversion_elec,
    'market for heat, district or industrial, natural gas':conversion_heat,
    'Market for uranium, in yellowcake, WNA':yellowcake_p
}
)

enrichment_centr_p.updateExchanges({
    'Electricity, high voltage, uranium enrichment mix':enrichment_centr_elec,
    'Market for uranium hexafluoride, WNA':dict(input=conversion_p,
                                                amount=1/SWU_per_kg_feed)
})

enrichment_diff_p.updateExchanges({
    'Electricity, high voltage, uranium enrichment mix':enrichment_diff_elec,
    'Market for uranium hexafluoride, WNA':dict(input=conversion_p,
                                                amount=1/SWU_per_kg_feed)
})

fuel_element_p.updateExchanges({
    'Market for uranium, WNA, per separative work unit#GLO': dict(input=enrichment_p,
                                                                  amount=SWU_per_kg_fuel),
    'market for low level radioactive waste':low_level_wt,
}) 

fuel_element_p.deleteExchanges('market group for electricity, medium voltage*', single=False)

fuel_element_p.addExchanges(fuel_fab_elec_inputs)

# No idea why electricity generator is saved as biosphere here
# so we have to change it
for exc in fuel_element_p.exchanges():
    if exc['name'] == 'market group for electricity, medium voltage':
        exc['type'] = 'technosphere'
        exc.save()

fuel_element_mix_p.updateExchanges({
    
    # Update amount : the special symbol *old_amount* references the previous amount of this exchange
    'Market for nuclear fuel element, for PWR, WNA': dict(input=fuel_element_p),
}) 

# As explained, to keep the model reasonably-sized
# we apply a single parameter to all construction inputs
construction_p.updateExchanges({
    'market group for concrete, normal':      old_amount * construction_intensity,
    'market for copper, cathode':       old_amount * construction_intensity,
    'reinforcing steel production':     old_amount *  construction_intensity,
    'steel production, low-alloyed, hot rolled':     old_amount * construction_intensity,
    'market for aluminium, cast alloy': old_amount * construction_intensity,
    'market group for electricity, low voltage':     old_amount * construction_intensity,
    'market for diesel, burned in building machine': old_amount * construction_intensity
})

water_to_air = 0.0023 #m3/kWh
water_input = 0.073769 #m3/kWh

# We will do this a bit later

# chemicals_p.updateExchanges({    
#     # Update amount : the special symbol *old_amount* references the previous amount of this exchange
#     'Water': river_cooling * water_to_air,
#     'Water': water_input - river_cooling * water_to_air,
#     'Water, cooling, unspecified natural origin': water_input
# }) 

elec_prod_p.updateExchanges({
    'Fuel elements, WNA':dict(amount=1/(burnup_rate * efficiency * 24 * 1000), input=fuel_element_mix_p),
    'Construction elements':dict(amount=1/(lifetime * availability * capacity * 8766 * 1000), input=construction_p),
    'Chemicals, use phase':chemicals_p
})

interim_storage.updateExchanges({
    'electricity production, nuclear, PWR, THEMIS':low_voltage,
})
```

In [45]:

```
for exch in chemicals_p.exchanges():
    
    if 'Water' in exch['name']:
        
        if 'air' in exch['categories']:
            attrs = {'amount':river_cooling * water_to_air}
            attrs.update(_amountToFormula(attrs['amount'], exch['amount']))
            exch.update(attrs)
            exch.save()
            
        if 'water' in exch['categories']:
            attrs = {'amount':water_input - river_cooling * water_to_air}
            attrs.update(_amountToFormula(attrs['amount'], exch['amount']))
            exch.update(attrs)
            exch.save()
            
        if 'in water' in exch['categories']:
            attrs = {'amount':water_input}
            attrs.update(_amountToFormula(attrs['amount'], exch['amount']))
            exch.update(attrs)
            exch.save()
```

In [46]:

```
parameters = [params._param_name(param, NameType.NAME) for param in params._param_registry().all()]
```

In [47]:

```
## Preliminary results
```

In [48]:

```
# Setting 100% centrifugation
enrichment_mix_switch.distrib = DistributionType.FIXED
enrichment_mix_switch.default = 'centrifugation'
```

In [49]:

```
multiLCAAlgebric([elec_prod,elec_prod_p], # The model 
    [impacts[0]], # Impacts
#     **{p.name:p.default for p in params._param_registry().all()}
)
```

```
Required param 'enrichment_centr_elec' was missing, replacing by default value : 50
Required param 'river_cooling' was missing, replacing by default value : 1
Required param 'efficiency' was missing, replacing by default value : 0.33
Required param 'integration_time_Rn222' was missing, replacing by default value : 80000
Required param 'ore_grade' was missing, replacing by default value : 0.001544
Required param 'share_ISL' was missing, replacing by default value : 0.574
Required param 'construction_intensity' was missing, replacing by default value : 1
Required param 'mining_electricity_switch' was missing, replacing by default value : dieselgenerator
Required param 'rate_enrichment' was missing, replacing by default value : 0.0415
Required param 'lifetime' was missing, replacing by default value : 60
Required param 'tailings_Rn222' was missing, replacing by default value : 0.01951
Required param 'fuel_fab_elec' was missing, replacing by default value : 36
Required param 'milling_electricity_switch' was missing, replacing by default value : dieselgenerator
Required param 'conversion_elec' was missing, replacing by default value : 11.8
Required param 'availability' was missing, replacing by default value : 0.9
Required param 'conversion_heat' was missing, replacing by default value : 26
```

Out[49]:

|  | climate change - global warming potential (GWP100)[kg CO2-Eq] |
| --- | --- |
| electricity production, nuclear, PWR, THEMIS | 0.006558 |
| electricity production, nuclear, PWR, parameterized | 0.006061 |

In [50]:

```
# Revert back, switch to choose enrichment technique
enrichment_mix_switch = newEnumParam(
    'enrichment_mix_switch',
    label='Enrichment technology',
    values={'centrifugation':0.8,
            'diffusion':0.2},
    default='centrifugation',
    dbname=USER_DB)

# Bins are not properly built, so we do it here 
enrichment_mix_switch._bins = [0]
for i in range(len(enrichment_mix_switch.values)) :
    enumvalue = enrichment_mix_switch.values[i]
    enrichment_mix_switch._bins.append(enrichment_mix_switch._bins[i] + enrichment_mix_switch.weights[enumvalue])
```

```
[ParamRegistry] Param enrichment_mix_switch was already defined in 'Nuclear_DB' : overriding.
```

In [51]:

```
transportation=findActivity(db_name='UNEP_IRP_EUR', code='unep_irp112005')
infra=findActivity(db_name='UNEP_IRP_EUR', code='unep_irp112006')
operating=findActivity(db_name='UNEP_IRP_EUR', code='unep_irp112007')
grid_connection=findActivity(db_name='UNEP_IRP_EUR', code='unep_irp112009')

decommissioning=findActivity('Decommissioning costs', db_name='UNEP_IRP_EUR')
encapsulation=findActivity('Encapsulation, WNA', db_name='UNEP_IRP_EUR')
repository=findActivity('Deep waste repository, WNA', db_name='UNEP_IRP_EUR')
```

In [52]:

```
activities_to_extract=[
    fuel_element_p,
    chemicals_p,
    construction_p,
    transportation,
    infra,
    operating,
    decommissioning,
    grid_connection,
    fuel_element_mix_p,
    enrichment_p,
    conversion_p,
    yellowcake_p,
    milling_p,
    open_pit_p,
    underground_p,
    ISL_p,
    elec_prod_p,
    encapsulation,
    interim_storage,
    repository]
```

In [53]:

```
pd.DataFrame(activities_to_extract).to_csv('data/activities_to_extract.csv')
```

In [ ]:

```

```
