# Supplementary material for: Parametric Life Cycle Assessment of Nuclear Power for Simplified Models
Source: Environ Sci Technol. 2023 Sep 12;57(38):14194–205. doi: 10.1021/acs.est.3c03190 (PMC10537461; doi:10.1021/acs.est.3c03190)
Supplement: Supplementary file 4 — es3c03190_si_004.zip [file es3c03190_si_004.zip › 4_gsa.html]

4\_gsa


# Global sensitivity analysis¶

Now we need to report the first order Sobol indices (S1) for all variable parameters defined ofr the reference LCA and for each impact category evaluated. Some Sobol indices may result in negative values. It is possible to assume convergence and zero values for these Sobol indices if: their 95% confidence intervals bringh very narrow confidence intervals.

In [1]:

```
# Let's get comfortable first
from IPython.core.display import display, HTML
display(HTML("<style>.container { width:80% !important; }</style>"))
```

```
C:\Users\Gibon\AppData\Local\Temp\ipykernel_42128\1931309167.py:2: DeprecationWarning: Importing display from IPython.core.display is deprecated since IPython 7.14, please import from IPython display
  from IPython.core.display import display, HTML
```

In [2]:

```
# There are quite a few modules to import
import brightway2 as bw
import bw2analyzer as bwa
import bw2io
import openpyxl
import pandas as pd
import numpy as np
import scipy as sp
import pickle
import re
from pypardiso import spsolve
import matplotlib.pyplot as plt
from matplotlib.patches import Rectangle
from matplotlib import cm
from lca_algebraic import *
# import networkx as nx
import time
from premise import *
from importlib import reload 

# This is a local function used to easily write the database
from utils.database_writer import database_writer
from utils import utils
```

In [3]:

```
# This forces svg to save text as text
plt.rcParams['svg.fonttype'] = 'none'
```

In [4]:

```
bw.projects.set_current('nuclear_param')
```

In [5]:

```
loadParams()
```

```
[ParamRegistry] Param share_ISL was already defined in 'Nuclear_DB' : overriding.
Warning : LogNormal does not support min/max boundaries for parameter :  ore_grade
[ParamRegistry] Param ore_grade was already defined in 'Nuclear_DB' : overriding.
[ParamRegistry] Param integration_time_Rn222 was already defined in 'Nuclear_DB' : overriding.
[ParamRegistry] Param tailings_Rn222 was already defined in 'Nuclear_DB' : overriding.
[ParamRegistry] Param conversion_elec was already defined in 'Nuclear_DB' : overriding.
[ParamRegistry] Param conversion_heat was already defined in 'Nuclear_DB' : overriding.
[ParamRegistry] Param rate_enrichment was already defined in 'Nuclear_DB' : overriding.
[ParamRegistry] Param rate_feed was already defined in 'Nuclear_DB' : overriding.
[ParamRegistry] Param rate_tailings was already defined in 'Nuclear_DB' : overriding.
[ParamRegistry] Param enrichment_centr_elec was already defined in 'Nuclear_DB' : overriding.
[ParamRegistry] Param enrichment_diff_elec was already defined in 'Nuclear_DB' : overriding.
[ParamRegistry] Param enrichment_centr_share was already defined in 'Nuclear_DB' : overriding.
[ParamRegistry] Param fuel_fab_elec was already defined in 'Nuclear_DB' : overriding.
[ParamRegistry] Param lifetime was already defined in 'Nuclear_DB' : overriding.
[ParamRegistry] Param capacity was already defined in 'Nuclear_DB' : overriding.
[ParamRegistry] Param construction_intensity was already defined in 'Nuclear_DB' : overriding.
[ParamRegistry] Param efficiency was already defined in 'Nuclear_DB' : overriding.
[ParamRegistry] Param availability was already defined in 'Nuclear_DB' : overriding.
[ParamRegistry] Param river_cooling was already defined in 'Nuclear_DB' : overriding.
[ParamRegistry] Param mining_electricity_switch was already defined in 'Nuclear_DB' : overriding.
[ParamRegistry] Param milling_electricity_switch was already defined in 'Nuclear_DB' : overriding.
[ParamRegistry] Param enrichment_mix_switch was already defined in 'Nuclear_DB' : overriding.
```

In [6]:

```
elec_prod_p = findActivity(name='electricity production, nuclear, PWR, parameterized', db_name='Nuclear_DB', loc='GLO')
elec_prod_p
```

Out[6]:

```
'electricity production, nuclear, PWR, parameterized' (kilowatt hour, GLO, None)
```

In [7]:

```
# List of impacts to consider
impacts_all = [m for m in bw.methods if 'EF v3.0'== m[0] if len(m)==3]

# Select 9 categories
impacts = [impacts_all[i] for i in [1, 5, 10, 13, 17, 21, 22, 23, 27]]
```

In [8]:

```
USER_DB = 'Nuclear_DB'
```

## Calculating Sobol indices¶

Here we calculate the first-order Sobol indices and plot it as a bar graph.

In [9]:

```
# First let's assign a color to each parameter, to remain consistent across figures
```

In [10]:

```
enrichment_mix_switch = newEnumParam(
    'enrichment_mix_switch',
    label='Enrichment technology',
    values={'centrifugation':0.8,
            'diffusion':0.2},
    default='centrifugation',
    dbname=USER_DB)

# Bins are not properly built, so we do it here 
enrichment_mix_switch._bins = [0]
for i in range(len(enrichment_mix_switch.values)) :
    enumvalue = enrichment_mix_switch.values[i]
    enrichment_mix_switch._bins.append(enrichment_mix_switch._bins[i] + enrichment_mix_switch.weights[enumvalue])
```

```
[ParamRegistry] Param enrichment_mix_switch was already defined in 'Nuclear_DB' : overriding.
```

In [12]:

```
params = sorted(stats._param_registry().keys())
```

In [13]:

```
param_labels = [stats._param_registry()[p].label for p in params]
[param_labels.remove(x) for x in ['Uranium enrichment tailings rate','Uranium feed rate','Share of centrifugation in enrichment']]
```

Out[13]:

```
[None, None, None]
```

In [14]:

```
param_labels
```

Out[14]:

```
['Availability of power plant',
 'Nameplate capacity',
 'Intensity of construction inputs\ncompared with default values',
 'Conversion electricity input',
 'Conversion heat input',
 'Efficiency of electricity generation',
 'Enrichment electricity, centrifuge',
 'Enrichment electricity, diffusion',
 'Enrichment technology',
 'Fuel fabrication electricity',
 'Integration time for radiation from milling tailings',
 'Lifetime of plant and on-site equipment',
 'Milling electricity, grid or diesel',
 'Mining electricity, grid or diesel',
 'Uranium ore grade',
 'Uranium enrichment rate',
 'River cooling',
 'Share of ISL, the rest is rescaled in proportion',
 'Rn222 from tailings, in Bq/s']
```

In [15]:

```
tab24 = utils.categorical_cmap(6,4,cmap='tab10')
```

In [16]:

```
colors = dict(zip(param_labels,tab24.colors))
```

In [20]:

```
n = 2**10

lambdas = stats._preMultiLCAAlgebric(elec_prod_p, impacts)
var_params = stats._extract_var_params(lambdas)

problem, _, Y = stats._stochastics(lambdas, impacts, n, var_params)

print("Processing Sobol indices ...")
sob = stats._sobols(impacts, problem, Y)
```

```
Generating samples ...
Transforming samples ...
Processing Sobol indices ...
Processing sobol for ('EF v3.0', 'climate change', 'global warming potential (GWP100)')
Processing sobol for ('EF v3.0', 'ecotoxicity: freshwater', 'comparative toxic unit for ecosystems (CTUe) ')
Processing sobol for ('EF v3.0', 'eutrophication: freshwater', 'fraction of nutrients reaching freshwater end compartment (P)')
Processing sobol for ('EF v3.0', 'human toxicity: carcinogenic', 'comparative toxic unit for human (CTUh) ')
Processing sobol for ('EF v3.0', 'human toxicity: non-carcinogenic', 'comparative toxic unit for human (CTUh) ')
Processing sobol for ('EF v3.0', 'ionising radiation: human health', 'human exposure efficiency relative to u235')
Processing sobol for ('EF v3.0', 'land use', 'soil quality index')
Processing sobol for ('EF v3.0', 'material resources: metals/minerals', 'abiotic depletion potential (ADP): elements (ultimate reserves)')
Processing sobol for ('EF v3.0', 'water use', 'user deprivation potential (deprivation-weighted water consumption)')
```

In [17]:

```
ind_norm = ['climate change - global warming potential (GWP100) [g CO2-Eq]',
       'ecotoxicity: freshwater - comparative toxic unit for ecosystems (CTUe) [CTUe]',
       'eutrophication: freshwater - fraction of nutrients reaching freshwater end compartment (P) [mg PO4-Eq]',
       'human toxicity: carcinogenic - comparative toxic unit for human (CTUh) [10$^{-12}$ CTUh]',
       'human toxicity: non-carcinogenic - comparative toxic unit for human (CTUh) [10$^{-12}$ CTUh]',
       'ionising radiation: human health - human exposure efficiency relative to u235 [Bq U235-Eq]',
       'land use - soil quality index [10$^{-3}$]',
       'material resources: metals/minerals - abiotic depletion potential (ADP): elements (ultimate reserves) [μg Sb-Eq]',
       'water use - user deprivation potential (deprivation-weighted water consumption) [l world eq. deprived]']
ind_norm = [ind.split(' - ')[0] for ind in ind_norm]
ind_clean = [ind[0].upper() + ind[1:] for ind in ind_norm]
```

In [22]:

```
sob_s1 = pd.DataFrame(sob.s1, index=[p.label for p in var_params], columns=ind_clean)
sob_s1.to_csv('output/sobol_full.csv')
```

In [23]:

```
sns.set(font_scale=1)
sns.set_style('whitegrid')

ax = sob_s1.T.plot(kind='barh',stacked=True,figsize=(11,6), color=colors)
ax.legend(loc='upper center', bbox_to_anchor=(0.5, -0.15),fancybox=False, shadow=False, ncol=3, frameon = True)
# ax.set_xlabel('First-order Sobol indices')
ax.set_xlim(0,1)
ax.set_title('First-order Sobol indices per impact category')

plt.tight_layout()
plt.savefig('output/sobol_full.svg')
```

In [24]:

```
# Setting 100% centrifugation
enrichment_mix_switch.distrib = DistributionType.FIXED
enrichment_mix_switch.default = 'centrifugation'
```

In [25]:

```
lambdas = stats._preMultiLCAAlgebric(elec_prod_p, impacts)
var_params = stats._extract_var_params(lambdas)

problem, _, Y = stats._stochastics(lambdas, impacts, n, var_params)

print("Processing Sobol indices ...")
sob = stats._sobols(impacts, problem, Y)
```

```
Generating samples ...
Transforming samples ...
Processing Sobol indices ...
Processing sobol for ('EF v3.0', 'climate change', 'global warming potential (GWP100)')
Processing sobol for ('EF v3.0', 'ecotoxicity: freshwater', 'comparative toxic unit for ecosystems (CTUe) ')
Processing sobol for ('EF v3.0', 'eutrophication: freshwater', 'fraction of nutrients reaching freshwater end compartment (P)')
Processing sobol for ('EF v3.0', 'human toxicity: carcinogenic', 'comparative toxic unit for human (CTUh) ')
Processing sobol for ('EF v3.0', 'human toxicity: non-carcinogenic', 'comparative toxic unit for human (CTUh) ')
Processing sobol for ('EF v3.0', 'ionising radiation: human health', 'human exposure efficiency relative to u235')
Processing sobol for ('EF v3.0', 'land use', 'soil quality index')
Processing sobol for ('EF v3.0', 'material resources: metals/minerals', 'abiotic depletion potential (ADP): elements (ultimate reserves)')
Processing sobol for ('EF v3.0', 'water use', 'user deprivation potential (deprivation-weighted water consumption)')
```

In [26]:

```
sob_s1_centr = pd.DataFrame(sob.s1, index=[p.label for p in var_params], columns=ind_clean)
sob_s1_centr.to_csv('output/sobol_centr.csv')
```

In [27]:

```
sns.set(font_scale=1)
sns.set_style('whitegrid')

ax = sob_s1_centr.T.plot(kind='barh',stacked=True,figsize=(11,6), color=colors)
ax.legend(loc='upper center', bbox_to_anchor=(0.5, -0.15),fancybox=False, shadow=False, ncol=3, frameon = True)
# ax.set_xlabel('First-order Sobol indices')
ax.set_xlim(0,1)
ax.set_title('First-order Sobol indices per impact category, with 100% centrifugation enrichment')

plt.tight_layout()
plt.savefig('output/sobol_centr.svg')
```

In [28]:

```
# Setting 100% diffusion
enrichment_mix_switch.distrib = DistributionType.FIXED
enrichment_mix_switch.default = 'diffusion'
```

In [29]:

```
lambdas = stats._preMultiLCAAlgebric(elec_prod_p, impacts)
var_params = stats._extract_var_params(lambdas)

problem, _, Y = stats._stochastics(lambdas, impacts, n, var_params)

print("Processing Sobol indices ...")
sob = stats._sobols(impacts, problem, Y)
```

```
Generating samples ...
Transforming samples ...
Processing Sobol indices ...
Processing sobol for ('EF v3.0', 'climate change', 'global warming potential (GWP100)')
Processing sobol for ('EF v3.0', 'ecotoxicity: freshwater', 'comparative toxic unit for ecosystems (CTUe) ')
Processing sobol for ('EF v3.0', 'eutrophication: freshwater', 'fraction of nutrients reaching freshwater end compartment (P)')
Processing sobol for ('EF v3.0', 'human toxicity: carcinogenic', 'comparative toxic unit for human (CTUh) ')
Processing sobol for ('EF v3.0', 'human toxicity: non-carcinogenic', 'comparative toxic unit for human (CTUh) ')
Processing sobol for ('EF v3.0', 'ionising radiation: human health', 'human exposure efficiency relative to u235')
Processing sobol for ('EF v3.0', 'land use', 'soil quality index')
Processing sobol for ('EF v3.0', 'material resources: metals/minerals', 'abiotic depletion potential (ADP): elements (ultimate reserves)')
Processing sobol for ('EF v3.0', 'water use', 'user deprivation potential (deprivation-weighted water consumption)')
```

In [18]:

```
sob_s1_diff = pd.DataFrame(sob.s1, index=[p.label for p in var_params], columns=ind_clean)
sob_s1_diff.to_csv('output/sobol_diff.csv')
```

```
---------------------------------------------------------------------------
NameError                                 Traceback (most recent call last)
Cell In [18], line 1
----> 1 sob_s1_diff = pd.DataFrame(sob.s1, index=[p.label for p in var_params], columns=ind_clean)
      2 sob_s1_diff.to_csv('output/sobol_diff.csv')

NameError: name 'sob' is not defined
```

In [19]:

```
tab24
```

Out[19]:

**from\_list**

under

bad

over

In [20]:

```
param_labels_ordered = [
    'Uranium ore grade',
    'Mining electricity, grid or diesel',
    'Milling electricity, grid or diesel',
    'Share of ISL, the rest is rescaled in proportion',
    'Rn222 from tailings, in Bq/s',
    'Integration time for radiation from milling tailings',
    'Conversion electricity input',
    'Conversion heat input',
    'Enrichment technology',
    'Enrichment electricity, centrifuge',
    'Enrichment electricity, diffusion',
    'Uranium enrichment rate',
    'Fuel fabrication electricity',
    'Nameplate capacity',
    'Availability of power plant',
    'Efficiency of electricity generation',
    'River cooling',
    'Lifetime of plant and on-site equipment',
    'Intensity of construction inputs\ncompared with default values',
]
```

In [21]:

```
param_labels_ordered
```

Out[21]:

```
['Uranium ore grade',
 'Mining electricity, grid or diesel',
 'Milling electricity, grid or diesel',
 'Share of ISL, the rest is rescaled in proportion',
 'Rn222 from tailings, in Bq/s',
 'Integration time for radiation from milling tailings',
 'Conversion electricity input',
 'Conversion heat input',
 'Enrichment technology',
 'Enrichment electricity, centrifuge',
 'Enrichment electricity, diffusion',
 'Uranium enrichment rate',
 'Fuel fabrication electricity',
 'Nameplate capacity',
 'Availability of power plant',
 'Efficiency of electricity generation',
 'River cooling',
 'Lifetime of plant and on-site equipment',
 'Intensity of construction inputs\ncompared with default values']
```

In [22]:

```
colors = dict(zip(param_labels_ordered,tab24.colors))
```

In [23]:

```
colors['Fuel fabrication electricity'] = np.array([1., 1., 85./255])
```

In [36]:

```
fig, axes = plt.subplots(1, 3, figsize=(11,6), sharey=True)

data_to_plot = [sob_s1, sob_s1_centr, sob_s1_diff]
titles = ['Full model',
          'Enrichment set to centrifugation only',
          'Enrichment set to gaseous diffusion only']

for i,ax in enumerate(axes):
    
    data_to_plot[i].reindex(param_labels_ordered).T.plot(kind='barh', stacked=True, color=colors, ax=ax)
    ax.legend('', frameon=False)
    
    # ax.set_xlabel('First-order Sobol indices')
#     plot.plot(kind='barh', stacked=True, color=colors, ax=ax)
    ax.set_xlim(0,1)
    ax.set_title(titles[i])

fig.legend(param_labels_ordered, loc='upper center', bbox_to_anchor=(.5,0), ncol=3, bbox_transform=fig.transFigure)

plt.savefig("output/sobol_indices_all.png",
            transparent=False,
            bbox_inches='tight',
            pad_inches=0,
            dpi=300)
plt.savefig("output/sobol_indices_all.svg",
            transparent=False,
            bbox_inches='tight',
            pad_inches=0,
            dpi=300)
```

In [37]:

```
sns.set(font_scale=.5)
sob_summary = pd.concat(data_to_plot, keys=titles)
sns.heatmap(sob_summary)
```

Out[37]:

```
<AxesSubplot:ylabel='None-None'>
```

In [38]:

```
sob_summary.to_csv('output/sob_all_indices.csv')
```

In [25]:

```
sob_summary = pd.read_csv('output/sob_all_indices.csv', index_col=[0,1])
```

In [27]:

```
sns.set(font_scale=1)
sns.set_style('whitegrid')

fig, axes = plt.subplots(1, 3, figsize=(11,6), sharey=True)

titles = ['Full model',
          'Enrichment set to centrifugation only',
          'Enrichment set to gaseous diffusion only']
data_to_plot = [sob_summary.loc[t] for t in titles]

for i,ax in enumerate(axes):
    
    data_to_plot[i].reindex(param_labels_ordered).T.plot(kind='barh', stacked=True, color=colors, ax=ax)
    ax.legend('', frameon=False)
    
    # ax.set_xlabel('First-order Sobol indices')
#     plot.plot(kind='barh', stacked=True, color=colors, ax=ax)
    ax.set_xlim(0,1)
    ax.set_title(titles[i])

fig.legend(param_labels_ordered, loc='upper center', bbox_to_anchor=(.5,0), ncol=3, bbox_transform=fig.transFigure)

plt.savefig("output/sobol_indices_all.png",
            transparent=False,
            bbox_inches='tight',
            pad_inches=0,
            dpi=300)
plt.savefig("output/sobol_indices_all.pdf",
            transparent=False,
            bbox_inches='tight',
            pad_inches=0,
            dpi=300)
```

In [ ]:

```

```
