# Supplementary material for: Parametric Life Cycle Assessment of Nuclear Power for Simplified Models
Source: Environ Sci Technol. 2023 Sep 12;57(38):14194–205. doi: 10.1021/acs.est.3c03190 (PMC10537461; doi:10.1021/acs.est.3c03190)
Supplement: Supplementary file 4 — es3c03190_si_004.zip [file es3c03190_si_004.zip › images/3_lcia.html]

3\_lcia


# lala

# lala

# LCIA¶

This notebook performs the life cycle impact assessment for the nuclear power life cycle inventory, including:

- a (foreground) contribution analysis,
- a calculation of LCIA models preserving parameters,
- a scenario analysis.

In [1]:

```
# Let's get comfortable first
from IPython.core.display import display, HTML
display(HTML("<style>.container { width:80% !important; }</style>"))
```

```
C:\Users\Gibon\AppData\Local\Temp\ipykernel_26960\1931309167.py:2: DeprecationWarning: Importing display from IPython.core.display is deprecated since IPython 7.14, please import from IPython display
  from IPython.core.display import display, HTML
```

In [50]:

```
# There are quite a few modules to import
import brightway2 as bw
import bw2analyzer as bwa
import bw2io
import openpyxl
import pandas as pd
import numpy as np
import scipy as sp
import pickle
import re
import matplotlib.pyplot as plt
import time

from pypardiso import spsolve
from matplotlib.patches import Rectangle
from matplotlib import cm
from lca_algebraic import *
# import networkx as nx
from premise import *
from importlib import reload 
from sympy import N, sympify, preview
from sympy.printing import mathematica_code

# This is a local function used to easily write the database
from utils.database_writer import database_writer
from utils import utils
```

In [3]:

```
# This forces svg to save text as text
plt.rcParams['svg.fonttype'] = 'none'
```

In [4]:

```
bw.projects.set_current('nuclear_param')
```

In [5]:

```
nuc_db = bw.Database('Nuclear_DB')
```

In [6]:

```
unep_db = bw.Database('UNEP_IRP_EUR')
```

In [7]:

```
loadParams()
```

```
[ParamRegistry] Param share_ISL was already defined in 'Nuclear_DB' : overriding.
Warning : LogNormal does not support min/max boundaries for parameter :  ore_grade
[ParamRegistry] Param ore_grade was already defined in 'Nuclear_DB' : overriding.
[ParamRegistry] Param integration_time_Rn222 was already defined in 'Nuclear_DB' : overriding.
[ParamRegistry] Param tailings_Rn222 was already defined in 'Nuclear_DB' : overriding.
[ParamRegistry] Param conversion_elec was already defined in 'Nuclear_DB' : overriding.
[ParamRegistry] Param conversion_heat was already defined in 'Nuclear_DB' : overriding.
[ParamRegistry] Param rate_enrichment was already defined in 'Nuclear_DB' : overriding.
[ParamRegistry] Param rate_feed was already defined in 'Nuclear_DB' : overriding.
[ParamRegistry] Param rate_tailings was already defined in 'Nuclear_DB' : overriding.
[ParamRegistry] Param enrichment_centr_elec was already defined in 'Nuclear_DB' : overriding.
[ParamRegistry] Param enrichment_diff_elec was already defined in 'Nuclear_DB' : overriding.
[ParamRegistry] Param enrichment_centr_share was already defined in 'Nuclear_DB' : overriding.
[ParamRegistry] Param fuel_fab_elec was already defined in 'Nuclear_DB' : overriding.
[ParamRegistry] Param lifetime was already defined in 'Nuclear_DB' : overriding.
[ParamRegistry] Param capacity was already defined in 'Nuclear_DB' : overriding.
[ParamRegistry] Param construction_intensity was already defined in 'Nuclear_DB' : overriding.
[ParamRegistry] Param efficiency was already defined in 'Nuclear_DB' : overriding.
[ParamRegistry] Param availability was already defined in 'Nuclear_DB' : overriding.
[ParamRegistry] Param river_cooling was already defined in 'Nuclear_DB' : overriding.
[ParamRegistry] Param mining_electricity_switch was already defined in 'Nuclear_DB' : overriding.
[ParamRegistry] Param milling_electricity_switch was already defined in 'Nuclear_DB' : overriding.
[ParamRegistry] Param enrichment_mix_switch was already defined in 'Nuclear_DB' : overriding.
```

## Contribution analysis¶

In [8]:

```
activities_foreground=pd.read_csv('data/activities_to_extract.csv', index_col=0)
```

In [9]:

```
activities_to_extract=[]

for n,d,l in activities_foreground[['name','database','location']].values:
    
    if 'electricity production' in n:
        print(n,d,l)
        elec_prod_p = findActivity(name=n, db_name=d, loc=l)
    
    activities_to_extract.append(findActivity(name=n, db_name=d, loc=l))
```

```
electricity production, nuclear, PWR, parameterized Nuclear_DB GLO
```

In [10]:

```
# This is how we will group and order our activities

process_agg = {
#                  "'Market for nuclear fuel element, for PWR, parameterized' (kilogram, GLO, None)":'Fuel fabrication',
                 "'Chemicals, use phase, parameterized' (unit, GLO, None)":'Electricity production',
                 "'Construction, parameterized' (unit, GLO, None)":'Construction',
                 "'Transportation' (unit, GLO, None)":'Transportation',
#                  "'Infrastructure elements and overhead costs' (unit, GLO, None)":'Construction',
                 "'Operating expenses' (unit, GLO, None)":'Electricity production',
                 "'Decommissioning costs' (unit, GLO, None)":'Decommissioning',
                 "'External cabling, overhead line' (kilometer, GLO, None)":'Grid connection',
                 "'Fuel elements, parameterized' (kilogram, GLO, None)":'Fuel fabrication',
                 "'Uranium production, enriched, parameterized' (unit, GLO, None)":'Enrichment',
                 "'Market for uranium hexafluoride, parameterized' (kilogram, GLO, None)":'Conversion',
                 "'Market for uranium, in yellowcake, parameterized' (kilogram, GLO, None)":'Milling',
#                  "'Market for milling, uranium, in yellowcake, parameterized' (kilogram, GLO, None)":'Milling',
                 "'Uranium mine operation, open cast, parameterized' (kilogram, GLO, None)":'Mining, open pit',
                 "'Uranium mine operation, underground, parameterized' (kilogram, GLO, None)":'Mining, underground',
                 "'Uranium mine operation, in-situ leaching, parameterized' (kilogram, GLO, None)":'Mining, ISL',
                 "'electricity production, nuclear, PWR, parameterized' (kilowatt hour, GLO, None)":'Electricity production',
                 "'Encapsulation, WNA' (year, EUR, None)":'Spent fuel management',
                 "'Interim storage of spent fuel, WNA' (year, GLO, None)":'Spent fuel management',
                 "'Deep waste repository, WNA' (year, EUR, None)":'Spent fuel management'}

order=pd.read_excel('data/foreground.xlsx', index_col=0)
```

In [11]:

```
# List of impacts to consider
impacts_all = [m for m in bw.methods if 'EF v3.0'== m[0] if len(m)==3]

# Select 9 categories
impacts = [impacts_all[i] for i in [1, 5, 10, 13, 17, 21, 22, 23, 27]]

contrib_an = dict()

for a in activities_to_extract:
    print(str(a))
    contrib_an[str(a)] = multiLCAAlgebric(elec_prod_p, # The model 
                     impacts, # Impacts
                     extract_activities=[a]
    )
```

```
'Market for nuclear fuel element, for PWR, parameterized' (kilogram, GLO, None)
```

```
Required param 'integration_time_Rn222' was missing, replacing by default value : 80000
Required param 'efficiency' was missing, replacing by default value : 0.33
Required param 'enrichment_centr_elec' was missing, replacing by default value : 50
Required param 'mining_electricity_switch' was missing, replacing by default value : dieselgenerator
Required param 'tailings_Rn222' was missing, replacing by default value : 0.01951
Required param 'milling_electricity_switch' was missing, replacing by default value : dieselgenerator
Required param 'enrichment_diff_elec' was missing, replacing by default value : 2500
Required param 'fuel_fab_elec' was missing, replacing by default value : 36
Required param 'conversion_elec' was missing, replacing by default value : 11.8
Required param 'share_ISL' was missing, replacing by default value : 0.574
Required param 'enrichment_mix_switch' was missing, replacing by default value : centrifugation
Required param 'rate_enrichment' was missing, replacing by default value : 0.0415
Required param 'ore_grade' was missing, replacing by default value : 0.001544
Required param 'conversion_heat' was missing, replacing by default value : 26
```

```
'Chemicals, use phase, parameterized' (unit, GLO, None)
```

```
Required param 'river_cooling' was missing, replacing by default value : 1.0
```

```
'Construction, parameterized' (unit, GLO, None)
```

```
Required param 'lifetime' was missing, replacing by default value : 60
Required param 'construction_intensity' was missing, replacing by default value : 1
Required param 'availability' was missing, replacing by default value : 0.9
```

```
'Transportation' (unit, GLO, None)
'Infrastructure elements and overhead costs' (unit, GLO, None)
'Operating expenses' (unit, GLO, None)
'Decommissioning costs' (unit, GLO, None)
'External cabling, overhead line' (kilometer, GLO, None)
'Fuel elements, parameterized' (kilogram, GLO, None)
```

```
Required param 'integration_time_Rn222' was missing, replacing by default value : 80000
Required param 'efficiency' was missing, replacing by default value : 0.33
Required param 'enrichment_centr_elec' was missing, replacing by default value : 50
Required param 'mining_electricity_switch' was missing, replacing by default value : dieselgenerator
Required param 'tailings_Rn222' was missing, replacing by default value : 0.01951
Required param 'milling_electricity_switch' was missing, replacing by default value : dieselgenerator
Required param 'enrichment_diff_elec' was missing, replacing by default value : 2500
Required param 'fuel_fab_elec' was missing, replacing by default value : 36
Required param 'conversion_elec' was missing, replacing by default value : 11.8
Required param 'share_ISL' was missing, replacing by default value : 0.574
Required param 'enrichment_mix_switch' was missing, replacing by default value : centrifugation
Required param 'rate_enrichment' was missing, replacing by default value : 0.0415
Required param 'ore_grade' was missing, replacing by default value : 0.001544
Required param 'conversion_heat' was missing, replacing by default value : 26
```

```
'Uranium production, enriched, parameterized' (unit, GLO, None)
```

```
Required param 'integration_time_Rn222' was missing, replacing by default value : 80000
Required param 'efficiency' was missing, replacing by default value : 0.33
Required param 'enrichment_centr_elec' was missing, replacing by default value : 50
Required param 'mining_electricity_switch' was missing, replacing by default value : dieselgenerator
Required param 'tailings_Rn222' was missing, replacing by default value : 0.01951
Required param 'milling_electricity_switch' was missing, replacing by default value : dieselgenerator
Required param 'enrichment_diff_elec' was missing, replacing by default value : 2500
Required param 'conversion_elec' was missing, replacing by default value : 11.8
Required param 'share_ISL' was missing, replacing by default value : 0.574
Required param 'enrichment_mix_switch' was missing, replacing by default value : centrifugation
Required param 'rate_enrichment' was missing, replacing by default value : 0.0415
Required param 'ore_grade' was missing, replacing by default value : 0.001544
Required param 'conversion_heat' was missing, replacing by default value : 26
```

```
'Market for uranium hexafluoride, parameterized' (kilogram, GLO, None)
```

```
Required param 'integration_time_Rn222' was missing, replacing by default value : 80000
Required param 'efficiency' was missing, replacing by default value : 0.33
Required param 'mining_electricity_switch' was missing, replacing by default value : dieselgenerator
Required param 'tailings_Rn222' was missing, replacing by default value : 0.01951
Required param 'milling_electricity_switch' was missing, replacing by default value : dieselgenerator
Required param 'share_ISL' was missing, replacing by default value : 0.574
Required param 'conversion_elec' was missing, replacing by default value : 11.8
Required param 'enrichment_mix_switch' was missing, replacing by default value : centrifugation
Required param 'rate_enrichment' was missing, replacing by default value : 0.0415
Required param 'ore_grade' was missing, replacing by default value : 0.001544
Required param 'conversion_heat' was missing, replacing by default value : 26
```

```
'Market for uranium, in yellowcake, parameterized' (kilogram, GLO, None)
```

```
Required param 'integration_time_Rn222' was missing, replacing by default value : 80000
Required param 'efficiency' was missing, replacing by default value : 0.33
Required param 'mining_electricity_switch' was missing, replacing by default value : dieselgenerator
Required param 'tailings_Rn222' was missing, replacing by default value : 0.01951
Required param 'milling_electricity_switch' was missing, replacing by default value : dieselgenerator
Required param 'share_ISL' was missing, replacing by default value : 0.574
Required param 'enrichment_mix_switch' was missing, replacing by default value : centrifugation
Required param 'rate_enrichment' was missing, replacing by default value : 0.0415
Required param 'ore_grade' was missing, replacing by default value : 0.001544
```

```
'Market for milling, uranium, in yellowcake, parameterized' (kilogram, GLO, None)
```

```
Required param 'integration_time_Rn222' was missing, replacing by default value : 80000
Required param 'efficiency' was missing, replacing by default value : 0.33
Required param 'mining_electricity_switch' was missing, replacing by default value : dieselgenerator
Required param 'tailings_Rn222' was missing, replacing by default value : 0.01951
Required param 'milling_electricity_switch' was missing, replacing by default value : dieselgenerator
Required param 'share_ISL' was missing, replacing by default value : 0.574
Required param 'enrichment_mix_switch' was missing, replacing by default value : centrifugation
Required param 'rate_enrichment' was missing, replacing by default value : 0.0415
Required param 'ore_grade' was missing, replacing by default value : 0.001544
```

```
'Uranium mine operation, open cast, parameterized' (kilogram, GLO, None)
```

```
Required param 'efficiency' was missing, replacing by default value : 0.33
Required param 'mining_electricity_switch' was missing, replacing by default value : dieselgenerator
Required param 'share_ISL' was missing, replacing by default value : 0.574
Required param 'enrichment_mix_switch' was missing, replacing by default value : centrifugation
Required param 'rate_enrichment' was missing, replacing by default value : 0.0415
Required param 'ore_grade' was missing, replacing by default value : 0.001544
```

```
'Uranium mine operation, underground, parameterized' (kilogram, GLO, None)
```

```
Required param 'efficiency' was missing, replacing by default value : 0.33
Required param 'mining_electricity_switch' was missing, replacing by default value : dieselgenerator
Required param 'share_ISL' was missing, replacing by default value : 0.574
Required param 'enrichment_mix_switch' was missing, replacing by default value : centrifugation
Required param 'rate_enrichment' was missing, replacing by default value : 0.0415
Required param 'ore_grade' was missing, replacing by default value : 0.001544
```

```
'Uranium mine operation, in-situ leaching, parameterized' (kilogram, GLO, None)
```

```
Required param 'efficiency' was missing, replacing by default value : 0.33
Required param 'mining_electricity_switch' was missing, replacing by default value : dieselgenerator
Required param 'share_ISL' was missing, replacing by default value : 0.574
Required param 'enrichment_mix_switch' was missing, replacing by default value : centrifugation
Required param 'rate_enrichment' was missing, replacing by default value : 0.0415
Required param 'ore_grade' was missing, replacing by default value : 0.001544
```

```
'electricity production, nuclear, PWR, parameterized' (kilowatt hour, GLO, None)
'Encapsulation, WNA' (year, EUR, None)
'Interim storage of spent fuel, WNA' (year, GLO, None)
'Deep waste repository, WNA' (year, EUR, None)
```

In [12]:

```
contr_df = pd.concat(contrib_an)
direct_inputs = pd.MultiIndex.from_tuples([(str(e.input),elec_prod_p['name']) for e in elec_prod_p.exchanges()])
contr_df_fg = (contr_df.loc[set(direct_inputs) & set(contr_df.index)]*1000).droplevel(1, axis=0)
contr_df = contr_df.droplevel(1, axis=0)
```

```
C:\Users\Gibon\AppData\Local\Temp\ipykernel_26960\2144738136.py:3: FutureWarning: Passing a set as an indexer is deprecated and will raise in a future version. Use a list instead.
  contr_df_fg = (contr_df.loc[set(direct_inputs) & set(contr_df.index)]*1000).droplevel(1, axis=0)
```

In [13]:

```
contr_df_agg = contr_df.groupby(process_agg, axis=0).sum().reindex(order.index)
```

In [14]:

```
contr_df_to_plot = order.T.dot(contr_df_agg).T
method_units, _ = utils.get_human_methods(impacts)
ind_norm = ['climate change - global warming potential (GWP100) [g CO2-Eq]',
       'ecotoxicity: freshwater - comparative toxic unit for ecosystems (CTUe) [CTUe]',
       'eutrophication: freshwater - fraction of nutrients reaching freshwater end compartment (P) [mg PO4-Eq]',
       'human toxicity: carcinogenic - comparative toxic unit for human (CTUh) [10$^{-12}$ CTUh]',
       'human toxicity: non-carcinogenic - comparative toxic unit for human (CTUh) [10$^{-12}$ CTUh]',
       'ionising radiation: human health - human exposure efficiency relative to u235 [Bq U235-Eq]',
       'land use - soil quality index [10$^{-3}$]',
       'material resources: metals/minerals - abiotic depletion potential (ADP): elements (ultimate reserves) [μg Sb-Eq]',
       'water use - user deprivation potential (deprivation-weighted water consumption) [l world eq. deprived]']
ind_norm = [ind.split(' - ')[0] for ind in ind_norm]
```

In [15]:

```
# Use clean names for the methods
ind_clean = [ind[0].upper() + ind[1:] for ind in ind_norm]
contr_df_to_plot.index = ind_clean
```

In [16]:

```
list(elec_prod_p.exchanges())
```

Out[16]:

```
[Exchange: 0 kilogram 'Fuel elements, parameterized' (kilogram, GLO, None) to 'electricity production, nuclear, PWR, parameterized' (kilowatt hour, GLO, None)>,
 Exchange: 1 unit 'Chemicals, use phase, parameterized' (unit, GLO, None) to 'electricity production, nuclear, PWR, parameterized' (kilowatt hour, GLO, None)>,
 Exchange: 0 unit 'Construction, parameterized' (unit, GLO, None) to 'electricity production, nuclear, PWR, parameterized' (kilowatt hour, GLO, None)>,
 Exchange: 1 unit 'Transportation' (unit, GLO, None) to 'electricity production, nuclear, PWR, parameterized' (kilowatt hour, GLO, None)>,
 Exchange: 2.11253918760193e-12 unit 'Infrastructure elements and overhead costs' (unit, GLO, None) to 'electricity production, nuclear, PWR, parameterized' (kilowatt hour, GLO, None)>,
 Exchange: 2.11253918760193e-12 unit 'Decommissioning costs' (unit, GLO, None) to 'electricity production, nuclear, PWR, parameterized' (kilowatt hour, GLO, None)>,
 Exchange: 2.2677408199016934e-11 year 'Interim storage of spent fuel, WNA' (year, GLO, None) to 'electricity production, nuclear, PWR, parameterized' (kilowatt hour, GLO, None)>,
 Exchange: 1.433563012155585e-11 year 'Encapsulation, WNA' (year, EUR, None) to 'electricity production, nuclear, PWR, parameterized' (kilowatt hour, GLO, None)>,
 Exchange: 1.433563012155585e-11 year 'Deep waste repository, WNA' (year, EUR, None) to 'electricity production, nuclear, PWR, parameterized' (kilowatt hour, GLO, None)>,
 Exchange: 1 kilowatt hour 'electricity production, nuclear, PWR, parameterized' (kilowatt hour, GLO, None) to 'electricity production, nuclear, PWR, parameterized' (kilowatt hour, GLO, None)>]
```

In [17]:

```
d = multiLCAAlgebric(elec_prod_p, # The model 
    impacts, # Impacts
#     **{p.name:p.default for p in params._param_registry().all()}
)
```

```
Required param 'river_cooling' was missing, replacing by default value : 1.0
Required param 'integration_time_Rn222' was missing, replacing by default value : 80000
Required param 'efficiency' was missing, replacing by default value : 0.33
Required param 'enrichment_centr_elec' was missing, replacing by default value : 50
Required param 'mining_electricity_switch' was missing, replacing by default value : dieselgenerator
Required param 'tailings_Rn222' was missing, replacing by default value : 0.01951
Required param 'lifetime' was missing, replacing by default value : 60
Required param 'availability' was missing, replacing by default value : 0.9
Required param 'milling_electricity_switch' was missing, replacing by default value : dieselgenerator
Required param 'enrichment_diff_elec' was missing, replacing by default value : 2500
Required param 'fuel_fab_elec' was missing, replacing by default value : 36
Required param 'conversion_elec' was missing, replacing by default value : 11.8
Required param 'share_ISL' was missing, replacing by default value : 0.574
Required param 'construction_intensity' was missing, replacing by default value : 1
Required param 'enrichment_mix_switch' was missing, replacing by default value : centrifugation
Required param 'rate_enrichment' was missing, replacing by default value : 0.0415
Required param 'ore_grade' was missing, replacing by default value : 0.001544
Required param 'conversion_heat' was missing, replacing by default value : 26
```

In [18]:

```
delta = (d.values - contr_df_to_plot.sum(1).values)/d.values

print(f'There is a slight variation, with maximum value {max(delta.T)[0]*100:.2f}%, we will keep the full results for the figure.')
```

```
There is a slight variation, with maximum value 0.19%, we will keep the full results for the figure.
```

In [19]:

```
contr_perc = contr_df_to_plot.div(contr_df_to_plot.sum(1),axis=0)
ax = contr_perc.plot(kind='barh',
                     stacked=True,
                     figsize=(11,6),
                     mark_right=True,
                     cmap='tab20',
                     xlim=(0,1))

ax.legend(loc='upper center',
          bbox_to_anchor=(0.5, -0.1),
          fancybox=True,
          shadow=False,
          ncol=3)

ax_values = ax.twinx()
ax_values.set_ylim(ax.get_ylim())
ax_values.set_yticklabels(['']+utils.change_units(pd.Series(d.values[0], index=contr_df_to_plot.index),
                                            method_units))

ax.set_xticklabels(['0%', '20%', '40%', '60%', '80%', '100%'])

ax.set_title('Nuclear power, average PWR, 1 kWh high voltage to grid')
plt.setp(ax.patches, linewidth=0)

plt.tight_layout()

plt.savefig("output/paper_contr_analysis.svg",
            transparent=True,
            bbox_inches='tight',
            pad_inches=0)

plt.savefig("output/paper_contr_analysis_nuclear.png",
            transparent=True,
            bbox_inches='tight',
            pad_inches=0,
            dpi=300)
```

```
C:\Users\Gibon\AppData\Local\Temp\ipykernel_26960\2833994671.py:17: UserWarning: FixedFormatter should only be used together with FixedLocator
  ax_values.set_yticklabels(['']+utils.change_units(pd.Series(d.values[0], index=contr_df_to_plot.index),
C:\Users\Gibon\AppData\Local\Temp\ipykernel_26960\2833994671.py:20: UserWarning: FixedFormatter should only be used together with FixedLocator
  ax.set_xticklabels(['0%', '20%', '40%', '60%', '80%', '100%'])
```

In [20]:

```
elec_prod = findActivity('electricity production, nuclear, PWR, THEMIS', loc='GLO', db_name='UNEP_IRP_EUR')
```

In [21]:

```
multiLCAAlgebric([elec_prod,elec_prod_p], # The model 
    impacts, # Impacts
#     **{p.name:p.default for p in params._param_registry().all()}
)
```

```
Required param 'river_cooling' was missing, replacing by default value : 1.0
Required param 'integration_time_Rn222' was missing, replacing by default value : 80000
Required param 'efficiency' was missing, replacing by default value : 0.33
Required param 'enrichment_centr_elec' was missing, replacing by default value : 50
Required param 'mining_electricity_switch' was missing, replacing by default value : dieselgenerator
Required param 'tailings_Rn222' was missing, replacing by default value : 0.01951
Required param 'lifetime' was missing, replacing by default value : 60
Required param 'availability' was missing, replacing by default value : 0.9
Required param 'milling_electricity_switch' was missing, replacing by default value : dieselgenerator
Required param 'enrichment_diff_elec' was missing, replacing by default value : 2500
Required param 'fuel_fab_elec' was missing, replacing by default value : 36
Required param 'conversion_elec' was missing, replacing by default value : 11.8
Required param 'share_ISL' was missing, replacing by default value : 0.574
Required param 'construction_intensity' was missing, replacing by default value : 1
Required param 'enrichment_mix_switch' was missing, replacing by default value : centrifugation
Required param 'rate_enrichment' was missing, replacing by default value : 0.0415
Required param 'ore_grade' was missing, replacing by default value : 0.001544
Required param 'conversion_heat' was missing, replacing by default value : 26
```

Out[21]:

|  | climate change - global warming potential (GWP100)[kg CO2-Eq] | ecotoxicity: freshwater - comparative toxic unit for ecosystems (CTUe) [CTUe] | eutrophication: freshwater - fraction of nutrients reaching freshwater end compartment (P)[kg PO4-Eq] | human toxicity: carcinogenic - comparative toxic unit for human (CTUh) [CTUh] | human toxicity: non-carcinogenic - comparative toxic unit for human (CTUh) [CTUh] | ionising radiation: human health - human exposure efficiency relative to u235[kBq U235-Eq] | land use - soil quality index[dimensionless] | material resources: metals/minerals - abiotic depletion potential (ADP): elements (ultimate reserves)[kg Sb-Eq] | water use - user deprivation potential (deprivation-weighted water consumption)[m3 world eq. deprived] |
| --- | --- | --- | --- | --- | --- | --- | --- | --- | --- |
| electricity production, nuclear, PWR, THEMIS | 0.006558 | 0.516874 | 0.000007 | 1.490705e-11 | 6.618314e-10 | 0.013154 | 0.026211 | 4.137142e-07 | 0.103022 |
| electricity production, nuclear, PWR, parameterized | 0.006061 | 0.552907 | 0.000006 | 1.398592e-11 | 6.098642e-10 | 0.406450 | 0.026269 | 3.993655e-07 | 0.102558 |

In [22]:

```
list_parameters()
```

Out[22]:

| group | name | label | default | min | max | std | distrib | unit | db |
| --- | --- | --- | --- | --- | --- | --- | --- | --- | --- |
|  | availability | Availability of power plant | 0.9 | 0.65 | 1 |  | triangle | dimensionless | Nuclear\_DB |
|  | capacity | Nameplate capacity | 1000.0 |  |  |  | fixed | MWe | Nuclear\_DB |
|  | construction\_intensity | Intensity of construction inputs compared with default values | 1 | 0.5 | 2 |  | triangle | dimensionless | Nuclear\_DB |
|  | conversion\_elec | Conversion electricity input | 11.8 | 10.3 | 16.9 |  | triangle | kWh/kg U in UF6 | Nuclear\_DB |
|  | conversion\_heat | Conversion heat input | 26 | 26 | 665 |  | triangle | kWh/kg U in UF6 | Nuclear\_DB |
|  | efficiency | Efficiency of electricity generation | 0.33 | 0.3 | 0.34 |  | linear | dimensionless | Nuclear\_DB |
|  | enrichment\_centr\_elec | Enrichment electricity, centrifuge | 50 | 40 | 100 |  | triangle | kWh/SWU | Nuclear\_DB |
|  | enrichment\_centr\_share | Share of centrifugation in enrichment | 1 | 0 | 1 |  | triangle |  | Nuclear\_DB |
|  | enrichment\_diff\_elec | Enrichment electricity, diffusion | 2500 | 2400 | 3000 |  | triangle | kWh/SWU | Nuclear\_DB |
|  | enrichment\_mix\_switch | Enrichment technology | centrifugation | 0 | 2 |  |  |  | Nuclear\_DB |
|  | fuel\_fab\_elec | Fuel fabrication electricity | 36 | 36 | 50 |  | triangle | kWh/kg fuel | Nuclear\_DB |
|  | integration\_time\_Rn222 | Integration time for radiation from milling tailings | 80000 | 100 | 80000 |  | triangle | years | Nuclear\_DB |
|  | lifetime | Lifetime of plant and on-site equipment | 60 | 30 | 80 |  | triangle | year | Nuclear\_DB |
|  | milling\_electricity\_switch | Milling electricity, grid or diesel | dieselgenerator | 0 | 2 |  |  |  | Nuclear\_DB |
|  | mining\_electricity\_switch | Mining electricity, grid or diesel | dieselgenerator | 0 | 2 |  |  |  | Nuclear\_DB |
|  | ore\_grade | Uranium ore grade | 0.001544 | 1e-05 | 0.02 | 0.731586 | lognormal | dimensionless | Nuclear\_DB |
|  | rate\_enrichment | Uranium enrichment rate | 0.0415 | 0.03 | 0.05 |  | triangle |  | Nuclear\_DB |
|  | rate\_feed | Uranium feed rate | 0.0071 |  |  |  | fixed |  | Nuclear\_DB |
|  | rate\_tailings | Uranium enrichment tailings rate | 0.0022 |  |  |  | fixed |  | Nuclear\_DB |
|  | river\_cooling | River cooling | 1.0 |  |  |  |  |  | Nuclear\_DB |
|  | share\_ISL | Share of ISL, the rest is rescaled in proportion | 0.574 | 0 | 1 |  | linear | dimensionless | Nuclear\_DB |
|  | tailings\_Rn222 | Rn222 from tailings, in Bq/s | 0.01951 | 0.01 | 1 |  | triangle | Bq/s | Nuclear\_DB |

In [23]:

```
simplified = sobol_simplify_model(
    elec_prod_p, # The model
    impacts, # Impacts to consider
    n=2**10, # For large model, you may test other value and ensure ST and sum(S1) are close to 1.0 
    fixed_mode = FixedParamMode.MEDIAN, # We replace minor parameters by median by default,
    min_ratio=0.9, # Min ratio of variability to explain
    num_digits=3)
```

```
Generating samples ...
Transforming samples ...
```

```
Param 'rate_feed' is marked as FIXED, but passed in parameters : ignored
Param 'rate_tailings' is marked as FIXED, but passed in parameters : ignored
Param 'capacity' is marked as FIXED, but passed in parameters : ignored
```

```
Processing sobol for ('EF v3.0', 'climate change', 'global warming potential (GWP100)')
Processing sobol for ('EF v3.0', 'ecotoxicity: freshwater', 'comparative toxic unit for ecosystems (CTUe) ')
Processing sobol for ('EF v3.0', 'eutrophication: freshwater', 'fraction of nutrients reaching freshwater end compartment (P)')
Processing sobol for ('EF v3.0', 'human toxicity: carcinogenic', 'comparative toxic unit for human (CTUh) ')
Processing sobol for ('EF v3.0', 'human toxicity: non-carcinogenic', 'comparative toxic unit for human (CTUh) ')
Processing sobol for ('EF v3.0', 'ionising radiation: human health', 'human exposure efficiency relative to u235')
Processing sobol for ('EF v3.0', 'land use', 'soil quality index')
Processing sobol for ('EF v3.0', 'material resources: metals/minerals', 'abiotic depletion potential (ADP): elements (ultimate reserves)')
Processing sobol for ('EF v3.0', 'water use', 'user deprivation potential (deprivation-weighted water consumption)')
> Method :  climate change - global warming potential (GWP100)
S1:  1.080627092130487
S2:  -0.1581170560073769
ST:  1.1060895687806527
Selected params :  ['enrichment_mix_switch'] explains:  1.0572706561585683
```

$\displaystyle \frac{1.89 \cdot 10^{-5} EnrichmentMixSwitchCentrifugation \left(77.3 - 263.0 \log{\left(10 \right)}^{2}\right) + 1.89 \cdot 10^{-5} EnrichmentMixSwitchDiffusion \left(523.0 - 1.55 \cdot 10^{3} \log{\left(10 \right)}^{2}\right) - 0.00211 \log{\left(10 \right)}^{2}}{0.34 - 0.98 \log{\left(10 \right)}^{2}}$

```
> Method :  ecotoxicity: freshwater - comparative toxic unit for ecosystems (CTUe) 
S1:  1.018319615059631
S2:  -0.150971464312209
ST:  1.0484903917580728
Selected params :  ['share_ISL', 'enrichment_mix_switch'] explains:  0.982069600070169
```

$\displaystyle \frac{- 0.205 EnrichmentMixSwitchCentrifugation ShareIsl + 0.605 EnrichmentMixSwitchCentrifugation ShareIsl \log{\left(10 \right)}^{2} - 0.758 EnrichmentMixSwitchCentrifugation \log{\left(10 \right)}^{2} - 0.205 EnrichmentMixSwitchDiffusion ShareIsl + 0.605 EnrichmentMixSwitchDiffusion ShareIsl \log{\left(10 \right)}^{2} - 1.13 EnrichmentMixSwitchDiffusion \log{\left(10 \right)}^{2} + \begin{cases} 0.38 & \text{for}\: Diffusion = EnrichmentMixSwitch \\0.255 & \text{for}\: Centrifugation = EnrichmentMixSwitch \end{cases} - 0.162 \log{\left(10 \right)}^{2}}{0.334 - 0.98 \log{\left(10 \right)}^{2}}$

```
> Method :  eutrophication: freshwater - fraction of nutrients reaching freshwater end compartment (P)
S1:  1.0843537351528332
S2:  -0.11903156970628656
ST:  1.1036857353633802
Selected params :  ['enrichment_mix_switch'] explains:  1.0547344919919774
```

$\displaystyle \frac{1.88 \cdot 10^{-5} EnrichmentMixSwitchCentrifugation \left(0.0633 - 0.209 \log{\left(10 \right)}^{2}\right) + 1.88 \cdot 10^{-5} EnrichmentMixSwitchDiffusion \left(0.242 - 0.748 \log{\left(10 \right)}^{2}\right) - 2.17 \cdot 10^{-6} \log{\left(10 \right)}^{2} + 7.23 \cdot 10^{-7}}{0.326 - 0.98 \log{\left(10 \right)}^{2}}$

```
> Method :  human toxicity: carcinogenic - comparative toxic unit for human (CTUh) 
S1:  1.0355654665088787
S2:  -0.09130416955879586
ST:  1.050037794631562
Selected params :  ['enrichment_mix_switch', 'share_ISL'] explains:  0.9454198023787088
```

$\displaystyle \frac{- 6.76 \cdot 10^{-12} EnrichmentMixSwitchCentrifugation ShareIsl \log{\left(10 \right)}^{2} + 2.24 \cdot 10^{-12} EnrichmentMixSwitchCentrifugation ShareIsl - 5.68 \cdot 10^{-12} EnrichmentMixSwitchCentrifugation \log{\left(10 \right)}^{2} - 6.76 \cdot 10^{-12} EnrichmentMixSwitchDiffusion ShareIsl \log{\left(10 \right)}^{2} + 2.24 \cdot 10^{-12} EnrichmentMixSwitchDiffusion ShareIsl - 1.04 \cdot 10^{-11} EnrichmentMixSwitchDiffusion \log{\left(10 \right)}^{2} + \begin{cases} 3.36 \cdot 10^{-12} & \text{for}\: Diffusion = EnrichmentMixSwitch \\1.81 \cdot 10^{-12} & \text{for}\: Centrifugation = EnrichmentMixSwitch \end{cases} - 4.93 \cdot 10^{-12} \log{\left(10 \right)}^{2} + 1.62 \cdot 10^{-12}}{0.323 - 0.98 \log{\left(10 \right)}^{2}}$

```
> Method :  human toxicity: non-carcinogenic - comparative toxic unit for human (CTUh) 
S1:  1.0106610487443368
S2:  -0.046446820379251456
ST:  1.01523607509617
Selected params :  ['share_ISL', 'enrichment_mix_switch'] explains:  0.9930532030907689
```

$\displaystyle \frac{- 5.72 \cdot 10^{-10} EnrichmentMixSwitchCentrifugation ShareIsl \log{\left(10 \right)}^{2} + 1.95 \cdot 10^{-10} EnrichmentMixSwitchCentrifugation ShareIsl - 1.18 \cdot 10^{-10} EnrichmentMixSwitchCentrifugation \log{\left(10 \right)}^{2} - 5.72 \cdot 10^{-10} EnrichmentMixSwitchDiffusion ShareIsl \log{\left(10 \right)}^{2} + 1.95 \cdot 10^{-10} EnrichmentMixSwitchDiffusion ShareIsl - 2.75 \cdot 10^{-10} EnrichmentMixSwitchDiffusion \log{\left(10 \right)}^{2} + \begin{cases} 3.7 \cdot 10^{-11} & \text{for}\: Centrifugation = EnrichmentMixSwitch \\9.04 \cdot 10^{-11} & \text{for}\: Diffusion = EnrichmentMixSwitch \end{cases} - 1.76 \cdot 10^{-10} \log{\left(10 \right)}^{2} + 5.97 \cdot 10^{-11}}{0.333 - 0.98 \log{\left(10 \right)}^{2}}$

```
> Method :  ionising radiation: human health - human exposure efficiency relative to u235
S1:  0.8102252100052628
S2:  -0.06922015927733452
ST:  1.2338033474561014
Selected params :  ['tailings_Rn222', 'share_ISL', 'integration_time_Rn222', 'efficiency', 'rate_enrichment', 'mining_electricity_switch', 'lifetime', 'conversion_heat', 'construction_intensity', 'milling_electricity_switch', 'ore_grade', 'enrichment_centr_elec', 'fuel_fab_elec', 'enrichment_centr_share', 'river_cooling', 'conversion_elec', 'availability', 'enrichment_diff_elec', 'enrichment_mix_switch'] explains:  0.8102252100052632
```

$\displaystyle \begin{cases} \frac{\left(Availability Lifetime \left(202.0 EnrichmentMixSwitchCentrifugation \left(1.0 OreGrade \left(0.209 EnrichmentCentrElec + 0.702\right) \left(0.0723 \log{\left(100 OreGrade \right)}^{2} - 0.98 \log{\left(10 \right)}^{2}\right) \left(1.22 RateEnrichment + 0.0049 \cdot \left(2 RateEnrichment - 1\right) \log{\left(- \frac{RateEnrichment}{RateEnrichment - 1} \right)} - 0.0325\right) e^{9.2 \cdot 10^{-6} IntegrationTimeRn\_{222} + 0.707 OreGrade} - \left(RateEnrichment - 0.0022\right) \left(1.04 OreGrade ShareIsl \left(\left(- 2.39 \log{\left(100 OreGrade \right)}^{2} + 32.4 \log{\left(10 \right)}^{2}\right) e^{0.0485 OreGrade} + \left(\begin{cases} 0.854 & \text{for}\: Dieselgenerator = MiningElectricitySwitch \\6.99 & \text{for}\: Grid = MiningElectricitySwitch \end{cases}\right) \log{\left(10 \right)}^{2} + 0.404 \log{\left(10 \right)}^{2}\right) e^{9.2 \cdot 10^{-6} IntegrationTimeRn\_{222} + 0.658 OreGrade} - 1.0 OreGrade \left(0.0723 \log{\left(100 OreGrade \right)}^{2} - 0.98 \log{\left(10 \right)}^{2}\right) \left(0.245 ConversionElec + 0.00229 ConversionHeat + 33.0\right) e^{9.2 \cdot 10^{-6} IntegrationTimeRn\_{222} + 0.707 OreGrade} - \left(ShareIsl - 1\right) \left(0.577 OreGrade \left(\left(- 84.0 \log{\left(100 OreGrade \right)}^{2} + 1.14 \cdot 10^{3} \log{\left(10 \right)}^{2}\right) e^{0.176 OreGrade} + \left(\begin{cases} 10.1 & \text{for}\: Grid = MiningElectricitySwitch \\1.23 & \text{for}\: Dieselgenerator = MiningElectricitySwitch \end{cases}\right) \log{\left(10 \right)}^{2} + 0.582 \log{\left(10 \right)}^{2}\right) e^{9.2 \cdot 10^{-6} IntegrationTimeRn\_{222} + 0.482 OreGrade} + 0.465 OreGrade \left(\left(- 12.9 \log{\left(100 OreGrade \right)}^{2} + 175.0 \log{\left(10 \right)}^{2}\right) e^{0.482 OreGrade} + \left(\begin{cases} 1.06 & \text{for}\: Dieselgenerator = MiningElectricitySwitch \\8.71 & \text{for}\: Grid = MiningElectricitySwitch \end{cases}\right) \log{\left(10 \right)}^{2} + 0.503 \log{\left(10 \right)}^{2}\right) e^{9.2 \cdot 10^{-6} IntegrationTimeRn\_{222} + 0.176 OreGrade} - \left(0.0723 \log{\left(100 OreGrade \right)}^{2} - 0.98 \log{\left(10 \right)}^{2}\right) \left(- 4.08 \cdot 10^{6} OreGrade TailingsRn\_{222} \cdot \left(1 - e^{9.2 \cdot 10^{-6} IntegrationTimeRn\_{222}}\right) e^{0.658 OreGrade} + 4.3 OreGrade e^{9.2 \cdot 10^{-6} IntegrationTimeRn\_{222} + 0.658 OreGrade} + \left(\begin{cases} 0.00122 & \text{for}\: Grid = MillingElectricitySwitch \\0.000149 & \text{for}\: Dieselgenerator = MillingElectricitySwitch \end{cases} + 0.00109\right) e^{9.2 \cdot 10^{-6} IntegrationTimeRn\_{222} + 0.658 OreGrade}\right)\right) e^{0.0485 OreGrade}\right)\right) + 202.0 EnrichmentMixSwitchDiffusion \left(1.0 OreGrade \left(0.209 EnrichmentDiffElec + 28.1\right) \left(0.0723 \log{\left(100 OreGrade \right)}^{2} - 0.98 \log{\left(10 \right)}^{2}\right) \left(1.22 RateEnrichment + 0.0049 \cdot \left(2 RateEnrichment - 1\right) \log{\left(- \frac{RateEnrichment}{RateEnrichment - 1} \right)} - 0.0325\right) e^{9.2 \cdot 10^{-6} IntegrationTimeRn\_{222} + 0.707 OreGrade} - \left(RateEnrichment - 0.0022\right) \left(1.04 OreGrade ShareIsl \left(\left(- 2.39 \log{\left(100 OreGrade \right)}^{2} + 32.4 \log{\left(10 \right)}^{2}\right) e^{0.0485 OreGrade} + \left(\begin{cases} 0.854 & \text{for}\: Dieselgenerator = MiningElectricitySwitch \\6.99 & \text{for}\: Grid = MiningElectricitySwitch \end{cases}\right) \log{\left(10 \right)}^{2} + 0.404 \log{\left(10 \right)}^{2}\right) e^{9.2 \cdot 10^{-6} IntegrationTimeRn\_{222} + 0.658 OreGrade} - 1.0 OreGrade \left(0.0723 \log{\left(100 OreGrade \right)}^{2} - 0.98 \log{\left(10 \right)}^{2}\right) \left(0.245 ConversionElec + 0.00229 ConversionHeat + 33.0\right) e^{9.2 \cdot 10^{-6} IntegrationTimeRn\_{222} + 0.707 OreGrade} - \left(ShareIsl - 1\right) \left(0.577 OreGrade \left(\left(- 84.0 \log{\left(100 OreGrade \right)}^{2} + 1.14 \cdot 10^{3} \log{\left(10 \right)}^{2}\right) e^{0.176 OreGrade} + \left(\begin{cases} 10.1 & \text{for}\: Grid = MiningElectricitySwitch \\1.23 & \text{for}\: Dieselgenerator = MiningElectricitySwitch \end{cases}\right) \log{\left(10 \right)}^{2} + 0.582 \log{\left(10 \right)}^{2}\right) e^{9.2 \cdot 10^{-6} IntegrationTimeRn\_{222} + 0.482 OreGrade} + 0.465 OreGrade \left(\left(- 12.9 \log{\left(100 OreGrade \right)}^{2} + 175.0 \log{\left(10 \right)}^{2}\right) e^{0.482 OreGrade} + \left(\begin{cases} 1.06 & \text{for}\: Dieselgenerator = MiningElectricitySwitch \\8.71 & \text{for}\: Grid = MiningElectricitySwitch \end{cases}\right) \log{\left(10 \right)}^{2} + 0.503 \log{\left(10 \right)}^{2}\right) e^{9.2 \cdot 10^{-6} IntegrationTimeRn\_{222} + 0.176 OreGrade} - \left(0.0723 \log{\left(100 OreGrade \right)}^{2} - 0.98 \log{\left(10 \right)}^{2}\right) \left(- 4.08 \cdot 10^{6} OreGrade TailingsRn\_{222} \cdot \left(1 - e^{9.2 \cdot 10^{-6} IntegrationTimeRn\_{222}}\right) e^{0.658 OreGrade} + 4.3 OreGrade e^{9.2 \cdot 10^{-6} IntegrationTimeRn\_{222} + 0.658 OreGrade} + \left(\begin{cases} 0.00122 & \text{for}\: Grid = MillingElectricitySwitch \\0.000149 & \text{for}\: Dieselgenerator = MillingElectricitySwitch \end{cases} + 0.00109\right) e^{9.2 \cdot 10^{-6} IntegrationTimeRn\_{222} + 0.658 OreGrade}\right)\right) e^{0.0485 OreGrade}\right)\right) + 1.0 OreGrade \left(0.121 FuelFabElec + 24.1\right) \left(0.0723 \log{\left(100 OreGrade \right)}^{2} - 0.98 \log{\left(10 \right)}^{2}\right) e^{9.2 \cdot 10^{-6} IntegrationTimeRn\_{222} + 0.707 OreGrade}\right) + Efficiency OreGrade \left(1.09 \cdot 10^{3} RateEnrichment - 0.6\right) \left(0.0723 \log{\left(100 OreGrade \right)}^{2} - 0.98 \log{\left(10 \right)}^{2}\right) \left(21.1 Availability Lifetime + 416.0 ConstructionIntensity + 1.58\right) e^{9.2 \cdot 10^{-6} IntegrationTimeRn\_{222} + 0.707 OreGrade}\right) \left(5.08 \cdot 10^{-5} RateEnrichment + 2.04 \cdot 10^{-7} \cdot \left(2 RateEnrichment - 1\right) \log{\left(- \frac{RateEnrichment}{RateEnrichment - 1} \right)} - 1.36 \cdot 10^{-6}\right) e^{- 9.2 \cdot 10^{-6} IntegrationTimeRn\_{222} - 0.707 OreGrade}}{Availability Efficiency Lifetime OreGrade \left(1.09 \cdot 10^{3} RateEnrichment - 0.6\right) \left(0.0723 \log{\left(100 OreGrade \right)}^{2} - 0.98 \log{\left(10 \right)}^{2}\right) \left(1.22 RateEnrichment + 0.0049 \cdot \left(2 RateEnrichment - 1\right) \log{\left(- \frac{RateEnrichment}{RateEnrichment - 1} \right)} - 0.0325\right)} & \text{for}\: OreGrade < 0.01 \\\frac{\left(0.00088 Availability Efficiency Lifetime OreGrade \left(1.09 \cdot 10^{3} RateEnrichment - 0.6\right) e^{9.2 \cdot 10^{-6} IntegrationTimeRn\_{222} + 0.707 OreGrade} + 4.17 \cdot 10^{-5} Availability Lifetime \left(202.0 EnrichmentMixSwitchCentrifugation \left(1.0 OreGrade \left(0.209 EnrichmentCentrElec + 0.702\right) \left(1.22 RateEnrichment + 0.0049 \cdot \left(2 RateEnrichment - 1\right) \log{\left(- \frac{RateEnrichment}{RateEnrichment - 1} \right)} - 0.0325\right) e^{9.2 \cdot 10^{-6} IntegrationTimeRn\_{222} + 0.707 OreGrade} + \left(RateEnrichment - 0.0022\right) \left(1.04 OreGrade ShareIsl \left(\begin{cases} 0.872 & \text{for}\: Dieselgenerator = MiningElectricitySwitch \\7.13 & \text{for}\: Grid = MiningElectricitySwitch \end{cases} + 33.1 e^{0.0485 OreGrade} + 0.412\right) e^{9.2 \cdot 10^{-6} IntegrationTimeRn\_{222} + 0.658 OreGrade} + 1.0 OreGrade \left(0.245 ConversionElec + 0.00229 ConversionHeat + 33.0\right) e^{9.2 \cdot 10^{-6} IntegrationTimeRn\_{222} + 0.707 OreGrade} - \left(ShareIsl - 1\right) \left(- 4.08 \cdot 10^{6} OreGrade TailingsRn\_{222} \cdot \left(1 - e^{9.2 \cdot 10^{-6} IntegrationTimeRn\_{222}}\right) e^{0.658 OreGrade} + 0.577 OreGrade \left(\begin{cases} 1.26 & \text{for}\: Dieselgenerator = MiningElectricitySwitch \\10.3 & \text{for}\: Grid = MiningElectricitySwitch \end{cases} + 1.16 \cdot 10^{3} e^{0.176 OreGrade} + 0.594\right) e^{9.2 \cdot 10^{-6} IntegrationTimeRn\_{222} + 0.482 OreGrade} + 0.465 OreGrade \left(\begin{cases} 8.88 & \text{for}\: Grid = MiningElectricitySwitch \\1.09 & \text{for}\: Dieselgenerator = MiningElectricitySwitch \end{cases} + 179.0 e^{0.482 OreGrade} + 0.514\right) e^{9.2 \cdot 10^{-6} IntegrationTimeRn\_{222} + 0.176 OreGrade} + 4.3 OreGrade e^{9.2 \cdot 10^{-6} IntegrationTimeRn\_{222} + 0.658 OreGrade} + \left(\begin{cases} 0.00122 & \text{for}\: Grid = MillingElectricitySwitch \\0.000149 & \text{for}\: Dieselgenerator = MillingElectricitySwitch \end{cases} + 0.00109\right) e^{9.2 \cdot 10^{-6} IntegrationTimeRn\_{222} + 0.658 OreGrade}\right) e^{0.0485 OreGrade}\right)\right) + 202.0 EnrichmentMixSwitchDiffusion \left(1.0 OreGrade \left(0.209 EnrichmentDiffElec + 28.1\right) \left(1.22 RateEnrichment + 0.0049 \cdot \left(2 RateEnrichment - 1\right) \log{\left(- \frac{RateEnrichment}{RateEnrichment - 1} \right)} - 0.0325\right) e^{9.2 \cdot 10^{-6} IntegrationTimeRn\_{222} + 0.707 OreGrade} + \left(RateEnrichment - 0.0022\right) \left(1.04 OreGrade ShareIsl \left(\begin{cases} 0.872 & \text{for}\: Dieselgenerator = MiningElectricitySwitch \\7.13 & \text{for}\: Grid = MiningElectricitySwitch \end{cases} + 33.1 e^{0.0485 OreGrade} + 0.412\right) e^{9.2 \cdot 10^{-6} IntegrationTimeRn\_{222} + 0.658 OreGrade} + 1.0 OreGrade \left(0.245 ConversionElec + 0.00229 ConversionHeat + 33.0\right) e^{9.2 \cdot 10^{-6} IntegrationTimeRn\_{222} + 0.707 OreGrade} - \left(ShareIsl - 1\right) \left(- 4.08 \cdot 10^{6} OreGrade TailingsRn\_{222} \cdot \left(1 - e^{9.2 \cdot 10^{-6} IntegrationTimeRn\_{222}}\right) e^{0.658 OreGrade} + 0.577 OreGrade \left(\begin{cases} 1.26 & \text{for}\: Dieselgenerator = MiningElectricitySwitch \\10.3 & \text{for}\: Grid = MiningElectricitySwitch \end{cases} + 1.16 \cdot 10^{3} e^{0.176 OreGrade} + 0.594\right) e^{9.2 \cdot 10^{-6} IntegrationTimeRn\_{222} + 0.482 OreGrade} + 0.465 OreGrade \left(\begin{cases} 8.88 & \text{for}\: Grid = MiningElectricitySwitch \\1.09 & \text{for}\: Dieselgenerator = MiningElectricitySwitch \end{cases} + 179.0 e^{0.482 OreGrade} + 0.514\right) e^{9.2 \cdot 10^{-6} IntegrationTimeRn\_{222} + 0.176 OreGrade} + 4.3 OreGrade e^{9.2 \cdot 10^{-6} IntegrationTimeRn\_{222} + 0.658 OreGrade} + \left(\begin{cases} 0.00122 & \text{for}\: Grid = MillingElectricitySwitch \\0.000149 & \text{for}\: Dieselgenerator = MillingElectricitySwitch \end{cases} + 0.00109\right) e^{9.2 \cdot 10^{-6} IntegrationTimeRn\_{222} + 0.658 OreGrade}\right) e^{0.0485 OreGrade}\right)\right) + 1.0 OreGrade \left(0.121 FuelFabElec + 24.1\right) e^{9.2 \cdot 10^{-6} IntegrationTimeRn\_{222} + 0.707 OreGrade}\right) + 4.17 \cdot 10^{-5} Efficiency OreGrade \left(416.0 ConstructionIntensity + 1.58\right) \left(1.09 \cdot 10^{3} RateEnrichment - 0.6\right) e^{9.2 \cdot 10^{-6} IntegrationTimeRn\_{222} + 0.707 OreGrade}\right) e^{- 9.2 \cdot 10^{-6} IntegrationTimeRn\_{222} - 0.707 OreGrade}}{Availability Efficiency Lifetime OreGrade \left(1.09 \cdot 10^{3} RateEnrichment - 0.6\right)} & \text{otherwise} \end{cases}$

```
> Method :  land use - soil quality index
S1:  1.0701700372428362
S2:  -0.1871872666676156
ST:  1.100916986042885
Selected params :  ['enrichment_mix_switch'] explains:  0.9836540729696489
```

$\displaystyle \frac{1.89 \cdot 10^{-5} EnrichmentMixSwitchCentrifugation \left(264.0 - 845.0 \log{\left(10 \right)}^{2}\right) + 1.89 \cdot 10^{-5} EnrichmentMixSwitchDiffusion \left(825.0 - 2.53 \cdot 10^{3} \log{\left(10 \right)}^{2}\right) - 0.0121 \log{\left(10 \right)}^{2} + 0.00402}{0.326 - 0.98 \log{\left(10 \right)}^{2}}$

```
> Method :  material resources: metals/minerals - abiotic depletion potential (ADP): elements (ultimate reserves)
S1:  0.9976240730246669
S2:  0.04529021092675637
ST:  1.0083906077658438
Selected params :  ['share_ISL', 'construction_intensity', 'enrichment_mix_switch'] explains:  0.9299085090064682
```

$\displaystyle \frac{- 4.15 \cdot 10^{-8} ConstructionIntensity \log{\left(10 \right)}^{2} + 1.35 \cdot 10^{-8} ConstructionIntensity - 1.47 \cdot 10^{-7} EnrichmentMixSwitchCentrifugation ShareIsl \log{\left(10 \right)}^{2} + 4.81 \cdot 10^{-8} EnrichmentMixSwitchCentrifugation ShareIsl - 5.16 \cdot 10^{-8} EnrichmentMixSwitchCentrifugation \log{\left(10 \right)}^{2} - 1.47 \cdot 10^{-7} EnrichmentMixSwitchDiffusion ShareIsl \log{\left(10 \right)}^{2} + 4.81 \cdot 10^{-8} EnrichmentMixSwitchDiffusion ShareIsl - 7.63 \cdot 10^{-8} EnrichmentMixSwitchDiffusion \log{\left(10 \right)}^{2} + \begin{cases} 1.65 \cdot 10^{-8} & \text{for}\: Centrifugation = EnrichmentMixSwitch \\2.46 \cdot 10^{-8} & \text{for}\: Diffusion = EnrichmentMixSwitch \end{cases} - 2.22 \cdot 10^{-7} \log{\left(10 \right)}^{2} + 7.26 \cdot 10^{-8}}{0.32 - 0.98 \log{\left(10 \right)}^{2}}$

```
> Method :  water use - user deprivation potential (deprivation-weighted water consumption)
S1:  1.0010039477854236
S2:  -0.0021225277494086284
ST:  1.000072635810356
Selected params :  ['river_cooling'] explains:  0.9964871331523276
```

$\displaystyle 0.0988 RiverCooling + 0.00547$

In [24]:

```
# Setting 100% centrifugation
enrichment_mix_switch.distrib = DistributionType.FIXED
enrichment_mix_switch.default = 'centrifugation'
```

In [25]:

```
simplified_centr = sobol_simplify_model(
    elec_prod_p, # The model
    impacts, # Impacts to consider
    n=2**10, # For large model, you may test other value and ensure ST and sum(S1) are close to 1.0 
    fixed_mode = FixedParamMode.MEDIAN, # We replace minor parameters by median by default,
    min_ratio=0.8, # Min ratio of variability to explain
    num_digits=3)
```

```
Generating samples ...
Transforming samples ...
```

```
Param 'rate_feed' is marked as FIXED, but passed in parameters : ignored
Param 'rate_tailings' is marked as FIXED, but passed in parameters : ignored
Param 'capacity' is marked as FIXED, but passed in parameters : ignored
Param 'enrichment_mix_switch' is marked as FIXED, but passed in parameters : ignored
```

```
Processing sobol for ('EF v3.0', 'climate change', 'global warming potential (GWP100)')
Processing sobol for ('EF v3.0', 'ecotoxicity: freshwater', 'comparative toxic unit for ecosystems (CTUe) ')
Processing sobol for ('EF v3.0', 'eutrophication: freshwater', 'fraction of nutrients reaching freshwater end compartment (P)')
Processing sobol for ('EF v3.0', 'human toxicity: carcinogenic', 'comparative toxic unit for human (CTUh) ')
Processing sobol for ('EF v3.0', 'human toxicity: non-carcinogenic', 'comparative toxic unit for human (CTUh) ')
Processing sobol for ('EF v3.0', 'ionising radiation: human health', 'human exposure efficiency relative to u235')
Processing sobol for ('EF v3.0', 'land use', 'soil quality index')
Processing sobol for ('EF v3.0', 'material resources: metals/minerals', 'abiotic depletion potential (ADP): elements (ultimate reserves)')
Processing sobol for ('EF v3.0', 'water use', 'user deprivation potential (deprivation-weighted water consumption)')
> Method :  climate change - global warming potential (GWP100)
S1:  0.8457845372803842
S2:  0.5901906107820551
ST:  1.213489960453173
Selected params :  ['ore_grade', 'share_ISL', 'construction_intensity', 'lifetime', 'efficiency'] explains:  0.80681332669308
```

$\displaystyle \begin{cases} \frac{1.0 \left(Efficiency OreGrade \left(0.0723 \log{\left(100 OreGrade \right)}^{2} - 0.98 \log{\left(10 \right)}^{2}\right) \left(0.0456 ConstructionIntensity + 0.00109 Lifetime + 0.00253\right) e^{0.707 OreGrade} + Lifetime \left(7.72 \cdot 10^{-6} OreGrade ShareIsl \left(\left(5.12 \log{\left(100 OreGrade \right)}^{2} - 69.4 \log{\left(10 \right)}^{2}\right) e^{0.0485 OreGrade} - 26.7 \log{\left(10 \right)}^{2}\right) e^{0.658 OreGrade} + 0.000552 OreGrade \left(0.0723 \log{\left(100 OreGrade \right)}^{2} - 0.98 \log{\left(10 \right)}^{2}\right) e^{0.707 OreGrade} - \left(ShareIsl - 1\right) \left(3.44 \cdot 10^{-6} OreGrade \left(\left(0.563 \log{\left(100 OreGrade \right)}^{2} - 7.63 \log{\left(10 \right)}^{2}\right) e^{0.482 OreGrade} - 33.2 \log{\left(10 \right)}^{2}\right) e^{0.176 OreGrade} + 4.28 \cdot 10^{-6} OreGrade \left(\left(5.29 \log{\left(100 OreGrade \right)}^{2} - 71.7 \log{\left(10 \right)}^{2}\right) e^{0.176 OreGrade} - 38.4 \log{\left(10 \right)}^{2}\right) e^{0.482 OreGrade} + \left(0.000201 OreGrade + 7.04 \cdot 10^{-7}\right) \left(0.0723 \log{\left(100 OreGrade \right)}^{2} - 0.98 \log{\left(10 \right)}^{2}\right) e^{0.658 OreGrade}\right) e^{0.0485 OreGrade}\right)\right) e^{- 0.707 OreGrade}}{Efficiency Lifetime OreGrade \left(0.0723 \log{\left(100 OreGrade \right)}^{2} - 0.98 \log{\left(10 \right)}^{2}\right)} & \text{for}\: OreGrade < 0.01 \\\frac{0.0456 ConstructionIntensity}{Lifetime} + 0.00109 + \frac{0.00253}{Lifetime} + \frac{5.77 \cdot 10^{-6} ShareIsl}{Efficiency} - \frac{0.000117 ShareIsl e^{- 0.482 OreGrade}}{Efficiency} - \frac{0.000168 ShareIsl e^{- 0.176 OreGrade}}{Efficiency} + \frac{0.00021 ShareIsl e^{- 0.0485 OreGrade}}{Efficiency} + \frac{0.00109}{Efficiency} + \frac{0.000117 e^{- 0.482 OreGrade}}{Efficiency} + \frac{0.000168 e^{- 0.176 OreGrade}}{Efficiency} - \frac{7.04 \cdot 10^{-7} ShareIsl}{Efficiency OreGrade} + \frac{7.04 \cdot 10^{-7}}{Efficiency OreGrade} & \text{otherwise} \end{cases}$

```
> Method :  ecotoxicity: freshwater - comparative toxic unit for ecosystems (CTUe) 
S1:  0.9899569225062979
S2:  0.11005458948800673
ST:  1.0214702479906157
Selected params :  ['share_ISL'] explains:  0.9429316209668863
```

$\displaystyle \frac{- 0.202 ShareIsl + 0.604 ShareIsl \log{\left(10 \right)}^{2} - 0.918 \log{\left(10 \right)}^{2} + 0.306}{0.33 - 0.98 \log{\left(10 \right)}^{2}}$

```
> Method :  eutrophication: freshwater - fraction of nutrients reaching freshwater end compartment (P)
S1:  0.9119143142107945
S2:  0.19740843863502824
ST:  1.082578513081035
Selected params :  ['mining_electricity_switch', 'share_ISL', 'ore_grade', 'construction_intensity', 'lifetime'] explains:  0.8460660035928778
```

$\displaystyle \begin{cases} \frac{\left(- 2.41 \cdot 10^{-5} Lifetime OreGrade ShareIsl \left(\left(- 0.0108 \log{\left(100 OreGrade \right)}^{2} + 0.147 \log{\left(10 \right)}^{2}\right) e^{0.0485 OreGrade} + \left(\begin{cases} 0.000384 & \text{for}\: Dieselgenerator = MiningElectricitySwitch \\0.0241 & \text{for}\: Grid = MiningElectricitySwitch \end{cases}\right) \log{\left(10 \right)}^{2} + 0.00028 \log{\left(10 \right)}^{2}\right) e^{0.658 OreGrade} - 1.0 Lifetime \left(ShareIsl - 1\right) \left(- 1.33 \cdot 10^{-5} OreGrade \left(\left(- 0.00332 \log{\left(100 OreGrade \right)}^{2} + 0.045 \log{\left(10 \right)}^{2}\right) e^{0.176 OreGrade} + \left(\begin{cases} 0.0347 & \text{for}\: Grid = MiningElectricitySwitch \\0.000554 & \text{for}\: Dieselgenerator = MiningElectricitySwitch \end{cases}\right) \log{\left(10 \right)}^{2} + 0.000403 \log{\left(10 \right)}^{2}\right) e^{0.482 OreGrade} - 1.07 \cdot 10^{-5} OreGrade \left(\left(- 0.000127 \log{\left(100 OreGrade \right)}^{2} + 0.00172 \log{\left(10 \right)}^{2}\right) e^{0.482 OreGrade} + \left(\begin{cases} 0.000479 & \text{for}\: Dieselgenerator = MiningElectricitySwitch \\0.03 & \text{for}\: Grid = MiningElectricitySwitch \end{cases}\right) \log{\left(10 \right)}^{2} + 0.000349 \log{\left(10 \right)}^{2}\right) e^{0.176 OreGrade} + \left(1.05 \cdot 10^{-6} OreGrade + 6.3 \cdot 10^{-10}\right) \left(0.0723 \log{\left(100 OreGrade \right)}^{2} - 0.98 \log{\left(10 \right)}^{2}\right) e^{0.658 OreGrade}\right) e^{0.0485 OreGrade} + 1.0 OreGrade \left(0.0723 \log{\left(100 OreGrade \right)}^{2} - 0.98 \log{\left(10 \right)}^{2}\right) \left(3.91 \cdot 10^{-5} ConstructionIntensity + 2.16 \cdot 10^{-6} Lifetime + 6.36 \cdot 10^{-7}\right) e^{0.707 OreGrade}\right) e^{- 0.707 OreGrade}}{Lifetime OreGrade \left(0.0723 \log{\left(100 OreGrade \right)}^{2} - 0.98 \log{\left(10 \right)}^{2}\right)} & \text{for}\: OreGrade < 0.01 \\\frac{\left(2.41 \cdot 10^{-5} Lifetime OreGrade ShareIsl \left(\begin{cases} 0.0246 & \text{for}\: Grid = MiningElectricitySwitch \\0.000392 & \text{for}\: Dieselgenerator = MiningElectricitySwitch \end{cases} + 0.15 e^{0.0485 OreGrade} + 0.000286\right) e^{0.658 OreGrade} + 2.16 \cdot 10^{-6} Lifetime OreGrade e^{0.707 OreGrade} - 1.0 Lifetime \left(ShareIsl - 1\right) \left(1.33 \cdot 10^{-5} OreGrade \left(\begin{cases} 0.000565 & \text{for}\: Dieselgenerator = MiningElectricitySwitch \\0.0354 & \text{for}\: Grid = MiningElectricitySwitch \end{cases} + 0.0459 e^{0.176 OreGrade} + 0.000412\right) e^{0.482 OreGrade} + 1.07 \cdot 10^{-5} OreGrade \left(\begin{cases} 0.0306 & \text{for}\: Grid = MiningElectricitySwitch \\0.000488 & \text{for}\: Dieselgenerator = MiningElectricitySwitch \end{cases} + 0.00176 e^{0.482 OreGrade} + 0.000356\right) e^{0.176 OreGrade} + 1.05 \cdot 10^{-6} OreGrade e^{0.658 OreGrade} + 6.3 \cdot 10^{-10} e^{0.658 OreGrade}\right) e^{0.0485 OreGrade} + 1.0 OreGrade \left(3.91 \cdot 10^{-5} ConstructionIntensity + 6.36 \cdot 10^{-7}\right) e^{0.707 OreGrade}\right) e^{- 0.707 OreGrade}}{Lifetime OreGrade} & \text{otherwise} \end{cases}$

```
> Method :  human toxicity: carcinogenic - comparative toxic unit for human (CTUh) 
S1:  0.9839480935220318
S2:  0.026805044188420453
ST:  1.005932688100229
Selected params :  ['share_ISL'] explains:  0.8301978045121593
```

$\displaystyle \frac{- 6.76 \cdot 10^{-12} ShareIsl \log{\left(10 \right)}^{2} + 2.19 \cdot 10^{-12} ShareIsl - 1.05 \cdot 10^{-11} \log{\left(10 \right)}^{2} + 3.33 \cdot 10^{-12}}{0.315 - 0.98 \log{\left(10 \right)}^{2}}$

```
> Method :  human toxicity: non-carcinogenic - comparative toxic unit for human (CTUh) 
S1:  0.9958024964818011
S2:  -0.0033415452377129574
ST:  1.0019015153025153
Selected params :  ['share_ISL'] explains:  0.9774575971634435
```

$\displaystyle \frac{- 5.73 \cdot 10^{-10} ShareIsl \log{\left(10 \right)}^{2} + 1.91 \cdot 10^{-10} ShareIsl - 2.93 \cdot 10^{-10} \log{\left(10 \right)}^{2} + 9.44 \cdot 10^{-11}}{0.325 - 0.98 \log{\left(10 \right)}^{2}}$

```
> Method :  ionising radiation: human health - human exposure efficiency relative to u235
S1:  0.8006192102045924
S2:  -0.047902590766627826
ST:  1.2329778147800958
Selected params :  ['tailings_Rn222', 'share_ISL', 'integration_time_Rn222'] explains:  0.8018948143803729
```

$\displaystyle 94.5 TailingsRn\_{222} \cdot \left(1 - e^{9.2 \cdot 10^{-6} IntegrationTimeRn\_{222}}\right) \left(ShareIsl - 1\right) e^{- 9.2 \cdot 10^{-6} IntegrationTimeRn\_{222}}$

```
> Method :  land use - soil quality index
S1:  0.9350990660317854
S2:  0.28128918334536485
ST:  1.096112247233266
Selected params :  ['share_ISL', 'ore_grade', 'lifetime'] explains:  0.8221959582500847
```

$\displaystyle \begin{cases} \frac{\left(2.42 \cdot 10^{-5} Lifetime OreGrade ShareIsl \left(\left(23.7 \log{\left(100 OreGrade \right)}^{2} - 322.0 \log{\left(10 \right)}^{2}\right) e^{0.0485 OreGrade} - 33.3 \log{\left(10 \right)}^{2}\right) e^{0.658 OreGrade} - 1.0 Lifetime \left(ShareIsl - 1\right) \left(1.34 \cdot 10^{-5} OreGrade \left(\left(6.93 \log{\left(100 OreGrade \right)}^{2} - 93.9 \log{\left(10 \right)}^{2}\right) e^{0.176 OreGrade} - 48.0 \log{\left(10 \right)}^{2}\right) e^{0.482 OreGrade} + 1.08 \cdot 10^{-5} OreGrade \left(\left(18.2 \log{\left(100 OreGrade \right)}^{2} - 247.0 \log{\left(10 \right)}^{2}\right) e^{0.482 OreGrade} - 41.5 \log{\left(10 \right)}^{2}\right) e^{0.176 OreGrade} + \left(0.00941 OreGrade + 3.7 \cdot 10^{-6}\right) \left(0.0723 \log{\left(100 OreGrade \right)}^{2} - 0.98 \log{\left(10 \right)}^{2}\right) e^{0.658 OreGrade}\right) e^{0.0485 OreGrade} + 1.0 OreGrade \left(0.00863 Lifetime + 0.393\right) \left(0.0723 \log{\left(100 OreGrade \right)}^{2} - 0.98 \log{\left(10 \right)}^{2}\right) e^{0.707 OreGrade}\right) e^{- 0.707 OreGrade}}{Lifetime OreGrade \left(0.0723 \log{\left(100 OreGrade \right)}^{2} - 0.98 \log{\left(10 \right)}^{2}\right)} & \text{for}\: OreGrade < 0.01 \\- 0.00547 ShareIsl - 0.000456 ShareIsl e^{- 0.482 OreGrade} - 0.000656 ShareIsl e^{- 0.176 OreGrade} + 0.000821 ShareIsl e^{- 0.0485 OreGrade} + 0.022 + 0.000456 e^{- 0.482 OreGrade} + 0.000656 e^{- 0.176 OreGrade} - \frac{3.7 \cdot 10^{-6} ShareIsl}{OreGrade} + \frac{3.7 \cdot 10^{-6}}{OreGrade} + \frac{0.393}{Lifetime} & \text{otherwise} \end{cases}$

```
> Method :  material resources: metals/minerals - abiotic depletion potential (ADP): elements (ultimate reserves)
S1:  0.9890516735528401
S2:  0.04398900098376855
ST:  1.0019956611624223
Selected params :  ['share_ISL'] explains:  0.8324545291860309
```

$\displaystyle \frac{- 1.47 \cdot 10^{-7} ShareIsl \log{\left(10 \right)}^{2} + 5.05 \cdot 10^{-8} ShareIsl - 3.2 \cdot 10^{-7} \log{\left(10 \right)}^{2} + 1.09 \cdot 10^{-7}}{0.337 - 0.98 \log{\left(10 \right)}^{2}}$

```
> Method :  water use - user deprivation potential (deprivation-weighted water consumption)
S1:  1.0000647205188684
S2:  -0.003987216161139988
ST:  0.9999778373812227
Selected params :  ['river_cooling'] explains:  0.9998468885658456
```

$\displaystyle 0.0988 RiverCooling + 0.00396$

In [166]:

```
for i,s in zip(impacts,simplified):
    print(i)
    display(simplify(s.expr.evalf(n=4)))
    print('-'*80)
```

```
('EF v3.0', 'climate change', 'global warming potential (GWP100)')
```

$\displaystyle 0.005094 enrichment\_{mix switch centrifugation} + 0.02999 enrichment\_{mix switch diffusion} + 0.00234$

```
--------------------------------------------------------------------------------
('EF v3.0', 'ecotoxicity: freshwater', 'comparative toxic unit for ecosystems (CTUe) ')
```

$\displaystyle - 0.6189 enrichment\_{mix switch centrifugation} share\_{ISL} + 0.7757 enrichment\_{mix switch centrifugation} - 0.6189 enrichment\_{mix switch diffusion} share\_{ISL} + 1.151 enrichment\_{mix switch diffusion} + 0.1764$

```
--------------------------------------------------------------------------------
('EF v3.0', 'eutrophication: freshwater', 'fraction of nutrients reaching freshwater end compartment (P)')
```

$\displaystyle 4.055 \cdot 10^{-6} enrichment\_{mix switch centrifugation} + 1.44 \cdot 10^{-5} enrichment\_{mix switch diffusion} + 2.215 \cdot 10^{-6}$

```
--------------------------------------------------------------------------------
('EF v3.0', 'human toxicity: carcinogenic', 'comparative toxic unit for human (CTUh) ')
```

$\displaystyle 6.898 \cdot 10^{-12} enrichment\_{mix switch centrifugation} share\_{ISL} + 5.841 \cdot 10^{-12} enrichment\_{mix switch centrifugation} + 6.898 \cdot 10^{-12} enrichment\_{mix switch diffusion} share\_{ISL} + 1.066 \cdot 10^{-11} enrichment\_{mix switch diffusion} + 5.046 \cdot 10^{-12}$

```
--------------------------------------------------------------------------------
('EF v3.0', 'human toxicity: non-carcinogenic', 'comparative toxic unit for human (CTUh) ')
```

$\displaystyle 5.865 \cdot 10^{-10} enrichment\_{mix switch centrifugation} share\_{ISL} + 1.204 \cdot 10^{-10} enrichment\_{mix switch centrifugation} + 5.865 \cdot 10^{-10} enrichment\_{mix switch diffusion} share\_{ISL} + 2.825 \cdot 10^{-10} enrichment\_{mix switch diffusion} + 1.794 \cdot 10^{-10}$

```
--------------------------------------------------------------------------------
('EF v3.0', 'ionising radiation: human health', 'human exposure efficiency relative to u235')
```

$\displaystyle \begin{cases} \frac{\left(availability lifetime \left(202.0 enrichment\_{mix switch centrifugation} \left(1.0 ore\_{grade} \left(0.209 enrichment\_{centr elec} + 0.702\right) \left(0.0723 \log{\left(100 ore\_{grade} \right)}^{2} - 5.196\right) \left(1.22 rate\_{enrichment} + 0.0049 \cdot \left(2.0 rate\_{enrichment} - 1.0\right) \log{\left(- \frac{rate\_{enrichment}}{rate\_{enrichment} - 1} \right)} - 0.03252\right) e^{9.2 \cdot 10^{-6} integration\_{time Rn222} + 0.707 ore\_{grade}} + \left(rate\_{enrichment} - 0.0022\right) \left(- 1.042 ore\_{grade} share\_{ISL} \left(4.53 mining\_{electricity switch dieselgenerator} + 37.06 mining\_{electricity switch grid} + \left(172.0 - 2.393 \log{\left(100 ore\_{grade} \right)}^{2}\right) e^{0.0485 ore\_{grade}} + 2.143\right) e^{9.2 \cdot 10^{-6} integration\_{time Rn222} + 0.658 ore\_{grade}} + 1.0 ore\_{grade} \left(0.0723 \log{\left(100 ore\_{grade} \right)}^{2} - 5.196\right) \left(0.2454 conversion\_{elec} + 0.002289 conversion\_{heat} + 33.02\right) e^{9.2 \cdot 10^{-6} integration\_{time Rn222} + 0.707 ore\_{grade}} + \left(share\_{ISL} - 1.0\right) \left(0.4648 ore\_{grade} \left(5.641 mining\_{electricity switch dieselgenerator} + 46.15 mining\_{electricity switch grid} + \left(929.3 - 12.93 \log{\left(100 ore\_{grade} \right)}^{2}\right) e^{0.482 ore\_{grade}} + 2.668\right) e^{9.2 \cdot 10^{-6} integration\_{time Rn222} + 0.176 ore\_{grade}} + 0.5774 ore\_{grade} \left(6.526 mining\_{electricity switch dieselgenerator} + 53.39 mining\_{electricity switch grid} + \left(6035.0 - 83.98 \log{\left(100 ore\_{grade} \right)}^{2}\right) e^{0.176 ore\_{grade}} + 3.087\right) e^{9.2 \cdot 10^{-6} integration\_{time Rn222} + 0.482 ore\_{grade}} - \left(0.0723 \log{\left(100 ore\_{grade} \right)}^{2} - 5.196\right) \left(4.078 \cdot 10^{6} ore\_{grade} tailings\_{Rn222} \left(e^{9.2 \cdot 10^{-6} integration\_{time Rn222}} - 1.0\right) e^{0.658 ore\_{grade}} + 4.301 ore\_{grade} e^{9.2 \cdot 10^{-6} integration\_{time Rn222} + 0.658 ore\_{grade}} + \left(0.0001492 milling\_{electricity switch dieselgenerator} + 0.00122 milling\_{electricity switch grid} + 0.001085\right) e^{9.2 \cdot 10^{-6} integration\_{time Rn222} + 0.658 ore\_{grade}}\right)\right) e^{0.0485 ore\_{grade}}\right)\right) + 202.0 enrichment\_{mix switch diffusion} \left(1.0 ore\_{grade} \left(0.209 enrichment\_{diff elec} + 28.13\right) \left(0.0723 \log{\left(100 ore\_{grade} \right)}^{2} - 5.196\right) \left(1.22 rate\_{enrichment} + 0.0049 \cdot \left(2.0 rate\_{enrichment} - 1.0\right) \log{\left(- \frac{rate\_{enrichment}}{rate\_{enrichment} - 1} \right)} - 0.03252\right) e^{9.2 \cdot 10^{-6} integration\_{time Rn222} + 0.707 ore\_{grade}} + \left(rate\_{enrichment} - 0.0022\right) \left(- 1.042 ore\_{grade} share\_{ISL} \left(4.53 mining\_{electricity switch dieselgenerator} + 37.06 mining\_{electricity switch grid} + \left(172.0 - 2.393 \log{\left(100 ore\_{grade} \right)}^{2}\right) e^{0.0485 ore\_{grade}} + 2.143\right) e^{9.2 \cdot 10^{-6} integration\_{time Rn222} + 0.658 ore\_{grade}} + 1.0 ore\_{grade} \left(0.0723 \log{\left(100 ore\_{grade} \right)}^{2} - 5.196\right) \left(0.2454 conversion\_{elec} + 0.002289 conversion\_{heat} + 33.02\right) e^{9.2 \cdot 10^{-6} integration\_{time Rn222} + 0.707 ore\_{grade}} + \left(share\_{ISL} - 1.0\right) \left(0.4648 ore\_{grade} \left(5.641 mining\_{electricity switch dieselgenerator} + 46.15 mining\_{electricity switch grid} + \left(929.3 - 12.93 \log{\left(100 ore\_{grade} \right)}^{2}\right) e^{0.482 ore\_{grade}} + 2.668\right) e^{9.2 \cdot 10^{-6} integration\_{time Rn222} + 0.176 ore\_{grade}} + 0.5774 ore\_{grade} \left(6.526 mining\_{electricity switch dieselgenerator} + 53.39 mining\_{electricity switch grid} + \left(6035.0 - 83.98 \log{\left(100 ore\_{grade} \right)}^{2}\right) e^{0.176 ore\_{grade}} + 3.087\right) e^{9.2 \cdot 10^{-6} integration\_{time Rn222} + 0.482 ore\_{grade}} - \left(0.0723 \log{\left(100 ore\_{grade} \right)}^{2} - 5.196\right) \left(4.078 \cdot 10^{6} ore\_{grade} tailings\_{Rn222} \left(e^{9.2 \cdot 10^{-6} integration\_{time Rn222}} - 1.0\right) e^{0.658 ore\_{grade}} + 4.301 ore\_{grade} e^{9.2 \cdot 10^{-6} integration\_{time Rn222} + 0.658 ore\_{grade}} + \left(0.0001492 milling\_{electricity switch dieselgenerator} + 0.00122 milling\_{electricity switch grid} + 0.001085\right) e^{9.2 \cdot 10^{-6} integration\_{time Rn222} + 0.658 ore\_{grade}}\right)\right) e^{0.0485 ore\_{grade}}\right)\right) + 1.0 ore\_{grade} \left(0.121 fuel\_{fab elec} + 24.07\right) \left(0.0723 \log{\left(100 ore\_{grade} \right)}^{2} - 5.196\right) e^{9.2 \cdot 10^{-6} integration\_{time Rn222} + 0.707 ore\_{grade}}\right) + efficiency ore\_{grade} \left(1090.0 rate\_{enrichment} - 0.6\right) \left(0.0723 \log{\left(100 ore\_{grade} \right)}^{2} - 5.196\right) \left(21.11 availability lifetime + 416.3 construction\_{intensity} + 1.576\right) e^{9.2 \cdot 10^{-6} integration\_{time Rn222} + 0.707 ore\_{grade}}\right) \left(5.082 \cdot 10^{-5} rate\_{enrichment} + 2.042 \cdot 10^{-7} \cdot \left(2.0 rate\_{enrichment} - 1.0\right) \log{\left(- \frac{rate\_{enrichment}}{rate\_{enrichment} - 1} \right)} - 1.355 \cdot 10^{-6}\right) e^{- 9.2 \cdot 10^{-6} integration\_{time Rn222} - 0.707 ore\_{grade}}}{availability efficiency lifetime ore\_{grade} \left(1090.0 rate\_{enrichment} - 0.6\right) \left(0.0723 \log{\left(100 ore\_{grade} \right)}^{2} - 5.196\right) \left(1.22 rate\_{enrichment} + 0.0049 \cdot \left(2.0 rate\_{enrichment} - 1.0\right) \log{\left(- \frac{rate\_{enrichment}}{rate\_{enrichment} - 1} \right)} - 0.03252\right)} & \text{for}\: ore\_{grade} < 0.01 \\\frac{\left(0.0008796 availability efficiency lifetime ore\_{grade} \left(1090.0 rate\_{enrichment} - 0.6\right) e^{9.2 \cdot 10^{-6} integration\_{time Rn222} + 0.707 ore\_{grade}} + 4.166 \cdot 10^{-5} availability lifetime \left(202.0 enrichment\_{mix switch centrifugation} \left(1.0 ore\_{grade} \left(0.209 enrichment\_{centr elec} + 0.702\right) \left(1.22 rate\_{enrichment} + 0.0049 \cdot \left(2.0 rate\_{enrichment} - 1.0\right) \log{\left(- \frac{rate\_{enrichment}}{rate\_{enrichment} - 1} \right)} - 0.03252\right) e^{9.2 \cdot 10^{-6} integration\_{time Rn222} + 0.707 ore\_{grade}} + \left(rate\_{enrichment} - 0.0022\right) \left(1.042 ore\_{grade} share\_{ISL} \left(0.8718 mining\_{electricity switch dieselgenerator} + 7.132 mining\_{electricity switch grid} + 33.09 e^{0.0485 ore\_{grade}} + 0.4124\right) e^{9.2 \cdot 10^{-6} integration\_{time Rn222} + 0.658 ore\_{grade}} + 1.0 ore\_{grade} \left(0.2454 conversion\_{elec} + 0.002289 conversion\_{heat} + 33.02\right) e^{9.2 \cdot 10^{-6} integration\_{time Rn222} + 0.707 ore\_{grade}} - \left(share\_{ISL} - 1.0\right) \left(4.078 \cdot 10^{6} ore\_{grade} tailings\_{Rn222} \left(e^{9.2 \cdot 10^{-6} integration\_{time Rn222}} - 1.0\right) e^{0.658 ore\_{grade}} + 0.4648 ore\_{grade} \left(1.086 mining\_{electricity switch dieselgenerator} + 8.883 mining\_{electricity switch grid} + 178.9 e^{0.482 ore\_{grade}} + 0.5135\right) e^{9.2 \cdot 10^{-6} integration\_{time Rn222} + 0.176 ore\_{grade}} + 0.5774 ore\_{grade} \left(1.256 mining\_{electricity switch dieselgenerator} + 10.28 mining\_{electricity switch grid} + 1162.0 e^{0.176 ore\_{grade}} + 0.5941\right) e^{9.2 \cdot 10^{-6} integration\_{time Rn222} + 0.482 ore\_{grade}} + 4.301 ore\_{grade} e^{9.2 \cdot 10^{-6} integration\_{time Rn222} + 0.658 ore\_{grade}} + \left(0.0001492 milling\_{electricity switch dieselgenerator} + 0.00122 milling\_{electricity switch grid} + 0.001085\right) e^{9.2 \cdot 10^{-6} integration\_{time Rn222} + 0.658 ore\_{grade}}\right) e^{0.0485 ore\_{grade}}\right)\right) + 202.0 enrichment\_{mix switch diffusion} \left(1.0 ore\_{grade} \left(0.209 enrichment\_{diff elec} + 28.13\right) \left(1.22 rate\_{enrichment} + 0.0049 \cdot \left(2.0 rate\_{enrichment} - 1.0\right) \log{\left(- \frac{rate\_{enrichment}}{rate\_{enrichment} - 1} \right)} - 0.03252\right) e^{9.2 \cdot 10^{-6} integration\_{time Rn222} + 0.707 ore\_{grade}} + \left(rate\_{enrichment} - 0.0022\right) \left(1.042 ore\_{grade} share\_{ISL} \left(0.8718 mining\_{electricity switch dieselgenerator} + 7.132 mining\_{electricity switch grid} + 33.09 e^{0.0485 ore\_{grade}} + 0.4124\right) e^{9.2 \cdot 10^{-6} integration\_{time Rn222} + 0.658 ore\_{grade}} + 1.0 ore\_{grade} \left(0.2454 conversion\_{elec} + 0.002289 conversion\_{heat} + 33.02\right) e^{9.2 \cdot 10^{-6} integration\_{time Rn222} + 0.707 ore\_{grade}} - \left(share\_{ISL} - 1.0\right) \left(4.078 \cdot 10^{6} ore\_{grade} tailings\_{Rn222} \left(e^{9.2 \cdot 10^{-6} integration\_{time Rn222}} - 1.0\right) e^{0.658 ore\_{grade}} + 0.4648 ore\_{grade} \left(1.086 mining\_{electricity switch dieselgenerator} + 8.883 mining\_{electricity switch grid} + 178.9 e^{0.482 ore\_{grade}} + 0.5135\right) e^{9.2 \cdot 10^{-6} integration\_{time Rn222} + 0.176 ore\_{grade}} + 0.5774 ore\_{grade} \left(1.256 mining\_{electricity switch dieselgenerator} + 10.28 mining\_{electricity switch grid} + 1162.0 e^{0.176 ore\_{grade}} + 0.5941\right) e^{9.2 \cdot 10^{-6} integration\_{time Rn222} + 0.482 ore\_{grade}} + 4.301 ore\_{grade} e^{9.2 \cdot 10^{-6} integration\_{time Rn222} + 0.658 ore\_{grade}} + \left(0.0001492 milling\_{electricity switch dieselgenerator} + 0.00122 milling\_{electricity switch grid} + 0.001085\right) e^{9.2 \cdot 10^{-6} integration\_{time Rn222} + 0.658 ore\_{grade}}\right) e^{0.0485 ore\_{grade}}\right)\right) + 1.0 ore\_{grade} \left(0.121 fuel\_{fab elec} + 24.07\right) e^{9.2 \cdot 10^{-6} integration\_{time Rn222} + 0.707 ore\_{grade}}\right) + 4.166 \cdot 10^{-5} efficiency ore\_{grade} \left(416.3 construction\_{intensity} + 1.576\right) \left(1090.0 rate\_{enrichment} - 0.6\right) e^{9.2 \cdot 10^{-6} integration\_{time Rn222} + 0.707 ore\_{grade}}\right) e^{- 9.2 \cdot 10^{-6} integration\_{time Rn222} - 0.707 ore\_{grade}}}{availability efficiency lifetime ore\_{grade} \left(1090.0 rate\_{enrichment} - 0.6\right)} & \text{otherwise} \end{cases}$

```
--------------------------------------------------------------------------------
('EF v3.0', 'land use', 'soil quality index')
```

$\displaystyle 0.01652 enrichment\_{mix switch centrifugation} + 0.04884 enrichment\_{mix switch diffusion} + 0.01215$

```
--------------------------------------------------------------------------------
('EF v3.0', 'material resources: metals/minerals', 'abiotic depletion potential (ADP): elements (ultimate reserves)')
```

$\displaystyle 4.233 \cdot 10^{-8} construction\_{intensity} + 1.5 \cdot 10^{-7} enrichment\_{mix switch centrifugation} share\_{ISL} + 5.272 \cdot 10^{-8} enrichment\_{mix switch centrifugation} + 1.5 \cdot 10^{-7} enrichment\_{mix switch diffusion} share\_{ISL} + 7.797 \cdot 10^{-8} enrichment\_{mix switch diffusion} + 2.27 \cdot 10^{-7}$

```
--------------------------------------------------------------------------------
('EF v3.0', 'water use', 'user deprivation potential (deprivation-weighted water consumption)')
```

$\displaystyle 0.09879 river\_{cooling} + 0.00546$

```
--------------------------------------------------------------------------------
```

In [169]:

```
simplified[-1].expr/41.95*1000
```

Out[169]:

$\displaystyle 2.35483671968117 river\_{cooling} + 0.130149832215156$

In [28]:

```
parameters_def = {params._param_name(param, NameType.NAME):param.default for param in params._param_registry().all()}
```

In [60]:

```
simplified[0].expr.subs({**parameters_def,
                               **{'mining_electricity_switch_dieselgenerator':1,
                                  'milling_electricity_switch_dieselgenerator':1,
                                  'mining_electricity_switch_grid':0,
                                  'milling_electricity_switch_grid':0,
                                  'enrichment_mix_switch_centrifugation':1,
                                  'milling_electricity_switch_grid':0,
                                 }}).evalf(n=4)
```

Out[60]:

$\displaystyle 0.0299 enrichment\_{mix switch diffusion} + 0.007432$

In [58]:

```
for s in simplified_centr:
    display(s.expr.evalf(n=4).simplify())
    print('-'*80)
```

$\displaystyle \begin{cases} \frac{1.0 \left(efficiency ore\_{grade} \left(0.0723 \log{\left(100 ore\_{grade} \right)}^{2} - 5.196\right) \left(0.04562 construction\_{intensity} + 0.001093 lifetime + 0.002532\right) e^{0.707 ore\_{grade}} + lifetime \left(7.717 \cdot 10^{-6} ore\_{grade} share\_{ISL} \left(\left(5.121 \log{\left(100 ore\_{grade} \right)}^{2} - 368.1\right) e^{0.0485 ore\_{grade}} - 141.4\right) e^{0.658 ore\_{grade}} + 0.0005519 ore\_{grade} \left(0.0723 \log{\left(100 ore\_{grade} \right)}^{2} - 5.196\right) e^{0.707 ore\_{grade}} - \left(share\_{ISL} - 1.0\right) \left(3.442 \cdot 10^{-6} ore\_{grade} \left(\left(0.5629 \log{\left(100 ore\_{grade} \right)}^{2} - 40.45\right) e^{0.482 ore\_{grade}} - 176.1\right) e^{0.176 ore\_{grade}} + 4.276 \cdot 10^{-6} ore\_{grade} \left(\left(5.288 \log{\left(100 ore\_{grade} \right)}^{2} - 380.0\right) e^{0.176 ore\_{grade}} - 203.8\right) e^{0.482 ore\_{grade}} + \left(0.0002014 ore\_{grade} + 7.043 \cdot 10^{-7}\right) \left(0.0723 \log{\left(100 ore\_{grade} \right)}^{2} - 5.196\right) e^{0.658 ore\_{grade}}\right) e^{0.0485 ore\_{grade}}\right)\right) e^{- 0.707 ore\_{grade}}}{efficiency lifetime ore\_{grade} \left(0.0723 \log{\left(100 ore\_{grade} \right)}^{2} - 5.196\right)} & \text{for}\: ore\_{grade} < 0.01 \\\frac{0.04562 construction\_{intensity}}{lifetime} + 0.001093 + \frac{0.002532}{lifetime} + \frac{5.774 \cdot 10^{-6} share\_{ISL}}{efficiency} - \frac{0.0001167 share\_{ISL} e^{- 0.482 ore\_{grade}}}{efficiency} - \frac{0.0001677 share\_{ISL} e^{- 0.176 ore\_{grade}}}{efficiency} + \frac{0.00021 share\_{ISL} e^{- 0.0485 ore\_{grade}}}{efficiency} + \frac{0.001093}{efficiency} + \frac{0.0001167 e^{- 0.482 ore\_{grade}}}{efficiency} + \frac{0.0001677 e^{- 0.176 ore\_{grade}}}{efficiency} - \frac{7.043 \cdot 10^{-7} share\_{ISL}}{efficiency ore\_{grade}} + \frac{7.043 \cdot 10^{-7}}{efficiency ore\_{grade}} & \text{otherwise} \end{cases}$

```
--------------------------------------------------------------------------------
```

$\displaystyle 0.9376 - 0.6161 share\_{ISL}$

```
--------------------------------------------------------------------------------
```

$\displaystyle \begin{cases} \frac{\left(- 2.408 \cdot 10^{-5} lifetime ore\_{grade} share\_{ISL} \left(0.002038 mining\_{electricity switch dieselgenerator} + 0.1277 mining\_{electricity switch grid} + \left(0.7779 - 0.01082 \log{\left(100 ore\_{grade} \right)}^{2}\right) e^{0.0485 ore\_{grade}} + 0.001484\right) e^{0.658 ore\_{grade}} + 1.0 lifetime \left(share\_{ISL} - 1.0\right) \left(1.074 \cdot 10^{-5} ore\_{grade} \left(0.002538 mining\_{electricity switch dieselgenerator} + 0.159 mining\_{electricity switch grid} + \left(0.009123 - 0.0001269 \log{\left(100 ore\_{grade} \right)}^{2}\right) e^{0.482 ore\_{grade}} + 0.001848\right) e^{0.176 ore\_{grade}} + 1.334 \cdot 10^{-5} ore\_{grade} \left(0.002936 mining\_{electricity switch dieselgenerator} + 0.184 mining\_{electricity switch grid} + \left(0.2387 - 0.003321 \log{\left(100 ore\_{grade} \right)}^{2}\right) e^{0.176 ore\_{grade}} + 0.002138\right) e^{0.482 ore\_{grade}} - \left(1.055 \cdot 10^{-6} ore\_{grade} + 6.299 \cdot 10^{-10}\right) \left(0.0723 \log{\left(100 ore\_{grade} \right)}^{2} - 5.196\right) e^{0.658 ore\_{grade}}\right) e^{0.0485 ore\_{grade}} + 1.0 ore\_{grade} \left(0.0723 \log{\left(100 ore\_{grade} \right)}^{2} - 5.196\right) \left(3.915 \cdot 10^{-5} construction\_{intensity} + 2.158 \cdot 10^{-6} lifetime + 6.357 \cdot 10^{-7}\right) e^{0.707 ore\_{grade}}\right) e^{- 0.707 ore\_{grade}}}{lifetime ore\_{grade} \left(0.0723 \log{\left(100 ore\_{grade} \right)}^{2} - 5.196\right)} & \text{for}\: ore\_{grade} < 0.01 \\\frac{\left(2.408 \cdot 10^{-5} lifetime ore\_{grade} share\_{ISL} \left(0.0003921 mining\_{electricity switch dieselgenerator} + 0.02457 mining\_{electricity switch grid} + 0.1497 e^{0.0485 ore\_{grade}} + 0.0002856\right) e^{0.658 ore\_{grade}} + 2.158 \cdot 10^{-6} lifetime ore\_{grade} e^{0.707 ore\_{grade}} - 1.0 lifetime \left(share\_{ISL} - 1.0\right) \left(1.074 \cdot 10^{-5} ore\_{grade} \left(0.0004884 mining\_{electricity switch dieselgenerator} + 0.03061 mining\_{electricity switch grid} + 0.001756 e^{0.482 ore\_{grade}} + 0.0003558\right) e^{0.176 ore\_{grade}} + 1.334 \cdot 10^{-5} ore\_{grade} \left(0.0005651 mining\_{electricity switch dieselgenerator} + 0.03541 mining\_{electricity switch grid} + 0.04594 e^{0.176 ore\_{grade}} + 0.0004116\right) e^{0.482 ore\_{grade}} + 1.055 \cdot 10^{-6} ore\_{grade} e^{0.658 ore\_{grade}} + 6.299 \cdot 10^{-10} e^{0.658 ore\_{grade}}\right) e^{0.0485 ore\_{grade}} + 1.0 ore\_{grade} \left(3.915 \cdot 10^{-5} construction\_{intensity} + 6.357 \cdot 10^{-7}\right) e^{0.707 ore\_{grade}}\right) e^{- 0.707 ore\_{grade}}}{lifetime ore\_{grade}} & \text{otherwise} \end{cases}$

```
--------------------------------------------------------------------------------
```

$\displaystyle 6.898 \cdot 10^{-12} share\_{ISL} + 1.077 \cdot 10^{-11}$

```
--------------------------------------------------------------------------------
```

$\displaystyle 5.842 \cdot 10^{-10} share\_{ISL} + 2.998 \cdot 10^{-10}$

```
--------------------------------------------------------------------------------
```

$\displaystyle - 94.5 tailings\_{Rn222} \left(share\_{ISL} - 1.0\right) \left(e^{9.2 \cdot 10^{-6} integration\_{time Rn222}} - 1.0\right) e^{- 9.2 \cdot 10^{-6} integration\_{time Rn222}}$

```
--------------------------------------------------------------------------------
```

$\displaystyle \begin{cases} \frac{\left(2.415 \cdot 10^{-5} lifetime ore\_{grade} share\_{ISL} \left(\left(23.74 \log{\left(100 ore\_{grade} \right)}^{2} - 1706.0\right) e^{0.0485 ore\_{grade}} - 176.7\right) e^{0.658 ore\_{grade}} - 1.0 lifetime \left(share\_{ISL} - 1.0\right) \left(1.338 \cdot 10^{-5} ore\_{grade} \left(\left(6.927 \log{\left(100 ore\_{grade} \right)}^{2} - 497.8\right) e^{0.176 ore\_{grade}} - 254.6\right) e^{0.482 ore\_{grade}} + 1.077 \cdot 10^{-5} ore\_{grade} \left(\left(18.21 \log{\left(100 ore\_{grade} \right)}^{2} - 1309.0\right) e^{0.482 ore\_{grade}} - 220.0\right) e^{0.176 ore\_{grade}} + \left(0.009409 ore\_{grade} + 3.704 \cdot 10^{-6}\right) \left(0.0723 \log{\left(100 ore\_{grade} \right)}^{2} - 5.196\right) e^{0.658 ore\_{grade}}\right) e^{0.0485 ore\_{grade}} + 1.0 ore\_{grade} \left(0.008631 lifetime + 0.3932\right) \left(0.0723 \log{\left(100 ore\_{grade} \right)}^{2} - 5.196\right) e^{0.707 ore\_{grade}}\right) e^{- 0.707 ore\_{grade}}}{lifetime ore\_{grade} \left(0.0723 \log{\left(100 ore\_{grade} \right)}^{2} - 5.196\right)} & \text{for}\: ore\_{grade} < 0.01 \\- 0.005473 share\_{ISL} - 0.0004562 share\_{ISL} e^{- 0.482 ore\_{grade}} - 0.0006557 share\_{ISL} e^{- 0.176 ore\_{grade}} + 0.0008214 share\_{ISL} e^{- 0.0485 ore\_{grade}} + 0.02204 + 0.0004562 e^{- 0.482 ore\_{grade}} + 0.0006557 e^{- 0.176 ore\_{grade}} - \frac{3.704 \cdot 10^{-6} share\_{ISL}}{ore\_{grade}} + \frac{3.704 \cdot 10^{-6}}{ore\_{grade}} + \frac{0.3932}{lifetime} & \text{otherwise} \end{cases}$

```
--------------------------------------------------------------------------------
```

$\displaystyle 1.497 \cdot 10^{-7} share\_{ISL} + 3.263 \cdot 10^{-7}$

```
--------------------------------------------------------------------------------
```

$\displaystyle 0.09879 river\_{cooling} + 0.00396$

```
--------------------------------------------------------------------------------
```

In [59]:

```
for s in simplified_centr:
    display(s.expr.subs({'ore_grade':ore_grade.default}).evalf(n=4).simplify())
    print('-'*80)
```

$\displaystyle \frac{0.04562 construction\_{intensity}}{lifetime} + 0.001093 + \frac{0.002532}{lifetime} - \frac{0.0005284 share\_{ISL}}{efficiency} + \frac{0.001848}{efficiency}$

```
--------------------------------------------------------------------------------
```

$\displaystyle 0.9376 - 0.6161 share\_{ISL}$

```
--------------------------------------------------------------------------------
```

$\displaystyle \frac{3.915 \cdot 10^{-5} construction\_{intensity}}{lifetime} - 3.504 \cdot 10^{-9} mining\_{electricity switch dieselgenerator} share\_{ISL} + 1.343 \cdot 10^{-8} mining\_{electricity switch dieselgenerator} - 2.198 \cdot 10^{-7} mining\_{electricity switch grid} share\_{ISL} + 8.417 \cdot 10^{-7} mining\_{electricity switch grid} + 1.509 \cdot 10^{-6} share\_{ISL} + 4.262 \cdot 10^{-6} + \frac{6.357 \cdot 10^{-7}}{lifetime}$

```
--------------------------------------------------------------------------------
```

$\displaystyle 6.898 \cdot 10^{-12} share\_{ISL} + 1.077 \cdot 10^{-11}$

```
--------------------------------------------------------------------------------
```

$\displaystyle 5.842 \cdot 10^{-10} share\_{ISL} + 2.998 \cdot 10^{-10}$

```
--------------------------------------------------------------------------------
```

$\displaystyle - 94.5 tailings\_{Rn222} \left(share\_{ISL} - 1.0\right) \left(e^{9.2 \cdot 10^{-6} integration\_{time Rn222}} - 1.0\right) e^{- 9.2 \cdot 10^{-6} integration\_{time Rn222}}$

```
--------------------------------------------------------------------------------
```

$\displaystyle - 0.008177 share\_{ISL} + 0.0256 + \frac{0.3932}{lifetime}$

```
--------------------------------------------------------------------------------
```

$\displaystyle 1.497 \cdot 10^{-7} share\_{ISL} + 3.263 \cdot 10^{-7}$

```
--------------------------------------------------------------------------------
```

$\displaystyle 0.09879 river\_{cooling} + 0.00396$

```
--------------------------------------------------------------------------------
```

In [62]:

```
(simplified_centr[-1].expr/41.95*1000).evalf(n=4)
```

Out[62]:

$\displaystyle 2.355 river\_{cooling} + 0.09439$

In [89]:

```
compare_simplified(elec_prod_p, impacts, simplified)
```

```
Generating samples ...
Transforming samples ...
Generating samples ...
Transforming samples ...
Generating samples ...
Transforming samples ...
Generating samples ...
Transforming samples ...
Generating samples ...
Transforming samples ...
Generating samples ...
Transforming samples ...
Generating samples ...
Transforming samples ...
Generating samples ...
Transforming samples ...
Generating samples ...
Transforming samples ...
```

In [83]:

```
# Revert back, switch to choose enrichment technique
enrichment_mix_switch = newEnumParam(
    'enrichment_mix_switch',
    label='Enrichment technology',
    values={'centrifugation':0.8,
            'diffusion':0.2},
    default='centrifugation',
    dbname='Nuclear_DB')

# Bins are not properly built, so we do it here 
enrichment_mix_switch._bins = [0]
for i in range(len(enrichment_mix_switch.values)) :
    enumvalue = enrichment_mix_switch.values[i]
    enrichment_mix_switch._bins.append(enrichment_mix_switch._bins[i] + enrichment_mix_switch.weights[enumvalue])
```

```
[ParamRegistry] Param enrichment_mix_switch was already defined in 'Nuclear_DB' : overriding.
```

## Monte-Carlo analysis¶

In [29]:

```
problem, _, Y = stats._stochastics(elec_prod_p, impacts, 2**16, var_params=None)
```

```
Generating samples ...
Transforming samples ...
```

```
Param 'rate_feed' is marked as FIXED, but passed in parameters : ignored
Param 'rate_tailings' is marked as FIXED, but passed in parameters : ignored
Param 'capacity' is marked as FIXED, but passed in parameters : ignored
```

In [37]:

```
sob = stats._sobols(impacts, problem, Y)
```

```
Processing sobol for ('EF v3.0', 'climate change', 'global warming potential (GWP100)')
Processing sobol for ('EF v3.0', 'ecotoxicity: freshwater', 'comparative toxic unit for ecosystems (CTUe) ')
Processing sobol for ('EF v3.0', 'eutrophication: freshwater', 'fraction of nutrients reaching freshwater end compartment (P)')
Processing sobol for ('EF v3.0', 'human toxicity: carcinogenic', 'comparative toxic unit for human (CTUh) ')
Processing sobol for ('EF v3.0', 'human toxicity: non-carcinogenic', 'comparative toxic unit for human (CTUh) ')
Processing sobol for ('EF v3.0', 'ionising radiation: human health', 'human exposure efficiency relative to u235')
Processing sobol for ('EF v3.0', 'land use', 'soil quality index')
Processing sobol for ('EF v3.0', 'material resources: metals/minerals', 'abiotic depletion potential (ADP): elements (ultimate reserves)')
Processing sobol for ('EF v3.0', 'water use', 'user deprivation potential (deprivation-weighted water consumption)')
```

In [40]:

```
stats._incer_stochastic_matrix(impacts, problem['names'], Y, sob)
```

```
interactive(children=(Dropdown(description='indice', options=('s1', 'st'), value='s1'), Dropdown(description='…
```

In [41]:

```
sns.set(font_scale=1)
fig, axes = plt.subplots(3,3,figsize=(20,15))

d = distrib(
    elec_prod_p,
    impacts,
    Y=Y,
    # Optional layout parameters
    axes=axes,
    percentiles=[5, 95])

for ax in axes.flatten():
    ax.set_title(ax.get_title().replace(' - ','\n'), fontsize=14)

plt.tight_layout()
plt.savefig('output/nuclear_distributions.svg')
```

## Make a string for the Excel model¶

In [99]:

```
full_expr = lca._modelToExpr(elec_prod_p, impacts)
```

In [100]:

```
default_dict = {p.name:p.default for p in  params._param_registry().all()}
```

In [101]:

```
full_expr[0][0].free_symbols
```

Out[101]:

```
{availability,
 construction_intensity,
 conversion_elec,
 conversion_heat,
 efficiency,
 enrichment_centr_elec,
 fuel_fab_elec,
 lifetime,
 milling_electricity_switch_dieselgenerator,
 milling_electricity_switch_grid,
 mining_electricity_switch_dieselgenerator,
 mining_electricity_switch_grid,
 ore_grade,
 rate_enrichment,
 share_ISL}
```

In [102]:

```
default_dict
default_dict['mining_electricity_switch_dieselgenerator'] = 1
default_dict['milling_electricity_switch_dieselgenerator'] = 1
default_dict['mining_electricity_switch_grid'] = 0
default_dict['milling_electricity_switch_grid'] = 0
default_dict['enrichment_mix_switch_centrifugation'] = 1
default_dict['enrichment_mix_switch_diffusion'] = 0
```

In [103]:

```
N(full_expr[0][0].subs(default_dict))
```

Out[103]:

$\displaystyle 0.00606104961511497$

In [39]:

```
lca.multiLCAAlgebric([elec_prod,elec_prod_p], # The model 
    impacts)
```

```
Required param 'mining_electricity_switch' was missing, replacing by default value : dieselgenerator
Required param 'fuel_fab_elec' was missing, replacing by default value : 36
Required param 'enrichment_diff_elec' was missing, replacing by default value : 2500
Required param 'lifetime' was missing, replacing by default value : 60
Required param 'ore_grade' was missing, replacing by default value : 0.001544
Required param 'share_ISL' was missing, replacing by default value : 0.574
Required param 'conversion_heat' was missing, replacing by default value : 26
Required param 'rate_enrichment' was missing, replacing by default value : 0.0415
Required param 'enrichment_mix_switch' was missing, replacing by default value : centrifugation
Required param 'milling_electricity_switch' was missing, replacing by default value : dieselgenerator
Required param 'conversion_elec' was missing, replacing by default value : 11.8
Required param 'efficiency' was missing, replacing by default value : 0.33
Required param 'tailings_Rn222' was missing, replacing by default value : 0.01951
Required param 'river_cooling' was missing, replacing by default value : 1.0
Required param 'integration_time_Rn222' was missing, replacing by default value : 80000
Required param 'construction_intensity' was missing, replacing by default value : 1
Required param 'enrichment_centr_elec' was missing, replacing by default value : 50
Required param 'availability' was missing, replacing by default value : 0.9
```

Out[39]:

|  | climate change - global warming potential (GWP100)[kg CO2-Eq] | ecotoxicity: freshwater - comparative toxic unit for ecosystems (CTUe) [CTUe] | eutrophication: freshwater - fraction of nutrients reaching freshwater end compartment (P)[kg PO4-Eq] | human toxicity: carcinogenic - comparative toxic unit for human (CTUh) [CTUh] | human toxicity: non-carcinogenic - comparative toxic unit for human (CTUh) [CTUh] | ionising radiation: human health - human exposure efficiency relative to u235[kBq U235-Eq] | land use - soil quality index[dimensionless] | material resources: metals/minerals - abiotic depletion potential (ADP): elements (ultimate reserves)[kg Sb-Eq] | water use - user deprivation potential (deprivation-weighted water consumption)[m3 world eq. deprived] |
| --- | --- | --- | --- | --- | --- | --- | --- | --- | --- |
| electricity production, nuclear, PWR, THEMIS | 0.006558 | 0.516874 | 0.000007 | 1.490705e-11 | 6.618314e-10 | 0.013154 | 0.026211 | 4.137142e-07 | 0.103022 |
| electricity production, nuclear, PWR, parameterized | 0.006061 | 0.552907 | 0.000006 | 1.398592e-11 | 6.098642e-10 | 0.406450 | 0.026269 | 3.993655e-07 | 0.102558 |

In [40]:

```
formulas = dict()

for imp, e in zip(impacts, full_expr[0]):
    formulas[imp] = utils.excelify('='+str(e))
```

In [41]:

```
formulas
```

Out[41]:

```
{('EF v3.0',
  'climate change',
  'global warming potential (GWP100)'): '=0.00109262104806847 + (0.396516247200945*fuel_fab_elec + 202.040816326531*(enrichment_mix_switch_centrifugation*(0.508954163846162*enrichment_centr_elec + 11.2882465408531 + (0.424375508389407*conversion_elec + 0.0394776697405151*conversion_heat + 1.04217599120411*share_ISL*(154.0*(0.0892326725829683*mining_electricity_switch_dieselgenerator + 0.177238805770639*mining_electricity_switch_grid)*IF((ore_grade < 0.01),1/(-0.0723*LN(100*ore_grade)^2/LN(10)^2 + 0.98),(1.02040816326531))*exp(-0.0485*ore_grade) + 6.15014367236657*IF((ore_grade < 0.01),1/(-0.0723*LN(100*ore_grade)^2/LN(10)^2 + 0.98),(1.02040816326531))*exp(-0.0485*ore_grade) + 70.836795369606) + 1.04217599120411*(1.0 - 1.0*share_ISL)*((0.445983379501385 - 0.445983379501385*share_ISL)*(191.8*(0.0892326725829683*mining_electricity_switch_dieselgenerator + 0.177238805770639*mining_electricity_switch_grid)*IF((ore_grade < 0.01),1/(-0.0723*LN(100*ore_grade)^2/LN(10)^2 + 0.98),(1.02040816326531))*exp(-0.482*ore_grade) + 7.65972439194745*IF((ore_grade < 0.01),1/(-0.0723*LN(100*ore_grade)^2/LN(10)^2 + 0.98),(1.02040816326531))*exp(-0.482*ore_grade) + 7.78564641515561)/(1.0 - 1.0*share_ISL) + (0.554016620498615 - 0.554016620498615*share_ISL)*(221.9*(0.0892326725829683*mining_electricity_switch_dieselgenerator + 0.177238805770639*mining_electricity_switch_grid)*IF((ore_grade < 0.01),1/(-0.0723*LN(100*ore_grade)^2/LN(10)^2 + 0.98),(1.02040816326531))*exp(-0.176*ore_grade) + 8.86179792791*IF((ore_grade < 0.01),1/(-0.0723*LN(100*ore_grade)^2/LN(10)^2 + 0.98),(1.02040816326531))*exp(-0.176*ore_grade) + 73.1369315092323)/(1.0 - 1.0*share_ISL) + 26.0972429849788 + 0.0258*(0.0892326725829683*milling_electricity_switch_dieselgenerator + 0.177238805770639*milling_electricity_switch_grid)/ore_grade + 0.087818031245362/ore_grade) + 20.7085137101814)/(1.219800704306 + 0.0049*(2*rate_enrichment - 1)*LN(rate_enrichment/(1 - rate_enrichment))/(rate_enrichment - 0.0022) - 0.0298418833469335/(rate_enrichment - 0.0022))) + enrichment_mix_switch_diffusion*(0.508954163846162*enrichment_diff_elec + 28.3616409780363 + (0.424375508389407*conversion_elec + 0.0394776697405151*conversion_heat + 1.04217599120411*share_ISL*(154.0*(0.0892326725829683*mining_electricity_switch_dieselgenerator + 0.177238805770639*mining_electricity_switch_grid)*IF((ore_grade < 0.01),1/(-0.0723*LN(100*ore_grade)^2/LN(10)^2 + 0.98),(1.02040816326531))*exp(-0.0485*ore_grade) + 6.15014367236657*IF((ore_grade < 0.01),1/(-0.0723*LN(100*ore_grade)^2/LN(10)^2 + 0.98),(1.02040816326531))*exp(-0.0485*ore_grade) + 70.836795369606) + 1.04217599120411*(1.0 - 1.0*share_ISL)*((0.445983379501385 - 0.445983379501385*share_ISL)*(191.8*(0.0892326725829683*mining_electricity_switch_dieselgenerator + 0.177238805770639*mining_electricity_switch_grid)*IF((ore_grade < 0.01),1/(-0.0723*LN(100*ore_grade)^2/LN(10)^2 + 0.98),(1.02040816326531))*exp(-0.482*ore_grade) + 7.65972439194745*IF((ore_grade < 0.01),1/(-0.0723*LN(100*ore_grade)^2/LN(10)^2 + 0.98),(1.02040816326531))*exp(-0.482*ore_grade) + 7.78564641515561)/(1.0 - 1.0*share_ISL) + (0.554016620498615 - 0.554016620498615*share_ISL)*(221.9*(0.0892326725829683*mining_electricity_switch_dieselgenerator + 0.177238805770639*mining_electricity_switch_grid)*IF((ore_grade < 0.01),1/(-0.0723*LN(100*ore_grade)^2/LN(10)^2 + 0.98),(1.02040816326531))*exp(-0.176*ore_grade) + 8.86179792791*IF((ore_grade < 0.01),1/(-0.0723*LN(100*ore_grade)^2/LN(10)^2 + 0.98),(1.02040816326531))*exp(-0.176*ore_grade) + 73.1369315092323)/(1.0 - 1.0*share_ISL) + 26.0972429849788 + 0.0258*(0.0892326725829683*milling_electricity_switch_dieselgenerator + 0.177238805770639*milling_electricity_switch_grid)/ore_grade + 0.087818031245362/ore_grade) + 20.7085137101814)/(1.219800704306 + 0.0049*(2*rate_enrichment - 1)*LN(rate_enrichment/(1 - rate_enrichment))/(rate_enrichment - 0.0022) - 0.0298418833469335/(rate_enrichment - 0.0022))))*(1.219800704306*rate_enrichment + 0.0049*(2*rate_enrichment - 1)*LN(-rate_enrichment/(rate_enrichment - 1)) - 0.0325254448964067) + 26.6263832957925)/(24000*efficiency*(1090.0*rate_enrichment - 0.600000000000001)) + 1.14077116130504e-10*(344721009.42676*construction_intensity + 19136892.1567673)/(availability*lifetime)',
 ('EF v3.0',
  'ecotoxicity: freshwater',
  'comparative toxic unit for ecosystems (CTUe) '): '=0.12944623336283 + (5.76959136016407*fuel_fab_elec + 202.040816326531*(enrichment_mix_switch_centrifugation*(7.82462592494476*enrichment_centr_elec + 773.904850120356 + (8.22318604899191*conversion_elec + 0.209171049040237*conversion_heat + 1.04217599120411*share_ISL*(154.0*(0.684306506985156*mining_electricity_switch_dieselgenerator + 2.53555759688304*mining_electricity_switch_grid)*IF((ore_grade < 0.01),1/(-0.0723*LN(100*ore_grade)^2/LN(10)^2 + 0.98),(1.02040816326531))*exp(-0.0485*ore_grade) + 51.7909361866492*IF((ore_grade < 0.01),1/(-0.0723*LN(100*ore_grade)^2/LN(10)^2 + 0.98),(1.02040816326531))*exp(-0.0485*ore_grade) + 4632.66129336244) + 1.04217599120411*(1.0 - 1.0*share_ISL)*((0.445983379501385 - 0.445983379501385*share_ISL)*(191.8*(0.684306506985156*mining_electricity_switch_dieselgenerator + 2.53555759688304*mining_electricity_switch_grid)*IF((ore_grade < 0.01),1/(-0.0723*LN(100*ore_grade)^2/LN(10)^2 + 0.98),(1.02040816326531))*exp(-0.482*ore_grade) + 64.5032568870085*IF((ore_grade < 0.01),1/(-0.0723*LN(100*ore_grade)^2/LN(10)^2 + 0.98),(1.02040816326531))*exp(-0.482*ore_grade) + 44979.121520835)/(1.0 - 1.0*share_ISL) + (0.554016620498615 - 0.554016620498615*share_ISL)*(221.9*(0.684306506985156*mining_electricity_switch_dieselgenerator + 2.53555759688304*mining_electricity_switch_grid)*IF((ore_grade < 0.01),1/(-0.0723*LN(100*ore_grade)^2/LN(10)^2 + 0.98),(1.02040816326531))*exp(-0.176*ore_grade) + 74.6260307780354*IF((ore_grade < 0.01),1/(-0.0723*LN(100*ore_grade)^2/LN(10)^2 + 0.98),(1.02040816326531))*exp(-0.176*ore_grade) + 11277.5340682197)/(1.0 - 1.0*share_ISL) + 1060.12172794702 + 0.0258*(0.684306506985156*milling_electricity_switch_dieselgenerator + 2.53555759688304*milling_electricity_switch_grid)/ore_grade + 3.20205664804829/ore_grade) + 423.398096119346)/(1.219800704306 + 0.0049*(2*rate_enrichment - 1)*LN(rate_enrichment/(1 - rate_enrichment))/(rate_enrichment - 0.0022) - 0.0298418833469335/(rate_enrichment - 0.0022))) + enrichment_mix_switch_diffusion*(7.82462592494476*enrichment_diff_elec + 612.861809122496 + (8.22318604899191*conversion_elec + 0.209171049040237*conversion_heat + 1.04217599120411*share_ISL*(154.0*(0.684306506985156*mining_electricity_switch_dieselgenerator + 2.53555759688304*mining_electricity_switch_grid)*IF((ore_grade < 0.01),1/(-0.0723*LN(100*ore_grade)^2/LN(10)^2 + 0.98),(1.02040816326531))*exp(-0.0485*ore_grade) + 51.7909361866492*IF((ore_grade < 0.01),1/(-0.0723*LN(100*ore_grade)^2/LN(10)^2 + 0.98),(1.02040816326531))*exp(-0.0485*ore_grade) + 4632.66129336244) + 1.04217599120411*(1.0 - 1.0*share_ISL)*((0.445983379501385 - 0.445983379501385*share_ISL)*(191.8*(0.684306506985156*mining_electricity_switch_dieselgenerator + 2.53555759688304*mining_electricity_switch_grid)*IF((ore_grade < 0.01),1/(-0.0723*LN(100*ore_grade)^2/LN(10)^2 + 0.98),(1.02040816326531))*exp(-0.482*ore_grade) + 64.5032568870085*IF((ore_grade < 0.01),1/(-0.0723*LN(100*ore_grade)^2/LN(10)^2 + 0.98),(1.02040816326531))*exp(-0.482*ore_grade) + 44979.121520835)/(1.0 - 1.0*share_ISL) + (0.554016620498615 - 0.554016620498615*share_ISL)*(221.9*(0.684306506985156*mining_electricity_switch_dieselgenerator + 2.53555759688304*mining_electricity_switch_grid)*IF((ore_grade < 0.01),1/(-0.0723*LN(100*ore_grade)^2/LN(10)^2 + 0.98),(1.02040816326531))*exp(-0.176*ore_grade) + 74.6260307780354*IF((ore_grade < 0.01),1/(-0.0723*LN(100*ore_grade)^2/LN(10)^2 + 0.98),(1.02040816326531))*exp(-0.176*ore_grade) + 11277.5340682197)/(1.0 - 1.0*share_ISL) + 1060.12172794702 + 0.0258*(0.684306506985156*milling_electricity_switch_dieselgenerator + 2.53555759688304*milling_electricity_switch_grid)/ore_grade + 3.20205664804829/ore_grade) + 423.398096119346)/(1.219800704306 + 0.0049*(2*rate_enrichment - 1)*LN(rate_enrichment/(1 - rate_enrichment))/(rate_enrichment - 0.0022) - 0.0298418833469335/(rate_enrichment - 0.0022))))*(1.219800704306*rate_enrichment + 0.0049*(2*rate_enrichment - 1)*LN(-rate_enrichment/(rate_enrichment - 1)) - 0.0325254448964067) + 857.115119677578)/(24000*efficiency*(1090.0*rate_enrichment - 0.600000000000001)) + 1.14077116130504e-10*(11997059425.2799*construction_intensity + 641114045.030553)/(availability*lifetime)',
 ('EF v3.0',
  'eutrophication: freshwater',
  'fraction of nutrients reaching freshwater end compartment (P)'): '=1.35045231125469e-6 + (0.000293608271994933*fuel_fab_elec + 202.040816326531*(enrichment_mix_switch_centrifugation*(0.000209415607336275*enrichment_centr_elec + 0.00354906231621896 + (0.000159576915665382*conversion_elec + 6.97111131045668e-7*conversion_heat + 1.04217599120411*share_ISL*(154.0*(2.49548981979895e-6*mining_electricity_switch_dieselgenerator + 0.000156389768554889*mining_electricity_switch_grid)*IF((ore_grade < 0.01),1/(-0.0723*LN(100*ore_grade)^2/LN(10)^2 + 0.98),(1.02040816326531))*exp(-0.0485*ore_grade) + 0.000279931131473367*IF((ore_grade < 0.01),1/(-0.0723*LN(100*ore_grade)^2/LN(10)^2 + 0.98),(1.02040816326531))*exp(-0.0485*ore_grade) + 0.149712701221427) + 1.04217599120411*(1.0 - 1.0*share_ISL)*((0.445983379501385 - 0.445983379501385*share_ISL)*(191.8*(2.49548981979895e-6*mining_electricity_switch_dieselgenerator + 0.000156389768554889*mining_electricity_switch_grid)*IF((ore_grade < 0.01),1/(-0.0723*LN(100*ore_grade)^2/LN(10)^2 + 0.98),(1.02040816326531))*exp(-0.482*ore_grade) + 0.000348641500107739*IF((ore_grade < 0.01),1/(-0.0723*LN(100*ore_grade)^2/LN(10)^2 + 0.98),(1.02040816326531))*exp(-0.482*ore_grade) + 0.00175570038495523)/(1.0 - 1.0*share_ISL) + (0.554016620498615 - 0.554016620498615*share_ISL)*(221.9*(2.49548981979895e-6*mining_electricity_switch_dieselgenerator + 0.000156389768554889*mining_electricity_switch_grid)*IF((ore_grade < 0.01),1/(-0.0723*LN(100*ore_grade)^2/LN(10)^2 + 0.98),(1.02040816326531))*exp(-0.176*ore_grade) + 0.000403355312168442*IF((ore_grade < 0.01),1/(-0.0723*LN(100*ore_grade)^2/LN(10)^2 + 0.98),(1.02040816326531))*exp(-0.176*ore_grade) + 0.045933022734742)/(1.0 - 1.0*share_ISL) + 0.043796778416286 + 0.0258*(2.49548981979895e-6*milling_electricity_switch_dieselgenerator + 0.000156389768554889*milling_electricity_switch_grid)/ore_grade + 2.41110340403162e-5/ore_grade) + 0.0160040374521204)/(1.219800704306 + 0.0049*(2*rate_enrichment - 1)*LN(rate_enrichment/(1 - rate_enrichment))/(rate_enrichment - 0.0022) - 0.0298418833469335/(rate_enrichment - 0.0022))) + enrichment_mix_switch_diffusion*(0.000209415607336275*enrichment_diff_elec + 0.0174003725428924 + (0.000159576915665382*conversion_elec + 6.97111131045668e-7*conversion_heat + 1.04217599120411*share_ISL*(154.0*(2.49548981979895e-6*mining_electricity_switch_dieselgenerator + 0.000156389768554889*mining_electricity_switch_grid)*IF((ore_grade < 0.01),1/(-0.0723*LN(100*ore_grade)^2/LN(10)^2 + 0.98),(1.02040816326531))*exp(-0.0485*ore_grade) + 0.000279931131473367*IF((ore_grade < 0.01),1/(-0.0723*LN(100*ore_grade)^2/LN(10)^2 + 0.98),(1.02040816326531))*exp(-0.0485*ore_grade) + 0.149712701221427) + 1.04217599120411*(1.0 - 1.0*share_ISL)*((0.445983379501385 - 0.445983379501385*share_ISL)*(191.8*(2.49548981979895e-6*mining_electricity_switch_dieselgenerator + 0.000156389768554889*mining_electricity_switch_grid)*IF((ore_grade < 0.01),1/(-0.0723*LN(100*ore_grade)^2/LN(10)^2 + 0.98),(1.02040816326531))*exp(-0.482*ore_grade) + 0.000348641500107739*IF((ore_grade < 0.01),1/(-0.0723*LN(100*ore_grade)^2/LN(10)^2 + 0.98),(1.02040816326531))*exp(-0.482*ore_grade) + 0.00175570038495523)/(1.0 - 1.0*share_ISL) + (0.554016620498615 - 0.554016620498615*share_ISL)*(221.9*(2.49548981979895e-6*mining_electricity_switch_dieselgenerator + 0.000156389768554889*mining_electricity_switch_grid)*IF((ore_grade < 0.01),1/(-0.0723*LN(100*ore_grade)^2/LN(10)^2 + 0.98),(1.02040816326531))*exp(-0.176*ore_grade) + 0.000403355312168442*IF((ore_grade < 0.01),1/(-0.0723*LN(100*ore_grade)^2/LN(10)^2 + 0.98),(1.02040816326531))*exp(-0.176*ore_grade) + 0.045933022734742)/(1.0 - 1.0*share_ISL) + 0.043796778416286 + 0.0258*(2.49548981979895e-6*milling_electricity_switch_dieselgenerator + 0.000156389768554889*milling_electricity_switch_grid)/ore_grade + 2.41110340403162e-5/ore_grade) + 0.0160040374521204)/(1.219800704306 + 0.0049*(2*rate_enrichment - 1)*LN(rate_enrichment/(1 - rate_enrichment))/(rate_enrichment - 0.0022) - 0.0298418833469335/(rate_enrichment - 0.0022))))*(1.219800704306*rate_enrichment + 0.0049*(2*rate_enrichment - 1)*LN(-rate_enrichment/(rate_enrichment - 1)) - 0.0325254448964067) + 0.0157057220303193)/(24000*efficiency*(1090.0*rate_enrichment - 0.600000000000001)) + 1.14077116130504e-10*(292334.208326767*construction_intensity + 4747.80549402654)/(availability*lifetime)',
 ('EF v3.0',
  'human toxicity: carcinogenic',
  'comparative toxic unit for human (CTUh) '): '=2.69575920297878e-12 + (9.25613540591443e-11*fuel_fab_elec + 202.040816326531*(enrichment_mix_switch_centrifugation*(8.6704760645527e-11*enrichment_centr_elec + 5.82155935411673e-8 + (8.06489843090717e-11*conversion_elec + 4.49751992147394e-12*conversion_heat + 1.04217599120411*share_ISL*(154.0*(7.50585344686937e-12*mining_electricity_switch_dieselgenerator + 4.34773520673846e-11*mining_electricity_switch_grid)*IF((ore_grade < 0.01),1/(-0.0723*LN(100*ore_grade)^2/LN(10)^2 + 0.98),(1.02040816326531))*exp(-0.0485*ore_grade) + 1.78959842731656e-9*IF((ore_grade < 0.01),1/(-0.0723*LN(100*ore_grade)^2/LN(10)^2 + 0.98),(1.02040816326531))*exp(-0.0485*ore_grade) + 4.54226727177433e-7) + 1.04217599120411*(1.0 - 1.0*share_ISL)*((0.445983379501385 - 0.445983379501385*share_ISL)*(191.8*(7.50585344686937e-12*mining_electricity_switch_dieselgenerator + 4.34773520673846e-11*mining_electricity_switch_grid)*IF((ore_grade < 0.01),1/(-0.0723*LN(100*ore_grade)^2/LN(10)^2 + 0.98),(1.02040816326531))*exp(-0.482*ore_grade) + 2.22886349583971e-9*IF((ore_grade < 0.01),1/(-0.0723*LN(100*ore_grade)^2/LN(10)^2 + 0.98),(1.02040816326531))*exp(-0.482*ore_grade) + 1.34469505515125e-8)/(1.0 - 1.0*share_ISL) + (0.554016620498615 - 0.554016620498615*share_ISL)*(221.9*(7.50585344686937e-12*mining_electricity_switch_dieselgenerator + 4.34773520673846e-11*mining_electricity_switch_grid)*IF((ore_grade < 0.01),1/(-0.0723*LN(100*ore_grade)^2/LN(10)^2 + 0.98),(1.02040816326531))*exp(-0.176*ore_grade) + 2.57864864299704e-9*IF((ore_grade < 0.01),1/(-0.0723*LN(100*ore_grade)^2/LN(10)^2 + 0.98),(1.02040816326531))*exp(-0.176*ore_grade) + 3.17772339515592e-8)/(1.0 - 1.0*share_ISL) + 1.24152769103262e-7 + 0.0258*(7.50585344686937e-12*milling_electricity_switch_dieselgenerator + 4.34773520673846e-11*milling_electricity_switch_grid)/ore_grade + 2.11075522201638e-11/ore_grade) + 1.62205237059389e-8)/(1.219800704306 + 0.0049*(2*rate_enrichment - 1)*LN(rate_enrichment/(1 - rate_enrichment))/(rate_enrichment - 0.0022) - 0.0298418833469335/(rate_enrichment - 0.0022))) + enrichment_mix_switch_diffusion*(8.6704760645527e-11*enrichment_diff_elec + 9.24945276088988e-8 + (8.06489843090717e-11*conversion_elec + 4.49751992147394e-12*conversion_heat + 1.04217599120411*share_ISL*(154.0*(7.50585344686937e-12*mining_electricity_switch_dieselgenerator + 4.34773520673846e-11*mining_electricity_switch_grid)*IF((ore_grade < 0.01),1/(-0.0723*LN(100*ore_grade)^2/LN(10)^2 + 0.98),(1.02040816326531))*exp(-0.0485*ore_grade) + 1.78959842731656e-9*IF((ore_grade < 0.01),1/(-0.0723*LN(100*ore_grade)^2/LN(10)^2 + 0.98),(1.02040816326531))*exp(-0.0485*ore_grade) + 4.54226727177433e-7) + 1.04217599120411*(1.0 - 1.0*share_ISL)*((0.445983379501385 - 0.445983379501385*share_ISL)*(191.8*(7.50585344686937e-12*mining_electricity_switch_dieselgenerator + 4.34773520673846e-11*mining_electricity_switch_grid)*IF((ore_grade < 0.01),1/(-0.0723*LN(100*ore_grade)^2/LN(10)^2 + 0.98),(1.02040816326531))*exp(-0.482*ore_grade) + 2.22886349583971e-9*IF((ore_grade < 0.01),1/(-0.0723*LN(100*ore_grade)^2/LN(10)^2 + 0.98),(1.02040816326531))*exp(-0.482*ore_grade) + 1.34469505515125e-8)/(1.0 - 1.0*share_ISL) + (0.554016620498615 - 0.554016620498615*share_ISL)*(221.9*(7.50585344686937e-12*mining_electricity_switch_dieselgenerator + 4.34773520673846e-11*mining_electricity_switch_grid)*IF((ore_grade < 0.01),1/(-0.0723*LN(100*ore_grade)^2/LN(10)^2 + 0.98),(1.02040816326531))*exp(-0.176*ore_grade) + 2.57864864299704e-9*IF((ore_grade < 0.01),1/(-0.0723*LN(100*ore_grade)^2/LN(10)^2 + 0.98),(1.02040816326531))*exp(-0.176*ore_grade) + 3.17772339515592e-8)/(1.0 - 1.0*share_ISL) + 1.24152769103262e-7 + 0.0258*(7.50585344686937e-12*milling_electricity_switch_dieselgenerator + 4.34773520673846e-11*milling_electricity_switch_grid)/ore_grade + 2.11075522201638e-11/ore_grade) + 1.62205237059389e-8)/(1.219800704306 + 0.0049*(2*rate_enrichment - 1)*LN(rate_enrichment/(1 - rate_enrichment))/(rate_enrichment - 0.0022) - 0.0298418833469335/(rate_enrichment - 0.0022))))*(1.219800704306*rate_enrichment + 0.0049*(2*rate_enrichment - 1)*LN(-rate_enrichment/(rate_enrichment - 1)) - 0.0325254448964067) + 1.45342531624198e-7)/(24000*efficiency*(1090.0*rate_enrichment - 0.600000000000001)) + 1.14077116130504e-10*(0.718683600095208*construction_intensity + 0.00591666055858505)/(availability*lifetime)',
 ('EF v3.0',
  'human toxicity: non-carcinogenic',
  'comparative toxic unit for human (CTUh) '): '=1.4113154456804e-10 + (3.47313570607353e-9*fuel_fab_elec + 202.040816326531*(enrichment_mix_switch_centrifugation*(3.22500676888899e-9*enrichment_centr_elec + 3.41285531950546e-7 + (3.22114261773152e-9*conversion_elec + 9.8359235959306e-11*conversion_heat + 1.04217599120411*share_ISL*(154.0*(9.87890071579491e-10*mining_electricity_switch_dieselgenerator + 1.91183079402386e-9*mining_electricity_switch_grid)*IF((ore_grade < 0.01),1/(-0.0723*LN(100*ore_grade)^2/LN(10)^2 + 0.98),(1.02040816326531))*exp(-0.0485*ore_grade) + 3.67645372505031e-8*IF((ore_grade < 0.01),1/(-0.0723*LN(100*ore_grade)^2/LN(10)^2 + 0.98),(1.02040816326531))*exp(-0.0485*ore_grade) + 2.80375253210534e-5) + 1.04217599120411*(1.0 - 1.0*share_ISL)*((0.445983379501385 - 0.445983379501385*share_ISL)*(191.8*(9.87890071579491e-10*mining_electricity_switch_dieselgenerator + 1.91183079402386e-9*mining_electricity_switch_grid)*IF((ore_grade < 0.01),1/(-0.0723*LN(100*ore_grade)^2/LN(10)^2 + 0.98),(1.02040816326531))*exp(-0.482*ore_grade) + 4.5788560030172e-8*IF((ore_grade < 0.01),1/(-0.0723*LN(100*ore_grade)^2/LN(10)^2 + 0.98),(1.02040816326531))*exp(-0.482*ore_grade) + 7.28410272776341e-7)/(1.0 - 1.0*share_ISL) + (0.554016620498615 - 0.554016620498615*share_ISL)*(221.9*(9.87890071579491e-10*mining_electricity_switch_dieselgenerator + 1.91183079402386e-9*mining_electricity_switch_grid)*IF((ore_grade < 0.01),1/(-0.0723*LN(100*ore_grade)^2/LN(10)^2 + 0.98),(1.02040816326531))*exp(-0.176*ore_grade) + 5.29743559473158e-8*IF((ore_grade < 0.01),1/(-0.0723*LN(100*ore_grade)^2/LN(10)^2 + 0.98),(1.02040816326531))*exp(-0.176*ore_grade) + 1.22218225897971e-6)/(1.0 - 1.0*share_ISL) + 1.98637636944006e-6 + 0.0258*(9.87890071579491e-10*milling_electricity_switch_dieselgenerator + 1.91183079402386e-9*milling_electricity_switch_grid)/ore_grade + 8.33029606228744e-10/ore_grade) + 4.18600113377616e-7)/(1.219800704306 + 0.0049*(2*rate_enrichment - 1)*LN(rate_enrichment/(1 - rate_enrichment))/(rate_enrichment - 0.0022) - 0.0298418833469335/(rate_enrichment - 0.0022))) + enrichment_mix_switch_diffusion*(3.22500676888899e-9*enrichment_diff_elec + 6.47923811754235e-7 + (3.22114261773152e-9*conversion_elec + 9.8359235959306e-11*conversion_heat + 1.04217599120411*share_ISL*(154.0*(9.87890071579491e-10*mining_electricity_switch_dieselgenerator + 1.91183079402386e-9*mining_electricity_switch_grid)*IF((ore_grade < 0.01),1/(-0.0723*LN(100*ore_grade)^2/LN(10)^2 + 0.98),(1.02040816326531))*exp(-0.0485*ore_grade) + 3.67645372505031e-8*IF((ore_grade < 0.01),1/(-0.0723*LN(100*ore_grade)^2/LN(10)^2 + 0.98),(1.02040816326531))*exp(-0.0485*ore_grade) + 2.80375253210534e-5) + 1.04217599120411*(1.0 - 1.0*share_ISL)*((0.445983379501385 - 0.445983379501385*share_ISL)*(191.8*(9.87890071579491e-10*mining_electricity_switch_dieselgenerator + 1.91183079402386e-9*mining_electricity_switch_grid)*IF((ore_grade < 0.01),1/(-0.0723*LN(100*ore_grade)^2/LN(10)^2 + 0.98),(1.02040816326531))*exp(-0.482*ore_grade) + 4.5788560030172e-8*IF((ore_grade < 0.01),1/(-0.0723*LN(100*ore_grade)^2/LN(10)^2 + 0.98),(1.02040816326531))*exp(-0.482*ore_grade) + 7.28410272776341e-7)/(1.0 - 1.0*share_ISL) + (0.554016620498615 - 0.554016620498615*share_ISL)*(221.9*(9.87890071579491e-10*mining_electricity_switch_dieselgenerator + 1.91183079402386e-9*mining_electricity_switch_grid)*IF((ore_grade < 0.01),1/(-0.0723*LN(100*ore_grade)^2/LN(10)^2 + 0.98),(1.02040816326531))*exp(-0.176*ore_grade) + 5.29743559473158e-8*IF((ore_grade < 0.01),1/(-0.0723*LN(100*ore_grade)^2/LN(10)^2 + 0.98),(1.02040816326531))*exp(-0.176*ore_grade) + 1.22218225897971e-6)/(1.0 - 1.0*share_ISL) + 1.98637636944006e-6 + 0.0258*(9.87890071579491e-10*milling_electricity_switch_dieselgenerator + 1.91183079402386e-9*milling_electricity_switch_grid)/ore_grade + 8.33029606228744e-10/ore_grade) + 4.18600113377616e-7)/(1.219800704306 + 0.0049*(2*rate_enrichment - 1)*LN(rate_enrichment/(1 - rate_enrichment))/(rate_enrichment - 0.0022) - 0.0298418833469335/(rate_enrichment - 0.0022))))*(1.219800704306*rate_enrichment + 0.0049*(2*rate_enrichment - 1)*LN(-rate_enrichment/(rate_enrichment - 1)) - 0.0325254448964067) + 5.22266326376196e-7)/(24000*efficiency*(1090.0*rate_enrichment - 0.600000000000001)) + 1.14077116130504e-10*(13.8923747590794*construction_intensity + 0.220722951251088)/(availability*lifetime)',
 ('EF v3.0',
  'ionising radiation: human health',
  'human exposure efficiency relative to u235'): '=0.000879607210257476 + (0.120978641909761*fuel_fab_elec + 202.040816326531*(enrichment_mix_switch_centrifugation*(0.208993767959516*enrichment_centr_elec + 0.70201883302818 + (0.245406721024908*conversion_elec + 0.00228859202129525*conversion_heat + 1.04217599120411*share_ISL*(154.0*(0.00554785313997973*mining_electricity_switch_dieselgenerator + 0.0453845121725285*mining_electricity_switch_grid)*IF((ore_grade < 0.01),1/(-0.0723*LN(100*ore_grade)^2/LN(10)^2 + 0.98),(1.02040816326531))*exp(-0.0485*ore_grade) + 0.404107269334488*IF((ore_grade < 0.01),1/(-0.0723*LN(100*ore_grade)^2/LN(10)^2 + 0.98),(1.02040816326531))*exp(-0.0485*ore_grade) + 33.0972116406022) + 1.04217599120411*(1.0 - 1.0*share_ISL)*(3912365.95624308*tailings_Rn222*(1 - exp(-9.19537252003111e-6*integration_time_Rn222)) + (0.445983379501385 - 0.445983379501385*share_ISL)*(191.8*(0.00554785313997973*mining_electricity_switch_dieselgenerator + 0.0453845121725285*mining_electricity_switch_grid)*IF((ore_grade < 0.01),1/(-0.0723*LN(100*ore_grade)^2/LN(10)^2 + 0.98),(1.02040816326531))*exp(-0.482*ore_grade) + 0.503297235443862*IF((ore_grade < 0.01),1/(-0.0723*LN(100*ore_grade)^2/LN(10)^2 + 0.98),(1.02040816326531))*exp(-0.482*ore_grade) + 178.86950076078)/(1.0 - 1.0*share_ISL) + (0.554016620498615 - 0.554016620498615*share_ISL)*(221.9*(0.00554785313997973*mining_electricity_switch_dieselgenerator + 0.0453845121725285*mining_electricity_switch_grid)*IF((ore_grade < 0.01),1/(-0.0723*LN(100*ore_grade)^2/LN(10)^2 + 0.98),(1.02040816326531))*exp(-0.176*ore_grade) + 0.582281838086512*IF((ore_grade < 0.01),1/(-0.0723*LN(100*ore_grade)^2/LN(10)^2 + 0.98),(1.02040816326531))*exp(-0.176*ore_grade) + 1161.55454657382)/(1.0 - 1.0*share_ISL) + 4.12632384355364 + 0.0258*(0.00554785313997973*milling_electricity_switch_dieselgenerator + 0.0453845121725285*milling_electricity_switch_grid)/ore_grade + 0.0010411214736684/ore_grade) + 33.0119954271685)/(1.219800704306 + 0.0049*(2*rate_enrichment - 1)*LN(rate_enrichment/(1 - rate_enrichment))/(rate_enrichment - 0.0022) - 0.0298418833469335/(rate_enrichment - 0.0022))) + enrichment_mix_switch_diffusion*(0.208993767959516*enrichment_diff_elec + 28.1302383360455 + (0.245406721024908*conversion_elec + 0.00228859202129525*conversion_heat + 1.04217599120411*share_ISL*(154.0*(0.00554785313997973*mining_electricity_switch_dieselgenerator + 0.0453845121725285*mining_electricity_switch_grid)*IF((ore_grade < 0.01),1/(-0.0723*LN(100*ore_grade)^2/LN(10)^2 + 0.98),(1.02040816326531))*exp(-0.0485*ore_grade) + 0.404107269334488*IF((ore_grade < 0.01),1/(-0.0723*LN(100*ore_grade)^2/LN(10)^2 + 0.98),(1.02040816326531))*exp(-0.0485*ore_grade) + 33.0972116406022) + 1.04217599120411*(1.0 - 1.0*share_ISL)*(3912365.95624308*tailings_Rn222*(1 - exp(-9.19537252003111e-6*integration_time_Rn222)) + (0.445983379501385 - 0.445983379501385*share_ISL)*(191.8*(0.00554785313997973*mining_electricity_switch_dieselgenerator + 0.0453845121725285*mining_electricity_switch_grid)*IF((ore_grade < 0.01),1/(-0.0723*LN(100*ore_grade)^2/LN(10)^2 + 0.98),(1.02040816326531))*exp(-0.482*ore_grade) + 0.503297235443862*IF((ore_grade < 0.01),1/(-0.0723*LN(100*ore_grade)^2/LN(10)^2 + 0.98),(1.02040816326531))*exp(-0.482*ore_grade) + 178.86950076078)/(1.0 - 1.0*share_ISL) + (0.554016620498615 - 0.554016620498615*share_ISL)*(221.9*(0.00554785313997973*mining_electricity_switch_dieselgenerator + 0.0453845121725285*mining_electricity_switch_grid)*IF((ore_grade < 0.01),1/(-0.0723*LN(100*ore_grade)^2/LN(10)^2 + 0.98),(1.02040816326531))*exp(-0.176*ore_grade) + 0.582281838086512*IF((ore_grade < 0.01),1/(-0.0723*LN(100*ore_grade)^2/LN(10)^2 + 0.98),(1.02040816326531))*exp(-0.176*ore_grade) + 1161.55454657382)/(1.0 - 1.0*share_ISL) + 4.12632384355364 + 0.0258*(0.00554785313997973*milling_electricity_switch_dieselgenerator + 0.0453845121725285*milling_electricity_switch_grid)/ore_grade + 0.0010411214736684/ore_grade) + 33.0119954271685)/(1.219800704306 + 0.0049*(2*rate_enrichment - 1)*LN(rate_enrichment/(1 - rate_enrichment))/(rate_enrichment - 0.0022) - 0.0298418833469335/(rate_enrichment - 0.0022))))*(1.219800704306*rate_enrichment + 0.0049*(2*rate_enrichment - 1)*LN(-rate_enrichment/(rate_enrichment - 1)) - 0.0325254448964067) + 24.0670532444177)/(24000*efficiency*(1090.0*rate_enrichment - 0.600000000000001)) + 1.14077116130504e-10*(152034843.173122*construction_intensity + 575589.2492842)/(availability*lifetime)',
 ('EF v3.0',
  'land use',
  'soil quality index'): '=0.00505692382821041 + (0.527854381819618*fuel_fab_elec + 202.040816326531*(enrichment_mix_switch_centrifugation*(0.65711431644277*enrichment_centr_elec + 48.9185558569082 + (0.577795780936465*conversion_elec + 0.0159469405318573*conversion_heat + 1.04217599120411*share_ISL*(154.0*(0.170495603413036*mining_electricity_switch_dieselgenerator + 0.10274921860281*mining_electricity_switch_grid)*IF((ore_grade < 0.01),1/(-0.0723*LN(100*ore_grade)^2/LN(10)^2 + 0.98),(1.02040816326531))*exp(-0.0485*ore_grade) + 12.2837545140374*IF((ore_grade < 0.01),1/(-0.0723*LN(100*ore_grade)^2/LN(10)^2 + 0.98),(1.02040816326531))*exp(-0.0485*ore_grade) + 328.371731950703) + 1.04217599120411*(1.0 - 1.0*share_ISL)*((0.445983379501385 - 0.445983379501385*share_ISL)*(191.8*(0.170495603413036*mining_electricity_switch_dieselgenerator + 0.10274921860281*mining_electricity_switch_grid)*IF((ore_grade < 0.01),1/(-0.0723*LN(100*ore_grade)^2/LN(10)^2 + 0.98),(1.02040816326531))*exp(-0.482*ore_grade) + 15.2988578947556*IF((ore_grade < 0.01),1/(-0.0723*LN(100*ore_grade)^2/LN(10)^2 + 0.98),(1.02040816326531))*exp(-0.482*ore_grade) + 251.907876301475)/(1.0 - 1.0*share_ISL) + (0.554016620498615 - 0.554016620498615*share_ISL)*(221.9*(0.170495603413036*mining_electricity_switch_dieselgenerator + 0.10274921860281*mining_electricity_switch_grid)*IF((ore_grade < 0.01),1/(-0.0723*LN(100*ore_grade)^2/LN(10)^2 + 0.98),(1.02040816326531))*exp(-0.176*ore_grade) + 17.699773549772*IF((ore_grade < 0.01),1/(-0.0723*LN(100*ore_grade)^2/LN(10)^2 + 0.98),(1.02040816326531))*exp(-0.176*ore_grade) + 95.8023759060552)/(1.0 - 1.0*share_ISL) + 389.532968103831 + 0.0258*(0.170495603413036*milling_electricity_switch_dieselgenerator + 0.10274921860281*milling_electricity_switch_grid)/ore_grade + 0.149808252120606/ore_grade) + 58.6211867812304)/(1.219800704306 + 0.0049*(2*rate_enrichment - 1)*LN(rate_enrichment/(1 - rate_enrichment))/(rate_enrichment - 0.0022) - 0.0298418833469335/(rate_enrichment - 0.0022))) + enrichment_mix_switch_diffusion*(0.65711431644277*enrichment_diff_elec + 100.203906410342 + (0.577795780936465*conversion_elec + 0.0159469405318573*conversion_heat + 1.04217599120411*share_ISL*(154.0*(0.170495603413036*mining_electricity_switch_dieselgenerator + 0.10274921860281*mining_electricity_switch_grid)*IF((ore_grade < 0.01),1/(-0.0723*LN(100*ore_grade)^2/LN(10)^2 + 0.98),(1.02040816326531))*exp(-0.0485*ore_grade) + 12.2837545140374*IF((ore_grade < 0.01),1/(-0.0723*LN(100*ore_grade)^2/LN(10)^2 + 0.98),(1.02040816326531))*exp(-0.0485*ore_grade) + 328.371731950703) + 1.04217599120411*(1.0 - 1.0*share_ISL)*((0.445983379501385 - 0.445983379501385*share_ISL)*(191.8*(0.170495603413036*mining_electricity_switch_dieselgenerator + 0.10274921860281*mining_electricity_switch_grid)*IF((ore_grade < 0.01),1/(-0.0723*LN(100*ore_grade)^2/LN(10)^2 + 0.98),(1.02040816326531))*exp(-0.482*ore_grade) + 15.2988578947556*IF((ore_grade < 0.01),1/(-0.0723*LN(100*ore_grade)^2/LN(10)^2 + 0.98),(1.02040816326531))*exp(-0.482*ore_grade) + 251.907876301475)/(1.0 - 1.0*share_ISL) + (0.554016620498615 - 0.554016620498615*share_ISL)*(221.9*(0.170495603413036*mining_electricity_switch_dieselgenerator + 0.10274921860281*mining_electricity_switch_grid)*IF((ore_grade < 0.01),1/(-0.0723*LN(100*ore_grade)^2/LN(10)^2 + 0.98),(1.02040816326531))*exp(-0.176*ore_grade) + 17.699773549772*IF((ore_grade < 0.01),1/(-0.0723*LN(100*ore_grade)^2/LN(10)^2 + 0.98),(1.02040816326531))*exp(-0.176*ore_grade) + 95.8023759060552)/(1.0 - 1.0*share_ISL) + 389.532968103831 + 0.0258*(0.170495603413036*milling_electricity_switch_dieselgenerator + 0.10274921860281*milling_electricity_switch_grid)/ore_grade + 0.149808252120606/ore_grade) + 58.6211867812304)/(1.219800704306 + 0.0049*(2*rate_enrichment - 1)*LN(rate_enrichment/(1 - rate_enrichment))/(rate_enrichment - 0.0022) - 0.0298418833469335/(rate_enrichment - 0.0022))))*(1.219800704306*rate_enrichment + 0.0049*(2*rate_enrichment - 1)*LN(-rate_enrichment/(rate_enrichment - 1)) - 0.0325254448964067) + 72.8574885027346)/(24000*efficiency*(1090.0*rate_enrichment - 0.600000000000001)) + 1.14077116130504e-10*(1206150153.65082*construction_intensity + 1592990884.93782)/(availability*lifetime)',
 ('EF v3.0',
  'material resources: metals/minerals',
  'abiotic depletion potential (ADP): elements (ultimate reserves)'): '=2.24900185004116e-7 + (7.13213946722281e-7*fuel_fab_elec + 202.040816326531*(enrichment_mix_switch_centrifugation*(4.20062055071576e-7*enrichment_centr_elec + 0.000348280534143669 + (3.46085741679439e-7*conversion_elec + 3.49410958910921e-8*conversion_heat + 1.04217599120411*share_ISL*(154.0*(7.4461463189856e-8*mining_electricity_switch_dieselgenerator + 2.67850732833362e-7*mining_electricity_switch_grid)*IF((ore_grade < 0.01),1/(-0.0723*LN(100*ore_grade)^2/LN(10)^2 + 0.98),(1.02040816326531))*exp(-0.0485*ore_grade) + 4.17716495157629e-6*IF((ore_grade < 0.01),1/(-0.0723*LN(100*ore_grade)^2/LN(10)^2 + 0.98),(1.02040816326531))*exp(-0.0485*ore_grade) + 0.00766589258638336) + 1.04217599120411*(1.0 - 1.0*share_ISL)*((0.445983379501385 - 0.445983379501385*share_ISL)*(191.8*(7.4461463189856e-8*mining_electricity_switch_dieselgenerator + 2.67850732833362e-7*mining_electricity_switch_grid)*IF((ore_grade < 0.01),1/(-0.0723*LN(100*ore_grade)^2/LN(10)^2 + 0.98),(1.02040816326531))*exp(-0.482*ore_grade) + 5.20246907605411e-6*IF((ore_grade < 0.01),1/(-0.0723*LN(100*ore_grade)^2/LN(10)^2 + 0.98),(1.02040816326531))*exp(-0.482*ore_grade) + 0.000136175813656906)/(1.0 - 1.0*share_ISL) + (0.554016620498615 - 0.554016620498615*share_ISL)*(221.9*(7.4461463189856e-8*mining_electricity_switch_dieselgenerator + 2.67850732833362e-7*mining_electricity_switch_grid)*IF((ore_grade < 0.01),1/(-0.0723*LN(100*ore_grade)^2/LN(10)^2 + 0.98),(1.02040816326531))*exp(-0.176*ore_grade) + 6.01891495295312e-6*IF((ore_grade < 0.01),1/(-0.0723*LN(100*ore_grade)^2/LN(10)^2 + 0.98),(1.02040816326531))*exp(-0.176*ore_grade) + 0.000145280896650256)/(1.0 - 1.0*share_ISL) + 0.00125982785229094 + 0.0258*(7.4461463189856e-8*milling_electricity_switch_dieselgenerator + 2.67850732833362e-7*milling_electricity_switch_grid)/ore_grade + 3.76042594876852e-8/ore_grade) + 0.000418106961908681)/(1.219800704306 + 0.0049*(2*rate_enrichment - 1)*LN(rate_enrichment/(1 - rate_enrichment))/(rate_enrichment - 0.0022) - 0.0298418833469335/(rate_enrichment - 0.0022))) + enrichment_mix_switch_diffusion*(4.20062055071576e-7*enrichment_diff_elec + 0.000611926843370239 + (3.46085741679439e-7*conversion_elec + 3.49410958910921e-8*conversion_heat + 1.04217599120411*share_ISL*(154.0*(7.4461463189856e-8*mining_electricity_switch_dieselgenerator + 2.67850732833362e-7*mining_electricity_switch_grid)*IF((ore_grade < 0.01),1/(-0.0723*LN(100*ore_grade)^2/LN(10)^2 + 0.98),(1.02040816326531))*exp(-0.0485*ore_grade) + 4.17716495157629e-6*IF((ore_grade < 0.01),1/(-0.0723*LN(100*ore_grade)^2/LN(10)^2 + 0.98),(1.02040816326531))*exp(-0.0485*ore_grade) + 0.00766589258638336) + 1.04217599120411*(1.0 - 1.0*share_ISL)*((0.445983379501385 - 0.445983379501385*share_ISL)*(191.8*(7.4461463189856e-8*mining_electricity_switch_dieselgenerator + 2.67850732833362e-7*mining_electricity_switch_grid)*IF((ore_grade < 0.01),1/(-0.0723*LN(100*ore_grade)^2/LN(10)^2 + 0.98),(1.02040816326531))*exp(-0.482*ore_grade) + 5.20246907605411e-6*IF((ore_grade < 0.01),1/(-0.0723*LN(100*ore_grade)^2/LN(10)^2 + 0.98),(1.02040816326531))*exp(-0.482*ore_grade) + 0.000136175813656906)/(1.0 - 1.0*share_ISL) + (0.554016620498615 - 0.554016620498615*share_ISL)*(221.9*(7.4461463189856e-8*mining_electricity_switch_dieselgenerator + 2.67850732833362e-7*mining_electricity_switch_grid)*IF((ore_grade < 0.01),1/(-0.0723*LN(100*ore_grade)^2/LN(10)^2 + 0.98),(1.02040816326531))*exp(-0.176*ore_grade) + 6.01891495295312e-6*IF((ore_grade < 0.01),1/(-0.0723*LN(100*ore_grade)^2/LN(10)^2 + 0.98),(1.02040816326531))*exp(-0.176*ore_grade) + 0.000145280896650256)/(1.0 - 1.0*share_ISL) + 0.00125982785229094 + 0.0258*(7.4461463189856e-8*milling_electricity_switch_dieselgenerator + 2.67850732833362e-7*milling_electricity_switch_grid)/ore_grade + 3.76042594876852e-8/ore_grade) + 0.000418106961908681)/(1.219800704306 + 0.0049*(2*rate_enrichment - 1)*LN(rate_enrichment/(1 - rate_enrichment))/(rate_enrichment - 0.0022) - 0.0298418833469335/(rate_enrichment - 0.0022))))*(1.219800704306*rate_enrichment + 0.0049*(2*rate_enrichment - 1)*LN(-rate_enrichment/(rate_enrichment - 1)) - 0.0325254448964067) + 0.000662018850933288)/(24000*efficiency*(1090.0*rate_enrichment - 0.600000000000001)) + 1.14077116130504e-10*(18198.9116208585*construction_intensity + 44.2180479090089)/(availability*lifetime)',
 ('EF v3.0',
  'water use',
  'user deprivation potential (deprivation-weighted water consumption)'): '=0.0987850017547607*river_cooling + 0.000576887378348076 + (0.120297151746396*fuel_fab_elec + 202.040816326531*(enrichment_mix_switch_centrifugation*(0.128354031325489*enrichment_centr_elec + 2.5432218290506 + (0.29298941455146*conversion_elec + 0.00237639492616074*conversion_heat + 1.04217599120411*share_ISL*(154.0*(0.00298224311975356*mining_electricity_switch_dieselgenerator + 0.112705922350704*mining_electricity_switch_grid)*IF((ore_grade < 0.01),1/(-0.0723*LN(100*ore_grade)^2/LN(10)^2 + 0.98),(1.02040816326531))*exp(-0.0485*ore_grade) + 0.309108654872383*IF((ore_grade < 0.01),1/(-0.0723*LN(100*ore_grade)^2/LN(10)^2 + 0.98),(1.02040816326531))*exp(-0.0485*ore_grade) + 95.7069888820048) + 1.04217599120411*(1.0 - 1.0*share_ISL)*((0.445983379501385 - 0.445983379501385*share_ISL)*(191.8*(0.00298224311975356*mining_electricity_switch_dieselgenerator + 0.112705922350704*mining_electricity_switch_grid)*IF((ore_grade < 0.01),1/(-0.0723*LN(100*ore_grade)^2/LN(10)^2 + 0.98),(1.02040816326531))*exp(-0.482*ore_grade) + 0.38498077925015*IF((ore_grade < 0.01),1/(-0.0723*LN(100*ore_grade)^2/LN(10)^2 + 0.98),(1.02040816326531))*exp(-0.482*ore_grade) + 41.7998217585077)/(1.0 - 1.0*share_ISL) + (0.554016620498615 - 0.554016620498615*share_ISL)*(221.9*(0.00298224311975356*mining_electricity_switch_dieselgenerator + 0.112705922350704*mining_electricity_switch_grid)*IF((ore_grade < 0.01),1/(-0.0723*LN(100*ore_grade)^2/LN(10)^2 + 0.98),(1.02040816326531))*exp(-0.176*ore_grade) + 0.445397470884298*IF((ore_grade < 0.01),1/(-0.0723*LN(100*ore_grade)^2/LN(10)^2 + 0.98),(1.02040816326531))*exp(-0.176*ore_grade) + 29.8451224584082)/(1.0 - 1.0*share_ISL) + 52.7642935163433 + 0.0258*(0.00298224311975356*milling_electricity_switch_dieselgenerator + 0.112705922350704*milling_electricity_switch_grid)/ore_grade + 0.00371804927536702/ore_grade) + 18.3907182562588)/(1.219800704306 + 0.0049*(2*rate_enrichment - 1)*LN(rate_enrichment/(1 - rate_enrichment))/(rate_enrichment - 0.0022) - 0.0298418833469335/(rate_enrichment - 0.0022))) + enrichment_mix_switch_diffusion*(0.128354031325489*enrichment_diff_elec + 71.3528214141035 + (0.29298941455146*conversion_elec + 0.00237639492616074*conversion_heat + 1.04217599120411*share_ISL*(154.0*(0.00298224311975356*mining_electricity_switch_dieselgenerator + 0.112705922350704*mining_electricity_switch_grid)*IF((ore_grade < 0.01),1/(-0.0723*LN(100*ore_grade)^2/LN(10)^2 + 0.98),(1.02040816326531))*exp(-0.0485*ore_grade) + 0.309108654872383*IF((ore_grade < 0.01),1/(-0.0723*LN(100*ore_grade)^2/LN(10)^2 + 0.98),(1.02040816326531))*exp(-0.0485*ore_grade) + 95.7069888820048) + 1.04217599120411*(1.0 - 1.0*share_ISL)*((0.445983379501385 - 0.445983379501385*share_ISL)*(191.8*(0.00298224311975356*mining_electricity_switch_dieselgenerator + 0.112705922350704*mining_electricity_switch_grid)*IF((ore_grade < 0.01),1/(-0.0723*LN(100*ore_grade)^2/LN(10)^2 + 0.98),(1.02040816326531))*exp(-0.482*ore_grade) + 0.38498077925015*IF((ore_grade < 0.01),1/(-0.0723*LN(100*ore_grade)^2/LN(10)^2 + 0.98),(1.02040816326531))*exp(-0.482*ore_grade) + 41.7998217585077)/(1.0 - 1.0*share_ISL) + (0.554016620498615 - 0.554016620498615*share_ISL)*(221.9*(0.00298224311975356*mining_electricity_switch_dieselgenerator + 0.112705922350704*mining_electricity_switch_grid)*IF((ore_grade < 0.01),1/(-0.0723*LN(100*ore_grade)^2/LN(10)^2 + 0.98),(1.02040816326531))*exp(-0.176*ore_grade) + 0.445397470884298*IF((ore_grade < 0.01),1/(-0.0723*LN(100*ore_grade)^2/LN(10)^2 + 0.98),(1.02040816326531))*exp(-0.176*ore_grade) + 29.8451224584082)/(1.0 - 1.0*share_ISL) + 52.7642935163433 + 0.0258*(0.00298224311975356*milling_electricity_switch_dieselgenerator + 0.112705922350704*milling_electricity_switch_grid)/ore_grade + 0.00371804927536702/ore_grade) + 18.3907182562588)/(1.219800704306 + 0.0049*(2*rate_enrichment - 1)*LN(rate_enrichment/(1 - rate_enrichment))/(rate_enrichment - 0.0022) - 0.0298418833469335/(rate_enrichment - 0.0022))))*(1.219800704306*rate_enrichment + 0.0049*(2*rate_enrichment - 1)*LN(-rate_enrichment/(rate_enrichment - 1)) - 0.0325254448964067) + 14.2908542532349)/(24000*efficiency*(1090.0*rate_enrichment - 0.600000000000001)) + 1.14077116130504e-10*(129846906.578373*construction_intensity + 1089640.83194551)/(availability*lifetime)'}
```

In [42]:

```
pd.Series(formulas).to_csv('output/nuclear_scenario1.csv')
```

In [43]:

```
formulas2 = dict()

for k,s in formulas.items():
    s2 = s
    mm = re.findall('[a-zA-Z_]+[0-9]*',s)
    mmm = set(mm) - {'IF', 'LN', 'exp', 'e'}
    scenario2 = {orig:orig+'2' if orig[-1] not in '0123456789' else orig+'_2' for orig in mmm}
    for kk,ss in scenario2.items():
        s2=s2.replace(kk,ss)
    formulas2[k] = s2
```

In [44]:

```
formulas2
```

Out[44]:

```
{('EF v3.0',
  'climate change',
  'global warming potential (GWP100)'): '=0.00109262104806847 + (0.396516247200945*fuel_fab_elec2 + 202.040816326531*(enrichment_mix_switch_centrifugation2*(0.508954163846162*enrichment_centr_elec2 + 11.2882465408531 + (0.424375508389407*conversion_elec2 + 0.0394776697405151*conversion_heat2 + 1.04217599120411*share_ISL2*(154.0*(0.0892326725829683*mining_electricity_switch_dieselgenerator2 + 0.177238805770639*mining_electricity_switch_grid2)*IF((ore_grade2 < 0.01),1/(-0.0723*LN(100*ore_grade2)^2/LN(10)^2 + 0.98),(1.02040816326531))*exp(-0.0485*ore_grade2) + 6.15014367236657*IF((ore_grade2 < 0.01),1/(-0.0723*LN(100*ore_grade2)^2/LN(10)^2 + 0.98),(1.02040816326531))*exp(-0.0485*ore_grade2) + 70.836795369606) + 1.04217599120411*(1.0 - 1.0*share_ISL2)*((0.445983379501385 - 0.445983379501385*share_ISL2)*(191.8*(0.0892326725829683*mining_electricity_switch_dieselgenerator2 + 0.177238805770639*mining_electricity_switch_grid2)*IF((ore_grade2 < 0.01),1/(-0.0723*LN(100*ore_grade2)^2/LN(10)^2 + 0.98),(1.02040816326531))*exp(-0.482*ore_grade2) + 7.65972439194745*IF((ore_grade2 < 0.01),1/(-0.0723*LN(100*ore_grade2)^2/LN(10)^2 + 0.98),(1.02040816326531))*exp(-0.482*ore_grade2) + 7.78564641515561)/(1.0 - 1.0*share_ISL2) + (0.554016620498615 - 0.554016620498615*share_ISL2)*(221.9*(0.0892326725829683*mining_electricity_switch_dieselgenerator2 + 0.177238805770639*mining_electricity_switch_grid2)*IF((ore_grade2 < 0.01),1/(-0.0723*LN(100*ore_grade2)^2/LN(10)^2 + 0.98),(1.02040816326531))*exp(-0.176*ore_grade2) + 8.86179792791*IF((ore_grade2 < 0.01),1/(-0.0723*LN(100*ore_grade2)^2/LN(10)^2 + 0.98),(1.02040816326531))*exp(-0.176*ore_grade2) + 73.1369315092323)/(1.0 - 1.0*share_ISL2) + 26.0972429849788 + 0.0258*(0.0892326725829683*milling_electricity_switch_dieselgenerator2 + 0.177238805770639*milling_electricity_switch_grid2)/ore_grade2 + 0.087818031245362/ore_grade2) + 20.7085137101814)/(1.219800704306 + 0.0049*(2*rate_enrichment2 - 1)*LN(rate_enrichment2/(1 - rate_enrichment2))/(rate_enrichment2 - 0.0022) - 0.0298418833469335/(rate_enrichment2 - 0.0022))) + enrichment_mix_switch_diffusion2*(0.508954163846162*enrichment_diff_elec2 + 28.3616409780363 + (0.424375508389407*conversion_elec2 + 0.0394776697405151*conversion_heat2 + 1.04217599120411*share_ISL2*(154.0*(0.0892326725829683*mining_electricity_switch_dieselgenerator2 + 0.177238805770639*mining_electricity_switch_grid2)*IF((ore_grade2 < 0.01),1/(-0.0723*LN(100*ore_grade2)^2/LN(10)^2 + 0.98),(1.02040816326531))*exp(-0.0485*ore_grade2) + 6.15014367236657*IF((ore_grade2 < 0.01),1/(-0.0723*LN(100*ore_grade2)^2/LN(10)^2 + 0.98),(1.02040816326531))*exp(-0.0485*ore_grade2) + 70.836795369606) + 1.04217599120411*(1.0 - 1.0*share_ISL2)*((0.445983379501385 - 0.445983379501385*share_ISL2)*(191.8*(0.0892326725829683*mining_electricity_switch_dieselgenerator2 + 0.177238805770639*mining_electricity_switch_grid2)*IF((ore_grade2 < 0.01),1/(-0.0723*LN(100*ore_grade2)^2/LN(10)^2 + 0.98),(1.02040816326531))*exp(-0.482*ore_grade2) + 7.65972439194745*IF((ore_grade2 < 0.01),1/(-0.0723*LN(100*ore_grade2)^2/LN(10)^2 + 0.98),(1.02040816326531))*exp(-0.482*ore_grade2) + 7.78564641515561)/(1.0 - 1.0*share_ISL2) + (0.554016620498615 - 0.554016620498615*share_ISL2)*(221.9*(0.0892326725829683*mining_electricity_switch_dieselgenerator2 + 0.177238805770639*mining_electricity_switch_grid2)*IF((ore_grade2 < 0.01),1/(-0.0723*LN(100*ore_grade2)^2/LN(10)^2 + 0.98),(1.02040816326531))*exp(-0.176*ore_grade2) + 8.86179792791*IF((ore_grade2 < 0.01),1/(-0.0723*LN(100*ore_grade2)^2/LN(10)^2 + 0.98),(1.02040816326531))*exp(-0.176*ore_grade2) + 73.1369315092323)/(1.0 - 1.0*share_ISL2) + 26.0972429849788 + 0.0258*(0.0892326725829683*milling_electricity_switch_dieselgenerator2 + 0.177238805770639*milling_electricity_switch_grid2)/ore_grade2 + 0.087818031245362/ore_grade2) + 20.7085137101814)/(1.219800704306 + 0.0049*(2*rate_enrichment2 - 1)*LN(rate_enrichment2/(1 - rate_enrichment2))/(rate_enrichment2 - 0.0022) - 0.0298418833469335/(rate_enrichment2 - 0.0022))))*(1.219800704306*rate_enrichment2 + 0.0049*(2*rate_enrichment2 - 1)*LN(-rate_enrichment2/(rate_enrichment2 - 1)) - 0.0325254448964067) + 26.6263832957925)/(24000*efficiency2*(1090.0*rate_enrichment2 - 0.600000000000001)) + 1.14077116130504e-10*(344721009.42676*construction_intensity2 + 19136892.1567673)/(availability2*lifetime2)',
 ('EF v3.0',
  'ecotoxicity: freshwater',
  'comparative toxic unit for ecosystems (CTUe) '): '=0.12944623336283 + (5.76959136016407*fuel_fab_elec2 + 202.040816326531*(enrichment_mix_switch_centrifugation2*(7.82462592494476*enrichment_centr_elec2 + 773.904850120356 + (8.22318604899191*conversion_elec2 + 0.209171049040237*conversion_heat2 + 1.04217599120411*share_ISL2*(154.0*(0.684306506985156*mining_electricity_switch_dieselgenerator2 + 2.53555759688304*mining_electricity_switch_grid2)*IF((ore_grade2 < 0.01),1/(-0.0723*LN(100*ore_grade2)^2/LN(10)^2 + 0.98),(1.02040816326531))*exp(-0.0485*ore_grade2) + 51.7909361866492*IF((ore_grade2 < 0.01),1/(-0.0723*LN(100*ore_grade2)^2/LN(10)^2 + 0.98),(1.02040816326531))*exp(-0.0485*ore_grade2) + 4632.66129336244) + 1.04217599120411*(1.0 - 1.0*share_ISL2)*((0.445983379501385 - 0.445983379501385*share_ISL2)*(191.8*(0.684306506985156*mining_electricity_switch_dieselgenerator2 + 2.53555759688304*mining_electricity_switch_grid2)*IF((ore_grade2 < 0.01),1/(-0.0723*LN(100*ore_grade2)^2/LN(10)^2 + 0.98),(1.02040816326531))*exp(-0.482*ore_grade2) + 64.5032568870085*IF((ore_grade2 < 0.01),1/(-0.0723*LN(100*ore_grade2)^2/LN(10)^2 + 0.98),(1.02040816326531))*exp(-0.482*ore_grade2) + 44979.121520835)/(1.0 - 1.0*share_ISL2) + (0.554016620498615 - 0.554016620498615*share_ISL2)*(221.9*(0.684306506985156*mining_electricity_switch_dieselgenerator2 + 2.53555759688304*mining_electricity_switch_grid2)*IF((ore_grade2 < 0.01),1/(-0.0723*LN(100*ore_grade2)^2/LN(10)^2 + 0.98),(1.02040816326531))*exp(-0.176*ore_grade2) + 74.6260307780354*IF((ore_grade2 < 0.01),1/(-0.0723*LN(100*ore_grade2)^2/LN(10)^2 + 0.98),(1.02040816326531))*exp(-0.176*ore_grade2) + 11277.5340682197)/(1.0 - 1.0*share_ISL2) + 1060.12172794702 + 0.0258*(0.684306506985156*milling_electricity_switch_dieselgenerator2 + 2.53555759688304*milling_electricity_switch_grid2)/ore_grade2 + 3.20205664804829/ore_grade2) + 423.398096119346)/(1.219800704306 + 0.0049*(2*rate_enrichment2 - 1)*LN(rate_enrichment2/(1 - rate_enrichment2))/(rate_enrichment2 - 0.0022) - 0.0298418833469335/(rate_enrichment2 - 0.0022))) + enrichment_mix_switch_diffusion2*(7.82462592494476*enrichment_diff_elec2 + 612.861809122496 + (8.22318604899191*conversion_elec2 + 0.209171049040237*conversion_heat2 + 1.04217599120411*share_ISL2*(154.0*(0.684306506985156*mining_electricity_switch_dieselgenerator2 + 2.53555759688304*mining_electricity_switch_grid2)*IF((ore_grade2 < 0.01),1/(-0.0723*LN(100*ore_grade2)^2/LN(10)^2 + 0.98),(1.02040816326531))*exp(-0.0485*ore_grade2) + 51.7909361866492*IF((ore_grade2 < 0.01),1/(-0.0723*LN(100*ore_grade2)^2/LN(10)^2 + 0.98),(1.02040816326531))*exp(-0.0485*ore_grade2) + 4632.66129336244) + 1.04217599120411*(1.0 - 1.0*share_ISL2)*((0.445983379501385 - 0.445983379501385*share_ISL2)*(191.8*(0.684306506985156*mining_electricity_switch_dieselgenerator2 + 2.53555759688304*mining_electricity_switch_grid2)*IF((ore_grade2 < 0.01),1/(-0.0723*LN(100*ore_grade2)^2/LN(10)^2 + 0.98),(1.02040816326531))*exp(-0.482*ore_grade2) + 64.5032568870085*IF((ore_grade2 < 0.01),1/(-0.0723*LN(100*ore_grade2)^2/LN(10)^2 + 0.98),(1.02040816326531))*exp(-0.482*ore_grade2) + 44979.121520835)/(1.0 - 1.0*share_ISL2) + (0.554016620498615 - 0.554016620498615*share_ISL2)*(221.9*(0.684306506985156*mining_electricity_switch_dieselgenerator2 + 2.53555759688304*mining_electricity_switch_grid2)*IF((ore_grade2 < 0.01),1/(-0.0723*LN(100*ore_grade2)^2/LN(10)^2 + 0.98),(1.02040816326531))*exp(-0.176*ore_grade2) + 74.6260307780354*IF((ore_grade2 < 0.01),1/(-0.0723*LN(100*ore_grade2)^2/LN(10)^2 + 0.98),(1.02040816326531))*exp(-0.176*ore_grade2) + 11277.5340682197)/(1.0 - 1.0*share_ISL2) + 1060.12172794702 + 0.0258*(0.684306506985156*milling_electricity_switch_dieselgenerator2 + 2.53555759688304*milling_electricity_switch_grid2)/ore_grade2 + 3.20205664804829/ore_grade2) + 423.398096119346)/(1.219800704306 + 0.0049*(2*rate_enrichment2 - 1)*LN(rate_enrichment2/(1 - rate_enrichment2))/(rate_enrichment2 - 0.0022) - 0.0298418833469335/(rate_enrichment2 - 0.0022))))*(1.219800704306*rate_enrichment2 + 0.0049*(2*rate_enrichment2 - 1)*LN(-rate_enrichment2/(rate_enrichment2 - 1)) - 0.0325254448964067) + 857.115119677578)/(24000*efficiency2*(1090.0*rate_enrichment2 - 0.600000000000001)) + 1.14077116130504e-10*(11997059425.2799*construction_intensity2 + 641114045.030553)/(availability2*lifetime2)',
 ('EF v3.0',
  'eutrophication: freshwater',
  'fraction of nutrients reaching freshwater end compartment (P)'): '=1.35045231125469e-6 + (0.000293608271994933*fuel_fab_elec2 + 202.040816326531*(enrichment_mix_switch_centrifugation2*(0.000209415607336275*enrichment_centr_elec2 + 0.00354906231621896 + (0.000159576915665382*conversion_elec2 + 6.97111131045668e-7*conversion_heat2 + 1.04217599120411*share_ISL2*(154.0*(2.49548981979895e-6*mining_electricity_switch_dieselgenerator2 + 0.000156389768554889*mining_electricity_switch_grid2)*IF((ore_grade2 < 0.01),1/(-0.0723*LN(100*ore_grade2)^2/LN(10)^2 + 0.98),(1.02040816326531))*exp(-0.0485*ore_grade2) + 0.000279931131473367*IF((ore_grade2 < 0.01),1/(-0.0723*LN(100*ore_grade2)^2/LN(10)^2 + 0.98),(1.02040816326531))*exp(-0.0485*ore_grade2) + 0.149712701221427) + 1.04217599120411*(1.0 - 1.0*share_ISL2)*((0.445983379501385 - 0.445983379501385*share_ISL2)*(191.8*(2.49548981979895e-6*mining_electricity_switch_dieselgenerator2 + 0.000156389768554889*mining_electricity_switch_grid2)*IF((ore_grade2 < 0.01),1/(-0.0723*LN(100*ore_grade2)^2/LN(10)^2 + 0.98),(1.02040816326531))*exp(-0.482*ore_grade2) + 0.000348641500107739*IF((ore_grade2 < 0.01),1/(-0.0723*LN(100*ore_grade2)^2/LN(10)^2 + 0.98),(1.02040816326531))*exp(-0.482*ore_grade2) + 0.00175570038495523)/(1.0 - 1.0*share_ISL2) + (0.554016620498615 - 0.554016620498615*share_ISL2)*(221.9*(2.49548981979895e-6*mining_electricity_switch_dieselgenerator2 + 0.000156389768554889*mining_electricity_switch_grid2)*IF((ore_grade2 < 0.01),1/(-0.0723*LN(100*ore_grade2)^2/LN(10)^2 + 0.98),(1.02040816326531))*exp(-0.176*ore_grade2) + 0.000403355312168442*IF((ore_grade2 < 0.01),1/(-0.0723*LN(100*ore_grade2)^2/LN(10)^2 + 0.98),(1.02040816326531))*exp(-0.176*ore_grade2) + 0.045933022734742)/(1.0 - 1.0*share_ISL2) + 0.043796778416286 + 0.0258*(2.49548981979895e-6*milling_electricity_switch_dieselgenerator2 + 0.000156389768554889*milling_electricity_switch_grid2)/ore_grade2 + 2.41110340403162e-5/ore_grade2) + 0.0160040374521204)/(1.219800704306 + 0.0049*(2*rate_enrichment2 - 1)*LN(rate_enrichment2/(1 - rate_enrichment2))/(rate_enrichment2 - 0.0022) - 0.0298418833469335/(rate_enrichment2 - 0.0022))) + enrichment_mix_switch_diffusion2*(0.000209415607336275*enrichment_diff_elec2 + 0.0174003725428924 + (0.000159576915665382*conversion_elec2 + 6.97111131045668e-7*conversion_heat2 + 1.04217599120411*share_ISL2*(154.0*(2.49548981979895e-6*mining_electricity_switch_dieselgenerator2 + 0.000156389768554889*mining_electricity_switch_grid2)*IF((ore_grade2 < 0.01),1/(-0.0723*LN(100*ore_grade2)^2/LN(10)^2 + 0.98),(1.02040816326531))*exp(-0.0485*ore_grade2) + 0.000279931131473367*IF((ore_grade2 < 0.01),1/(-0.0723*LN(100*ore_grade2)^2/LN(10)^2 + 0.98),(1.02040816326531))*exp(-0.0485*ore_grade2) + 0.149712701221427) + 1.04217599120411*(1.0 - 1.0*share_ISL2)*((0.445983379501385 - 0.445983379501385*share_ISL2)*(191.8*(2.49548981979895e-6*mining_electricity_switch_dieselgenerator2 + 0.000156389768554889*mining_electricity_switch_grid2)*IF((ore_grade2 < 0.01),1/(-0.0723*LN(100*ore_grade2)^2/LN(10)^2 + 0.98),(1.02040816326531))*exp(-0.482*ore_grade2) + 0.000348641500107739*IF((ore_grade2 < 0.01),1/(-0.0723*LN(100*ore_grade2)^2/LN(10)^2 + 0.98),(1.02040816326531))*exp(-0.482*ore_grade2) + 0.00175570038495523)/(1.0 - 1.0*share_ISL2) + (0.554016620498615 - 0.554016620498615*share_ISL2)*(221.9*(2.49548981979895e-6*mining_electricity_switch_dieselgenerator2 + 0.000156389768554889*mining_electricity_switch_grid2)*IF((ore_grade2 < 0.01),1/(-0.0723*LN(100*ore_grade2)^2/LN(10)^2 + 0.98),(1.02040816326531))*exp(-0.176*ore_grade2) + 0.000403355312168442*IF((ore_grade2 < 0.01),1/(-0.0723*LN(100*ore_grade2)^2/LN(10)^2 + 0.98),(1.02040816326531))*exp(-0.176*ore_grade2) + 0.045933022734742)/(1.0 - 1.0*share_ISL2) + 0.043796778416286 + 0.0258*(2.49548981979895e-6*milling_electricity_switch_dieselgenerator2 + 0.000156389768554889*milling_electricity_switch_grid2)/ore_grade2 + 2.41110340403162e-5/ore_grade2) + 0.0160040374521204)/(1.219800704306 + 0.0049*(2*rate_enrichment2 - 1)*LN(rate_enrichment2/(1 - rate_enrichment2))/(rate_enrichment2 - 0.0022) - 0.0298418833469335/(rate_enrichment2 - 0.0022))))*(1.219800704306*rate_enrichment2 + 0.0049*(2*rate_enrichment2 - 1)*LN(-rate_enrichment2/(rate_enrichment2 - 1)) - 0.0325254448964067) + 0.0157057220303193)/(24000*efficiency2*(1090.0*rate_enrichment2 - 0.600000000000001)) + 1.14077116130504e-10*(292334.208326767*construction_intensity2 + 4747.80549402654)/(availability2*lifetime2)',
 ('EF v3.0',
  'human toxicity: carcinogenic',
  'comparative toxic unit for human (CTUh) '): '=2.69575920297878e-12 + (9.25613540591443e-11*fuel_fab_elec2 + 202.040816326531*(enrichment_mix_switch_centrifugation2*(8.6704760645527e-11*enrichment_centr_elec2 + 5.82155935411673e-8 + (8.06489843090717e-11*conversion_elec2 + 4.49751992147394e-12*conversion_heat2 + 1.04217599120411*share_ISL2*(154.0*(7.50585344686937e-12*mining_electricity_switch_dieselgenerator2 + 4.34773520673846e-11*mining_electricity_switch_grid2)*IF((ore_grade2 < 0.01),1/(-0.0723*LN(100*ore_grade2)^2/LN(10)^2 + 0.98),(1.02040816326531))*exp(-0.0485*ore_grade2) + 1.78959842731656e-9*IF((ore_grade2 < 0.01),1/(-0.0723*LN(100*ore_grade2)^2/LN(10)^2 + 0.98),(1.02040816326531))*exp(-0.0485*ore_grade2) + 4.54226727177433e-7) + 1.04217599120411*(1.0 - 1.0*share_ISL2)*((0.445983379501385 - 0.445983379501385*share_ISL2)*(191.8*(7.50585344686937e-12*mining_electricity_switch_dieselgenerator2 + 4.34773520673846e-11*mining_electricity_switch_grid2)*IF((ore_grade2 < 0.01),1/(-0.0723*LN(100*ore_grade2)^2/LN(10)^2 + 0.98),(1.02040816326531))*exp(-0.482*ore_grade2) + 2.22886349583971e-9*IF((ore_grade2 < 0.01),1/(-0.0723*LN(100*ore_grade2)^2/LN(10)^2 + 0.98),(1.02040816326531))*exp(-0.482*ore_grade2) + 1.34469505515125e-8)/(1.0 - 1.0*share_ISL2) + (0.554016620498615 - 0.554016620498615*share_ISL2)*(221.9*(7.50585344686937e-12*mining_electricity_switch_dieselgenerator2 + 4.34773520673846e-11*mining_electricity_switch_grid2)*IF((ore_grade2 < 0.01),1/(-0.0723*LN(100*ore_grade2)^2/LN(10)^2 + 0.98),(1.02040816326531))*exp(-0.176*ore_grade2) + 2.57864864299704e-9*IF((ore_grade2 < 0.01),1/(-0.0723*LN(100*ore_grade2)^2/LN(10)^2 + 0.98),(1.02040816326531))*exp(-0.176*ore_grade2) + 3.17772339515592e-8)/(1.0 - 1.0*share_ISL2) + 1.24152769103262e-7 + 0.0258*(7.50585344686937e-12*milling_electricity_switch_dieselgenerator2 + 4.34773520673846e-11*milling_electricity_switch_grid2)/ore_grade2 + 2.11075522201638e-11/ore_grade2) + 1.62205237059389e-8)/(1.219800704306 + 0.0049*(2*rate_enrichment2 - 1)*LN(rate_enrichment2/(1 - rate_enrichment2))/(rate_enrichment2 - 0.0022) - 0.0298418833469335/(rate_enrichment2 - 0.0022))) + enrichment_mix_switch_diffusion2*(8.6704760645527e-11*enrichment_diff_elec2 + 9.24945276088988e-8 + (8.06489843090717e-11*conversion_elec2 + 4.49751992147394e-12*conversion_heat2 + 1.04217599120411*share_ISL2*(154.0*(7.50585344686937e-12*mining_electricity_switch_dieselgenerator2 + 4.34773520673846e-11*mining_electricity_switch_grid2)*IF((ore_grade2 < 0.01),1/(-0.0723*LN(100*ore_grade2)^2/LN(10)^2 + 0.98),(1.02040816326531))*exp(-0.0485*ore_grade2) + 1.78959842731656e-9*IF((ore_grade2 < 0.01),1/(-0.0723*LN(100*ore_grade2)^2/LN(10)^2 + 0.98),(1.02040816326531))*exp(-0.0485*ore_grade2) + 4.54226727177433e-7) + 1.04217599120411*(1.0 - 1.0*share_ISL2)*((0.445983379501385 - 0.445983379501385*share_ISL2)*(191.8*(7.50585344686937e-12*mining_electricity_switch_dieselgenerator2 + 4.34773520673846e-11*mining_electricity_switch_grid2)*IF((ore_grade2 < 0.01),1/(-0.0723*LN(100*ore_grade2)^2/LN(10)^2 + 0.98),(1.02040816326531))*exp(-0.482*ore_grade2) + 2.22886349583971e-9*IF((ore_grade2 < 0.01),1/(-0.0723*LN(100*ore_grade2)^2/LN(10)^2 + 0.98),(1.02040816326531))*exp(-0.482*ore_grade2) + 1.34469505515125e-8)/(1.0 - 1.0*share_ISL2) + (0.554016620498615 - 0.554016620498615*share_ISL2)*(221.9*(7.50585344686937e-12*mining_electricity_switch_dieselgenerator2 + 4.34773520673846e-11*mining_electricity_switch_grid2)*IF((ore_grade2 < 0.01),1/(-0.0723*LN(100*ore_grade2)^2/LN(10)^2 + 0.98),(1.02040816326531))*exp(-0.176*ore_grade2) + 2.57864864299704e-9*IF((ore_grade2 < 0.01),1/(-0.0723*LN(100*ore_grade2)^2/LN(10)^2 + 0.98),(1.02040816326531))*exp(-0.176*ore_grade2) + 3.17772339515592e-8)/(1.0 - 1.0*share_ISL2) + 1.24152769103262e-7 + 0.0258*(7.50585344686937e-12*milling_electricity_switch_dieselgenerator2 + 4.34773520673846e-11*milling_electricity_switch_grid2)/ore_grade2 + 2.11075522201638e-11/ore_grade2) + 1.62205237059389e-8)/(1.219800704306 + 0.0049*(2*rate_enrichment2 - 1)*LN(rate_enrichment2/(1 - rate_enrichment2))/(rate_enrichment2 - 0.0022) - 0.0298418833469335/(rate_enrichment2 - 0.0022))))*(1.219800704306*rate_enrichment2 + 0.0049*(2*rate_enrichment2 - 1)*LN(-rate_enrichment2/(rate_enrichment2 - 1)) - 0.0325254448964067) + 1.45342531624198e-7)/(24000*efficiency2*(1090.0*rate_enrichment2 - 0.600000000000001)) + 1.14077116130504e-10*(0.718683600095208*construction_intensity2 + 0.00591666055858505)/(availability2*lifetime2)',
 ('EF v3.0',
  'human toxicity: non-carcinogenic',
  'comparative toxic unit for human (CTUh) '): '=1.4113154456804e-10 + (3.47313570607353e-9*fuel_fab_elec2 + 202.040816326531*(enrichment_mix_switch_centrifugation2*(3.22500676888899e-9*enrichment_centr_elec2 + 3.41285531950546e-7 + (3.22114261773152e-9*conversion_elec2 + 9.8359235959306e-11*conversion_heat2 + 1.04217599120411*share_ISL2*(154.0*(9.87890071579491e-10*mining_electricity_switch_dieselgenerator2 + 1.91183079402386e-9*mining_electricity_switch_grid2)*IF((ore_grade2 < 0.01),1/(-0.0723*LN(100*ore_grade2)^2/LN(10)^2 + 0.98),(1.02040816326531))*exp(-0.0485*ore_grade2) + 3.67645372505031e-8*IF((ore_grade2 < 0.01),1/(-0.0723*LN(100*ore_grade2)^2/LN(10)^2 + 0.98),(1.02040816326531))*exp(-0.0485*ore_grade2) + 2.80375253210534e-5) + 1.04217599120411*(1.0 - 1.0*share_ISL2)*((0.445983379501385 - 0.445983379501385*share_ISL2)*(191.8*(9.87890071579491e-10*mining_electricity_switch_dieselgenerator2 + 1.91183079402386e-9*mining_electricity_switch_grid2)*IF((ore_grade2 < 0.01),1/(-0.0723*LN(100*ore_grade2)^2/LN(10)^2 + 0.98),(1.02040816326531))*exp(-0.482*ore_grade2) + 4.5788560030172e-8*IF((ore_grade2 < 0.01),1/(-0.0723*LN(100*ore_grade2)^2/LN(10)^2 + 0.98),(1.02040816326531))*exp(-0.482*ore_grade2) + 7.28410272776341e-7)/(1.0 - 1.0*share_ISL2) + (0.554016620498615 - 0.554016620498615*share_ISL2)*(221.9*(9.87890071579491e-10*mining_electricity_switch_dieselgenerator2 + 1.91183079402386e-9*mining_electricity_switch_grid2)*IF((ore_grade2 < 0.01),1/(-0.0723*LN(100*ore_grade2)^2/LN(10)^2 + 0.98),(1.02040816326531))*exp(-0.176*ore_grade2) + 5.29743559473158e-8*IF((ore_grade2 < 0.01),1/(-0.0723*LN(100*ore_grade2)^2/LN(10)^2 + 0.98),(1.02040816326531))*exp(-0.176*ore_grade2) + 1.22218225897971e-6)/(1.0 - 1.0*share_ISL2) + 1.98637636944006e-6 + 0.0258*(9.87890071579491e-10*milling_electricity_switch_dieselgenerator2 + 1.91183079402386e-9*milling_electricity_switch_grid2)/ore_grade2 + 8.33029606228744e-10/ore_grade2) + 4.18600113377616e-7)/(1.219800704306 + 0.0049*(2*rate_enrichment2 - 1)*LN(rate_enrichment2/(1 - rate_enrichment2))/(rate_enrichment2 - 0.0022) - 0.0298418833469335/(rate_enrichment2 - 0.0022))) + enrichment_mix_switch_diffusion2*(3.22500676888899e-9*enrichment_diff_elec2 + 6.47923811754235e-7 + (3.22114261773152e-9*conversion_elec2 + 9.8359235959306e-11*conversion_heat2 + 1.04217599120411*share_ISL2*(154.0*(9.87890071579491e-10*mining_electricity_switch_dieselgenerator2 + 1.91183079402386e-9*mining_electricity_switch_grid2)*IF((ore_grade2 < 0.01),1/(-0.0723*LN(100*ore_grade2)^2/LN(10)^2 + 0.98),(1.02040816326531))*exp(-0.0485*ore_grade2) + 3.67645372505031e-8*IF((ore_grade2 < 0.01),1/(-0.0723*LN(100*ore_grade2)^2/LN(10)^2 + 0.98),(1.02040816326531))*exp(-0.0485*ore_grade2) + 2.80375253210534e-5) + 1.04217599120411*(1.0 - 1.0*share_ISL2)*((0.445983379501385 - 0.445983379501385*share_ISL2)*(191.8*(9.87890071579491e-10*mining_electricity_switch_dieselgenerator2 + 1.91183079402386e-9*mining_electricity_switch_grid2)*IF((ore_grade2 < 0.01),1/(-0.0723*LN(100*ore_grade2)^2/LN(10)^2 + 0.98),(1.02040816326531))*exp(-0.482*ore_grade2) + 4.5788560030172e-8*IF((ore_grade2 < 0.01),1/(-0.0723*LN(100*ore_grade2)^2/LN(10)^2 + 0.98),(1.02040816326531))*exp(-0.482*ore_grade2) + 7.28410272776341e-7)/(1.0 - 1.0*share_ISL2) + (0.554016620498615 - 0.554016620498615*share_ISL2)*(221.9*(9.87890071579491e-10*mining_electricity_switch_dieselgenerator2 + 1.91183079402386e-9*mining_electricity_switch_grid2)*IF((ore_grade2 < 0.01),1/(-0.0723*LN(100*ore_grade2)^2/LN(10)^2 + 0.98),(1.02040816326531))*exp(-0.176*ore_grade2) + 5.29743559473158e-8*IF((ore_grade2 < 0.01),1/(-0.0723*LN(100*ore_grade2)^2/LN(10)^2 + 0.98),(1.02040816326531))*exp(-0.176*ore_grade2) + 1.22218225897971e-6)/(1.0 - 1.0*share_ISL2) + 1.98637636944006e-6 + 0.0258*(9.87890071579491e-10*milling_electricity_switch_dieselgenerator2 + 1.91183079402386e-9*milling_electricity_switch_grid2)/ore_grade2 + 8.33029606228744e-10/ore_grade2) + 4.18600113377616e-7)/(1.219800704306 + 0.0049*(2*rate_enrichment2 - 1)*LN(rate_enrichment2/(1 - rate_enrichment2))/(rate_enrichment2 - 0.0022) - 0.0298418833469335/(rate_enrichment2 - 0.0022))))*(1.219800704306*rate_enrichment2 + 0.0049*(2*rate_enrichment2 - 1)*LN(-rate_enrichment2/(rate_enrichment2 - 1)) - 0.0325254448964067) + 5.22266326376196e-7)/(24000*efficiency2*(1090.0*rate_enrichment2 - 0.600000000000001)) + 1.14077116130504e-10*(13.8923747590794*construction_intensity2 + 0.220722951251088)/(availability2*lifetime2)',
 ('EF v3.0',
  'ionising radiation: human health',
  'human exposure efficiency relative to u235'): '=0.000879607210257476 + (0.120978641909761*fuel_fab_elec2 + 202.040816326531*(enrichment_mix_switch_centrifugation2*(0.208993767959516*enrichment_centr_elec2 + 0.70201883302818 + (0.245406721024908*conversion_elec2 + 0.00228859202129525*conversion_heat2 + 1.04217599120411*share_ISL2*(154.0*(0.00554785313997973*mining_electricity_switch_dieselgenerator2 + 0.0453845121725285*mining_electricity_switch_grid2)*IF((ore_grade2 < 0.01),1/(-0.0723*LN(100*ore_grade2)^2/LN(10)^2 + 0.98),(1.02040816326531))*exp(-0.0485*ore_grade2) + 0.404107269334488*IF((ore_grade2 < 0.01),1/(-0.0723*LN(100*ore_grade2)^2/LN(10)^2 + 0.98),(1.02040816326531))*exp(-0.0485*ore_grade2) + 33.0972116406022) + 1.04217599120411*(1.0 - 1.0*share_ISL2)*(3912365.95624308*tailings_Rn222_2*(1 - exp(-9.19537252003111e-6*integration_time_Rn222_2)) + (0.445983379501385 - 0.445983379501385*share_ISL2)*(191.8*(0.00554785313997973*mining_electricity_switch_dieselgenerator2 + 0.0453845121725285*mining_electricity_switch_grid2)*IF((ore_grade2 < 0.01),1/(-0.0723*LN(100*ore_grade2)^2/LN(10)^2 + 0.98),(1.02040816326531))*exp(-0.482*ore_grade2) + 0.503297235443862*IF((ore_grade2 < 0.01),1/(-0.0723*LN(100*ore_grade2)^2/LN(10)^2 + 0.98),(1.02040816326531))*exp(-0.482*ore_grade2) + 178.86950076078)/(1.0 - 1.0*share_ISL2) + (0.554016620498615 - 0.554016620498615*share_ISL2)*(221.9*(0.00554785313997973*mining_electricity_switch_dieselgenerator2 + 0.0453845121725285*mining_electricity_switch_grid2)*IF((ore_grade2 < 0.01),1/(-0.0723*LN(100*ore_grade2)^2/LN(10)^2 + 0.98),(1.02040816326531))*exp(-0.176*ore_grade2) + 0.582281838086512*IF((ore_grade2 < 0.01),1/(-0.0723*LN(100*ore_grade2)^2/LN(10)^2 + 0.98),(1.02040816326531))*exp(-0.176*ore_grade2) + 1161.55454657382)/(1.0 - 1.0*share_ISL2) + 4.12632384355364 + 0.0258*(0.00554785313997973*milling_electricity_switch_dieselgenerator2 + 0.0453845121725285*milling_electricity_switch_grid2)/ore_grade2 + 0.0010411214736684/ore_grade2) + 33.0119954271685)/(1.219800704306 + 0.0049*(2*rate_enrichment2 - 1)*LN(rate_enrichment2/(1 - rate_enrichment2))/(rate_enrichment2 - 0.0022) - 0.0298418833469335/(rate_enrichment2 - 0.0022))) + enrichment_mix_switch_diffusion2*(0.208993767959516*enrichment_diff_elec2 + 28.1302383360455 + (0.245406721024908*conversion_elec2 + 0.00228859202129525*conversion_heat2 + 1.04217599120411*share_ISL2*(154.0*(0.00554785313997973*mining_electricity_switch_dieselgenerator2 + 0.0453845121725285*mining_electricity_switch_grid2)*IF((ore_grade2 < 0.01),1/(-0.0723*LN(100*ore_grade2)^2/LN(10)^2 + 0.98),(1.02040816326531))*exp(-0.0485*ore_grade2) + 0.404107269334488*IF((ore_grade2 < 0.01),1/(-0.0723*LN(100*ore_grade2)^2/LN(10)^2 + 0.98),(1.02040816326531))*exp(-0.0485*ore_grade2) + 33.0972116406022) + 1.04217599120411*(1.0 - 1.0*share_ISL2)*(3912365.95624308*tailings_Rn222_2*(1 - exp(-9.19537252003111e-6*integration_time_Rn222_2)) + (0.445983379501385 - 0.445983379501385*share_ISL2)*(191.8*(0.00554785313997973*mining_electricity_switch_dieselgenerator2 + 0.0453845121725285*mining_electricity_switch_grid2)*IF((ore_grade2 < 0.01),1/(-0.0723*LN(100*ore_grade2)^2/LN(10)^2 + 0.98),(1.02040816326531))*exp(-0.482*ore_grade2) + 0.503297235443862*IF((ore_grade2 < 0.01),1/(-0.0723*LN(100*ore_grade2)^2/LN(10)^2 + 0.98),(1.02040816326531))*exp(-0.482*ore_grade2) + 178.86950076078)/(1.0 - 1.0*share_ISL2) + (0.554016620498615 - 0.554016620498615*share_ISL2)*(221.9*(0.00554785313997973*mining_electricity_switch_dieselgenerator2 + 0.0453845121725285*mining_electricity_switch_grid2)*IF((ore_grade2 < 0.01),1/(-0.0723*LN(100*ore_grade2)^2/LN(10)^2 + 0.98),(1.02040816326531))*exp(-0.176*ore_grade2) + 0.582281838086512*IF((ore_grade2 < 0.01),1/(-0.0723*LN(100*ore_grade2)^2/LN(10)^2 + 0.98),(1.02040816326531))*exp(-0.176*ore_grade2) + 1161.55454657382)/(1.0 - 1.0*share_ISL2) + 4.12632384355364 + 0.0258*(0.00554785313997973*milling_electricity_switch_dieselgenerator2 + 0.0453845121725285*milling_electricity_switch_grid2)/ore_grade2 + 0.0010411214736684/ore_grade2) + 33.0119954271685)/(1.219800704306 + 0.0049*(2*rate_enrichment2 - 1)*LN(rate_enrichment2/(1 - rate_enrichment2))/(rate_enrichment2 - 0.0022) - 0.0298418833469335/(rate_enrichment2 - 0.0022))))*(1.219800704306*rate_enrichment2 + 0.0049*(2*rate_enrichment2 - 1)*LN(-rate_enrichment2/(rate_enrichment2 - 1)) - 0.0325254448964067) + 24.0670532444177)/(24000*efficiency2*(1090.0*rate_enrichment2 - 0.600000000000001)) + 1.14077116130504e-10*(152034843.173122*construction_intensity2 + 575589.2492842)/(availability2*lifetime2)',
 ('EF v3.0',
  'land use',
  'soil quality index'): '=0.00505692382821041 + (0.527854381819618*fuel_fab_elec2 + 202.040816326531*(enrichment_mix_switch_centrifugation2*(0.65711431644277*enrichment_centr_elec2 + 48.9185558569082 + (0.577795780936465*conversion_elec2 + 0.0159469405318573*conversion_heat2 + 1.04217599120411*share_ISL2*(154.0*(0.170495603413036*mining_electricity_switch_dieselgenerator2 + 0.10274921860281*mining_electricity_switch_grid2)*IF((ore_grade2 < 0.01),1/(-0.0723*LN(100*ore_grade2)^2/LN(10)^2 + 0.98),(1.02040816326531))*exp(-0.0485*ore_grade2) + 12.2837545140374*IF((ore_grade2 < 0.01),1/(-0.0723*LN(100*ore_grade2)^2/LN(10)^2 + 0.98),(1.02040816326531))*exp(-0.0485*ore_grade2) + 328.371731950703) + 1.04217599120411*(1.0 - 1.0*share_ISL2)*((0.445983379501385 - 0.445983379501385*share_ISL2)*(191.8*(0.170495603413036*mining_electricity_switch_dieselgenerator2 + 0.10274921860281*mining_electricity_switch_grid2)*IF((ore_grade2 < 0.01),1/(-0.0723*LN(100*ore_grade2)^2/LN(10)^2 + 0.98),(1.02040816326531))*exp(-0.482*ore_grade2) + 15.2988578947556*IF((ore_grade2 < 0.01),1/(-0.0723*LN(100*ore_grade2)^2/LN(10)^2 + 0.98),(1.02040816326531))*exp(-0.482*ore_grade2) + 251.907876301475)/(1.0 - 1.0*share_ISL2) + (0.554016620498615 - 0.554016620498615*share_ISL2)*(221.9*(0.170495603413036*mining_electricity_switch_dieselgenerator2 + 0.10274921860281*mining_electricity_switch_grid2)*IF((ore_grade2 < 0.01),1/(-0.0723*LN(100*ore_grade2)^2/LN(10)^2 + 0.98),(1.02040816326531))*exp(-0.176*ore_grade2) + 17.699773549772*IF((ore_grade2 < 0.01),1/(-0.0723*LN(100*ore_grade2)^2/LN(10)^2 + 0.98),(1.02040816326531))*exp(-0.176*ore_grade2) + 95.8023759060552)/(1.0 - 1.0*share_ISL2) + 389.532968103831 + 0.0258*(0.170495603413036*milling_electricity_switch_dieselgenerator2 + 0.10274921860281*milling_electricity_switch_grid2)/ore_grade2 + 0.149808252120606/ore_grade2) + 58.6211867812304)/(1.219800704306 + 0.0049*(2*rate_enrichment2 - 1)*LN(rate_enrichment2/(1 - rate_enrichment2))/(rate_enrichment2 - 0.0022) - 0.0298418833469335/(rate_enrichment2 - 0.0022))) + enrichment_mix_switch_diffusion2*(0.65711431644277*enrichment_diff_elec2 + 100.203906410342 + (0.577795780936465*conversion_elec2 + 0.0159469405318573*conversion_heat2 + 1.04217599120411*share_ISL2*(154.0*(0.170495603413036*mining_electricity_switch_dieselgenerator2 + 0.10274921860281*mining_electricity_switch_grid2)*IF((ore_grade2 < 0.01),1/(-0.0723*LN(100*ore_grade2)^2/LN(10)^2 + 0.98),(1.02040816326531))*exp(-0.0485*ore_grade2) + 12.2837545140374*IF((ore_grade2 < 0.01),1/(-0.0723*LN(100*ore_grade2)^2/LN(10)^2 + 0.98),(1.02040816326531))*exp(-0.0485*ore_grade2) + 328.371731950703) + 1.04217599120411*(1.0 - 1.0*share_ISL2)*((0.445983379501385 - 0.445983379501385*share_ISL2)*(191.8*(0.170495603413036*mining_electricity_switch_dieselgenerator2 + 0.10274921860281*mining_electricity_switch_grid2)*IF((ore_grade2 < 0.01),1/(-0.0723*LN(100*ore_grade2)^2/LN(10)^2 + 0.98),(1.02040816326531))*exp(-0.482*ore_grade2) + 15.2988578947556*IF((ore_grade2 < 0.01),1/(-0.0723*LN(100*ore_grade2)^2/LN(10)^2 + 0.98),(1.02040816326531))*exp(-0.482*ore_grade2) + 251.907876301475)/(1.0 - 1.0*share_ISL2) + (0.554016620498615 - 0.554016620498615*share_ISL2)*(221.9*(0.170495603413036*mining_electricity_switch_dieselgenerator2 + 0.10274921860281*mining_electricity_switch_grid2)*IF((ore_grade2 < 0.01),1/(-0.0723*LN(100*ore_grade2)^2/LN(10)^2 + 0.98),(1.02040816326531))*exp(-0.176*ore_grade2) + 17.699773549772*IF((ore_grade2 < 0.01),1/(-0.0723*LN(100*ore_grade2)^2/LN(10)^2 + 0.98),(1.02040816326531))*exp(-0.176*ore_grade2) + 95.8023759060552)/(1.0 - 1.0*share_ISL2) + 389.532968103831 + 0.0258*(0.170495603413036*milling_electricity_switch_dieselgenerator2 + 0.10274921860281*milling_electricity_switch_grid2)/ore_grade2 + 0.149808252120606/ore_grade2) + 58.6211867812304)/(1.219800704306 + 0.0049*(2*rate_enrichment2 - 1)*LN(rate_enrichment2/(1 - rate_enrichment2))/(rate_enrichment2 - 0.0022) - 0.0298418833469335/(rate_enrichment2 - 0.0022))))*(1.219800704306*rate_enrichment2 + 0.0049*(2*rate_enrichment2 - 1)*LN(-rate_enrichment2/(rate_enrichment2 - 1)) - 0.0325254448964067) + 72.8574885027346)/(24000*efficiency2*(1090.0*rate_enrichment2 - 0.600000000000001)) + 1.14077116130504e-10*(1206150153.65082*construction_intensity2 + 1592990884.93782)/(availability2*lifetime2)',
 ('EF v3.0',
  'material resources: metals/minerals',
  'abiotic depletion potential (ADP): elements (ultimate reserves)'): '=2.24900185004116e-7 + (7.13213946722281e-7*fuel_fab_elec2 + 202.040816326531*(enrichment_mix_switch_centrifugation2*(4.20062055071576e-7*enrichment_centr_elec2 + 0.000348280534143669 + (3.46085741679439e-7*conversion_elec2 + 3.49410958910921e-8*conversion_heat2 + 1.04217599120411*share_ISL2*(154.0*(7.4461463189856e-8*mining_electricity_switch_dieselgenerator2 + 2.67850732833362e-7*mining_electricity_switch_grid2)*IF((ore_grade2 < 0.01),1/(-0.0723*LN(100*ore_grade2)^2/LN(10)^2 + 0.98),(1.02040816326531))*exp(-0.0485*ore_grade2) + 4.17716495157629e-6*IF((ore_grade2 < 0.01),1/(-0.0723*LN(100*ore_grade2)^2/LN(10)^2 + 0.98),(1.02040816326531))*exp(-0.0485*ore_grade2) + 0.00766589258638336) + 1.04217599120411*(1.0 - 1.0*share_ISL2)*((0.445983379501385 - 0.445983379501385*share_ISL2)*(191.8*(7.4461463189856e-8*mining_electricity_switch_dieselgenerator2 + 2.67850732833362e-7*mining_electricity_switch_grid2)*IF((ore_grade2 < 0.01),1/(-0.0723*LN(100*ore_grade2)^2/LN(10)^2 + 0.98),(1.02040816326531))*exp(-0.482*ore_grade2) + 5.20246907605411e-6*IF((ore_grade2 < 0.01),1/(-0.0723*LN(100*ore_grade2)^2/LN(10)^2 + 0.98),(1.02040816326531))*exp(-0.482*ore_grade2) + 0.000136175813656906)/(1.0 - 1.0*share_ISL2) + (0.554016620498615 - 0.554016620498615*share_ISL2)*(221.9*(7.4461463189856e-8*mining_electricity_switch_dieselgenerator2 + 2.67850732833362e-7*mining_electricity_switch_grid2)*IF((ore_grade2 < 0.01),1/(-0.0723*LN(100*ore_grade2)^2/LN(10)^2 + 0.98),(1.02040816326531))*exp(-0.176*ore_grade2) + 6.01891495295312e-6*IF((ore_grade2 < 0.01),1/(-0.0723*LN(100*ore_grade2)^2/LN(10)^2 + 0.98),(1.02040816326531))*exp(-0.176*ore_grade2) + 0.000145280896650256)/(1.0 - 1.0*share_ISL2) + 0.00125982785229094 + 0.0258*(7.4461463189856e-8*milling_electricity_switch_dieselgenerator2 + 2.67850732833362e-7*milling_electricity_switch_grid2)/ore_grade2 + 3.76042594876852e-8/ore_grade2) + 0.000418106961908681)/(1.219800704306 + 0.0049*(2*rate_enrichment2 - 1)*LN(rate_enrichment2/(1 - rate_enrichment2))/(rate_enrichment2 - 0.0022) - 0.0298418833469335/(rate_enrichment2 - 0.0022))) + enrichment_mix_switch_diffusion2*(4.20062055071576e-7*enrichment_diff_elec2 + 0.000611926843370239 + (3.46085741679439e-7*conversion_elec2 + 3.49410958910921e-8*conversion_heat2 + 1.04217599120411*share_ISL2*(154.0*(7.4461463189856e-8*mining_electricity_switch_dieselgenerator2 + 2.67850732833362e-7*mining_electricity_switch_grid2)*IF((ore_grade2 < 0.01),1/(-0.0723*LN(100*ore_grade2)^2/LN(10)^2 + 0.98),(1.02040816326531))*exp(-0.0485*ore_grade2) + 4.17716495157629e-6*IF((ore_grade2 < 0.01),1/(-0.0723*LN(100*ore_grade2)^2/LN(10)^2 + 0.98),(1.02040816326531))*exp(-0.0485*ore_grade2) + 0.00766589258638336) + 1.04217599120411*(1.0 - 1.0*share_ISL2)*((0.445983379501385 - 0.445983379501385*share_ISL2)*(191.8*(7.4461463189856e-8*mining_electricity_switch_dieselgenerator2 + 2.67850732833362e-7*mining_electricity_switch_grid2)*IF((ore_grade2 < 0.01),1/(-0.0723*LN(100*ore_grade2)^2/LN(10)^2 + 0.98),(1.02040816326531))*exp(-0.482*ore_grade2) + 5.20246907605411e-6*IF((ore_grade2 < 0.01),1/(-0.0723*LN(100*ore_grade2)^2/LN(10)^2 + 0.98),(1.02040816326531))*exp(-0.482*ore_grade2) + 0.000136175813656906)/(1.0 - 1.0*share_ISL2) + (0.554016620498615 - 0.554016620498615*share_ISL2)*(221.9*(7.4461463189856e-8*mining_electricity_switch_dieselgenerator2 + 2.67850732833362e-7*mining_electricity_switch_grid2)*IF((ore_grade2 < 0.01),1/(-0.0723*LN(100*ore_grade2)^2/LN(10)^2 + 0.98),(1.02040816326531))*exp(-0.176*ore_grade2) + 6.01891495295312e-6*IF((ore_grade2 < 0.01),1/(-0.0723*LN(100*ore_grade2)^2/LN(10)^2 + 0.98),(1.02040816326531))*exp(-0.176*ore_grade2) + 0.000145280896650256)/(1.0 - 1.0*share_ISL2) + 0.00125982785229094 + 0.0258*(7.4461463189856e-8*milling_electricity_switch_dieselgenerator2 + 2.67850732833362e-7*milling_electricity_switch_grid2)/ore_grade2 + 3.76042594876852e-8/ore_grade2) + 0.000418106961908681)/(1.219800704306 + 0.0049*(2*rate_enrichment2 - 1)*LN(rate_enrichment2/(1 - rate_enrichment2))/(rate_enrichment2 - 0.0022) - 0.0298418833469335/(rate_enrichment2 - 0.0022))))*(1.219800704306*rate_enrichment2 + 0.0049*(2*rate_enrichment2 - 1)*LN(-rate_enrichment2/(rate_enrichment2 - 1)) - 0.0325254448964067) + 0.000662018850933288)/(24000*efficiency2*(1090.0*rate_enrichment2 - 0.600000000000001)) + 1.14077116130504e-10*(18198.9116208585*construction_intensity2 + 44.2180479090089)/(availability2*lifetime2)',
 ('EF v3.0',
  'water use',
  'user deprivation potential (deprivation-weighted water consumption)'): '=0.0987850017547607*river_cooling2 + 0.000576887378348076 + (0.120297151746396*fuel_fab_elec2 + 202.040816326531*(enrichment_mix_switch_centrifugation2*(0.128354031325489*enrichment_centr_elec2 + 2.5432218290506 + (0.29298941455146*conversion_elec2 + 0.00237639492616074*conversion_heat2 + 1.04217599120411*share_ISL2*(154.0*(0.00298224311975356*mining_electricity_switch_dieselgenerator2 + 0.112705922350704*mining_electricity_switch_grid2)*IF((ore_grade2 < 0.01),1/(-0.0723*LN(100*ore_grade2)^2/LN(10)^2 + 0.98),(1.02040816326531))*exp(-0.0485*ore_grade2) + 0.309108654872383*IF((ore_grade2 < 0.01),1/(-0.0723*LN(100*ore_grade2)^2/LN(10)^2 + 0.98),(1.02040816326531))*exp(-0.0485*ore_grade2) + 95.7069888820048) + 1.04217599120411*(1.0 - 1.0*share_ISL2)*((0.445983379501385 - 0.445983379501385*share_ISL2)*(191.8*(0.00298224311975356*mining_electricity_switch_dieselgenerator2 + 0.112705922350704*mining_electricity_switch_grid2)*IF((ore_grade2 < 0.01),1/(-0.0723*LN(100*ore_grade2)^2/LN(10)^2 + 0.98),(1.02040816326531))*exp(-0.482*ore_grade2) + 0.38498077925015*IF((ore_grade2 < 0.01),1/(-0.0723*LN(100*ore_grade2)^2/LN(10)^2 + 0.98),(1.02040816326531))*exp(-0.482*ore_grade2) + 41.7998217585077)/(1.0 - 1.0*share_ISL2) + (0.554016620498615 - 0.554016620498615*share_ISL2)*(221.9*(0.00298224311975356*mining_electricity_switch_dieselgenerator2 + 0.112705922350704*mining_electricity_switch_grid2)*IF((ore_grade2 < 0.01),1/(-0.0723*LN(100*ore_grade2)^2/LN(10)^2 + 0.98),(1.02040816326531))*exp(-0.176*ore_grade2) + 0.445397470884298*IF((ore_grade2 < 0.01),1/(-0.0723*LN(100*ore_grade2)^2/LN(10)^2 + 0.98),(1.02040816326531))*exp(-0.176*ore_grade2) + 29.8451224584082)/(1.0 - 1.0*share_ISL2) + 52.7642935163433 + 0.0258*(0.00298224311975356*milling_electricity_switch_dieselgenerator2 + 0.112705922350704*milling_electricity_switch_grid2)/ore_grade2 + 0.00371804927536702/ore_grade2) + 18.3907182562588)/(1.219800704306 + 0.0049*(2*rate_enrichment2 - 1)*LN(rate_enrichment2/(1 - rate_enrichment2))/(rate_enrichment2 - 0.0022) - 0.0298418833469335/(rate_enrichment2 - 0.0022))) + enrichment_mix_switch_diffusion2*(0.128354031325489*enrichment_diff_elec2 + 71.3528214141035 + (0.29298941455146*conversion_elec2 + 0.00237639492616074*conversion_heat2 + 1.04217599120411*share_ISL2*(154.0*(0.00298224311975356*mining_electricity_switch_dieselgenerator2 + 0.112705922350704*mining_electricity_switch_grid2)*IF((ore_grade2 < 0.01),1/(-0.0723*LN(100*ore_grade2)^2/LN(10)^2 + 0.98),(1.02040816326531))*exp(-0.0485*ore_grade2) + 0.309108654872383*IF((ore_grade2 < 0.01),1/(-0.0723*LN(100*ore_grade2)^2/LN(10)^2 + 0.98),(1.02040816326531))*exp(-0.0485*ore_grade2) + 95.7069888820048) + 1.04217599120411*(1.0 - 1.0*share_ISL2)*((0.445983379501385 - 0.445983379501385*share_ISL2)*(191.8*(0.00298224311975356*mining_electricity_switch_dieselgenerator2 + 0.112705922350704*mining_electricity_switch_grid2)*IF((ore_grade2 < 0.01),1/(-0.0723*LN(100*ore_grade2)^2/LN(10)^2 + 0.98),(1.02040816326531))*exp(-0.482*ore_grade2) + 0.38498077925015*IF((ore_grade2 < 0.01),1/(-0.0723*LN(100*ore_grade2)^2/LN(10)^2 + 0.98),(1.02040816326531))*exp(-0.482*ore_grade2) + 41.7998217585077)/(1.0 - 1.0*share_ISL2) + (0.554016620498615 - 0.554016620498615*share_ISL2)*(221.9*(0.00298224311975356*mining_electricity_switch_dieselgenerator2 + 0.112705922350704*mining_electricity_switch_grid2)*IF((ore_grade2 < 0.01),1/(-0.0723*LN(100*ore_grade2)^2/LN(10)^2 + 0.98),(1.02040816326531))*exp(-0.176*ore_grade2) + 0.445397470884298*IF((ore_grade2 < 0.01),1/(-0.0723*LN(100*ore_grade2)^2/LN(10)^2 + 0.98),(1.02040816326531))*exp(-0.176*ore_grade2) + 29.8451224584082)/(1.0 - 1.0*share_ISL2) + 52.7642935163433 + 0.0258*(0.00298224311975356*milling_electricity_switch_dieselgenerator2 + 0.112705922350704*milling_electricity_switch_grid2)/ore_grade2 + 0.00371804927536702/ore_grade2) + 18.3907182562588)/(1.219800704306 + 0.0049*(2*rate_enrichment2 - 1)*LN(rate_enrichment2/(1 - rate_enrichment2))/(rate_enrichment2 - 0.0022) - 0.0298418833469335/(rate_enrichment2 - 0.0022))))*(1.219800704306*rate_enrichment2 + 0.0049*(2*rate_enrichment2 - 1)*LN(-rate_enrichment2/(rate_enrichment2 - 1)) - 0.0325254448964067) + 14.2908542532349)/(24000*efficiency2*(1090.0*rate_enrichment2 - 0.600000000000001)) + 1.14077116130504e-10*(129846906.578373*construction_intensity2 + 1089640.83194551)/(availability2*lifetime2)'}
```

In [45]:

```
pd.Series(formulas2).to_csv('output/nuclear_scenario2.csv')
```

In [46]:

```
# Setting 100% centrifugation
enrichment_mix_switch.distrib = DistributionType.FIXED
enrichment_mix_switch.default = 'centrifugation'
```

In [63]:

```
# Revert back, switch to choose enrichment technique
enrichment_mix_switch = newEnumParam(
    'enrichment_mix_switch',
    label='Enrichment technology',
    values={'centrifugation':0.8,
            'diffusion':0.2},
    default='centrifugation',
    dbname='Nuclear_DB')

# Bins are not properly built, so we do it here 
enrichment_mix_switch._bins = [0]
for i in range(len(enrichment_mix_switch.values)) :
    enumvalue = enrichment_mix_switch.values[i]
    enrichment_mix_switch._bins.append(enrichment_mix_switch._bins[i] + enrichment_mix_switch.weights[enumvalue])
```

```
[ParamRegistry] Param enrichment_mix_switch was already defined in 'Nuclear_DB' : overriding.
```

## Scenario analysis¶

In this section, we load a set of scenarios, either one-at-a-time or combinations of changing variables, to explore the potential range of LCIA results.

In [64]:

```
scenarios = pd.read_excel('data/nuclear_scenario_data.xlsx', index_col=0, header=0)
scenarios.drop('Group', inplace=True, axis=1)
```

In [65]:

```
scenario_names  = scenarios.columns
scenario_values = scenarios.T.to_dict(orient='list')

parameters = [params._param_name(param, NameType.NAME) for param in params._param_registry().all()]
```

In [66]:

```
scenarios_filtered = scenarios.loc[scenarios.index & parameters]
scenarios_dict = scenarios_filtered.T.to_dict(orient='list')
```

```
C:\Users\Gibon\AppData\Local\Temp\ipykernel_28944\1634600665.py:1: FutureWarning: Index.__and__ operating as a set operation is deprecated, in the future this will be a logical operation matching Series.__and__.  Use index.intersection(other) instead.
  scenarios_filtered = scenarios.loc[scenarios.index & parameters]
```

In [67]:

```
d = multiLCAAlgebric(elec_prod_p, # The model 
    impacts, # Impacts
    **scenarios_dict
)
```

```
Param 'rate_feed' is marked as FIXED, but passed in parameters : ignored
Param 'rate_tailings' is marked as FIXED, but passed in parameters : ignored
Param 'capacity' is marked as FIXED, but passed in parameters : ignored
```

In [68]:

```
d['water use - user deprivation potential (deprivation-weighted water consumption)[m3 world eq. deprived]'] /= 42.95 # conversion from global m3 to raw liters
```

In [69]:

```
d.index=scenario_names
```

In [70]:

```
d_norm = d / d.max()
```

In [71]:

```
d.columns
```

Out[71]:

```
Index(['climate change - global warming potential (GWP100)[kg CO2-Eq]',
       'ecotoxicity: freshwater - comparative toxic unit for ecosystems (CTUe) [CTUe]',
       'eutrophication: freshwater - fraction of nutrients reaching freshwater end compartment (P)[kg PO4-Eq]',
       'human toxicity: carcinogenic - comparative toxic unit for human (CTUh) [CTUh]',
       'human toxicity: non-carcinogenic - comparative toxic unit for human (CTUh) [CTUh]',
       'ionising radiation: human health - human exposure efficiency relative to u235[kBq U235-Eq]',
       'land use - soil quality index[dimensionless]',
       'material resources: metals/minerals - abiotic depletion potential (ADP): elements (ultimate reserves)[kg Sb-Eq]',
       'water use - user deprivation potential (deprivation-weighted water consumption)[m3 world eq. deprived]'],
      dtype='object')
```

In [72]:

```
ind_norm = ['climate change - global warming potential (GWP100) [g CO2-Eq]',
       'ecotoxicity: freshwater - comparative toxic unit for ecosystems (CTUe) [CTUe]',
       'eutrophication: freshwater - fraction of nutrients reaching freshwater end compartment (P) [mg PO4-Eq]',
       'human toxicity: carcinogenic - comparative toxic unit for human (CTUh) [10$^{-12}$ CTUh]',
       'human toxicity: non-carcinogenic - comparative toxic unit for human (CTUh) [10$^{-12}$ CTUh]',
       'ionising radiation: human health - human exposure efficiency relative to u235 [Bq U235-Eq]',
       'land use - soil quality index [10$^{-3}$]',
       'material resources: metals/minerals - abiotic depletion potential (ADP): elements (ultimate reserves) [μg Sb-Eq]',
       'water use - user deprivation potential (deprivation-weighted water consumption) [l world eq. deprived]']
ind_norm_wrap = [ind.replace(' - ','\n').replace(' [','\n[') for ind in ind_norm]
ind_norm_wrap_short = ['\n'.join(ind.split('\n')[i] for i in [0,2]) for ind in ind_norm_wrap]
```

In [73]:

```
# Scaling scores to the right amount

scales = [1e3, 1, 1e6, 1e12, 1e12, 1e3, 1e3, 1e9, 1e3]
d_scaled = d*scales
d_scaled.columns = ind_norm_wrap_short
d_norm.columns = ind_norm_wrap_short
```

In [74]:

```
def str_sigfig(x, sf):
    x_sf = round(x,sf)
    digits_before_dot = np.ceil(np.log10(x))
    if digits_before_dot<=sf:
        digits_after_dot = int(sf - digits_before_dot)
        return f'{x_sf:.{digits_after_dot}f}'
    else:
        return f'{round(x_sf, int(3-digits_before_dot)):.0f}'
```

In [75]:

```
d_formatted = d_scaled.applymap(lambda x: str_sigfig(x,3))
```

In [76]:

```
d_norm.shape
```

Out[76]:

```
(22, 9)
```

In [77]:

```
sns.set(font_scale=1)
fig, ax = plt.subplots(figsize=(16,9))
sns.heatmap(d_norm, cmap='Oranges', annot=d_formatted, fmt='')

ax.add_patch(Rectangle((0, 0), 9, 1, edgecolor='black', fill=False, lw=1))
ax.add_patch(Rectangle((0, 20), 9, 2, edgecolor='black', fill=False, lw=1))

plt.tight_layout()
plt.savefig('output/parameter_analysis.svg')
plt.savefig('output/parameter_analysis.png')
```

## Dashboard¶

In [ ]:

```
oat_dashboard_interact(elec_prod_p, impacts, n=2000)
```

## Sensitivity to ore grade¶

In [92]:

```
# Setting 100% diffusion
enrichment_mix_switch.distrib = DistributionType.FIXED
enrichment_mix_switch.default = 'centrifugation'
```

In [93]:

```
n=2000
varying_param=ore_grade
range_param=varying_param.range(n)

# if log
range_param=[np.log10(g) for g in np.logspace(varying_param.min,varying_param.max,num=n)]

all_param_names = params._param_registry().keys()
param_values = {name: params._param_registry()[name].default for name in all_param_names}
param_values[varying_param.name] = range_param
ore_grade_sensitivity = multiLCAAlgebric(elec_prod_p, impacts, **param_values)

pname = varying_param.name
pname = '%s [%s]' % (pname, varying_param.unit)

ore_grade_sensitivity.insert(0, pname, range_param)
ore_grade_sensitivity = ore_grade_sensitivity.set_index(pname)

ore_grade_sensitivity['water use - user deprivation potential (deprivation-weighted water consumption)[m3 world eq. deprived]'] /= 42.95 # conversion from global m3 to raw liters
```

```
Param 'rate_feed' is marked as FIXED, but passed in parameters : ignored
Param 'rate_tailings' is marked as FIXED, but passed in parameters : ignored
Param 'capacity' is marked as FIXED, but passed in parameters : ignored
Param 'enrichment_mix_switch' is marked as FIXED, but passed in parameters : ignored
```

In [94]:

```
# Setting 100% diffusion
enrichment_mix_switch.distrib = DistributionType.FIXED
enrichment_mix_switch.default = 'diffusion'
```

In [95]:

```
all_param_names = params._param_registry().keys()
param_values = {name: params._param_registry()[name].default for name in all_param_names}
param_values[varying_param.name] = range_param
ore_grade_sensitivity_diff = multiLCAAlgebric(elec_prod_p, impacts, **param_values)

pname = varying_param.name
pname = '%s [%s]' % (pname, varying_param.unit)

ore_grade_sensitivity_diff.insert(0, pname, range_param)
ore_grade_sensitivity_diff = ore_grade_sensitivity_diff.set_index(pname)

ore_grade_sensitivity_diff['water use - user deprivation potential (deprivation-weighted water consumption)[m3 world eq. deprived]'] /= 42.95 # conversion from global m3 to raw liters
```

```
Param 'rate_feed' is marked as FIXED, but passed in parameters : ignored
Param 'rate_tailings' is marked as FIXED, but passed in parameters : ignored
Param 'capacity' is marked as FIXED, but passed in parameters : ignored
Param 'enrichment_mix_switch' is marked as FIXED, but passed in parameters : ignored
```

In [96]:

```
sns.set(font_scale=.8)
fig, axes = plt.subplots(3,3,figsize=(16,9))

delta = ore_grade_sensitivity_diff-ore_grade_sensitivity

for i,impact in enumerate(ore_grade_sensitivity):
    ax=axes[i//3,i%3]
    ore_grade_sensitivity[impact].plot(logx=True, ax=ax)
    ore_grade_sensitivity_diff[impact].plot(logx=True, ax=ax)
    pd.concat([ore_grade_sensitivity[impact], delta[impact]],axis=1).plot.area(ax=ax, color=((0,0,0,0),(.5,.5,.5,.5)),legend=False)
    ax.set_ylabel(ind_norm_wrap_short[i])
    ymin, ymax=ax.get_ylim()
    ax.set_ylim((0,ymax*1.1))
#     ax.legend('')

plt.tight_layout()
```

In [97]:

```
# Revert back, switch to choose enrichment technique
enrichment_mix_switch = newEnumParam(
    'enrichment_mix_switch',
    label='Enrichment technology',
    values={'centrifugation':0.8,
            'diffusion':0.2},
    default='centrifugation',
    dbname='Nuclear_DB')

# Bins are not properly built, so we do it here 
enrichment_mix_switch._bins = [0]
for i in range(len(enrichment_mix_switch.values)) :
    enumvalue = enrichment_mix_switch.values[i]
    enrichment_mix_switch._bins.append(enrichment_mix_switch._bins[i] + enrichment_mix_switch.weights[enumvalue])
```

```
[ParamRegistry] Param enrichment_mix_switch was already defined in 'Nuclear_DB' : overriding.
```

## Sensitivity to extraction technique¶

In [113]:

```
# Setting 100% centrifugation
enrichment_mix_switch.distrib = DistributionType.FIXED
enrichment_mix_switch.default = 'centrifugation'
```

In [114]:

```
n=2000
varying_param=share_ISL
range_param=varying_param.range(n)

# if log
range_param=[np.log10(g) for g in np.logspace(varying_param.min,varying_param.max,num=n)]

all_param_names = params._param_registry().keys()
param_values = {name: params._param_registry()[name].default for name in all_param_names}
param_values[varying_param.name] = range_param
sensitivity = multiLCAAlgebric(elec_prod_p, impacts, **param_values)

pname = varying_param.name
pname = '%s [%s]' % (pname, varying_param.unit)

sensitivity.insert(0, pname, range_param)
sensitivity = sensitivity.set_index(pname)

sensitivity['water use - user deprivation potential (deprivation-weighted water consumption)[m3 world eq. deprived]'] /= 42.95 # conversion from global m3 to raw liters
```

```
Param 'rate_feed' is marked as FIXED, but passed in parameters : ignored
Param 'rate_tailings' is marked as FIXED, but passed in parameters : ignored
Param 'capacity' is marked as FIXED, but passed in parameters : ignored
Param 'enrichment_mix_switch' is marked as FIXED, but passed in parameters : ignored
<lambdifygenerated-522>:2: RuntimeWarning: invalid value encountered in divide
  return 0.00109262104806847 + (1/24000)*(0.396516247200945*fuel_fab_elec + 202.040816326531*(0.508954163846162*enrichment_centr_elec + 11.2882465408531 + 1.0*(0.424375508389407*conversion_elec + 0.0394776697405151*conversion_heat + 1.04217599120411*share_ISL*(154.0*(0.0892326725829683*mining_electricity_switch_dieselgenerator + 0.177238805770639*mining_electricity_switch_grid)*select([less(ore_grade, 0.01),True], [(-0.0723*log(100*ore_grade)**2/log(10)**2 + 0.98)**(-1.0),1.02040816326531], default=nan)*exp(-0.0485*ore_grade) + 6.15014367236657*select([less(ore_grade, 0.01),True], [(-0.0723*log(100*ore_grade)**2/log(10)**2 + 0.98)**(-1.0),1.02040816326531], default=nan)*exp(-0.0485*ore_grade) + 70.836795369606) + 1.04217599120411*(1.0 - 1.0*share_ISL)*((0.445983379501385 - 0.445983379501385*share_ISL)*(191.8*(0.0892326725829683*mining_electricity_switch_dieselgenerator + 0.177238805770639*mining_electricity_switch_grid)*select([less(ore_grade, 0.01),True], [(-0.0723*log(100*ore_grade)**2/log(10)**2 + 0.98)**(-1.0),1.02040816326531], default=nan)*exp(-0.482*ore_grade) + 7.65972439194745*select([less(ore_grade, 0.01),True], [(-0.0723*log(100*ore_grade)**2/log(10)**2 + 0.98)**(-1.0),1.02040816326531], default=nan)*exp(-0.482*ore_grade) + 7.78564641515561)/(1.0 - 1.0*share_ISL) + (0.554016620498615 - 0.554016620498615*share_ISL)*(221.9*(0.0892326725829683*mining_electricity_switch_dieselgenerator + 0.177238805770639*mining_electricity_switch_grid)*select([less(ore_grade, 0.01),True], [(-0.0723*log(100*ore_grade)**2/log(10)**2 + 0.98)**(-1.0),1.02040816326531], default=nan)*exp(-0.176*ore_grade) + 8.86179792791*select([less(ore_grade, 0.01),True], [(-0.0723*log(100*ore_grade)**2/log(10)**2 + 0.98)**(-1.0),1.02040816326531], default=nan)*exp(-0.176*ore_grade) + 73.1369315092323)/(1.0 - 1.0*share_ISL) + 26.0972429849788 + 0.0258*(0.0892326725829683*milling_electricity_switch_dieselgenerator + 0.177238805770639*milling_electricity_switch_grid)/ore_grade + 0.087818031245362/ore_grade) + 20.7085137101814)/(1.219800704306 + 0.0049*(2*rate_enrichment - 1)*log(rate_enrichment/(1 - rate_enrichment))/(rate_enrichment - 0.0022) - 0.0298418833469335/(rate_enrichment - 0.0022)))*(1.219800704306*rate_enrichment + 0.0049*(2*rate_enrichment - 1)*log(-rate_enrichment/(rate_enrichment - 1)) - 0.0325254448964067) + 26.6263832957925)/(efficiency*(1090.0*rate_enrichment - 0.600000000000001)) + 1.14077116130504e-10*(344721009.42676*construction_intensity + 19136892.1567673)/(availability*lifetime)
<lambdifygenerated-524>:2: RuntimeWarning: invalid value encountered in divide
  return 1.35045231125469e-6 + (1/24000)*(0.000293608271994933*fuel_fab_elec + 202.040816326531*(0.000209415607336275*enrichment_centr_elec + 0.00354906231621896 + 1.0*(0.000159576915665382*conversion_elec + 6.97111131045668e-7*conversion_heat + 1.04217599120411*share_ISL*(154.0*(2.49548981979895e-6*mining_electricity_switch_dieselgenerator + 0.000156389768554889*mining_electricity_switch_grid)*select([less(ore_grade, 0.01),True], [(-0.0723*log(100*ore_grade)**2/log(10)**2 + 0.98)**(-1.0),1.02040816326531], default=nan)*exp(-0.0485*ore_grade) + 0.000279931131473367*select([less(ore_grade, 0.01),True], [(-0.0723*log(100*ore_grade)**2/log(10)**2 + 0.98)**(-1.0),1.02040816326531], default=nan)*exp(-0.0485*ore_grade) + 0.149712701221427) + 1.04217599120411*(1.0 - 1.0*share_ISL)*((0.445983379501385 - 0.445983379501385*share_ISL)*(191.8*(2.49548981979895e-6*mining_electricity_switch_dieselgenerator + 0.000156389768554889*mining_electricity_switch_grid)*select([less(ore_grade, 0.01),True], [(-0.0723*log(100*ore_grade)**2/log(10)**2 + 0.98)**(-1.0),1.02040816326531], default=nan)*exp(-0.482*ore_grade) + 0.000348641500107739*select([less(ore_grade, 0.01),True], [(-0.0723*log(100*ore_grade)**2/log(10)**2 + 0.98)**(-1.0),1.02040816326531], default=nan)*exp(-0.482*ore_grade) + 0.00175570038495523)/(1.0 - 1.0*share_ISL) + (0.554016620498615 - 0.554016620498615*share_ISL)*(221.9*(2.49548981979895e-6*mining_electricity_switch_dieselgenerator + 0.000156389768554889*mining_electricity_switch_grid)*select([less(ore_grade, 0.01),True], [(-0.0723*log(100*ore_grade)**2/log(10)**2 + 0.98)**(-1.0),1.02040816326531], default=nan)*exp(-0.176*ore_grade) + 0.000403355312168442*select([less(ore_grade, 0.01),True], [(-0.0723*log(100*ore_grade)**2/log(10)**2 + 0.98)**(-1.0),1.02040816326531], default=nan)*exp(-0.176*ore_grade) + 0.045933022734742)/(1.0 - 1.0*share_ISL) + 0.043796778416286 + 0.0258*(2.49548981979895e-6*milling_electricity_switch_dieselgenerator + 0.000156389768554889*milling_electricity_switch_grid)/ore_grade + 2.41110340403162e-5/ore_grade) + 0.0160040374521204)/(1.219800704306 + 0.0049*(2*rate_enrichment - 1)*log(rate_enrichment/(1 - rate_enrichment))/(rate_enrichment - 0.0022) - 0.0298418833469335/(rate_enrichment - 0.0022)))*(1.219800704306*rate_enrichment + 0.0049*(2*rate_enrichment - 1)*log(-rate_enrichment/(rate_enrichment - 1)) - 0.0325254448964067) + 0.0157057220303193)/(efficiency*(1090.0*rate_enrichment - 0.600000000000001)) + 1.14077116130504e-10*(292334.208326767*construction_intensity + 4747.80549402654)/(availability*lifetime)
<lambdifygenerated-525>:2: RuntimeWarning: invalid value encountered in divide
  return 2.69575920297878e-12 + (1/24000)*(9.25613540591443e-11*fuel_fab_elec + 202.040816326531*(8.6704760645527e-11*enrichment_centr_elec + 5.82155935411673e-8 + 1.0*(8.06489843090717e-11*conversion_elec + 4.49751992147394e-12*conversion_heat + 1.04217599120411*share_ISL*(154.0*(7.50585344686937e-12*mining_electricity_switch_dieselgenerator + 4.34773520673846e-11*mining_electricity_switch_grid)*select([less(ore_grade, 0.01),True], [(-0.0723*log(100*ore_grade)**2/log(10)**2 + 0.98)**(-1.0),1.02040816326531], default=nan)*exp(-0.0485*ore_grade) + 1.78959842731656e-9*select([less(ore_grade, 0.01),True], [(-0.0723*log(100*ore_grade)**2/log(10)**2 + 0.98)**(-1.0),1.02040816326531], default=nan)*exp(-0.0485*ore_grade) + 4.54226727177433e-7) + 1.04217599120411*(1.0 - 1.0*share_ISL)*((0.445983379501385 - 0.445983379501385*share_ISL)*(191.8*(7.50585344686937e-12*mining_electricity_switch_dieselgenerator + 4.34773520673846e-11*mining_electricity_switch_grid)*select([less(ore_grade, 0.01),True], [(-0.0723*log(100*ore_grade)**2/log(10)**2 + 0.98)**(-1.0),1.02040816326531], default=nan)*exp(-0.482*ore_grade) + 2.22886349583971e-9*select([less(ore_grade, 0.01),True], [(-0.0723*log(100*ore_grade)**2/log(10)**2 + 0.98)**(-1.0),1.02040816326531], default=nan)*exp(-0.482*ore_grade) + 1.34469505515125e-8)/(1.0 - 1.0*share_ISL) + (0.554016620498615 - 0.554016620498615*share_ISL)*(221.9*(7.50585344686937e-12*mining_electricity_switch_dieselgenerator + 4.34773520673846e-11*mining_electricity_switch_grid)*select([less(ore_grade, 0.01),True], [(-0.0723*log(100*ore_grade)**2/log(10)**2 + 0.98)**(-1.0),1.02040816326531], default=nan)*exp(-0.176*ore_grade) + 2.57864864299704e-9*select([less(ore_grade, 0.01),True], [(-0.0723*log(100*ore_grade)**2/log(10)**2 + 0.98)**(-1.0),1.02040816326531], default=nan)*exp(-0.176*ore_grade) + 3.17772339515592e-8)/(1.0 - 1.0*share_ISL) + 1.24152769103262e-7 + 0.0258*(7.50585344686937e-12*milling_electricity_switch_dieselgenerator + 4.34773520673846e-11*milling_electricity_switch_grid)/ore_grade + 2.11075522201638e-11/ore_grade) + 1.62205237059389e-8)/(1.219800704306 + 0.0049*(2*rate_enrichment - 1)*log(rate_enrichment/(1 - rate_enrichment))/(rate_enrichment - 0.0022) - 0.0298418833469335/(rate_enrichment - 0.0022)))*(1.219800704306*rate_enrichment + 0.0049*(2*rate_enrichment - 1)*log(-rate_enrichment/(rate_enrichment - 1)) - 0.0325254448964067) + 1.45342531624198e-7)/(efficiency*(1090.0*rate_enrichment - 0.600000000000001)) + 1.14077116130504e-10*(0.718683600095208*construction_intensity + 0.00591666055858505)/(availability*lifetime)
<lambdifygenerated-526>:2: RuntimeWarning: invalid value encountered in divide
  return 1.4113154456804e-10 + (1/24000)*(3.47313570607353e-9*fuel_fab_elec + 202.040816326531*(3.22500676888899e-9*enrichment_centr_elec + 3.41285531950546e-7 + 1.0*(3.22114261773152e-9*conversion_elec + 9.8359235959306e-11*conversion_heat + 1.04217599120411*share_ISL*(154.0*(9.87890071579491e-10*mining_electricity_switch_dieselgenerator + 1.91183079402386e-9*mining_electricity_switch_grid)*select([less(ore_grade, 0.01),True], [(-0.0723*log(100*ore_grade)**2/log(10)**2 + 0.98)**(-1.0),1.02040816326531], default=nan)*exp(-0.0485*ore_grade) + 3.67645372505031e-8*select([less(ore_grade, 0.01),True], [(-0.0723*log(100*ore_grade)**2/log(10)**2 + 0.98)**(-1.0),1.02040816326531], default=nan)*exp(-0.0485*ore_grade) + 2.80375253210534e-5) + 1.04217599120411*(1.0 - 1.0*share_ISL)*((0.445983379501385 - 0.445983379501385*share_ISL)*(191.8*(9.87890071579491e-10*mining_electricity_switch_dieselgenerator + 1.91183079402386e-9*mining_electricity_switch_grid)*select([less(ore_grade, 0.01),True], [(-0.0723*log(100*ore_grade)**2/log(10)**2 + 0.98)**(-1.0),1.02040816326531], default=nan)*exp(-0.482*ore_grade) + 4.5788560030172e-8*select([less(ore_grade, 0.01),True], [(-0.0723*log(100*ore_grade)**2/log(10)**2 + 0.98)**(-1.0),1.02040816326531], default=nan)*exp(-0.482*ore_grade) + 7.28410272776341e-7)/(1.0 - 1.0*share_ISL) + (0.554016620498615 - 0.554016620498615*share_ISL)*(221.9*(9.87890071579491e-10*mining_electricity_switch_dieselgenerator + 1.91183079402386e-9*mining_electricity_switch_grid)*select([less(ore_grade, 0.01),True], [(-0.0723*log(100*ore_grade)**2/log(10)**2 + 0.98)**(-1.0),1.02040816326531], default=nan)*exp(-0.176*ore_grade) + 5.29743559473158e-8*select([less(ore_grade, 0.01),True], [(-0.0723*log(100*ore_grade)**2/log(10)**2 + 0.98)**(-1.0),1.02040816326531], default=nan)*exp(-0.176*ore_grade) + 1.22218225897971e-6)/(1.0 - 1.0*share_ISL) + 1.98637636944006e-6 + 0.0258*(9.87890071579491e-10*milling_electricity_switch_dieselgenerator + 1.91183079402386e-9*milling_electricity_switch_grid)/ore_grade + 8.33029606228744e-10/ore_grade) + 4.18600113377616e-7)/(1.219800704306 + 0.0049*(2*rate_enrichment - 1)*log(rate_enrichment/(1 - rate_enrichment))/(rate_enrichment - 0.0022) - 0.0298418833469335/(rate_enrichment - 0.0022)))*(1.219800704306*rate_enrichment + 0.0049*(2*rate_enrichment - 1)*log(-rate_enrichment/(rate_enrichment - 1)) - 0.0325254448964067) + 5.22266326376196e-7)/(efficiency*(1090.0*rate_enrichment - 0.600000000000001)) + 1.14077116130504e-10*(13.8923747590794*construction_intensity + 0.220722951251088)/(availability*lifetime)
<lambdifygenerated-528>:2: RuntimeWarning: invalid value encountered in divide
  return 0.00505692382821041 + (1/24000)*(0.527854381819618*fuel_fab_elec + 202.040816326531*(0.65711431644277*enrichment_centr_elec + 48.9185558569082 + 1.0*(0.577795780936465*conversion_elec + 0.0159469405318573*conversion_heat + 1.04217599120411*share_ISL*(154.0*(0.170495603413036*mining_electricity_switch_dieselgenerator + 0.10274921860281*mining_electricity_switch_grid)*select([less(ore_grade, 0.01),True], [(-0.0723*log(100*ore_grade)**2/log(10)**2 + 0.98)**(-1.0),1.02040816326531], default=nan)*exp(-0.0485*ore_grade) + 12.2837545140374*select([less(ore_grade, 0.01),True], [(-0.0723*log(100*ore_grade)**2/log(10)**2 + 0.98)**(-1.0),1.02040816326531], default=nan)*exp(-0.0485*ore_grade) + 328.371731950703) + 1.04217599120411*(1.0 - 1.0*share_ISL)*((0.445983379501385 - 0.445983379501385*share_ISL)*(191.8*(0.170495603413036*mining_electricity_switch_dieselgenerator + 0.10274921860281*mining_electricity_switch_grid)*select([less(ore_grade, 0.01),True], [(-0.0723*log(100*ore_grade)**2/log(10)**2 + 0.98)**(-1.0),1.02040816326531], default=nan)*exp(-0.482*ore_grade) + 15.2988578947556*select([less(ore_grade, 0.01),True], [(-0.0723*log(100*ore_grade)**2/log(10)**2 + 0.98)**(-1.0),1.02040816326531], default=nan)*exp(-0.482*ore_grade) + 251.907876301475)/(1.0 - 1.0*share_ISL) + (0.554016620498615 - 0.554016620498615*share_ISL)*(221.9*(0.170495603413036*mining_electricity_switch_dieselgenerator + 0.10274921860281*mining_electricity_switch_grid)*select([less(ore_grade, 0.01),True], [(-0.0723*log(100*ore_grade)**2/log(10)**2 + 0.98)**(-1.0),1.02040816326531], default=nan)*exp(-0.176*ore_grade) + 17.699773549772*select([less(ore_grade, 0.01),True], [(-0.0723*log(100*ore_grade)**2/log(10)**2 + 0.98)**(-1.0),1.02040816326531], default=nan)*exp(-0.176*ore_grade) + 95.8023759060552)/(1.0 - 1.0*share_ISL) + 389.532968103831 + 0.0258*(0.170495603413036*milling_electricity_switch_dieselgenerator + 0.10274921860281*milling_electricity_switch_grid)/ore_grade + 0.149808252120606/ore_grade) + 58.6211867812304)/(1.219800704306 + 0.0049*(2*rate_enrichment - 1)*log(rate_enrichment/(1 - rate_enrichment))/(rate_enrichment - 0.0022) - 0.0298418833469335/(rate_enrichment - 0.0022)))*(1.219800704306*rate_enrichment + 0.0049*(2*rate_enrichment - 1)*log(-rate_enrichment/(rate_enrichment - 1)) - 0.0325254448964067) + 72.8574885027346)/(efficiency*(1090.0*rate_enrichment - 0.600000000000001)) + 1.14077116130504e-10*(1206150153.65082*construction_intensity + 1592990884.93782)/(availability*lifetime)
<lambdifygenerated-523>:2: RuntimeWarning: invalid value encountered in divide
  return 0.12944623336283 + (1/24000)*(5.76959136016407*fuel_fab_elec + 202.040816326531*(7.82462592494476*enrichment_centr_elec + 773.904850120356 + 1.0*(8.22318604899191*conversion_elec + 0.209171049040237*conversion_heat + 1.04217599120411*share_ISL*(154.0*(0.684306506985156*mining_electricity_switch_dieselgenerator + 2.53555759688304*mining_electricity_switch_grid)*select([less(ore_grade, 0.01),True], [(-0.0723*log(100*ore_grade)**2/log(10)**2 + 0.98)**(-1.0),1.02040816326531], default=nan)*exp(-0.0485*ore_grade) + 51.7909361866492*select([less(ore_grade, 0.01),True], [(-0.0723*log(100*ore_grade)**2/log(10)**2 + 0.98)**(-1.0),1.02040816326531], default=nan)*exp(-0.0485*ore_grade) + 4632.66129336244) + 1.04217599120411*(1.0 - 1.0*share_ISL)*((0.445983379501385 - 0.445983379501385*share_ISL)*(191.8*(0.684306506985156*mining_electricity_switch_dieselgenerator + 2.53555759688304*mining_electricity_switch_grid)*select([less(ore_grade, 0.01),True], [(-0.0723*log(100*ore_grade)**2/log(10)**2 + 0.98)**(-1.0),1.02040816326531], default=nan)*exp(-0.482*ore_grade) + 64.5032568870085*select([less(ore_grade, 0.01),True], [(-0.0723*log(100*ore_grade)**2/log(10)**2 + 0.98)**(-1.0),1.02040816326531], default=nan)*exp(-0.482*ore_grade) + 44979.121520835)/(1.0 - 1.0*share_ISL) + (0.554016620498615 - 0.554016620498615*share_ISL)*(221.9*(0.684306506985156*mining_electricity_switch_dieselgenerator + 2.53555759688304*mining_electricity_switch_grid)*select([less(ore_grade, 0.01),True], [(-0.0723*log(100*ore_grade)**2/log(10)**2 + 0.98)**(-1.0),1.02040816326531], default=nan)*exp(-0.176*ore_grade) + 74.6260307780354*select([less(ore_grade, 0.01),True], [(-0.0723*log(100*ore_grade)**2/log(10)**2 + 0.98)**(-1.0),1.02040816326531], default=nan)*exp(-0.176*ore_grade) + 11277.5340682197)/(1.0 - 1.0*share_ISL) + 1060.12172794702 + 0.0258*(0.684306506985156*milling_electricity_switch_dieselgenerator + 2.53555759688304*milling_electricity_switch_grid)/ore_grade + 3.20205664804829/ore_grade) + 423.398096119346)/(1.219800704306 + 0.0049*(2*rate_enrichment - 1)*log(rate_enrichment/(1 - rate_enrichment))/(rate_enrichment - 0.0022) - 0.0298418833469335/(rate_enrichment - 0.0022)))*(1.219800704306*rate_enrichment + 0.0049*(2*rate_enrichment - 1)*log(-rate_enrichment/(rate_enrichment - 1)) - 0.0325254448964067) + 857.115119677578)/(efficiency*(1090.0*rate_enrichment - 0.600000000000001)) + 1.14077116130504e-10*(11997059425.2799*construction_intensity + 641114045.030553)/(availability*lifetime)
<lambdifygenerated-527>:2: RuntimeWarning: invalid value encountered in divide
  return 0.000879607210257476 + (1/24000)*(0.120978641909761*fuel_fab_elec + 202.040816326531*(0.208993767959516*enrichment_centr_elec + 0.70201883302818 + 1.0*(0.245406721024908*conversion_elec + 0.00228859202129525*conversion_heat + 1.04217599120411*share_ISL*(154.0*(0.00554785313997973*mining_electricity_switch_dieselgenerator + 0.0453845121725285*mining_electricity_switch_grid)*select([less(ore_grade, 0.01),True], [(-0.0723*log(100*ore_grade)**2/log(10)**2 + 0.98)**(-1.0),1.02040816326531], default=nan)*exp(-0.0485*ore_grade) + 0.404107269334488*select([less(ore_grade, 0.01),True], [(-0.0723*log(100*ore_grade)**2/log(10)**2 + 0.98)**(-1.0),1.02040816326531], default=nan)*exp(-0.0485*ore_grade) + 33.0972116406022) + 1.04217599120411*(1.0 - 1.0*share_ISL)*(3912365.95624308*tailings_Rn222*(1 - exp(-9.19537252003111e-6*integration_time_Rn222)) + (0.445983379501385 - 0.445983379501385*share_ISL)*(191.8*(0.00554785313997973*mining_electricity_switch_dieselgenerator + 0.0453845121725285*mining_electricity_switch_grid)*select([less(ore_grade, 0.01),True], [(-0.0723*log(100*ore_grade)**2/log(10)**2 + 0.98)**(-1.0),1.02040816326531], default=nan)*exp(-0.482*ore_grade) + 0.503297235443862*select([less(ore_grade, 0.01),True], [(-0.0723*log(100*ore_grade)**2/log(10)**2 + 0.98)**(-1.0),1.02040816326531], default=nan)*exp(-0.482*ore_grade) + 178.86950076078)/(1.0 - 1.0*share_ISL) + (0.554016620498615 - 0.554016620498615*share_ISL)*(221.9*(0.00554785313997973*mining_electricity_switch_dieselgenerator + 0.0453845121725285*mining_electricity_switch_grid)*select([less(ore_grade, 0.01),True], [(-0.0723*log(100*ore_grade)**2/log(10)**2 + 0.98)**(-1.0),1.02040816326531], default=nan)*exp(-0.176*ore_grade) + 0.582281838086512*select([less(ore_grade, 0.01),True], [(-0.0723*log(100*ore_grade)**2/log(10)**2 + 0.98)**(-1.0),1.02040816326531], default=nan)*exp(-0.176*ore_grade) + 1161.55454657382)/(1.0 - 1.0*share_ISL) + 4.12632384355364 + 0.0258*(0.00554785313997973*milling_electricity_switch_dieselgenerator + 0.0453845121725285*milling_electricity_switch_grid)/ore_grade + 0.0010411214736684/ore_grade) + 33.0119954271685)/(1.219800704306 + 0.0049*(2*rate_enrichment - 1)*log(rate_enrichment/(1 - rate_enrichment))/(rate_enrichment - 0.0022) - 0.0298418833469335/(rate_enrichment - 0.0022)))*(1.219800704306*rate_enrichment + 0.0049*(2*rate_enrichment - 1)*log(-rate_enrichment/(rate_enrichment - 1)) - 0.0325254448964067) + 24.0670532444177)/(efficiency*(1090.0*rate_enrichment - 0.600000000000001)) + 1.14077116130504e-10*(152034843.173122*construction_intensity + 575589.2492842)/(availability*lifetime)
<lambdifygenerated-529>:2: RuntimeWarning: invalid value encountered in divide
  return 2.24900185004116e-7 + (1/24000)*(7.13213946722281e-7*fuel_fab_elec + 202.040816326531*(4.20062055071576e-7*enrichment_centr_elec + 0.000348280534143669 + 1.0*(3.46085741679439e-7*conversion_elec + 3.49410958910921e-8*conversion_heat + 1.04217599120411*share_ISL*(154.0*(7.4461463189856e-8*mining_electricity_switch_dieselgenerator + 2.67850732833362e-7*mining_electricity_switch_grid)*select([less(ore_grade, 0.01),True], [(-0.0723*log(100*ore_grade)**2/log(10)**2 + 0.98)**(-1.0),1.02040816326531], default=nan)*exp(-0.0485*ore_grade) + 4.17716495157629e-6*select([less(ore_grade, 0.01),True], [(-0.0723*log(100*ore_grade)**2/log(10)**2 + 0.98)**(-1.0),1.02040816326531], default=nan)*exp(-0.0485*ore_grade) + 0.00766589258638336) + 1.04217599120411*(1.0 - 1.0*share_ISL)*((0.445983379501385 - 0.445983379501385*share_ISL)*(191.8*(7.4461463189856e-8*mining_electricity_switch_dieselgenerator + 2.67850732833362e-7*mining_electricity_switch_grid)*select([less(ore_grade, 0.01),True], [(-0.0723*log(100*ore_grade)**2/log(10)**2 + 0.98)**(-1.0),1.02040816326531], default=nan)*exp(-0.482*ore_grade) + 5.20246907605411e-6*select([less(ore_grade, 0.01),True], [(-0.0723*log(100*ore_grade)**2/log(10)**2 + 0.98)**(-1.0),1.02040816326531], default=nan)*exp(-0.482*ore_grade) + 0.000136175813656906)/(1.0 - 1.0*share_ISL) + (0.554016620498615 - 0.554016620498615*share_ISL)*(221.9*(7.4461463189856e-8*mining_electricity_switch_dieselgenerator + 2.67850732833362e-7*mining_electricity_switch_grid)*select([less(ore_grade, 0.01),True], [(-0.0723*log(100*ore_grade)**2/log(10)**2 + 0.98)**(-1.0),1.02040816326531], default=nan)*exp(-0.176*ore_grade) + 6.01891495295312e-6*select([less(ore_grade, 0.01),True], [(-0.0723*log(100*ore_grade)**2/log(10)**2 + 0.98)**(-1.0),1.02040816326531], default=nan)*exp(-0.176*ore_grade) + 0.000145280896650256)/(1.0 - 1.0*share_ISL) + 0.00125982785229094 + 0.0258*(7.4461463189856e-8*milling_electricity_switch_dieselgenerator + 2.67850732833362e-7*milling_electricity_switch_grid)/ore_grade + 3.76042594876852e-8/ore_grade) + 0.000418106961908681)/(1.219800704306 + 0.0049*(2*rate_enrichment - 1)*log(rate_enrichment/(1 - rate_enrichment))/(rate_enrichment - 0.0022) - 0.0298418833469335/(rate_enrichment - 0.0022)))*(1.219800704306*rate_enrichment + 0.0049*(2*rate_enrichment - 1)*log(-rate_enrichment/(rate_enrichment - 1)) - 0.0325254448964067) + 0.000662018850933288)/(efficiency*(1090.0*rate_enrichment - 0.600000000000001)) + 1.14077116130504e-10*(18198.9116208585*construction_intensity + 44.2180479090089)/(availability*lifetime)
<lambdifygenerated-530>:2: RuntimeWarning: invalid value encountered in divide
  return 0.0987850017547607*river_cooling + 0.000576887378348076 + (1/24000)*(0.120297151746396*fuel_fab_elec + 202.040816326531*(0.128354031325489*enrichment_centr_elec + 2.5432218290506 + 1.0*(0.29298941455146*conversion_elec + 0.00237639492616074*conversion_heat + 1.04217599120411*share_ISL*(154.0*(0.00298224311975356*mining_electricity_switch_dieselgenerator + 0.112705922350704*mining_electricity_switch_grid)*select([less(ore_grade, 0.01),True], [(-0.0723*log(100*ore_grade)**2/log(10)**2 + 0.98)**(-1.0),1.02040816326531], default=nan)*exp(-0.0485*ore_grade) + 0.309108654872383*select([less(ore_grade, 0.01),True], [(-0.0723*log(100*ore_grade)**2/log(10)**2 + 0.98)**(-1.0),1.02040816326531], default=nan)*exp(-0.0485*ore_grade) + 95.7069888820048) + 1.04217599120411*(1.0 - 1.0*share_ISL)*((0.445983379501385 - 0.445983379501385*share_ISL)*(191.8*(0.00298224311975356*mining_electricity_switch_dieselgenerator + 0.112705922350704*mining_electricity_switch_grid)*select([less(ore_grade, 0.01),True], [(-0.0723*log(100*ore_grade)**2/log(10)**2 + 0.98)**(-1.0),1.02040816326531], default=nan)*exp(-0.482*ore_grade) + 0.38498077925015*select([less(ore_grade, 0.01),True], [(-0.0723*log(100*ore_grade)**2/log(10)**2 + 0.98)**(-1.0),1.02040816326531], default=nan)*exp(-0.482*ore_grade) + 41.7998217585077)/(1.0 - 1.0*share_ISL) + (0.554016620498615 - 0.554016620498615*share_ISL)*(221.9*(0.00298224311975356*mining_electricity_switch_dieselgenerator + 0.112705922350704*mining_electricity_switch_grid)*select([less(ore_grade, 0.01),True], [(-0.0723*log(100*ore_grade)**2/log(10)**2 + 0.98)**(-1.0),1.02040816326531], default=nan)*exp(-0.176*ore_grade) + 0.445397470884298*select([less(ore_grade, 0.01),True], [(-0.0723*log(100*ore_grade)**2/log(10)**2 + 0.98)**(-1.0),1.02040816326531], default=nan)*exp(-0.176*ore_grade) + 29.8451224584082)/(1.0 - 1.0*share_ISL) + 52.7642935163433 + 0.0258*(0.00298224311975356*milling_electricity_switch_dieselgenerator + 0.112705922350704*milling_electricity_switch_grid)/ore_grade + 0.00371804927536702/ore_grade) + 18.3907182562588)/(1.219800704306 + 0.0049*(2*rate_enrichment - 1)*log(rate_enrichment/(1 - rate_enrichment))/(rate_enrichment - 0.0022) - 0.0298418833469335/(rate_enrichment - 0.0022)))*(1.219800704306*rate_enrichment + 0.0049*(2*rate_enrichment - 1)*log(-rate_enrichment/(rate_enrichment - 1)) - 0.0325254448964067) + 14.2908542532349)/(efficiency*(1090.0*rate_enrichment - 0.600000000000001)) + 1.14077116130504e-10*(129846906.578373*construction_intensity + 1089640.83194551)/(availability*lifetime)
```

In [115]:

```
# Setting 100% diffusion
enrichment_mix_switch.distrib = DistributionType.FIXED
enrichment_mix_switch.default = 'diffusion'
```

In [116]:

```
all_param_names = params._param_registry().keys()
param_values = {name: params._param_registry()[name].default for name in all_param_names}
param_values[varying_param.name] = range_param
sensitivity_diff = multiLCAAlgebric(elec_prod_p, impacts, **param_values)

pname = varying_param.name
pname = '%s [%s]' % (pname, varying_param.unit)

sensitivity_diff.insert(0, pname, range_param)
sensitivity_diff = sensitivity_diff.set_index(pname)

sensitivity_diff['water use - user deprivation potential (deprivation-weighted water consumption)[m3 world eq. deprived]'] /= 42.95 # conversion from global m3 to raw liters
```

```
Param 'rate_feed' is marked as FIXED, but passed in parameters : ignored
Param 'rate_tailings' is marked as FIXED, but passed in parameters : ignored
Param 'capacity' is marked as FIXED, but passed in parameters : ignored
Param 'enrichment_mix_switch' is marked as FIXED, but passed in parameters : ignored
<lambdifygenerated-533>:2: RuntimeWarning: invalid value encountered in divide
  return 1.35045231125469e-6 + (1/24000)*(0.000293608271994933*fuel_fab_elec + 202.040816326531*(0.000209415607336275*enrichment_diff_elec + 0.0174003725428924 + 1.0*(0.000159576915665382*conversion_elec + 6.97111131045668e-7*conversion_heat + 1.04217599120411*share_ISL*(154.0*(2.49548981979895e-6*mining_electricity_switch_dieselgenerator + 0.000156389768554889*mining_electricity_switch_grid)*select([less(ore_grade, 0.01),True], [(-0.0723*log(100*ore_grade)**2/log(10)**2 + 0.98)**(-1.0),1.02040816326531], default=nan)*exp(-0.0485*ore_grade) + 0.000279931131473367*select([less(ore_grade, 0.01),True], [(-0.0723*log(100*ore_grade)**2/log(10)**2 + 0.98)**(-1.0),1.02040816326531], default=nan)*exp(-0.0485*ore_grade) + 0.149712701221427) + 1.04217599120411*(1.0 - 1.0*share_ISL)*((0.445983379501385 - 0.445983379501385*share_ISL)*(191.8*(2.49548981979895e-6*mining_electricity_switch_dieselgenerator + 0.000156389768554889*mining_electricity_switch_grid)*select([less(ore_grade, 0.01),True], [(-0.0723*log(100*ore_grade)**2/log(10)**2 + 0.98)**(-1.0),1.02040816326531], default=nan)*exp(-0.482*ore_grade) + 0.000348641500107739*select([less(ore_grade, 0.01),True], [(-0.0723*log(100*ore_grade)**2/log(10)**2 + 0.98)**(-1.0),1.02040816326531], default=nan)*exp(-0.482*ore_grade) + 0.00175570038495523)/(1.0 - 1.0*share_ISL) + (0.554016620498615 - 0.554016620498615*share_ISL)*(221.9*(2.49548981979895e-6*mining_electricity_switch_dieselgenerator + 0.000156389768554889*mining_electricity_switch_grid)*select([less(ore_grade, 0.01),True], [(-0.0723*log(100*ore_grade)**2/log(10)**2 + 0.98)**(-1.0),1.02040816326531], default=nan)*exp(-0.176*ore_grade) + 0.000403355312168442*select([less(ore_grade, 0.01),True], [(-0.0723*log(100*ore_grade)**2/log(10)**2 + 0.98)**(-1.0),1.02040816326531], default=nan)*exp(-0.176*ore_grade) + 0.045933022734742)/(1.0 - 1.0*share_ISL) + 0.043796778416286 + 0.0258*(2.49548981979895e-6*milling_electricity_switch_dieselgenerator + 0.000156389768554889*milling_electricity_switch_grid)/ore_grade + 2.41110340403162e-5/ore_grade) + 0.0160040374521204)/(1.219800704306 + 0.0049*(2*rate_enrichment - 1)*log(rate_enrichment/(1 - rate_enrichment))/(rate_enrichment - 0.0022) - 0.0298418833469335/(rate_enrichment - 0.0022)))*(1.219800704306*rate_enrichment + 0.0049*(2*rate_enrichment - 1)*log(-rate_enrichment/(rate_enrichment - 1)) - 0.0325254448964067) + 0.0157057220303193)/(efficiency*(1090.0*rate_enrichment - 0.600000000000001)) + 1.14077116130504e-10*(292334.208326767*construction_intensity + 4747.80549402654)/(availability*lifetime)
<lambdifygenerated-531>:2: RuntimeWarning: invalid value encountered in divide
  return 0.00109262104806847 + (1/24000)*(0.396516247200945*fuel_fab_elec + 202.040816326531*(0.508954163846162*enrichment_diff_elec + 28.3616409780363 + 1.0*(0.424375508389407*conversion_elec + 0.0394776697405151*conversion_heat + 1.04217599120411*share_ISL*(154.0*(0.0892326725829683*mining_electricity_switch_dieselgenerator + 0.177238805770639*mining_electricity_switch_grid)*select([less(ore_grade, 0.01),True], [(-0.0723*log(100*ore_grade)**2/log(10)**2 + 0.98)**(-1.0),1.02040816326531], default=nan)*exp(-0.0485*ore_grade) + 6.15014367236657*select([less(ore_grade, 0.01),True], [(-0.0723*log(100*ore_grade)**2/log(10)**2 + 0.98)**(-1.0),1.02040816326531], default=nan)*exp(-0.0485*ore_grade) + 70.836795369606) + 1.04217599120411*(1.0 - 1.0*share_ISL)*((0.445983379501385 - 0.445983379501385*share_ISL)*(191.8*(0.0892326725829683*mining_electricity_switch_dieselgenerator + 0.177238805770639*mining_electricity_switch_grid)*select([less(ore_grade, 0.01),True], [(-0.0723*log(100*ore_grade)**2/log(10)**2 + 0.98)**(-1.0),1.02040816326531], default=nan)*exp(-0.482*ore_grade) + 7.65972439194745*select([less(ore_grade, 0.01),True], [(-0.0723*log(100*ore_grade)**2/log(10)**2 + 0.98)**(-1.0),1.02040816326531], default=nan)*exp(-0.482*ore_grade) + 7.78564641515561)/(1.0 - 1.0*share_ISL) + (0.554016620498615 - 0.554016620498615*share_ISL)*(221.9*(0.0892326725829683*mining_electricity_switch_dieselgenerator + 0.177238805770639*mining_electricity_switch_grid)*select([less(ore_grade, 0.01),True], [(-0.0723*log(100*ore_grade)**2/log(10)**2 + 0.98)**(-1.0),1.02040816326531], default=nan)*exp(-0.176*ore_grade) + 8.86179792791*select([less(ore_grade, 0.01),True], [(-0.0723*log(100*ore_grade)**2/log(10)**2 + 0.98)**(-1.0),1.02040816326531], default=nan)*exp(-0.176*ore_grade) + 73.1369315092323)/(1.0 - 1.0*share_ISL) + 26.0972429849788 + 0.0258*(0.0892326725829683*milling_electricity_switch_dieselgenerator + 0.177238805770639*milling_electricity_switch_grid)/ore_grade + 0.087818031245362/ore_grade) + 20.7085137101814)/(1.219800704306 + 0.0049*(2*rate_enrichment - 1)*log(rate_enrichment/(1 - rate_enrichment))/(rate_enrichment - 0.0022) - 0.0298418833469335/(rate_enrichment - 0.0022)))*(1.219800704306*rate_enrichment + 0.0049*(2*rate_enrichment - 1)*log(-rate_enrichment/(rate_enrichment - 1)) - 0.0325254448964067) + 26.6263832957925)/(efficiency*(1090.0*rate_enrichment - 0.600000000000001)) + 1.14077116130504e-10*(344721009.42676*construction_intensity + 19136892.1567673)/(availability*lifetime)
<lambdifygenerated-534>:2: RuntimeWarning: invalid value encountered in divide
  return 2.69575920297878e-12 + (1/24000)*(9.25613540591443e-11*fuel_fab_elec + 202.040816326531*(8.6704760645527e-11*enrichment_diff_elec + 9.24945276088988e-8 + 1.0*(8.06489843090717e-11*conversion_elec + 4.49751992147394e-12*conversion_heat + 1.04217599120411*share_ISL*(154.0*(7.50585344686937e-12*mining_electricity_switch_dieselgenerator + 4.34773520673846e-11*mining_electricity_switch_grid)*select([less(ore_grade, 0.01),True], [(-0.0723*log(100*ore_grade)**2/log(10)**2 + 0.98)**(-1.0),1.02040816326531], default=nan)*exp(-0.0485*ore_grade) + 1.78959842731656e-9*select([less(ore_grade, 0.01),True], [(-0.0723*log(100*ore_grade)**2/log(10)**2 + 0.98)**(-1.0),1.02040816326531], default=nan)*exp(-0.0485*ore_grade) + 4.54226727177433e-7) + 1.04217599120411*(1.0 - 1.0*share_ISL)*((0.445983379501385 - 0.445983379501385*share_ISL)*(191.8*(7.50585344686937e-12*mining_electricity_switch_dieselgenerator + 4.34773520673846e-11*mining_electricity_switch_grid)*select([less(ore_grade, 0.01),True], [(-0.0723*log(100*ore_grade)**2/log(10)**2 + 0.98)**(-1.0),1.02040816326531], default=nan)*exp(-0.482*ore_grade) + 2.22886349583971e-9*select([less(ore_grade, 0.01),True], [(-0.0723*log(100*ore_grade)**2/log(10)**2 + 0.98)**(-1.0),1.02040816326531], default=nan)*exp(-0.482*ore_grade) + 1.34469505515125e-8)/(1.0 - 1.0*share_ISL) + (0.554016620498615 - 0.554016620498615*share_ISL)*(221.9*(7.50585344686937e-12*mining_electricity_switch_dieselgenerator + 4.34773520673846e-11*mining_electricity_switch_grid)*select([less(ore_grade, 0.01),True], [(-0.0723*log(100*ore_grade)**2/log(10)**2 + 0.98)**(-1.0),1.02040816326531], default=nan)*exp(-0.176*ore_grade) + 2.57864864299704e-9*select([less(ore_grade, 0.01),True], [(-0.0723*log(100*ore_grade)**2/log(10)**2 + 0.98)**(-1.0),1.02040816326531], default=nan)*exp(-0.176*ore_grade) + 3.17772339515592e-8)/(1.0 - 1.0*share_ISL) + 1.24152769103262e-7 + 0.0258*(7.50585344686937e-12*milling_electricity_switch_dieselgenerator + 4.34773520673846e-11*milling_electricity_switch_grid)/ore_grade + 2.11075522201638e-11/ore_grade) + 1.62205237059389e-8)/(1.219800704306 + 0.0049*(2*rate_enrichment - 1)*log(rate_enrichment/(1 - rate_enrichment))/(rate_enrichment - 0.0022) - 0.0298418833469335/(rate_enrichment - 0.0022)))*(1.219800704306*rate_enrichment + 0.0049*(2*rate_enrichment - 1)*log(-rate_enrichment/(rate_enrichment - 1)) - 0.0325254448964067) + 1.45342531624198e-7)/(efficiency*(1090.0*rate_enrichment - 0.600000000000001)) + 1.14077116130504e-10*(0.718683600095208*construction_intensity + 0.00591666055858505)/(availability*lifetime)
<lambdifygenerated-532>:2: RuntimeWarning: invalid value encountered in divide
  return 0.12944623336283 + (1/24000)*(5.76959136016407*fuel_fab_elec + 202.040816326531*(7.82462592494476*enrichment_diff_elec + 612.861809122496 + 1.0*(8.22318604899191*conversion_elec + 0.209171049040237*conversion_heat + 1.04217599120411*share_ISL*(154.0*(0.684306506985156*mining_electricity_switch_dieselgenerator + 2.53555759688304*mining_electricity_switch_grid)*select([less(ore_grade, 0.01),True], [(-0.0723*log(100*ore_grade)**2/log(10)**2 + 0.98)**(-1.0),1.02040816326531], default=nan)*exp(-0.0485*ore_grade) + 51.7909361866492*select([less(ore_grade, 0.01),True], [(-0.0723*log(100*ore_grade)**2/log(10)**2 + 0.98)**(-1.0),1.02040816326531], default=nan)*exp(-0.0485*ore_grade) + 4632.66129336244) + 1.04217599120411*(1.0 - 1.0*share_ISL)*((0.445983379501385 - 0.445983379501385*share_ISL)*(191.8*(0.684306506985156*mining_electricity_switch_dieselgenerator + 2.53555759688304*mining_electricity_switch_grid)*select([less(ore_grade, 0.01),True], [(-0.0723*log(100*ore_grade)**2/log(10)**2 + 0.98)**(-1.0),1.02040816326531], default=nan)*exp(-0.482*ore_grade) + 64.5032568870085*select([less(ore_grade, 0.01),True], [(-0.0723*log(100*ore_grade)**2/log(10)**2 + 0.98)**(-1.0),1.02040816326531], default=nan)*exp(-0.482*ore_grade) + 44979.121520835)/(1.0 - 1.0*share_ISL) + (0.554016620498615 - 0.554016620498615*share_ISL)*(221.9*(0.684306506985156*mining_electricity_switch_dieselgenerator + 2.53555759688304*mining_electricity_switch_grid)*select([less(ore_grade, 0.01),True], [(-0.0723*log(100*ore_grade)**2/log(10)**2 + 0.98)**(-1.0),1.02040816326531], default=nan)*exp(-0.176*ore_grade) + 74.6260307780354*select([less(ore_grade, 0.01),True], [(-0.0723*log(100*ore_grade)**2/log(10)**2 + 0.98)**(-1.0),1.02040816326531], default=nan)*exp(-0.176*ore_grade) + 11277.5340682197)/(1.0 - 1.0*share_ISL) + 1060.12172794702 + 0.0258*(0.684306506985156*milling_electricity_switch_dieselgenerator + 2.53555759688304*milling_electricity_switch_grid)/ore_grade + 3.20205664804829/ore_grade) + 423.398096119346)/(1.219800704306 + 0.0049*(2*rate_enrichment - 1)*log(rate_enrichment/(1 - rate_enrichment))/(rate_enrichment - 0.0022) - 0.0298418833469335/(rate_enrichment - 0.0022)))*(1.219800704306*rate_enrichment + 0.0049*(2*rate_enrichment - 1)*log(-rate_enrichment/(rate_enrichment - 1)) - 0.0325254448964067) + 857.115119677578)/(efficiency*(1090.0*rate_enrichment - 0.600000000000001)) + 1.14077116130504e-10*(11997059425.2799*construction_intensity + 641114045.030553)/(availability*lifetime)
<lambdifygenerated-538>:2: RuntimeWarning: invalid value encountered in divide
  return 2.24900185004116e-7 + (1/24000)*(7.13213946722281e-7*fuel_fab_elec + 202.040816326531*(4.20062055071576e-7*enrichment_diff_elec + 0.000611926843370239 + 1.0*(3.46085741679439e-7*conversion_elec + 3.49410958910921e-8*conversion_heat + 1.04217599120411*share_ISL*(154.0*(7.4461463189856e-8*mining_electricity_switch_dieselgenerator + 2.67850732833362e-7*mining_electricity_switch_grid)*select([less(ore_grade, 0.01),True], [(-0.0723*log(100*ore_grade)**2/log(10)**2 + 0.98)**(-1.0),1.02040816326531], default=nan)*exp(-0.0485*ore_grade) + 4.17716495157629e-6*select([less(ore_grade, 0.01),True], [(-0.0723*log(100*ore_grade)**2/log(10)**2 + 0.98)**(-1.0),1.02040816326531], default=nan)*exp(-0.0485*ore_grade) + 0.00766589258638336) + 1.04217599120411*(1.0 - 1.0*share_ISL)*((0.445983379501385 - 0.445983379501385*share_ISL)*(191.8*(7.4461463189856e-8*mining_electricity_switch_dieselgenerator + 2.67850732833362e-7*mining_electricity_switch_grid)*select([less(ore_grade, 0.01),True], [(-0.0723*log(100*ore_grade)**2/log(10)**2 + 0.98)**(-1.0),1.02040816326531], default=nan)*exp(-0.482*ore_grade) + 5.20246907605411e-6*select([less(ore_grade, 0.01),True], [(-0.0723*log(100*ore_grade)**2/log(10)**2 + 0.98)**(-1.0),1.02040816326531], default=nan)*exp(-0.482*ore_grade) + 0.000136175813656906)/(1.0 - 1.0*share_ISL) + (0.554016620498615 - 0.554016620498615*share_ISL)*(221.9*(7.4461463189856e-8*mining_electricity_switch_dieselgenerator + 2.67850732833362e-7*mining_electricity_switch_grid)*select([less(ore_grade, 0.01),True], [(-0.0723*log(100*ore_grade)**2/log(10)**2 + 0.98)**(-1.0),1.02040816326531], default=nan)*exp(-0.176*ore_grade) + 6.01891495295312e-6*select([less(ore_grade, 0.01),True], [(-0.0723*log(100*ore_grade)**2/log(10)**2 + 0.98)**(-1.0),1.02040816326531], default=nan)*exp(-0.176*ore_grade) + 0.000145280896650256)/(1.0 - 1.0*share_ISL) + 0.00125982785229094 + 0.0258*(7.4461463189856e-8*milling_electricity_switch_dieselgenerator + 2.67850732833362e-7*milling_electricity_switch_grid)/ore_grade + 3.76042594876852e-8/ore_grade) + 0.000418106961908681)/(1.219800704306 + 0.0049*(2*rate_enrichment - 1)*log(rate_enrichment/(1 - rate_enrichment))/(rate_enrichment - 0.0022) - 0.0298418833469335/(rate_enrichment - 0.0022)))*(1.219800704306*rate_enrichment + 0.0049*(2*rate_enrichment - 1)*log(-rate_enrichment/(rate_enrichment - 1)) - 0.0325254448964067) + 0.000662018850933288)/(efficiency*(1090.0*rate_enrichment - 0.600000000000001)) + 1.14077116130504e-10*(18198.9116208585*construction_intensity + 44.2180479090089)/(availability*lifetime)
<lambdifygenerated-535>:2: RuntimeWarning: invalid value encountered in divide
  return 1.4113154456804e-10 + (1/24000)*(3.47313570607353e-9*fuel_fab_elec + 202.040816326531*(3.22500676888899e-9*enrichment_diff_elec + 6.47923811754235e-7 + 1.0*(3.22114261773152e-9*conversion_elec + 9.8359235959306e-11*conversion_heat + 1.04217599120411*share_ISL*(154.0*(9.87890071579491e-10*mining_electricity_switch_dieselgenerator + 1.91183079402386e-9*mining_electricity_switch_grid)*select([less(ore_grade, 0.01),True], [(-0.0723*log(100*ore_grade)**2/log(10)**2 + 0.98)**(-1.0),1.02040816326531], default=nan)*exp(-0.0485*ore_grade) + 3.67645372505031e-8*select([less(ore_grade, 0.01),True], [(-0.0723*log(100*ore_grade)**2/log(10)**2 + 0.98)**(-1.0),1.02040816326531], default=nan)*exp(-0.0485*ore_grade) + 2.80375253210534e-5) + 1.04217599120411*(1.0 - 1.0*share_ISL)*((0.445983379501385 - 0.445983379501385*share_ISL)*(191.8*(9.87890071579491e-10*mining_electricity_switch_dieselgenerator + 1.91183079402386e-9*mining_electricity_switch_grid)*select([less(ore_grade, 0.01),True], [(-0.0723*log(100*ore_grade)**2/log(10)**2 + 0.98)**(-1.0),1.02040816326531], default=nan)*exp(-0.482*ore_grade) + 4.5788560030172e-8*select([less(ore_grade, 0.01),True], [(-0.0723*log(100*ore_grade)**2/log(10)**2 + 0.98)**(-1.0),1.02040816326531], default=nan)*exp(-0.482*ore_grade) + 7.28410272776341e-7)/(1.0 - 1.0*share_ISL) + (0.554016620498615 - 0.554016620498615*share_ISL)*(221.9*(9.87890071579491e-10*mining_electricity_switch_dieselgenerator + 1.91183079402386e-9*mining_electricity_switch_grid)*select([less(ore_grade, 0.01),True], [(-0.0723*log(100*ore_grade)**2/log(10)**2 + 0.98)**(-1.0),1.02040816326531], default=nan)*exp(-0.176*ore_grade) + 5.29743559473158e-8*select([less(ore_grade, 0.01),True], [(-0.0723*log(100*ore_grade)**2/log(10)**2 + 0.98)**(-1.0),1.02040816326531], default=nan)*exp(-0.176*ore_grade) + 1.22218225897971e-6)/(1.0 - 1.0*share_ISL) + 1.98637636944006e-6 + 0.0258*(9.87890071579491e-10*milling_electricity_switch_dieselgenerator + 1.91183079402386e-9*milling_electricity_switch_grid)/ore_grade + 8.33029606228744e-10/ore_grade) + 4.18600113377616e-7)/(1.219800704306 + 0.0049*(2*rate_enrichment - 1)*log(rate_enrichment/(1 - rate_enrichment))/(rate_enrichment - 0.0022) - 0.0298418833469335/(rate_enrichment - 0.0022)))*(1.219800704306*rate_enrichment + 0.0049*(2*rate_enrichment - 1)*log(-rate_enrichment/(rate_enrichment - 1)) - 0.0325254448964067) + 5.22266326376196e-7)/(efficiency*(1090.0*rate_enrichment - 0.600000000000001)) + 1.14077116130504e-10*(13.8923747590794*construction_intensity + 0.220722951251088)/(availability*lifetime)
<lambdifygenerated-536>:2: RuntimeWarning: invalid value encountered in divide
  return 0.000879607210257476 + (1/24000)*(0.120978641909761*fuel_fab_elec + 202.040816326531*(0.208993767959516*enrichment_diff_elec + 28.1302383360455 + 1.0*(0.245406721024908*conversion_elec + 0.00228859202129525*conversion_heat + 1.04217599120411*share_ISL*(154.0*(0.00554785313997973*mining_electricity_switch_dieselgenerator + 0.0453845121725285*mining_electricity_switch_grid)*select([less(ore_grade, 0.01),True], [(-0.0723*log(100*ore_grade)**2/log(10)**2 + 0.98)**(-1.0),1.02040816326531], default=nan)*exp(-0.0485*ore_grade) + 0.404107269334488*select([less(ore_grade, 0.01),True], [(-0.0723*log(100*ore_grade)**2/log(10)**2 + 0.98)**(-1.0),1.02040816326531], default=nan)*exp(-0.0485*ore_grade) + 33.0972116406022) + 1.04217599120411*(1.0 - 1.0*share_ISL)*(3912365.95624308*tailings_Rn222*(1 - exp(-9.19537252003111e-6*integration_time_Rn222)) + (0.445983379501385 - 0.445983379501385*share_ISL)*(191.8*(0.00554785313997973*mining_electricity_switch_dieselgenerator + 0.0453845121725285*mining_electricity_switch_grid)*select([less(ore_grade, 0.01),True], [(-0.0723*log(100*ore_grade)**2/log(10)**2 + 0.98)**(-1.0),1.02040816326531], default=nan)*exp(-0.482*ore_grade) + 0.503297235443862*select([less(ore_grade, 0.01),True], [(-0.0723*log(100*ore_grade)**2/log(10)**2 + 0.98)**(-1.0),1.02040816326531], default=nan)*exp(-0.482*ore_grade) + 178.86950076078)/(1.0 - 1.0*share_ISL) + (0.554016620498615 - 0.554016620498615*share_ISL)*(221.9*(0.00554785313997973*mining_electricity_switch_dieselgenerator + 0.0453845121725285*mining_electricity_switch_grid)*select([less(ore_grade, 0.01),True], [(-0.0723*log(100*ore_grade)**2/log(10)**2 + 0.98)**(-1.0),1.02040816326531], default=nan)*exp(-0.176*ore_grade) + 0.582281838086512*select([less(ore_grade, 0.01),True], [(-0.0723*log(100*ore_grade)**2/log(10)**2 + 0.98)**(-1.0),1.02040816326531], default=nan)*exp(-0.176*ore_grade) + 1161.55454657382)/(1.0 - 1.0*share_ISL) + 4.12632384355364 + 0.0258*(0.00554785313997973*milling_electricity_switch_dieselgenerator + 0.0453845121725285*milling_electricity_switch_grid)/ore_grade + 0.0010411214736684/ore_grade) + 33.0119954271685)/(1.219800704306 + 0.0049*(2*rate_enrichment - 1)*log(rate_enrichment/(1 - rate_enrichment))/(rate_enrichment - 0.0022) - 0.0298418833469335/(rate_enrichment - 0.0022)))*(1.219800704306*rate_enrichment + 0.0049*(2*rate_enrichment - 1)*log(-rate_enrichment/(rate_enrichment - 1)) - 0.0325254448964067) + 24.0670532444177)/(efficiency*(1090.0*rate_enrichment - 0.600000000000001)) + 1.14077116130504e-10*(152034843.173122*construction_intensity + 575589.2492842)/(availability*lifetime)
<lambdifygenerated-539>:2: RuntimeWarning: invalid value encountered in divide
  return 0.0987850017547607*river_cooling + 0.000576887378348076 + (1/24000)*(0.120297151746396*fuel_fab_elec + 202.040816326531*(0.128354031325489*enrichment_diff_elec + 71.3528214141035 + 1.0*(0.29298941455146*conversion_elec + 0.00237639492616074*conversion_heat + 1.04217599120411*share_ISL*(154.0*(0.00298224311975356*mining_electricity_switch_dieselgenerator + 0.112705922350704*mining_electricity_switch_grid)*select([less(ore_grade, 0.01),True], [(-0.0723*log(100*ore_grade)**2/log(10)**2 + 0.98)**(-1.0),1.02040816326531], default=nan)*exp(-0.0485*ore_grade) + 0.309108654872383*select([less(ore_grade, 0.01),True], [(-0.0723*log(100*ore_grade)**2/log(10)**2 + 0.98)**(-1.0),1.02040816326531], default=nan)*exp(-0.0485*ore_grade) + 95.7069888820048) + 1.04217599120411*(1.0 - 1.0*share_ISL)*((0.445983379501385 - 0.445983379501385*share_ISL)*(191.8*(0.00298224311975356*mining_electricity_switch_dieselgenerator + 0.112705922350704*mining_electricity_switch_grid)*select([less(ore_grade, 0.01),True], [(-0.0723*log(100*ore_grade)**2/log(10)**2 + 0.98)**(-1.0),1.02040816326531], default=nan)*exp(-0.482*ore_grade) + 0.38498077925015*select([less(ore_grade, 0.01),True], [(-0.0723*log(100*ore_grade)**2/log(10)**2 + 0.98)**(-1.0),1.02040816326531], default=nan)*exp(-0.482*ore_grade) + 41.7998217585077)/(1.0 - 1.0*share_ISL) + (0.554016620498615 - 0.554016620498615*share_ISL)*(221.9*(0.00298224311975356*mining_electricity_switch_dieselgenerator + 0.112705922350704*mining_electricity_switch_grid)*select([less(ore_grade, 0.01),True], [(-0.0723*log(100*ore_grade)**2/log(10)**2 + 0.98)**(-1.0),1.02040816326531], default=nan)*exp(-0.176*ore_grade) + 0.445397470884298*select([less(ore_grade, 0.01),True], [(-0.0723*log(100*ore_grade)**2/log(10)**2 + 0.98)**(-1.0),1.02040816326531], default=nan)*exp(-0.176*ore_grade) + 29.8451224584082)/(1.0 - 1.0*share_ISL) + 52.7642935163433 + 0.0258*(0.00298224311975356*milling_electricity_switch_dieselgenerator + 0.112705922350704*milling_electricity_switch_grid)/ore_grade + 0.00371804927536702/ore_grade) + 18.3907182562588)/(1.219800704306 + 0.0049*(2*rate_enrichment - 1)*log(rate_enrichment/(1 - rate_enrichment))/(rate_enrichment - 0.0022) - 0.0298418833469335/(rate_enrichment - 0.0022)))*(1.219800704306*rate_enrichment + 0.0049*(2*rate_enrichment - 1)*log(-rate_enrichment/(rate_enrichment - 1)) - 0.0325254448964067) + 14.2908542532349)/(efficiency*(1090.0*rate_enrichment - 0.600000000000001)) + 1.14077116130504e-10*(129846906.578373*construction_intensity + 1089640.83194551)/(availability*lifetime)
<lambdifygenerated-537>:2: RuntimeWarning: invalid value encountered in divide
  return 0.00505692382821041 + (1/24000)*(0.527854381819618*fuel_fab_elec + 202.040816326531*(0.65711431644277*enrichment_diff_elec + 100.203906410342 + 1.0*(0.577795780936465*conversion_elec + 0.0159469405318573*conversion_heat + 1.04217599120411*share_ISL*(154.0*(0.170495603413036*mining_electricity_switch_dieselgenerator + 0.10274921860281*mining_electricity_switch_grid)*select([less(ore_grade, 0.01),True], [(-0.0723*log(100*ore_grade)**2/log(10)**2 + 0.98)**(-1.0),1.02040816326531], default=nan)*exp(-0.0485*ore_grade) + 12.2837545140374*select([less(ore_grade, 0.01),True], [(-0.0723*log(100*ore_grade)**2/log(10)**2 + 0.98)**(-1.0),1.02040816326531], default=nan)*exp(-0.0485*ore_grade) + 328.371731950703) + 1.04217599120411*(1.0 - 1.0*share_ISL)*((0.445983379501385 - 0.445983379501385*share_ISL)*(191.8*(0.170495603413036*mining_electricity_switch_dieselgenerator + 0.10274921860281*mining_electricity_switch_grid)*select([less(ore_grade, 0.01),True], [(-0.0723*log(100*ore_grade)**2/log(10)**2 + 0.98)**(-1.0),1.02040816326531], default=nan)*exp(-0.482*ore_grade) + 15.2988578947556*select([less(ore_grade, 0.01),True], [(-0.0723*log(100*ore_grade)**2/log(10)**2 + 0.98)**(-1.0),1.02040816326531], default=nan)*exp(-0.482*ore_grade) + 251.907876301475)/(1.0 - 1.0*share_ISL) + (0.554016620498615 - 0.554016620498615*share_ISL)*(221.9*(0.170495603413036*mining_electricity_switch_dieselgenerator + 0.10274921860281*mining_electricity_switch_grid)*select([less(ore_grade, 0.01),True], [(-0.0723*log(100*ore_grade)**2/log(10)**2 + 0.98)**(-1.0),1.02040816326531], default=nan)*exp(-0.176*ore_grade) + 17.699773549772*select([less(ore_grade, 0.01),True], [(-0.0723*log(100*ore_grade)**2/log(10)**2 + 0.98)**(-1.0),1.02040816326531], default=nan)*exp(-0.176*ore_grade) + 95.8023759060552)/(1.0 - 1.0*share_ISL) + 389.532968103831 + 0.0258*(0.170495603413036*milling_electricity_switch_dieselgenerator + 0.10274921860281*milling_electricity_switch_grid)/ore_grade + 0.149808252120606/ore_grade) + 58.6211867812304)/(1.219800704306 + 0.0049*(2*rate_enrichment - 1)*log(rate_enrichment/(1 - rate_enrichment))/(rate_enrichment - 0.0022) - 0.0298418833469335/(rate_enrichment - 0.0022)))*(1.219800704306*rate_enrichment + 0.0049*(2*rate_enrichment - 1)*log(-rate_enrichment/(rate_enrichment - 1)) - 0.0325254448964067) + 72.8574885027346)/(efficiency*(1090.0*rate_enrichment - 0.600000000000001)) + 1.14077116130504e-10*(1206150153.65082*construction_intensity + 1592990884.93782)/(availability*lifetime)
```

In [120]:

```
sns.set(font_scale=.8)
fig, axes = plt.subplots(3,3,figsize=(16,9))

sensitivity.fillna(method='ffill', inplace=True)
sensitivity_diff.fillna(method='ffill', inplace=True)
delta = sensitivity_diff-sensitivity

for i,impact in enumerate(sensitivity):
    ax=axes[i//3,i%3]
    sensitivity[impact].plot(ax=ax)
    sensitivity_diff[impact].plot(ax=ax)
    pd.concat([sensitivity[impact], delta[impact]],axis=1).plot.area(ax=ax, color=((0,0,0,0),(.5,.5,.5,.5)),legend=False)
    ax.set_ylabel(ind_norm_wrap_short[i])
    ymin, ymax=ax.get_ylim()
    ax.set_ylim((0,ymax*1.1))
#     ax.legend('')

plt.tight_layout()
```

In [ ]:

```
problem, params, Y = stats._stochastics(
        elec_prod_p, impacts, n=1000,
        var_params=None, sample_method=stats.StochasticMethod.SALTELLI)
```

In [ ]:

```

```
