# Supplementary figures and images for: Parametric Life Cycle Assessment of Nuclear Power for Simplified Models
Source: Environ Sci Technol. 2023 Sep 12;57(38):14194–205. doi: 10.1021/acs.est.3c03190 (PMC10537461; doi:10.1021/acs.est.3c03190)

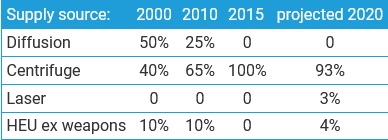

Supplement: Supplementary file 4 — es3c03190_si_004.zip [file es3c03190_si_004.zip › images/enrichment_market_shares.png]

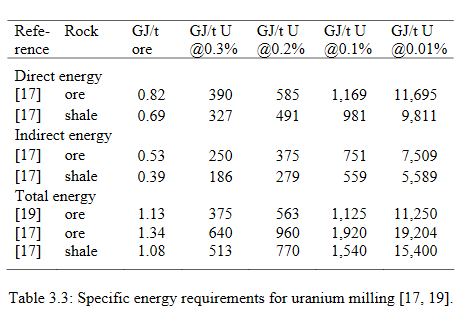

Supplement: Supplementary file 4 — es3c03190_si_004.zip [file es3c03190_si_004.zip › images/milling1.png]

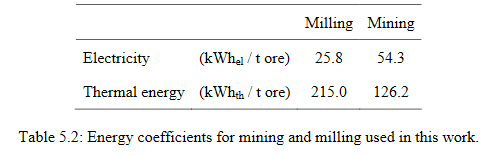

Supplement: Supplementary file 4 — es3c03190_si_004.zip [file es3c03190_si_004.zip › images/milling2.png]

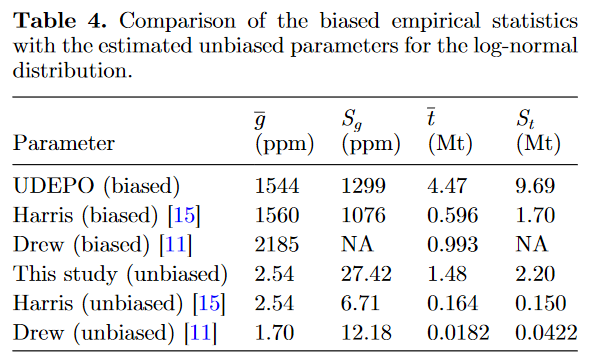

Supplement: Supplementary file 4 — es3c03190_si_004.zip [file es3c03190_si_004.zip › images/ore_grade.png]

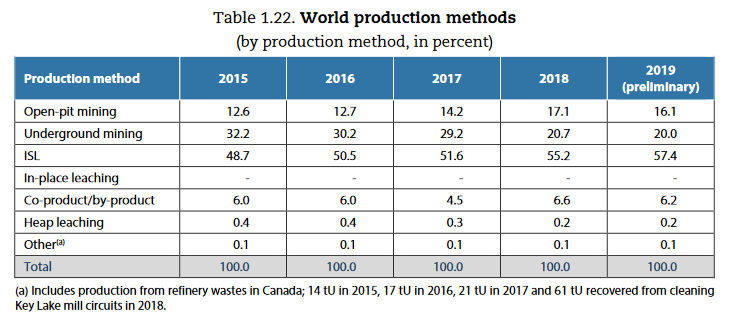

Supplement: Supplementary file 4 — es3c03190_si_004.zip [file es3c03190_si_004.zip › images/techniques.png]

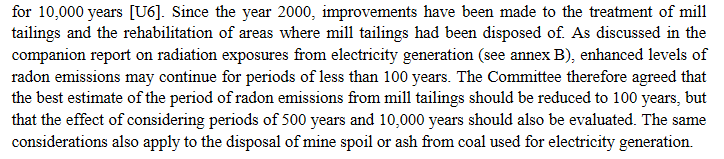

Supplement: Supplementary file 4 — es3c03190_si_004.zip [file es3c03190_si_004.zip › images/unscear_quote.png]
